# Implementation and Outcomes of a Comprehensive Type 2 Diabetes Program in Rural Guatemala

**DOI:** 10.1371/journal.pone.0161152

**Published:** 2016-09-01

**Authors:** David Flood, Sandy Mux, Boris Martinez, Pablo García, Kate Douglas, Vera Goldberg, Waleska Lopez, Peter Rohloff

**Affiliations:** 1 Wuqu’ Kawoq | Maya Health Alliance, Santiago Sacatepéquez, Sacatepéquez, Guatemala; 2 Medicine-Pediatrics Residency Program, University of Minnesota, Minneapolis, Minnesota, United States of America; 3 Internal Medicine Residency Program, Saint Peter’s University Hospital, New Brunswick, New Jersey, United States of America; 4 Harvard Medical School, Boston, Massachusetts, United States of America; 5 Division of Global Health Equity, Brigham and Women’s Hospital, Boston, Massachusetts, United States of America; Florida International University Herbert Wertheim College of Medicine, UNITED STATES

## Abstract

**Background:**

The burden of chronic, non-communicable diseases such as diabetes is growing rapidly in low- and middle-income countries. Implementing management programs for diabetes and other chronic diseases for underserved populations is thus a critical global health priority. However, there is a notable dearth of shared programmatic and outcomes data from diabetes treatment programs in these settings.

**Program Description:**

We describe our experiences as a non-governmental organization designing and implementing a type 2 diabetes program serving Maya indigenous people in rural Guatemala. We detail the practical challenges and solutions we have developed to build and sustain diabetes programming in this setting.

**Methods:**

We conduct a retrospective chart review from our electronic medical record to evaluate our program’s performance. We generate a cohort profile, assess cross-sectional indicators using a framework adapted from the literature, and report on clinical longitudinal outcomes.

**Results:**

A total of 142 patients were identified for the chart review. The cohort showed a decrease in hemoglobin A1C from a mean of 9.2% to 8.1% over an average of 2.1 years of follow-up (p <0.001). The proportions of patients meeting glycemic targets were 53% for hemoglobin A1C < 8% and 32% for the stricter target of hemoglobin A1C < 7%.

**Conclusion:**

We first offer programmatic experiences to address a gap in resources relating to the practical issues of designing and implementing global diabetes management interventions. We then present clinical data suggesting that favorable diabetes outcomes can be attained in poor areas of rural Guatemala.

## Introduction

The global burden of chronic, non-communicable diseases (NCDs) like diabetes is growing rapidly [1]. In fact, every country with consistent data has observed increasing numbers of people with type 2 diabetes [2]. Four-fifths of the world’s estimated 380 million people with diabetes live in low- and middle-income countries (LMICs) [2,3]. Adjusted diabetes prevalence rates are higher in poorer countries [4], and rates are also rising sharply in rural areas of LMICs [5]. Individuals with diabetes in LMICs tend to be younger than those in high-income countries [3] and face disproportionate premature mortality [6]. At the same time, adequate medical care for chronic diseases like diabetes care is often limited or unavailable in many LMICs [7].

This study focuses on diabetes in Guatemala, an LMIC in Latin America with a population of approximately 16 million people. The most robust data on the diabetes burden in Guatemala come from a 2006 survey showing an adjusted adult diabetes prevalence of 9.6%, which was similar to the U.S. prevalence when the study was conducted [8]. Subsequent statistical modeling exercises using this underlying data have estimated diabetes prevalence rates in Guatemala to be 10.9% or higher [9,10]. An important limitation of the 2006 survey was that it was conducted in a single urban municipality with few indigenous inhabitants [11]. Gaps in knowledge therefore remain regarding the burden of diabetes in rural, indigenous Maya communities that comprise approximately 50% of the population [12]. Additionally, over time, point prevalence rates may understate the rising absolute diabetes burden in Guatemala where the population is growing rapidly [13].

### Designing and Implementing Diabetes Programs for in LMICs

Given the epidemiologic data above, implementing high-quality clinical management programs for diabetes and other chronic diseases for populations in LMICs like Guatemala is a critical public health priority [14]. However, there is a significant gap in the global diabetes literature relating to the design, financing, and implementation of comprehensive diabetes treatment programs in LMICs—in particular rural areas of LMICs.

Many factors limit access to good diabetes care in LMICs [7]. For example, in Guatemala, due to limitations in public-sector funding and capacity, the majority of health care for chronic diseases like diabetes is financed by household spending and delivered outside the public health system [15, 16]. In this context, improving diabetes outcomes requires not only strengthening the public sector, but also meeting the urgent needs of patients who seek care in the private sector, which is the dominant domain for diabetes care in Guatemala and many other LMICS. We therefore view the delivery of global chronic disease care as a design space in which private and non-governmental organizations (NGOs) must innovate and share programmatic experiences.

### Study objectives

This study reports on the design, implementation, and evaluation of an adult type 2 diabetes program serving indigenous populations in rural Guatemala. Our objectives are twofold. First, we share design and implementation experiences with our adult type 2 diabetes management program and, second, we conduct a chart review to assess our program’s clinical outcomes. The overarching goal is that this study is to assist fellow global health practitioners in implementing high-quality diabetes clinical programming tailored to other low-resource settings in LMICs.

## Methods

### Institutional Context

The work in this study was conducted through our affiliation with Wuqu’ Kawoq | Maya Health Alliance (www.wuqukawoq.org), an NGO operating health programs in indigenous Maya communities in the rural central highlands of Guatemala since 2007. This study was conducted in accordance with the principles expressed in the Declaration of Helsinki, and it was reviewed and approved by the Institutional Review Boards of Wuqu’ Kawoq | Maya Health Alliance (WK-2015-002) and Harvard Medical School (IRB15-0735). Both IRBs approved waiving informed consent from participants after determining that the study posed minimal risk given its non-experimental chart-review design and that the research could not practicably be conducted without a waiver of consent, given the lack of contact information for many subjects and the need to analyze data from deceased and lost-to-follow-up cases.

### Site Description

The diabetes program operates in four central highland towns in rural Guatemala. These towns are predominantly Maya indigenous in terms of culture and language, possess poverty rates ranging from 60–85% [17], and have diabetes care available in both the public and private sectors. Public health centers offer free monthly glucose monitoring and distribute oral anti-diabetic pills, but services are severely impacted by long wait times, frequent supply stock-outs, and inability to offer routine measurement of hemoglobin A1C or insulin management. Large public hospitals in urban areas are primarily utilized for acute diabetes-related complications and are difficult to access for rural patients. In the private health sector, numerous private, physician-operated clinics treat individuals with diabetes and dispense diabetes medications in these towns. Private providers are popularly viewed to be more responsive, but care quality is highly heterogeneous and expensive such that patients drop in and out of treatment as finances permit [18].

### Program Overview

#### Program History and Prior Research

We previously conducted formative research on patients with diabetes presenting to our primary care clinics [19]. We found that poor glycemic control and significant end-organ complications were common. Patients reported that diabetes medicines were prohibitively expensive, family support was low, and medical care was usually sought with multiple competing providers on an intermittent and primarily fee-for-service basis. Patients expressed limited biomedical knowledge about the causes, treatments, and long-term complications of diabetes. Most people with diabetes lacked Spanish language fluency and expressed preference for Mayan languages.

#### Program Design

The program provides ambulatory diabetes management and coordinates referrals for acute care and specialty medical appointments. All services are free, including reimbursement for transportation expenses. The program’s primary diabetes providers are bilingual (Mayan and Spanish) nurses. Nurse-directed care was implemented because physician density is very low in rural Guatemala [20], and there is a surplus of experienced, bilingual nurses. Clinical visits with each patient are conducted at least once every three months. A staff physician is on call for emergencies and evaluates each patient at least once every six months. Clinics are conducted in NGO-owned facilities or donated community center space. Table 1 outlines some of the challenges, opportunities, and solutions for implementation of diabetes programming in our setting.

**Table 1:** Challenges, Opportunities, and Solutions for Implementation of Diabetes Programs in Guatemala.

Challenge	Opportunities	Solution
Cultural and linguistic barriers to biomedical care for indigenous Maya population	Rising number of educated young Maya professionals experienced in issues related to language, cultural, and health advocacy	Employ exclusively native speakers of Mayan languages as front-line health providers
Low physician density	Large labor pool of indigenous nurses	Nurse-driven diabetes protocols
High cost of diabetes medications	Dynamic generics industry in Guatemala	Limited formulary of locally purchased generic drugs
Low availability of laboratory diagnostics	Validated point-of-care tests available on the local market	Use of point-of-care laboratory testing for hemoglobin A1C
Patients live in rural, difficult-to-access villages	• Availability of open-source electronic medical record platforms • Extensive, reliable cellular network coverage throughout country	Deployment of smart-phone-based data entry and open-source electronic medical record
Low levels of education and health literacy	• Patients often work from home or return to home during day • Culture in which home visits are normalized	Home visit program by diabetes educator using locally adapted curriculum
Patients present late in disease course, often with significant end-organ damage	Excellent subspecialty care available in capital city	Centralized case management system to coordinate referrals from rural health workers to pre-selected subspecialty clinics
Chronic disease care is expensive and requires long-term commitments to beneficiaries	Blended financing models are emerging in global health	• Crowdfunding provides funding for extraordinary and catastrophic care • Grant-based fundraising allows for exploration of new areas of clinical innovation • Cross-training of primary care staff allows for coverage of core salary obligations from general operating funds

#### Clinical Details

Diabetes nurses manage glycemic treatment using a step-wise clinical algorithm developed from national [21], regional [22], and international [23–25] diabetes guidelines. (See S1 File for our current clinical protocol). A primary challenge has been to adapt commonly accepted clinical standards to the rural Guatemalan setting where risk-reward tradeoffs are distinctive. For example, most international guidelines advocate lowering hemoglobin A1C to less than 7%. However, in our setting, there is minimal access to emergency services in the event of acute hypoglycemia and so we have therefore chosen a less strict glycemic goal of a hemoglobin A1C of 8% or less. Uncontrolled patients or those undergoing active insulin titration receive blood glucose self-monitoring equipment and test strips; however, due to their high cost, we do not dispense them to stable patients. Many patients using insulin do not have refrigeration in the home; in such cases, we dispense new vials of insulin each month. We screen and treat for hypertension, nephropathy, and peripheral neuropathy. We have carried out ophthalmologic referrals, microalbuminuria screening, and lipid management on an ad hoc basis, but these services are not as of yet protocolized for all patients.

#### Diabetes Education

New or uncontrolled patients are offered regular home education visits, which are conducted by a bilingual diabetes nurse educator using a curriculum adapted from the U.S. National Heart, Lung and Blood Institute’s *Salud Para Su Corazón* (Health for Your Heart) community health worker model for Latinos and adapted and validated by our team for use in Mayan-speaking populations [26–27]. The curriculum is available under Supporting Information (S2 File).

#### Laboratory testing and drug procurement

Our program formulary consists solely of generic medicines, including common oral diabetes drugs (metformin and glyburide), insulin (NPH and regular), and anti-hypertensive agents (ACE inhibitors, among others). Laboratory tests are carried out using point-of-care devices when feasible or in fee-for-service regional laboratories. We monitor glycemic control with point-of-care hemoglobin A1C testing; while this test is more expensive than blood glucose monitoring and not routinely used in rural Guatemala, we have found it to be an indispensible clinical tool.

#### Complex and Referral Care

Bilingual (Spanish-Mayan) caseworkers provide transportation, accompaniment, and interpretation for patients referred from rural communities to urban health facilities. Patients with acute diabetes-related complications are referred to national or regional public hospitals where caseworkers advocate for them. Patients with non-acute specialty medical needs are referred to public or private facilities with which we have developed working relationships.

#### Financing

Financial sustainability has been a central challenge given that diabetes patients require long-term commitments, per-patient costs rise over time as disease severity progresses, and the global funding landscape for diabetes programs is limited. Our core diabetes expenses are supported through donor fundraising. We partner with a popular global health crowdfunding platform, Watsi (www.watsi.org), to fund extraordinary care. Grant-based funding cycles are too short-term and unpredictable to be relied upon to fund chronic disease care, so we reserve grants for research and programmatic innovation. Estimates from institutional balance sheets place the cost of the program at approximately $220 per patient per year. Although a formal cost analysis is outside the scope of this paper, three general observation include that: (1) per-patient costs vary significantly depending on disease severity; (2) human capital for service provision and not generic medicines or other consumables (including insulin) is the primary overall cost driver; and (3) program nurses, who are paid approximately $7500 per year, provide high value for cost.

### Chart Review

In our retrospective chart review, we identified active adult type 2 diabetes patients who had been enrolled in the diabetes program for at least 6 months as of 1 May 2015. We defined an “active patient” as having had at least one clinical encounter documented in the one-year period from 5/1/2014-5/1/2015. The electronic medical record (EMR) utilized in our program is OpenMRS (http://openmrs.org/), a popular open-source medical informatics platform.

Our pre-defined search inclusion criteria included age ≥18 years and diabetes diagnosis as defined by a hemoglobin A1C ≥6.5, random blood glucose ≥200 mg/dL, history of a diabetes-related prescription (metformin, sulfonylurea, or insulin), or assignment to the diabetes module or problem list in the EMR. A total of 236 hits were initially generated. Each record was then manually reviewed to remove patients meeting exclusion criteria: residence in a non-program community, no visit documented in the defined time period, program enrollment < 6 months, diagnosis of type 1 or gestational diabetes, and erroneous EMR entries. A final list of 142 patients was identified. Data on these patients were manually extracted from the EMR to a spreadsheet, and separate authors reviewed the entire spreadsheet for errors. Variables extracted were demographic (date of birth, gender, preferred language, years of education, municipality of residence), historical (years with diabetes diagnosis, date of program enrollment), and clinical (hemoglobin A1C, systolic and diastolic blood pressures, height and weight, creatinine, proteinuria, medication prescriptions, frequency of encounters).

### Data Analysis

Chart review data was imported from a spreadsheet into Stata version 13 (College Station, TX) for statistical analysis. A demographic and clinical profile of the cohort was first generated using descriptive statistics. Subsequently, to assess cross-sectional outcomes, we adapted a framework for use of electronic heath records in evaluating quality of diabetes care in LMICs [28]. Into this framework, we added indicators we considered to be highly relevant in our context and clinical workflow (history of home visits, nephropathy screening with serum creatinine, appropriate clinical intensity as defined by four or more clinical visits per year, and proportion of patients on insulin); removed other indicators that reflected elements of care that our program either cannot yet regularly offer or which could not be accurately assessed in the chart review (ophthalmology referral, serial BMI measurements, lipid management); and adapted other indicators based on clinical goals defined *a priori* in our clinical protocols (hemoglobin A1C goal <8%, blood pressure goal <140/90 mmHg). To assess retrospective program outcomes for the cohort identified in the chart review, we compared initial and most recent mean hemoglobin A1C and blood pressure values. We also compared the proportion of patients who met the program’s glycemic and blood pressure goals. We tested for significance using paired-sample t-test for continuous variables (hemoglobin A1C, blood pressure) and McNemar’s test for the proportion of patients meeting the clinical goals.

## Results

### Cohort profile

A cohort profile including demographics, history of diabetes and program enrollment, and basic clinical data is outlined in Table 2. Key findings included that the cohort consisted predominantly of women, that most patients expressed preference for a Mayan language over Spanish, and that education levels were low. Patients had a median time since diabetes diagnosis of seven years (IQR 4–12) and were diagnosed at a mean age of 47.2 ± 11.7 years. As of 5/1/2015, the 142 patients comprising the cohort had been enrolled our diabetes program for a median of 2.5 years (ICR 1.3–3.8).

**Table 2:** Demographic profile of Type 2 Diabetes Cohort.

Characteristic	Value
*Age–years (n = 142)*	56.1 ± 11.8
*Female–% (n = 142)*	80.3
*Language preference–% (n = 130)*	
Kaqchikel Mayan	50.8
Spanish	37.7
K’iche’ Mayan	11.5
*Education (n = 130)*	
Grades completed, median (IQR)	2 (0–4)
Completed primary school–%	20.8
*Time with diabetes diagnosis–years (n = 130)*	
Median (IQR)	7 (4–12)
*Age at diagnosis*, *years (n = 130)*	47.2 ± 11.7
*Time enrolled in program–years (n = 142)*	
Median (IQR)	2.5 (1.3–3.8)

For continuous variables with normal distribution, values are given as mean ± standard deviation. For continuous variables with nonnormal distribution, median and interquartile range (IQR) are specified. Some non-clinical data including preferred language, years with diabetes, and education attained were not available for all patients, as indicated by *n* in parentheses.

Relevant clinical indicators, summarized in Table 3, included an average hemoglobin A1C of 8.1 ± 2.1. Nearly half of the cohort (45.8%) carried a diagnosis of hypertension. The mean BMI was 28.0 ± 5.0; the majority of patients had an abnormal BMI (BMI ≥ 25), and over 30% met criteria for obesity (BMI ≥ 30). Kidney disease was common with over 40% of patients having an abnormal glomerular filtration rate (GFR ≤ 60) and a third of patients having gross dipstick proteinuria.

**Table 3:** Clinical profile of Type 2 Diabetes Cohort.

Clinical Characteristic	Value
*Mean hemoglobin A1C –%*	8.1 ± 2.1
*Hypertension*	
Diagnosis of hypertension–%	45.8
Systolic BP, mean–mmHg	121.8 ± 20.4
Diastolic BP, mean–mmHg	74.9 ± 10.2
*Body mass index*	
Mean	28.0 ± 5.0
BMI ≥ 25 –%	70.8
BMI ≥ 30 –%	30.8
*Diabetic nephropathy indicators*^*a*^	
GFR 30–60 –%	40.1
GFR ≤ 30 –%	3.5
Proteinuria–%	33.6
On dialysis–%	2.1
*Medication prescriptions–%*	
Metformin	85.9
Sulfonylurea	44.4
Insulin NPH	25.4
Insulin regular	2.8
No insulin or oral anti-diabetic agent	5.0
ACE inhibitor	43.7
*Number of diabetes-related encounters in last 12 months*	
Median	11.5
Interquartile range	8–15

BP, blood pressure; BMI, body mass index, GFR, glomerular filtration rate.

^a^ GFR was estimated from clinical variables using the CKD-EPI equation.

An overview of medication prescriptions revealed that metformin was the most commonly prescribed drug (85.9%), that approximately one-quarter of patients had been prescribed insulin, and that 44% of patients were prescribed an ACE inhibitor. Only 5.0% of patients were not prescribed any oral diabetes medicine or insulin. The median number of diabetes-related encounters per patient per year was 11.5 (IQR 8–15).

### Cross-sectional indicators

Cross-sectional indicators are displayed in Table 4. In terms of process of care outcomes over the year ending on 5/1/2015, nearly all patients (99%) had at least one A1C measurement, and 95% of the cohort had four or more visits during the year. One-half of the cohort received a home education visit. The vast majority of patients who met clinical indications for metformin and an ACE inhibitor were prescribed these drugs. However, only 38% of patients who had uncontrolled blood sugars as defined by the program’s target hemoglobin A1C ≥ 8% were receiving insulin.

**Table 4:** Cross-sectional outcomes indicators for Type 2 Diabetes Cohort.

**I. Process of care**	%
*A*. *Timely detection of type 2 diabetes complications and comorbidity in the last year (n = 142)*	
At least one measurement of hemoglobin A1C	99.3
Comprehensive foot evaluation	98.6
Measurement of creatinine and rate of glomerular filtration	90.1
Four or more clinical encounters during year	95.1
*B*. *Non-pharmacological treatment in the last year (n = 142)*	
Diabetes self-care education provided in home visit	50
*C*. *Pharmacological treatment in the last year*	
Overweight/obese (BMI ≥25 kg/m^2^) patients with hemoglobin A1C ≥ 6.5 who received metformin, unless contraindicated (n = 66)	98.8
Patients with hemoglobin A1C ≥ 8% who received insulin (n = 64)	37.5
Patients with hypertension receiving inhibitors of angiotensin converting enzyme or angiotensin-receptor blocker, unless contraindicated (n = 65)	95.4
**II. Health outcomes**	
Hemoglobin A1C < 8% in last measurement (n = 142)	54.9
Blood pressure <140/90 mmHg in last 3 measurements (n = 139)	59.0
Hemoglobin A1C < 8% in last measurement and blood pressure <140/90 mmHg in last 3 measurements (n = 139)	29.5

In terms of health outcomes, 57% of the cohort met the program’s glycemic goal of hemoglobin A1C < 8% at their last recorded measurement, and 59% had well-controlled blood pressures at the most recent three measurements. Overall, 29.5% of patients met the composite indicator of both hemoglobin A1C < 8% in last measurement and blood pressure <140/90 mmHg in last 3 measurements. Of note, 32.4% of patients had a most recent hemoglobin A1C < 7%.

### Longitudinal outcomes indicators

Table 5 reports the mean clinical values and health outcomes indicators for patients in the chart review cohort at enrollment and at last recorded value. The median length of follow-up as defined by time between initial and last values was 2.1 (IQR 0.9–3.3) years for hemoglobin A1C and 2.4 (IQR 1.1–3.4) years for systolic and diastolic blood pressure.

**Table 5:** Longitudinal outcomes indicators for Type 2 Diabetes Cohort.

Characteristic (n = 142)	Initial	Last	p-value
*Raw clinical values*, *mean*			
Hemoglobin A1C –%	9.2 ± 2.4	8.1 ± 2.1	0.00
Systolic BP–mmHg	124.3 ± 20.0	121.8 ± 20.4	0.17
Diastolic BP–mmHg	77.6 ± 11.2	74.9 ± 10.2	0.02
*Health outcomes indicators–%*			
Percent of patients with hemoglobin A1C < 8%	38.0	54.9	0.001
Percent of patients with blood pressure <140/90 mmHg	69.7	73.2	0.46
Percent of patients with hemoglobin A1C <8% and blood pressure <140/90 mmHg	25.4	38.0	0.01

BP, blood pressure.

We found that the mean hemoglobin A1C in the cohort showed a statistically significantly decrease from 9.2% ± 2.4 on initial presentation to 8.1% ± 2.1 at the most recent measurement (p = 0.00). Mean blood pressure also decreased, though the difference was only significant for mean diastolic blood pressure (p = 0.02) and not systolic blood pressure (p = 0.17). We also observed a statistically significant increase in the proportion of patients who exhibited adequate glycemic control (p = 0.001), as well as the percent of patients meeting the composite goal of A1C <8% and blood pressure <140/90 mmHg (p = 0.01). No significant change was found in the percent of patients meeting blood pressure goals alone.

## Discussion

This study describes the design, implementation, and outcomes of a type 2 diabetes program for Maya indigenous adults in rural Guatemala.

In the first part of the paper, we offer programmatic experiences to address a gap in published accounts of the practical issues of designing and implementing diabetes management interventions in LMICs. Although there is an increasingly robust literature on diabetes prevention, screening, education, and self-management in global health settings [29–35], there is a notable lack of shared programmatic and outcome data from comprehensive diabetes management programs in LMICs. In addition, existing diabetes care guidelines tailored to resource-limited settings tend to emphasize clinical aspects of disease management and not discuss local implementation barriers [22–24]. Important exceptions include Partners In Health’s 2011 chronic care integration manual tailored to Rwanda [36], a recent systematic review on diabetes delivery models in resource-limited settings [37], and the programmatic experiences shared by various groups in Sub-Saharan Africa [38–42].

Several design and implementation experiences detailed in this paper may be generalizable to practitioners in other settings. First, in our program, nurses provide the bulk of direct patient care, education, and care coordination. In our context, we have found that there is a surplus of well-trained nurses who speak indigenous Maya languages, that well-supported nurses can provide high value diabetes care for their cost, and that nurses are motivated to carry out time-consuming, high-contact activities such as home visits, education sessions, and social work tasks. The model of “task-shifting” from to non-physician providers has been shown to be effective in other settings [40, 43–46]. Our experience adds to this literature suggesting that nurse-led interventions are a highly promising strategy for expanding quality diabetes care [47–48].

Our experience also reveals some of the practical challenges in adapting widely-accepted diabetes clinical standards to local environments where resources are limited and risk-reward trade-offs are unique. We have observed that the underlying assumptions of published international guidelines sometimes do not hold in our setting. An example of this is the use of hemoglobin A1C, a test that is recommended in all guidelines but is rarely used in rural Guatemala due to its expense and unavailability. Like Partners In Health’s experience in Rwanda [36], we have found this test to be essential to quality care and have worked through various implementation barriers to offer point-of-care testing to all of our patients.

Additionally, international diabetes guidelines typically recommend stricter glycemic targets of hemoglobin A1C ≤ 7.0. Not only does this recommendation assume that hemoglobin A1C testing can always be utilized, it also does not take into account the much greater risks of hypoglycemia in rural, isolated towns with limited emergency services compared to the high-income countries where the major diabetes clinical trials (such as the ACCORD [49] and UKPDS [50] studies) were carried out. Consequently, in our program, we have chosen a less strict glycemic target, though we acknowledge that this decision can and should be debated. Nevertheless, our experiences with hemoglobin A1C access and targets illustrate the gap between global diabetes guidelines and the implementation of diabetes programs on the ground.

Finally, we describe our blended diabetes financial model, consisting of operating funds from donations, crowdfunding, and grants. Financing is a primary challenge for our program given the limited funding environment for adult NCD services compared with infectious diseases and maternal and child health programs. Additionally, diabetes is an incurable disease that progressively worsens over time, and each patient we enroll requires a long-term financial commitment. Ultimately, providing high-quality diabetes care is expensive compared to other global health interventions. We attempt to keep costs down by utilizing non-physician (nurse) providers, restricting our formulary to generic drugs, and negotiating with our suppliers. A future research aim is to conduct a cost-effectiveness analysis to better elucidate the costs and benefits of our program.

In the second part of this paper, we conduct a retrospective chart review to examine program outcomes. In terms of clinical indicators, we found that the cohort showed a decrease in hemoglobin A1C from a mean of 9.2% to 8.1% over an average of 2.1 years of follow-up. The proportions of patients meeting glycemic targets were 57% for the less strict targets that we use programmatically (hemoglobin A1C < 8%) and 32% for the stricter threshold (hemoglobin A1C < 7%).

Our results appear favorable compared to other rural type 2 diabetes treatment programs in resource-limited settings. For example, a nurse-led program with 80 patients in Kwazulu Natal, South Africa found average hemoglobin A1C to be 10.8% at baseline, 8.4% at two years, and 9.7% at four years [40]. The VIDA program in Mexico reported that 39% of patients had a hemoglobin A1C less than 7.0% at the intervention’s conclusion [51]. A study in Western Kenya described a self-management program with insulin-dependent patients had a median hemoglobin A1C of 9.1% after 12 months [42]. Putting these figures in context, in the U.S., the most recent national-level data from the U.S. report that 63.3% of Mexican-American people with diabetes over 40 years of age had hemoglobin A1C of less than 8.0% [52].

Despite clear difficulties comparing our small cohort with data from other global health sites and national-level data in the U.S., we offer preliminary results suggesting that good diabetes outcomes can be attained in poor areas of rural Guatemala with a cohort of patients who predominantly speak Mayan indigenous languages, have low levels of education, and have high rates of overweight and obesity. We hypothesize that important drivers of favorable clinical outcomes in our context arise from a design process that has emphasized frequent contact and collaboration with patients and their families as indicated by the high frequency of patient interactions (95% of with four or more clinical visits during the year, median 11.5 encounters per year), our use of highly competent and motivated indigenous nurses as primary diabetes providers, and the high proportion of patients receiving home education visits (50% of patients).

Areas meriting additional investigation and quality improvement initiatives include an examination of the factors contributing to the gender disproportion in our cohort (80% female), further inquiry into dietary aspects such as food insecurity that may contribute to the high observed prevalence of obesity (30.8% with BMI ≥ 30), and better understanding barriers to insulin utilization since 38% of patients with uncontrolled diabetes were not prescribed insulin. On this final point, there is a large literature primarily originating from high-income countries relating to the fear of insulin, or “psychological insulin resistance” [53]. We are interested in studying this concept in the rural Guatemalan setting as, anecdotally, we have observed that poverty and a weak health system seem to produce traumatic patient experiences with insulin such as accidental life-threatening overdoses, incurrence of debts to purchase insulin vials and syringes, or insulin initiations in the last days of life [18–19]. Since in our program insulin is dispensed free of charge yet is not widely accepted by patients, this study suggests that achieving optimal insulin treatment in LMICs is not only an issue of access to insulin [54]. Finally, a detailed exploration of the process of adapting our diabetes clinical guidelines to the Maya population in rural Guatemala was beyond the scope of this paper but an important future direction for our research team. In fact, we are currently analyzing diabetes knowledge- and self-care outcomes in our cohort and plan to conduct, as part of that analysis, a more structure investigation of the process of cross-cultural guidelines adaptation.

This study has several weaknesses or limitations. First, our program and sample size is small and may not generalize to other institutional context or settings within Guatemala. Second, cost is a significant barrier to scaling-up diabetes programs in LMICs, yet we cannot at this time offer a robust cost- effectiveness analysis of our program. We are, however, able to use institutional balance sheets to estimate average cost/patient/year of $220. Third, retention is a critical aspect of diabetes treatment programs in global health, and it is especially important in settings like Guatemala where fragmented care is a fundamental feature of the experience of indigenous people with diabetes [18–19]. However, our study design of a retrospective chart review, with a relatively short follow-up interval, is as yet unable to offer longer-term insights into program retention rates and disease-related complications.

## Supporting Information

S1 File Diabetes Protocol (English and Spanish). Current protocol used in diabetes programming for Wuqu’ Kawoq | Maya Health Alliance.
(PDF)

S2 File Home Education Manual. Home education manual adapted from U.S. National Heart, Lung and Blood Institute’s *Salud Para Su Corazón (Health for Your Heart)* community health worker model for Latinos.
(PDF)
