# Supplementary material for: Implementation and Outcomes of a Comprehensive Type 2 Diabetes Program in Rural Guatemala
Source: PLoS One. 2016 Sep 1;11(9):e0161152. doi: 10.1371/journal.pone.0161152 (PMC5008811; doi:10.1371/journal.pone.0161152)
Supplement: S1 File — Current protocol used in diabetes programming for Wuqu’ Kawoq | Maya Health Alliance. (PDF) [file pone.0161152.s001.pdf]

PROTOCOL FOR DIABETES MANAGEMENT IN ADULTS, VERSION 2.0

16 June 2014

1. Program Personnel

This protocol is designed with the following personnel roles in mind:

Table: Program personnel

Title	Responsibilities
Diabetes clinical nurse	Responsible for the day-to-day management of the diabetes program. Serves patients in monthly diabetes clinics in each community. Fields calls relating to urgent issues (e.g., critical hypoglycemia or serious foot lesion) and non-urgent issues (e.g., medication logistics or coordinating routine laboratory examinations).
Diabetes nurse educator	Responsible for delivering education in serial home visits. Also helps to coordinate glucometer assignments, draw blood, or visit sick patients.
Diabetes coordinator	Responsible for the general coordination of patient care, protocol design, and non-urgent clinical consultations.
On-call physician	Responsible for all urgent clinical consultations.
Staff physicians	Responsible for carrying out clinical visits with each diabetes patient at least two times per year.

2. General program principles

2.1. Language

All clinical consultations should be conducted in the patient's language of preference. If a patient states that he or she is indifferent between a Mayan language and Spanish, the encounter should be conducted in the Mayan language.

2.2. Levels of support

The diabetes team always has two levels of support for any questions, problem, or emergencies that may arise.

- For non-urgent consultations, a call should be made or email sent to the "diabetes coordinator."
- For urgent consultations, a call should be made to the "on-call physician."

2.3. Frequency of patient visits

All patients should come to clinic each month to collect their medicines and/or have a consult with the diabetes nurse.

- “Complex” patients who meet any one of the following criteria must have a consult with the diabetes nurse each month.

Table: Patients who requiring a monthly clinical visits with diabetes clinical nurse

A1C ≥9.0
Injecting insulin
Uncontrolled hypertension (BP regularly ≥160/100)
Type 1 diabetes
History of serious diabetes complication (for example, on dialysis, TFG/GFR <45, previous serious foot infection, previous stroke or myocardial infarction)

- “Non-complex” patients who do not meet any of these criteria may collect their medicines monthly and have clinical visits every 3 months.

Note: Some patients may not be “complex” according to this definition but may still prefer to pass with the diabetes nurse each month. Every effort should be made to accommodate this request in order to foster the patient’s alliance with the diabetes program

2.9. Physician visits

In addition to clinical visits with the diabetes clinical nurse, each patient should have an appointment with a physician every six month to review medications, preform a cardiac examination, and manage complications.

2.10. Documentation

It is essential for the quality of the diabetes program to maintain good, up-to-date medical records and patient information. There are several elements to consider.

- First, document all clinical encounters in OpenMRS the day of the encounter. Also, update the current medication of the “Regimen” page in OpenMRS.

- Second, provide all patients with a diabetes health tracking card, which should be updated at each clinical encounter. This card should include clinic telephone numbers and instructions to call if any question or issue arises.

- Third, make sure that at least one telephone number for reaching the patient is in OpenMRS; if it is possible, having two phone numbers is better.

3. Diagnosis of diabetes

Patients must meet one of the following criteria to be diagnosed with diabetes.

Table: Diabetes diagnosis

**Test**	**Result**
Fasting blood glucose	≥126 mg/dl
Random blood glucose	≥200 mg/dl if there are symptoms of hyperglycemia (blurry vision, thirst, urinary frequency, weight loss)
A1C	≥ 6.5%

A test result diagnosing diabetes should be repeated on a different day to confirm the result, unless the diagnosis is also clear from the clinical presentation. Due to the relatively high cost of the A1C test, it is generally better to first use a glucose test to screen for diabetes in a newly presenting patient.

4. Enrollment criteria

There is not enough space for the many diabetes patients who wish to join the diabetes program. The following general rules can assist the diabetes team on case-by-case decisions regarding enrollment of new patients. New patients should also agree to try their best to attend monthly appointments, permit home education visits with the diabetes educator, and adhere to their medication regimen.

4.1. New "complex" patients

We can always enroll complex diabetic patients in its communities. Diabetes patients are considered complex if they have a history of lower limb amputation, on-going kidney disease or dialysis, previous stroke or heart attack, BMI <20, age <30 years, type I diabetes, or random glucose >500.

4.2. New "non-complex" diabetes patients

We cannot always enroll non-complex diabetes patients in its communities. Such patients will be enrolled when space becomes available, as communicated by the diabetes coordinator. The diabetes clinical nurse should keep a list of patients' names and numbers who have asked to join the program and use this list to enroll new patients when availability permits.

5. The initial consult

5.1. Components of initial visit

The first visit of a new diabetes patient should include the following elements:

Table: Tasks to complete during the initial visit

Obtain a medical history<br>• Years with diabetes<br>• Previous and current medication(s) and diabetes regimen(s)<br>• Family members with diabetes<br>• History of any diabetes complications or hospitalizations<br>• Other medical conditions<br>• If there is regular alcohol consumption
Preform a foot exam
Check for protein in the urine
Check for ketones in the urine
Blood glucose test
Glycosylated hemoglobin test (A1C)
Serum creatinine
Blood pressure
BMI (obtain weight and height)

5.2. Initial visits requiring immediate consultation with a physician

The initial visit is an important time to detect patients who may have complicated, severe, or abnormal cases. The diabetes clinical nurse should contact the on-call physician before initiating treatment for patients with any of the following:

Table: New patients who require immediate consultation

A1C >12.0
BMI <25
Ketones in urine
Age <30 years
History of any severe diabetes complication (for example, lower limb amputation, chronic kidney disease, stroke, or heart attack)
Pregnancy
Type 1 diabetes

6. The follow-up visit

In general, follow-up visits should include the following components:

Table: Tasks to complete during the follow-up visit

Task	Frequency
Review medications	Every visit
Foot exam	Every visit
Check for protein in the urine	See guidelines below
Blood glucose test	If the patient is on insulin, one must check blood glucose. If the patient is not taking insulin, a blood glucose test is not required but can be done at the patient's request.
A1C test	See guidelines below
Serum creatinine	See guidelines below
Blood pressure	Every visit
BMI (weight and height)	Every 6 months

7. Glycemic Control

7.1. Glycosylate hemoglobin (A1C)

In general, glycosylated hemoglobin is the key test to interpret each patient's glycemic control. However, if the fasting blood glucose is greater than 200 mg/dl, the A1c is not very useful because we already know that the A1C will be very high. In the majority of patients who have a fasting blood glucose <200mg/dl and who do not have symptoms of hypoglycemia, you should not make major decisions about medication management without considering the results of the A1C test.

7.2. A1C goal

The "A1C goal" is a way of thinking about a patient's ideal blood sugar control. The A1C goal might be different for different patients, depending on age, previous complications, and quality of life. Also, it is important to remember that there can be a trade-off between better glucose control and higher risk of negative effects such as hypoglycemia.

Below are the A1C goals in our clinics:

- The general A1C goal for patients is between 6.5-8.0.
- However, patients who (1) are less than 40 years of age or (2) have been diagnosed less than 5 years ago may have a stricter A1C goal of 6.5-7.0.
- In cases of elderly (>65 years old) or frail patients, A1C values between 8.0-9.0 may be acceptable.

7.3. Frequency of A1C measurement

Some patients require more frequent A1C testing than other patients. The general guidelines for A1C monitoring are:

- Every 3 months if patient is poorly controlled, has had a recent medication change, has type 1diabetes, or has a history of a serious diabetes complication.

- Every 3-6 months for stable patients who are meeting treatment goals.

8. Medications

The medications used in our clinic to control diabetes are the following:

Table: Our diabetes formulary

Medication	Dosage	How it works	Side effects	Notes
Metformin 850 mg tablet	0-3 tablets each day (divided in 2-3 doses daily)	(1) Decreases production of glucose in the liver and (2) increases insulin sensitivity in the tissues	• **Gastrointestinal side effects are common** (metallic taste in the mouth, diarrhea, nausea/vomiting, abdominal pain)<br>• Lactic acidosis	**See rules below for use in patients with kidney disease.**
Glibenclamide (also known as glyburide) 5 mg tablet	0-3 tablets each day (divided in 1-2 doses daily)	Increases insulin release from the pancreas	• **Hypoglycemia**	Glibenclamide y glimepiride are both in the "sulfonylurea" class of medicines.
Glimepiride 2 mg tablet	0-2 tablets each day	Increases insulin release from the pancreas	• **Less risk of hypoglycemia than with glibenclamide**	
Regular Insulin ("rapid" or "crystalline") (100 U/ml)	1-3 times a day with meals	Replaces the body's prandial insulin production	• Weight gain<br>• Hypoglycemia	Effect peaks at 2-4 hours and lasts 5-8 hours.
NPH Insulin (100 U/ml)	1-2 times a day before breakfast and/or after dinner	Replaces the body's basal insulin production	• Weight gain<br>• Hypoglycemia	Effect peaks at 4-8 hours and lasts 12-18 hours.

9. Treatment with oral hypoglycemic pills

9.1. Treatment of new patients

Table: Initial treatment guidelines

Initial A1C	Initial treatment
<7.0	None
7.0-8.0	Metformin 850mg daily (breakfast)
>8.0	Metformin 850mg BID (breakfast and dinner)

For new patients, metformin can be started without a creatinine on record as long as there is a plan to get a creatinine within 1 week of initiation of therapy.

9.2. Treatment of follow-up patients

Using the step-wise algorithm below, oral medication should be adjusted upward or downward to the patient's A1C goal. A patient's regimen should generally not be adjusted more than 2 steps from one month to the next without notifying the diabetes coordinator.

Table: Step-wise algorithm for dosing hypoglycemic drugs

Step	Dosage
Step 1	metformin 850mg daily (breakfast)
Step 2	metformin 850mg BID (breakfast and dinner)
Step 3	metformin 850mg BID (breakfast and dinner) and glibenclamide 5mg daily (at lunch)
Step 4	metformin 850mg BID (breakfast and dinner) and glibenclamide 10mg daily (at lunch)
Step 5	metformin 850mg TID (at each meal) and glibenclamide 10mg daily (at lunch)
Step 6	metformin 850mg TID (breakfast and dinner) and glibenclamide 7.5mg BID (breakfast and dinner)

9.3. Metformin in patients with chronic kidney disease

There is a risk of lactic acidosis, a serious side effect, in patients taking metformin who have severely decreased kidney function. However, the risk of lactic acidosis is very small, and in general, metformin is the safest, most effective oral medicine for diabetes.

The general guidelines for using metformin with decreased renal function are:

- If the GFR of a patient taking metformin falls below 45, confirm the GFR with a second creatinine within 1 month. The general guidelines are:

GFR	What to do?
30-45	Reduce the metformin dose to a maximum of 1.5 pills (1275 mg) per day
<30	Stop all metformin

- Before stopping or reducing the metformin dosage, one must have a plan to control blood sugar. For example, in some cases, it might be necessary to start insulin or increase the dose of insulin with a patient who will no longer be taking the same dose of metformin. In other cases, the patient can be well controlled with only glibenclamide. It is not acceptable to stop metformin without a plan to control blood sugar.

- After, inform the diabetes coordinator of the changes if it was necessary to reduce or stop metformin due to renal function

9.4. Use of glibenclamide versus glimepiride

As shown above, glibenclamide and glimepiride are both medications in the "sulfonylurea" class. Drugs in this class work by increasing the amount of insulin produced in the patient's pancreas. Thus, patients who take these medications are at risk for hypoglycemia if too much insulin release is stimulated. Due to this risk of hypoglycemia, all patients who are taking glibenclamide or glimepiride should be asked about symptoms of hypoglycemia at every encounter.

Glibenclamide is the first option over glimepiride as it works for most patients and is much more affordable than glimepiride. However, the risk of hypoglycemia is higher with glibenclamide than glimepiride.

What do to if a patient reports a reliable history of hypoglycemia that appears to be caused by glibenclamide?

- First, provide education about the importance of frequent small meals in reducing risk of hypoglycemia.

- Second, if hypoglycemic episodes persist, try to control patient's blood sugar with a lower dose of glibenclamide. For example, the dose of metformin can be raised and the dose of glibenclamide can be lowered.

- Third, glimepiride can be offered in place of glibenclamide if a patient is well-controlled and has repeated, symptomatic hypoglycemia uncorrected by education.

How should glimepiride be used in practice?

- In our diabetes program, dosing of glimepiride is either 2mg or 4mg once daily at lunch.

- It is important to remember that glibenclamide is NOT more effective than glimepiride in reducing blood sugar. Patients with uncontrolled A1C should be switched to insulin, not glimepiride. That is, switching to glimperide is not an effective alternative to insulin.

- The diabetes coordinator or on-call doctor should be notified when a patient is switched from glibenclamide to glimepiride.

10. Treatment with insulin

The natural course of type 2 diabetes is that oral medicines become less effective as the function of the pancreas declines. Thus, many patients with years of type 2 diabetes will require treatment with insulin.

10.1. Indications for insulin therapy

Table: Indications for insulin

Type 1 diabetes
Maximum oral therapy fails to achieve A1C goal
Kidney dysfunction prohibiting use of metformin and glibenclamide
Pregnancy (in some cases)

10.2. General principles of starting insulin

- Unless a new patient presents already injecting insulin, new patients should never be started on insulin at the initial visit without consulting with the on-call physician or diabetes coordinator.

- Patients who are started on insulin should be called or visited at home within 24 hours of initiating insulin treatment. This encounter should be documented in OpenMRS.

- Rapid insulin should only be initiated or adjusted in consultation with the diabetes coordinator or the on-call physician.

- Unless the patient has type 1 diabetes, if possible, metformin should be continued in addition to the insulin. On the other hand, a patient should never take both insulin and a sulfonulyrea (glibenclamide or glimepiride).

10.3. Insulin titration

When insulin is indicated, the following are guidelines for dosing:

- Step 1: Start NPH insulin 10 units before breakfast. Stop glibenclamide and continue metformin.

- Step 2 (next visit): Add NPH insulin 5 units before dinner to the NPH insulin 10 units before breakfast.

- Step 3 (next visit): Adjust the NPH insulin regimen using the table below. In general, when indicated, the size of the dose increase should be small (2 units), and the size of the dose decrease can be slightly larger (4 units).

Table: NPH insulin titration

**Timing of hyperglycemia or hypoglycemia**	**Hyperglycemia**	**Hypoglycemia**
Morning or middle of night	Increase evening NPH insulin by 2 units	Decrease evening NPH insulin by 4 units
Mid-day or evening	Increase morning NPH insulin by 2 units	Decrease morning NPH insulin by 4 units

10.4. Glucose data

All patients with uncontrolled A1C who are undergoing insulin adjustment should have a glucometer at home to measure blood glucose for 3 consecutive days each month.

- If a patient is injecting insulin only in the morning, he/she should measure and record the blood glucose before lunch and before dinner (2 times per day).

- If a patient is injecting insulin in the morning and evening, he/she should measure and record blood glucose before breakfast, before lunch, before dinner, and at 10:00 p.m. (4 time per day).

10.5. Education about insulin

- Patients should be regularly reminded on proper injection technique. Injections should be given into an area of pinched skin, with the needle inserted perpendicularly up to the hilt (thus into the subcutaneous tissue). The best sites of injection are in the abdomen, but other sites include the upper arms, the thighs, and buttocks. Using the same site may help with consistent absorption.

- At each visit, all patients injecting insulin should be taught about the danger signs of hypoglycemia and instructed to have a snack readily available in the home in case of hypoglycemia.

- All patients should be instructed on the importance of not fasting, not missing meals, or going extended periods of time without eating. If possible, the best diet consists of small, frequent meals.

10.6. Insulin storage

- Whether opened or unopened, all types of insulin expire in 1 month if unrefrigerated. If a refrigerator is unavailable, insulin should be stored in a cool place out of direct sunlight. Patients who do not have a refrigerator will need new insulin vials every month.

- If stored in a refrigerator, unopened bottles are good until the expiration date printed on the bottle.

10.7 Insulin use when a patient is sick

When patients who inject insulin are sick and cannot eat, give them instructions to inject ½ of their normal dose of NPH insulin and to not inject Regular Insulin. Diabetes sick with the flu or diarrhea also have a higher risk of hyperglycemia and hypoglycemia.

11. Critical situations

Critical situations involving hyperglycemia and hypoglycemia will arise in the diabetes program. Theses situations can be life-threatening and require immediate action. It is also important to educate patients about these risks at regular clinical visits.

11.1. Patients presenting or calling with possible critical hypoglycemia.

First, check glucose if possible. Inquire about danger signs as shown in the table below. Ask about any recent consumption of alcohol.

Table: Danger signs of hypoglycemia

Sweating
Tremors
Dizziness
Anxiety
Agitation
Confusion
Palpitations
Lethargy

- If the patient can take in liquids or eat, instruct the patient to have an adequate snack to raise the blood sugar. The best snack for patient with hypoglycemia will include first a carbohydrate with rapid action (see below), followed by a carbohydrate with slow action (like fruits, vegetables, or tortillas). Stay in touch with patient until symptoms resolve. If the patient's symptoms do not quickly improve, send the patient to the health center or hospital and call the physician-on-call.

Table: Examples of carbohydrates with rapid action and appropriate portions

4 ounces of canned juice
4 ounces of soda
1 tablespoon of honey or sugar
2-3 small candies

- If patient cannot take in liquids or eat, send the patient to the health center or hospital immediately and call the on-call physician.

Always instruct the patient's family to inform all emergency medical providers that the patient is a diabetic whose blood sugar may be low.

11.2. Patients presenting or calling with possible critical hyperglycemia

- First, check the blood glucose and urine ketones, if possible. If there are ketones in the urine, this is a sign that there might not be enough insulin in the body. It may be that the patient has a class of diabetes more like diabetes type 1 than diabetes type 2. Patients with ketones in the urine also can have an emergency called diabetes ketoacidosis that requires hospitalization.

- Second, call on-call physician if (1) ketones are positive in urine or (2) any of the following warning signs of dangerous hyperglycemia are present:

Table: Danger signs of hyperglycemia

**Finding**	**Notes**
Signs of dehydration	Dry mouth, decreased urination
Low blood pressure	<90 systolic
Altered mental status	Agitation, disorientation, sleepiness, coma

12. Foot Care

12.1. Regular foot care

The clinical diabetes nurse should conduct a diabetes foot exam at every visit. If there are toenail infections (tinea pedis), the nurse should treat it with Clotriplex cream.

In addition, the nurse should give education about proper foot care, including:

- Wash feet daily

- Cut toenails each week very carefully

- Use appropriate shoes that are not too small

- Call us if an ulcer develops

12.2. Acute ulcer care

If a patient presents or calls with an ulcer, it is important to first know the severity of the ulcer.

- If the ulcer is small and only involves the skin, take a photo and send it the diabetes coordinator within 24 hours. Also, cover the wound with gauze to protect it. Give education about examining the foot daily, the importance of alleviated pressure in the foot, and what to do if the wound worsens.

- If there is a more severe ulcer, take a photo and call the physician-on-call immediately.

12.3. Foot care kit

The diabetes clinical nurse and the nurse educator should always bring a small bag of supplies for foot care to all clinics and home visits. The bag should include Vaseline, gloves, 4x4 gauze, medical tape, alcohol, and a small bottle of saline solution.

13. Watching out for nephropathy

13.1. Serum creatinine

All patients should have creatinine checked at the initial visit and at subsequent intervals as follows:

Table: Frequency of creatinine screening

Glomerular filtration rate (GFR)	Frequency of creatinine measurements
>50	Once per year
30-50	Every 6 months
<30	Depends on the case; must communicate with the diabetes coordinator

Always use the CKD-EPI calculator to calculate GFR with each patient that has a creatinine > 1.0. Record the GFR in OpenMRS.

13.2. Protein in the urine

All patients should have urine checked for protein using a dipstick at least once per year. Results should be managed as follows:

- A patient who has 1+ or greater proteinuria should have urine checked again in 3-6 months. After two readings of 1+ or more proteinuria within 6 months, if the patient is not already taking enalapril and has a GFR >30, start 5mg enalapril daily. Remember, a creatinine must have been checked within the previous year before starting enalapril.

- Review immediately and consult the diabetes coordinator for any patients with new findings of 2+ or greater proteinuria.

14. Blood pressure management

Blood pressure control is a vital part of adequate management of diabetes, as hypertension can contribute to worsening diabetes complications in the eyes, kidneys, and blood vessels.

14.1. Diagnosis of high blood pressure

- 1 reading of ≥160 systolic or ≥110 diastolic

- 2 readings of 140-160/ 90-110 at least a month apart

14.2. Goal blood pressure

The general goal blood pressure in diabetes patients is <140/90.

14.3. Management of acute hypertension

- If a patient has no symptoms but has BP ≥180 systolic or ≥120 diastolic, consult with the diabetes coordinator.

- If patient has any of the below danger signs and has BP ≥180 systolic or ≥120 diastolic, call the on-call doctor.

Table: Danger signs of hypertensive emergency

Severe, new headache
Blurry vision
Leg swelling
Chest pain
Acute change in mental status

14.4. Anti-hypertensive medications

Our formulary consists of the following oral medications for treating hypertension:

Table: Our anti-hypertensive formulary

Agent	Usual doses	Side effects
Enalapril 20mg	2.5-20mg	Cough, worsens kidney function, angioedema (a type of swelling more common in the lips, tongue, and face)
Amlodipina 5mg	5-10mg	Leg swelling
HCTZ 25mg	12.5-25mg	Hyperglycemia
Atenolol 100mg	25-100mg	Bradycardia, fatigue, sexual dysfunction

14.5. Management of chronic hypertension

The following table shows the steps for treating hypertension. In general, a patient's regimen should not be adjusted more than 1 step from one month to the next without informing the diabetes coordinator.

Table: Stepwise management of chronic hypertension

Step	Action	Note
Step 1 after diagnosis of hypertension	Start 5mg enalapril once daily	Before starting enalapril, must have previous creatinine test with GFR > 30 within 1 year
Step 2 if BP still ≥140/90 at next month's visit	Increase enalapril to 10mg once daily	Check creatinine and potassium between 3-14 days after changing enalapril if the patient's GFR is less than 50. With the higher enalapril dose, contact the diabetes coordinator if (1) the creatinine increases ≥30% or (2) the potassium level is outside the normal range of 3.5-5.0 mEq/L.
Step 3 if BP still ≥140/90 at next month's visit	Increase enalapril to 20g once daily	Check creatinine and potassium between 3-14 days after changing enalapril if the patient's GFR is less than 50. With the higher enalapril dose, contact the diabetes coordinator if (1) the creatinine increases ≥30% or (2) the potassium level is outside the normal range of 3.5-5.0 mEq/L
Step 4 if BP still ≥140/90 at next month's visit	Add amlodipine 5mg once daily	
Step 5 if BP still ≥140/90 at next month's visit	Increase amlodipine to 10mg once daily	
Step 6 if BP still ≥140/90 at next month's visit	Consult diabetes coordinator before starting third agent	

14.6. Side effects of enalapril

Enalapril is an excellent medicine for diabetics because it helps to protect the kidneys. However, enalapril has some side effect that we should consider.

- It can decrease renal function. Enalapril can cause an increase in creatinine that is more worrisome in patients with chronic kidney disease. For this reason, when we increase the dose to 10mg or 20mg daily in patient with GFR <50, we must check creatinine and potassium within 3-14 days.

- Cough. Enalapril can cause chronic cough in some patients. The benefits of enalapril are significant, so before stopping enalapril for the side effect of cough, we should be very confident in several visits that enalapril is the true cause of the cough.

- Angioedema. Enalapril can cause angioedema, a type of swelling that is more common in the lips, tongue, and face. This is a dangerous side effect, and the diabetes clinical nurse should call the on-call-physician immediately if a case of angioedema is suspected.

14.7. Education for diabetes with hypertension

Also, we should provide education to patients with chronic hypertension about the importance of reducing salt intake, exercising regularly, and reducing alcohol intake.

15. Diabetes education

15.1. In the clinic

Many studies have shown that education is an important part of delivering quality diabetes care. Every clinical encounter is an opportunity for the our staff to deliver education.

14.8. In the home

In addition, all patients who have not met A1C goal should be referred for serial home visits conducted by the diabetes nurse educator.

16. Eye treatment

Diabetics have a high risk of cataract and retinopathy. The diabetes nurse should maintain a list of patients with cataracts and severe vision problems for the revision of the diabetes coordinator.

17. Microalbuminuria

Measuring and managing microalbuminuria is important to detect the first signs of renal damage.

17.1. Logistical details:

- Use Ultralab for this urine test
- Ask the patient for a minimum of 40 mL of urine
- Urine can be kept for 7 days if it is refrigerated; thus, we can gather all the samples and bring them to Ultralab at the same time.

17.2. Details about clinical management

Clinically, it is important to manage the albumin to creatinine ratio (ACR) with a random urine sample:

**Albumin to creatinine ratio (ACR)**	**Results**	**What to do**
<30 mg/g	negative	nothing
>30 mg/g	positive	Repeat the test in 3-6 months

All patients need 1 microalbumin test. **A second test is not necessary if the patient is already taking enalapril.**

Rules for managing results:

- First microalbumin result:
    - ACR <30: Do not do anything
    - ACR ≥30: Must repeat in 3 months if the patient is not taking enalapril

- Second microalbumin result:
    - ACR <30: Do a third microalbumin test
    - ACR ≥30: Begin enalapril 5mg daily (If GFR >30)

- Third microalbumin result:
    - If 2/3 of the ACR results are <30: Do not do anything
    - If 2/3 of the ACR results are ≥30: Begin enalapril 5mg daily

PROTOCOLO PARA EL MANEJO DE DIABETES EN ADULTOS, VERSIÓN 2.0

16 junio 2014

1. Personal del programa

Este protocolo se ha diseñado con lo siguiente personal en mente:

Tabla: El personal del programa

**Cargo**	**Responsabilidades**
Enfermera clínica de la diabetes	Responsable del manejo del día a día del programa de diabetes. Atiende a pacientes en clínicas de diabetes mensuales en cada comunidad. Recibe llamadas de pacientes en cuestiones urgentes (por ejemplo, la hipoglucemia crítica o lesiones graves en los pies) y las cuestiones no urgentes (por ejemplo, la logística de los medicamentos o la coordinación de exámenes de laboratorio).
Enfermera educadora de la diabetes	Responsable de la entrega de la educación en las visitas a domicilio. También ayuda a coordinar asignaciones del glucómetro, hacer la extracción de sangre, o visitar a los pacientes enfermos.
Coordinador de programa de la diabetes	Responsable de la coordinación general de atención, el diseño del protocolo, y las consultas clínicas que no son urgentes.
Médico de turno	Responsable de todas las consultas clínicas urgentes.
Médicos generales	Responsable de realizar las visitas clínicas de cada paciente de la diabetes por lo menos dos veces al año.

2. Principios generales del programa

2.1. Idioma

Toda el personal de diabetes debe atender a pacientes en el idioma de la preferencia del paciente. Si un paciente dice que él o ella es indiferente entre un idioma maya y español, el encuentro debe realizarse en el idioma maya.

2.2. Los niveles de apoyo

El equipo de la diabetes siempre tiene dos niveles de apoyo para cualquier pregunta, problema, o emergencias que puedan surgir.

- Para consultas no urgentes, debe hacer una llamada o un correo electrónico enviado al “coordinador de la diabetes.”

- Para consultas urgentes, debe hacer una llamada al “médico de turno.”

2.3. Frecuencia de las visitas de los pacientes

Todos los pacientes deben llegar a la clínica cada mes para recoger sus medicamentos y / o tener una consulta con la enfermera de la diabetes.

- Pacientes “complicados” que cumplan cualquiera de los siguientes criterios deben tener una consulta con la enfermera clínica de la diabetes cada mes.

Tabla: Los pacientes que requieren una consulta mensualmente

A1C ≥9.0
La inyección de la insulina
La hipertensión no controlada (BP ≥160/100)
Diabetes tipo 1
Historia de una complicación grave de la diabetes (por ejemplo, en diálisis, TFG/GFR <45, infección grave del pie, derrame o infarto de corazón)

- Pacientes “no complicados” que no cumplan con alguno de estos criterios pueden recoger sus medicamentos mensualmente y tener una consulta cada 3 meses.

Nota: Algunos pacientes pueden ser “no complicados” según esta definición, pero todavía quieren pasar con la enfermera de la diabetes cada mes. Se debe hacer todo lo posible para acomodar esta solicitud con el fin de fomentar la alianza de la paciente con el programa de diabetes.

2.9. Visitas con el médico

Además de las consultas con la enfermera clínica, cada paciente debe tener una cita con un médico cada 6 meses para revisión de medicamentos, revisión cardiaca, y manejo de complicaciones.

2.10. Documentación

Es esencial para la calidad del programa de diabetes mantener bien el record médico y datos de los pacientes. Hay varios elementos a considerar.

- En primer lugar, documentar todos los encuentros clínicos en OpenMRS el día del encuentro. También, actualizar los medicamentos actuales en la página de “régimen” en OpenMRS.

- En segundo lugar, entregar un carnet de la diabetes a todos los pacientes, que debe ser actualizado en cada encuentro clínico. Se debe incluir números telefónicos de la clinica en el carnet y instrucciones para llamar si viene una cuestión o asunto.

- En tercer lugar, asegurarse que hay por lo menos un número de celular del paciente en OpenMRS; si es posible, tener 2 números sería mejor.

3. La diagnosis de la diabetes

Los pacientes con uno de los siguientes criterios pueden ser diagnosticados con diabetes.

Tabla: Diagnosis de la diabetes

Prueba	Resultado
Prueba de glucemia en ayunas	≥126 mg/dl
Prueba de glucemia al azar	≥200 mg/dl si hay síntomas de hiperglucemia (vista borrosa, sed, orina frecuente, perdida de peso)
Prueba de A1C	≥ 6.5%

Un resultado que da la diagnosis de la diabetes debe ser repetido en un día diferente para confirmar el resultado, a menos que el diagnóstico también es claro de la presentación y historia clínica. Debido al costo relativamente alto de la prueba A1C, es generalmente mejor utilizar primero una prueba de glucosa para detectar la diabetes en un paciente que presenta nuevamente.

4. Los criterios de inclusión del programa

No hay espacio suficiente para todos los pacientes con diabetes que quieren participar en el programa de diabetes. Las siguientes normas generales pueden ayudar al equipo de la diabetes en las decisiones de aceptar nuevos pacientes. Los nuevos pacientes también deben estar de acuerdo de hacer todo lo posible para acudir a las citas mensuales, aceptar las visitas de educación en el hogar, y intentar a adherir a su régimen de medicación.

4.1. Pacientes nuevas "graves"

Siempre pueden aceptar a los diabéticos graves en sus comunidades. Se considera tal si tiene antecedentes de amputación de miembros inferiores, muy alto creatinina o diálisis, derrame o ataque cardiaco, IMC <20, de edad <30 años, diabetes tipo I, o glucosa al azar >500.

4.2. Pacientes "no graves"

Debido a recursos, no siempre puede aceptar a los pacientes de diabetes no complejos en sus comunidades. Estos pacientes serán inscritos cuando el espacio esté disponible, según lo comunicado por el coordinador de la diabetes. La enfermera de la clínica de la diabetes debe mantener una lista de nombres y números que hayan solicitado participar en el programa y utilizar esta lista para inscribir a nuevos pacientes cuando la disponibilidad lo permite.

5. La primera consulta

5.1. Componentes de la primera visita

La primera visita de un nuevo paciente con diabetes debe incluir los siguientes elementos:

Tabla: Tareas para hacer durante la visita inicial

Tomar historia del caso<br>• Años con diabetes<br>• Régimen previo y actual de medicinas<br>• Familiares con diabetes<br>• Historia de las complicaciones de la diabetes o las hospitalizaciones<br>• Otras condiciones médicas<br>• Si hay el consumo regular de alcohol
Revisión de los pies
Revisión de proteína en la orina
Revisión de cetonas en la orina
Prueba de glucosa
Prueba de hemoglobina glicosilada (A1C)
Prueba de creatinina
Revisión de presión arterial
IMC (peso y talla)

5.2. Visitas iniciales que requieren una consulta inmediata con un médico

La primera visita es momento importante para detectar a los pacientes que pueden tener los casos complicados, graves o anormales. La enfermera de la clínica de la diabetes debe comunicarse con el médico de turno antes de iniciar el tratamiento para los pacientes nuevos con cualquiera de los siguientes:

Tabla: Pacientes nuevos que necesitan una consulta inmediata con un médico

A1C >12.0
IMC <25
Cetonas en orina
Edad <30 años
Historia de una complicación grave de la diabetes (por ejemplo, amputación de pie, enfermedad renal crónica, derrame o infarto de corazón)
Embarazo
Diabetes tipo 1

6. Visitas de seguimiento

En general, las visitas de seguimiento deben incluir los siguientes componentes:

Tabla: Tareas para completar durante las visitas de seguimiento

**Tarea**	**Frecuencia**
Revisión de régimen	Cada consulta
Revisión de los pies	Cada consulta
Revisión de proteína en la orina	Ver normas abajo
Prueba de glucosa	Si el paciente está inyectándose la insulina, siempre hay que realizar una prueba de glucosa. Si el paciente no está inyectándose la insulina, no se requiere una prueba de glucosa, pero se puede realizar si el paciente la pida.
Prueba de A1C	Ver normas abajo
Prueba de creatinina	Ver normas abajo
Revisión de presión arterial	Cada consulta
IMC (peso y talla)	Cada 6 meses

7. Control de glucemia

7.1. La hemoglobina glicosilada (A1C)

Por lo general, la hemoglobina glicosilada es la prueba clave para la interpretación del control de cada paciente. Sin embargo, si la prueba de glucemia en ayunas esta mayor de 200 mg/dl, la A1C no es muy útil porque ya sabemos que la A1C va a salir muy alto. En la mayoría de pacientes que tienen la glucemia en ayunas <200 mg/dl y que no tienen síntomas de hipoglucemia, no se deben tomar decisiones mayores sobre el manejo de medicamentos sin considerar los resultados de la A1C.

7.2. Meta de A1C

La “meta de A1C” es una manera de pensar en el control ideal de azúcar en la sangre de un paciente. La meta de A1C podría ser diferente para los distintos pacientes, dependiendo de la edad, las complicaciones anteriores, y la calidad de vida. Además, es importante recordar que puede haber una elección entre el mejor control de la glucosa y un mayor riesgo de efectos negativos como la hipoglucemia.

Abajo son los metas de A1C en la clínica:

- El meta general de A1C para los pacientes es entre 6.5-8.0%.

- Sin embargo, los pacientes que (1) tienen menos de 40 años de edad o (2) se han diagnosticado menos de 5 años pueden tener un meta de A1C más estricta de 6.5-7.0%.

- En los casos de gente más de 65 años o pacientes frágiles, los valores de A1C entre 8.0-9.0% pueden ser aceptables.

7.3. Frecuencia de medir A1C

Algunos pacientes requieren pruebas de A1C más frecuentes que otros pacientes. Las prácticas generales de la frecuencia de la prueba de A1C son:

- Cada 3 meses, si el paciente no se controla bien, ha tenido un cambio de medicación reciente, tiene diabetes tipo 1, o tiene un historial de una complicación de la diabetes grave.

- Cada 3-6 meses para los pacientes estables que están cumpliendo los objetivos del tratamiento.

8. Medicamentos

Los medicamentos que se usan en la clínica para control de la diabetes son los siguientes:

Tabla: Medicinas para la diabetes en el formulario

Medicamento	Posología	¿Cómo funciona?	Efectos secundarios	Notas
Metformina 850 mg tableta	0-3 tabletas cada día (divididas en 2-3 tomas diarias)	(1) Disminuye la producción de glucosa en el hígado y (2) aumenta la sensibilidad a la insulina en los tejidos	• **Efectos secundarios gastrointestinales son comunes** (sabor metálico en la boca, diarrea, nauseas, vómitos, dolor abdominal)<br>• La acidosis láctica	**Ver las reglas abajo para su uso en pacientes con función renal disminuida.**
Glibenclamida 5 mg tableta	0-3 tabletas cada día (divididas en 1-2 tomas diarias)	Aumenta la liberación de insulina por el páncreas	• **Hipoglucemia**	Glibenclamida y glimepirida son en la misma clase de medicina, se llama “sulfonilureas”
Glimepirida 2 mg tableta	0-2 tabletas al día	Aumenta la liberación de insulina por el	• **Menor riesgo de hipoglucemia con glibenclamida que**	

		páncreas	**glimepirida**	
Insulina Regular ("rápida" o "cristalina") (100 U/ml)	1-3 veces al día con comidas	Sustituye a la producción de insulina prandial en el cuerpo	• Aumento de peso<br>• Hipoglucemia	Pico de efecto en 2-4 horas y tiene una duración de 5-8 horas.
Insulina NPH (100 U/ml)	1-2 veces al día, antes de desayuno y/o antes de la cena	Sustituye a la producción de insulina basal en el cuerpo	• Aumento de peso<br>• Hipoglucemia	Pico de efecto en 4-8 horas y tiene una duración de 12-18 horas.

9. El tratamiento con hipoglucémicos orales

9.1. Tratamiento de pacientes nuevas

Tabla: Guía de tratamiento para pacientes nuevos

A1C	Tratamiento
<7.0	Nada
7.0-8.0	Metformina 850 mg diario (desayuno)
>8.0	Metformina 850 mg BID (desayuno y cena)

Para los nuevos pacientes, la metformina se puede iniciar sin la creatinina en el record medico si haya un plan para conseguir una creatinina dentro de 1 semana después de empezar la terapia.

9.2. Tratamiento de pacientes de seguimiento

Usando el algoritmo abajo, se debe ajustar los medicamento hacia arriba o hacia abajo para el objetivo de A1C del paciente. Generalmente, el régimen de un paciente no se ajusta más o menos de 2 pasos de un mes al siguiente sin avisar al coordinador de la diabetes.

Tabla: El algoritmo para la dosificación de los hipoglucemiantes orales

Paso	Dosificación
Paso 1	metformina 850mg diario (desayuno)
Paso 2	metformina 850mg BID (desayuno y cena)
Paso 3	metformina 850mg BID (desayuno y cena) and glibenclamida 5mg diario (almuerzo)
Paso 4	metformina 850mg BID (desayuno y cena) and glibenclamida 10mg diario (almuerzo)
Paso 5	metformina 850mg TID (desayuno, almuerzo y cena) and glibenclamida 10mg diario (almuerzo)
Paso 6	metformina 850mg TID (desayuno, almuerzo y cena) and

	glibenclamida 7.5mg BID (desayuno y cena)

9.3. El uso de metformina en pacientes con función renal disminuida

Existe el riesgo de acidosis láctica, un efecto secundario grave, en pacientes que toman metformina que se han disminuido gravemente su función renal. Sin embargo, el riesgo de acidosis láctica es muy pequeño y, en general, la metformina es el medicamento oral más seguro y efectivo para la diabetes.

Las reglas generales del uso de metformina con función renal disminuida son:

- Si la TFG/GFR del paciente tomando metformina llega por debajo de 45, confirmar la TFG/GFR con una segunda creatinina dentro en 1 meses. Las reglas generales son:

TFG/GFR	Qué hacer?
30-50	Reducir la dosis de metformina hasta máximo de 1.5 pastillas (1275 mg) por día
<30	Quitar toda la dosis de metformina

- Antes de quitar o reducir la dosis metformina, hay que tener un plan para controlar el azúcar en la sangre. Por ejemplo, en algunos casos, tal vez hay que empezar insulina o aumentar la dosis de la insulina con una paciente que ya no está tomando la misma dosis de metformina. En otros casos, el paciente puede ser bien controlada con la glibenclamida sola. No es aceptable quitar metformina sin un plan de controlar el azúcar.

- Después, avisar al coordinador de diabetes de los cambios si hay que reducir o quitar la metformina debido a función renal.

9.4. El uso de glibenclamida y glimepirida

Como se ve arriba, la glibenclamida y glimepirida son medicamentos de la clase "sulfonilurea." Los medicamentos de esta clase aumenta la cantidad de insulina producida en el páncreas del paciente. Por lo tanto, los pacientes que toman estos medicamentos están en riesgo de hipoglucemia si se estimula la liberación excesiva de insulina. Debido a este riesgo de hipoglucemia, todos los pacientes que están tomando glibenclamida o glimepirida se les debe preguntar sobre los síntomas de la hipoglucemia en cada encuentro.

La glibenclamida es la primera opción sobre la glimepirida porque funciona para la mayoría de los pacientes y es mucho menos caro que la glimepirida. Sin embargo, es cierto que el riesgo de hipoglucemia es mayor con glibenclamida que glimepirida.

¿Qué hacer si un paciente habla de una historia confiable de la hipoglucemia causado por glibenclamida?

- En primer lugar, dar educación sobre la importancia de las comidas pequeñas frecuentes para reducir el riesgo de hipoglucemia.

- En segundo lugar, si persisten los episodios de hipoglucemia, tratar de controlar el azúcar en la sangre del paciente con una dosis más baja de la glibenclamida. Por ejemplo, la dosis de metformina se puede aumentar y la dosis de glibenclamida se puede bajar.

- En tercer lugar, puede substituir la glimepirida en el lugar de la glibenclamida si un paciente está bien controlado y ha tenido hipoglucemia sintomática varias veces sin corregir por la educación.

¿Cómo usar la glimepirida en la práctica?

- En el programa de diabetes, la dosificación de glimepirida es 2 mg o 4 mg una vez al día en el almuerzo.

- Es importante recordar que la glibenclamida no es más eficaz que la glimepirida en la reducción de azúcar en la sangre. Los pacientes con A1C incontrolada deben cambiar a la insulina, no glimepirida. Es decir, cambiar a glimepirida no es una alternativa eficaz a la insulina.

- El coordinador de la diabetes o el médico de turno deben ser notificados cuando un paciente se cambia de glibenclamida a la glimepirida.

10. Tratamiento con insulina

El curso natural de la diabetes tipo 2 es que con el tiempo los medicamentos orales son menos eficaces porque la función del páncreas empeora. Por lo tanto, muchos pacientes con años de diabetes tipo 2 se necesitan tratamiento con insulina.

10.1. Las indicaciones para el tratamiento con insulina

Tabla: Indicaciones para la insulina

Diabetes tipo 1
Terapia oral máxima y sin lograr la meta de A1C
Función renal disminuida prohibiendo use de metformina y glibenclamida
Embarazo (en algunos casos)

10.2. Principios generales de iniciar la insulina

- Los nuevos pacientes nunca deben comenzar con la insulina en la primera visita sin consultar con el médico de turno o el coordinador de la diabetes.

- Debemos llamar o visitar todos los pacientes que se han iniciado la insulina dentro de las 24 horas después de empezar sus inyecciones. Este encuentro se debe documentar en OpenMRS.

- Solamente ajustar insulina regular en consulta con el coordinador de la diabetes o el médico de turno.

- A menos que el paciente tiene diabetes tipo 1, si es posible, la metformina debe ser continuado, además de la insulina. Por otro lado, un paciente nunca debería tener tanto la insulina y un sulfonilurea (glibenclamida o glimepirida).

10.3. Ajustes de insulina

Abajo son guías para la dosificación de insulina:

- Paso 1: Empezar la insulina NPH 10 unidades antes del desayuno. Quitar la glibenclamida y continuar la metformina.

- Paso 2 (siguiente visita): Agregar la insulina NPH 5 unidades antes de la cena a las 10 unidades de insulina NPH antes del desayuno.

- Paso 3 (siguiente visita): Cambiar el régimen de NPH utilizando la tabla abajo. En general, cuando se indica, la magnitud del aumento de la dosis debe ser pequeña (2 unidades), y la magnitud de la disminución de la dosis puede ser un poco más grande (4 unidades).

Tabla: Dosificación de insulina NPH

**Hora de la hiperglucemia o hipoglucemia**	**Hiperglucemia**	**Hipoglucemia**
Medio de la noche o la madrugada	Aumentar la dosis de la insulina NPH en la noche por 2 unidades	Bajar la dosis de la insulina NPH en la noche por 4 unidades
Medio día o antes de la cena	Aumentar la dosis de la insulina NPH en la mañana por 2 unidades	Bajar la dosis de la insulina NPH en la mañana por 4 unidades

10.4. Datos de glucosa

Todos los pacientes con A1C no controlado quien se hacen una intensificación de su dosis de la insulina deben tener un glucómetro en la casa para medir glucosa por 3 días consecutivos cada mes.

- Si un paciente se inyecta sólo en la mañana, debe medir la glucosa antes del almuerzo y antes de la cena (2 veces al día).

- Si un paciente se inyecta en la mañana y por la tarde, debe medir la glucosa antes del desayuno, antes del almuerzo, antes de la cena, y a las 10:00 de la noche (4 veces al día).

10.5. Educación sobre la insulina

- Los pacientes se les debe recordar periódicamente sobre la técnica adecuada de inyección. Las inyecciones deben administrarse en un área de piel pellizcada con aguja insertada perpendicularmente hasta en el tejido subcutáneo. Los mejores sitios de inyección están en el abdomen, pero otros sitios son los brazos, los muslos y las nalgas. Utilizando el mismo sitio puede ayudar a la absorción consistente.

- En cada visita, todos los pacientes que se inyectan insulina se debe enseñar acerca de las señales de peligro de la hipoglucemia y las instrucciones para tener una refacción disponible en el hogar en caso de hipoglucemia.

- Todos los pacientes deben ser instruidos sobre la importancia de no ayunar, no olvidarse a comer un tiempo, o ir largos tiempos sin comer. Si es posible, la mejor dieta consiste en comidas pequeñas y frecuentes.

10.6. Almacenamiento de la insulina

- Si abierto o no abierto, todos los tipos de insulina vence en 1 mes si no está refrigerada. Si un refrigerador no está disponible, se puede poner la insulina en un lugar fresco y sin la luz solar directa. Los pacientes que no tienen un refrigerador necesitarán nuevos frascos de insulina todos los meses.

- Si se almacena en un refrigerador, un frasco no abierto vence hasta la fecha de imprimido en la botella.

10.7. Uso de la insulina cuando un paciente se enferma

Cuando los pacientes que se inyectan insulina se enferman y no pueden comer, darles instrucciones para inyectar 1/2 de su dosis normal de la insulina NPH y no inyectar insulina Regular. Los diabéticos enfermos con gripes o diarreas también tienen un mayor riesgo de hiperglucemia y la hipoglucemia.

11. Situaciones críticas

Las situaciones críticas de la hiperglucemia y la hipoglucemia van a ocurrir en el programa de diabetes. Estas situaciones pueden ser potencialmente mortales y requieren una acción inmediata. También es importante educar a los pacientes sobre estos riesgos en las visitas clínicas regulares.

11.1. Visita o llamada de paciente con posible hipoglucemia crítico

Primero, verifique los datos glucosa si es posible. Pregunte acerca de los signos de peligro, como se muestra en el cuadro abajo. Pregunte acerca de cualquier consumo reciente de alcohol.

Tabla: Señales de peligro de la hipoglucemia

Sudores
Temblores
Mareos
Ansiedad
Agitación
Confusión
Palpitaciones
Cansancio marcado

- Si el paciente puede tomar o comer, diga a los pacientes a tener una refacción adecuada para aumentar el azúcar en su sangre. La mejor refacción para los pacientes con hipoglucemia incluirá primero un carbohidrato de acción rápida (ver abajo), seguido de un carbohidrato de acción lenta (como las frutas, verduras, o tortillas). Mantenga en contacto con el paciente hasta que los síntomas desaparezcan. Si los síntomas del paciente no mejoran rápidamente, mande al paciente al centro de salud u hospital y llamé al médico de turno.

  Tabla: Ejemplos de carbohidratos de acción rápida y su tamaño apropiados

4 onzas de jugo de lata
4 onzas de gaseosa
1 cucharada de miel o azúcar
2-3 dulces pequeñas

- Si el paciente no puede tomar y comer, mande al paciente al centro de salud u hospital inmediatamente y llame al médico de turno.

Siempre diga a la familia del paciente que informe a todos los proveedores de servicios médicos de emergencia que el paciente es diabético y posiblemente se bajó el azúcar en la sangre.

11.2. Visita o llamada de paciente con posible hiperglucemia crítico

- Primero, verifique los datos de glucosa en sangre y las cetonas en la orina, si es posible. Si hay cetonas en la orina, eso da un señal que tal vez no hay suficiente insulina en el cuerpo. Puede ser que la paciente tiene una clase de diabetes más como diabetes I que diabetes II. Pacientes con cetonas en la orina también pueden tener una emergencia se llama ketoacidosis diabética que requiere hospitalización.

- En segundo lugar, llame al médico de turno, si (1) cetonas son positivas en la orina o (2) cualquier señal de peligro de la hiperglucemia peligrosa abajo:

Tabla: Señales de peligro de la hipoglucemia

**Señal**	**Notas**
Signos de deshidratación	Boca seca, disminución de orina
Presión arterial baja	<90 sistólica
Estado mental alterado	Agitación, desorientación, somnolencia, coma

12. Cuidado de pies

12.1. El cuidado regular del pie

La enfermera clínica de la diabetes debe realizar un examen de los pies de diabéticos en cada visita. Si hay infección de las uñas de dedos (tinea pedis), debe tratar con crema de Clotriplex.

Además, la enfermera debe dar educación del bien cuidado de los pies, incluyendo:

- Lavar los pies diario
- Cortar las uñas de los pies cada semana con mucho cuidado
- Usar zapatos o caites apropiados (y no demasiado pequeños)
- Llamarnos si desarrolla un úlcera.

12.2. Cuidado de la úlcera aguda

Si un paciente se presenta o llamadas con una úlcera, es importante saber primero la gravedad de la úlcera.

- Si la úlcera es pequeña y solamente involucra el piel, tomar una foto y mandarla a la coordinador de diabetes dentro de 24 horas. También, cubrir la herida con una gasa para protegerla. Dar educación de revisión diario del pie, la importancia de aliviar presión en la herida, y que hacer si la herida empeora.

- Si hay la presencia de una úlcera más grave, tomar una foto y llamar al médico del turno inmediatamente.

12.3. Kit de cuidado de los pies

La enfermera de la clínica de la diabetes y la enfermera educadora siempre deben llevar consigo una pequeña bolsa de suministros para el cuidado del pie en cualquier

visita clínica o visita de casa. La bolsa debe incluir vaselina, guantes, gasa 4x4, cinta médica, alcohol, y una pequeña botella de solución salina.

13. Vigilar nefropatía

13.1. Creatinina en la sangre

Hay que revisar la creatinina en la primera visita y en los intervalos de la siguiente manera:

Tabla: La frecuencia de pruebas de creatinina

**Tasa de filtración glomerular (TFG/GFR)**	**Frecuencia de medidas de creatinina**
>50	Una vez por año
30-50	Cada 6 meses
<30	Depende del caso; hay que comunicar con el coordinador de diabetes

Siempre usar la calculadora de CKD-EPI para calcular TFG/GFR con cada paciente que tiene creatinina >1.0. Ponga el número de TFG/GFR en OpenMRS.

13.2. Proteína en la orina

Todos los pacientes deben hacer una prueba de proteinuria utilizando una tira al menos una vez al año. Los resultados deben ser manejados de la siguiente manera:

- Un paciente que tiene 1+ o más proteinuria debe tener otra revisión de proteinuria en 3-6 meses. Después de dos pruebas de 1+ más proteinuria en 6 meses, si el paciente no está tomando enalapril y tiene una TFG/GFR >30, empiece enalapril 5 mg al día. Recuerde, una creatinina debe haber sido revisado en el año anterior antes de iniciar tratamiento con enalapril.

- Revisar inmediatamente y consultar con el coordinador de la diabetes si un paciente tiene nuevos hallazgos de 2+ o más proteinuria.

14. Manejo de presión arterial

Control de la presión de la sangre es una parte vital del manejo adecuado de la diabetes porque la hipertensión puede contribuir a agravar complicaciones de la diabetes en los ojos, los riñones, y los vasos sanguíneos.

14.1. El diagnóstico de la hipertensión arterial

- 1 medida de ≥160 sistólica o ≥110 diastólica

- 2 medidas de 140-160/90-110 con por lo menos un mes de diferencia

14.2. La meta de presión arterial en los diabéticos

La meta general de presión arterial en los pacientes con diabetes es <140/90.

14.3. Manejo de hipertensión aguda

- Si un paciente no tiene síntomas de peligro, pero tiene la presión arterial ≥180 sistólica o ≥120 diastólica, consultar con el coordinador de la diabetes.

- Si el paciente tiene alguno de los signos de peligro por debajo y tiene la presión arterial ≥180 sistólica o ≥120 diastólica, llamar al medico del turno.

Tabla: Señales de peligro en emergencias hipertensivas

Nuevo dolor de cabeza muy fuerte
Visión borrosa
Hinchazón de las piernas
Dolor de pecho
Cambio agudo en el estado mental

14.4. Los medicamentos anti-hipertensivos

El formulario tiene los siguientes medicamentos orales para el tratamiento de la hipertensión:

Tabla: El formulario antihipertensivo

**Medicamento**	**Posología**	**Efecto secundario**
Enalapril	2.5-20 mg (pastilla de 20 mg)	**Tos, empeora la función renal, angioedema (un tipo de hinchazón, más común en los labios, la lengua, y la cara)**
Amlodipina	5-10 mg (pastilla de 5 mg)	**Hinchazón de pies**
HCTZ	12.5-25 mg (pastilla de 25 mg)	Hiperglucemia
Atenolol	25-100 mg (pastilla de 100 mg)	La bradicardia, fatiga, disfunción sexual

14.5. Manejo de la hipertensión crónica

La siguiente tabla muestra los pasos para tratar la hipertensión crónica. En general, el régimen de un paciente no debe ser ajustado más de 1 paso de un mes a otro sin avisar al coordinadora de la diabetes.

Tabla: El algoritmo para el manejo de la hipertensión crónica

**Paso**	**Dosificación**	**Nota**
Paso 1 (después del diagnóstico de la hipertensión)	Empezar 5 mg enalapril una vez al día	Hay que tener prueba de creatinina con TFG/GFR >30 dentro de 1 año antes de empezar enalapril
Paso 2 (si presión ≥140/90 en la visita del próximo mes)	Aumentar el enalapril a 10 mg una vez al día	Hacer prueba de creatinina y potasio entre 3-14 días después del cambio de enalapril si TFG/GFR del paciente es <50. Con la dosis de enalapril más alta, contactar el coordinador de diabetes si (1) la creatinina aumenta ≥30% o (2) el potasio es fuera del rango normal de 3.5-5.0 mEq/L.
Paso 3 (si presión ≥140/90 en la visita del próximo mes)	Aumentar el enalapril a 20 mg una vez al día	Hacer prueba de creatinina y potasio entre 3-14 días después del cambio de enalapril si TFG/GFR del paciente es <50. Con la dosis de enalapril más alta, contactar el coordinador de diabetes si (1) la creatinina aumenta ≥30% o (2) el potasio es fuera del rango normal de 3.5-5.0 mEq/L.
Paso 4 (si presión ≥140/90 en la visita del próximo mes)	Agregar amlodipina 5 mg una vez al día	
Paso 5 si presión ≥140/90 en la visita del próximo mes	Aumentar amlodipina a 10 mg una vez al día	
Paso 6 si presión ≥140/90 en la visita del próximo mes	Consultar con el coordinador de la diabetes	

14.6. Efectos secundarios de enalapril

Enalapril es una excelente medicina para los diabéticos porque ayuda a proteger los riñones. Sin embargo, enalapril tiene algunos efectos secundarios que debemos tener en cuenta.

- Disminuye en función renal. Enalapril puede causar un aumento de la creatinina que es más preocupante en pacientes con enfermedad renal crónica. Por esta razón, cuando aumentamos la dosis hasta 10 mg o 20 mg diario en pacientes con TFG/GFR <50, tenemos que revisar creatinina y potasio entre 3-14 días.

- Tos. Enalapril puede causar una tos crónica en algunos pacientes. Los beneficios del enalapril son grandes, así que antes de quitar los enalapril por este efecto de tos, debemos ser muy confiados en varias visitas que enalapril es la verdadera causa de la tos.

- Angioedema. Enalapril puede causar angioedema, un tipo de hinchazón más común en los labios, la lengua, y la cara. Este es un efecto secundario grave, y la enfermera de la clínica de la diabetes debe llamar al médico de turno inmediatamente si se sospecha un caso de angioedema.

14.7. Educación para diabéticos con hipertensión

Además, debe mosdar educación a los pacientes con hipertensión crónica sobre la importancia de reducir el consume de sal, la realización de ejercicio regular, y la reducción de la ingesta de alcohol.

15. Educación de diabetes

15.1. En la clínica

Muchos estudios han demostrado que la educación es una parte importante de la buena atención de la diabetes. Cada encuentro clínico es una oportunidad que el personal entregue educación.

15.2. En la casa

Además, todos los pacientes que no han cumplido con el objetivo de A1C deben ser referidos para el programa de las visitas a domicilio realizadas por la enfermera educadora.

16. Tratamiento de ojos

Los diabéticos tienen un alto riesgo de las cataratas y la retinopatía. La enfermera de la diabetes debe mantener una lista de los pacientes con cataratas o las quejas graves de vista para la revisión del coordinador de la diabetes.

17. Microalbumina en la orina

Medir y manejar la microalbumina en la orina es importante para detectar los primeros signos de daño renal.

17.1. Las detalles de la logística:

- Usar Ultralab con esta prueba de orina

- Pedir mínimo 40 mL de orina del paciente

- La orina se puede guardar por 7 días si es refrigerado, así que podemos juntar todos los pruebas y solo hacer una entrega a Ultralab.

17.2. Las detallas del manejo

Clínicamente, lo importante es manejar la tasa de albumina por creatinina en una muestra de orina al azar:

**Tasa de albumina por creatinina**	**Resultado**	**Qué hacer**
<30 mg/g	negativo	nada
>30 mg/g	positivo	otra prueba en 3-6 meses

Todos los pacientes necesitan 1 prueba de microalbúmina. **No es necesario la segunda prueba si la paciente ya está tomando enalapril.**

Reglas para el manejo de resultados:

- Primer resultado de microalbumina
  - ACR <30: No hacer nada
  - ACR ≥30: Hay que repetirlo en 3 meses si paciente no está toma enalapril

- Segundo resultado de microalbumina
  - ACR <30: Hacer tercera prueba de microalbumina
  - ACR ≥30: Empezar enalapril 5mg diario (si TFG/GFR >30)

- Tercer resultado de microalbumina:
  - Si 2/3 resultados son ACR <30: No hacer nada
  - Si 2/3 resultados son ACR ≥30: Empezar enalapril 5mg diario (si TFG/G.FR >30)
