# Supplementary material for: Implementation and Outcomes of a Comprehensive Type 2 Diabetes Program in Rural Guatemala
Source: PLoS One. 2016 Sep 1;11(9):e0161152. doi: 10.1371/journal.pone.0161152 (PMC5008811; doi:10.1371/journal.pone.0161152)
Supplement: S2 File — Home education manual adapted from U.S. National Heart, Lung and Blood Institute’s Salud Para Su Corazón (Health for Your Heart) community health worker model for Latinos. (PDF) [file pone.0161152.s002.pdf]

SALUD DEL CORAZÓN

Raxnaqil ri K'uxaj

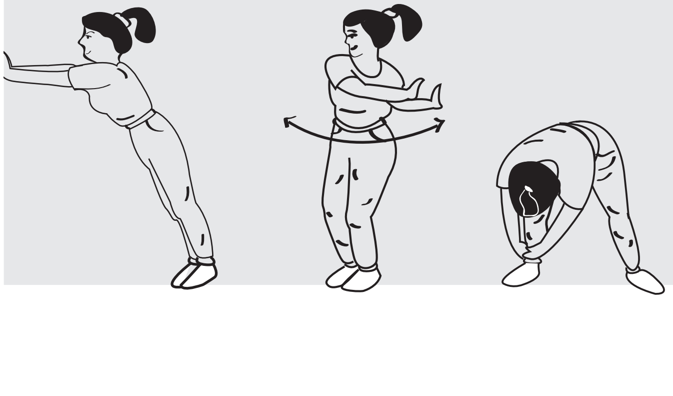

SESIÓN DE CAPACITACIÓN: CÓMO PREPARARSE PARA HACER UNA PRESENTACIÓN

NOTA : *Si su objectivo es capacitar a promotores de salud (taq tzijöy aq'omab'äl).*

Los buenos promotores de salud (taq jeb'ël tzijöy aq'omab'äl) deben saber desenvolverse y hacer presentaciones en público. En esta sección encontrará información sobre cómo un promotor de salud (tzijöy aq'omab'äl) puede prepararse para hacer un proyecto de capacitación para otros promotores de salud (taq tzijöy aq'omab'äl) usando el manual.

DIGA:

· Cuando ustedes finalicen la capacitación, podrán dirigir su propio proyecto para grupos de su comunidad. Como promotores de salud (taq tzijöy aq'omab'äl), no estarán dando consejo o asesoramiento médico. Ustedes ofrecerán información y apoyo a otros para animarlos a que tengan vidas más sanas y a que aprovechen los servicios de salud disponibles en la comunidad.

NOTA : *Repase con cada promotor de salud (tzijöy aq'omab'äl) las hojas "Sugerencias para impartir el manual" y "9 reglas para la enseñanza de grupos" (páginas 2 – 3)*

DIGA:

· Ahora vamos a repasar algunos pasos que les ayudarán a preparar una presentación más interesante y motivadora del proyecto su corazón, su vida.

· El manual acerca de la salud del corazón tiene 7 sesiones. Las sesiones cubren los siguientes temas:

DIGA:

· Empecemos leyendo las "Sugerencias para impartir el manual" (página 'n') y las "Siete reglas para la enseñanza de grupos" (página 'n'). Les recomiendo que usen estas hojas cuando preparen la presentación y enseñen el manual.

DIGA:

· Aquí tienen algunas ideas para practicar más y ganar más confianza:

  · Observen a un promotor (tzijöy aq'omab'äl) con experiencia enseñar una sesión completa o todo el manual.
  · Presenten una sesión completa a otros promotores de salud (taq tzijöy aq'omab'äl).
  · Enseñen en equipo una sesión o todo el manual a un grupo de la comunidad, con la ayuda de un promotor (tzijöy aq'omab'äl) con más experiencia. Pídanle al coordinador de su proyecto que les brinde apoyo.

9 REGLAS PARA LA ENSEÑANZA DE GRUPOS

1. Mantenga contacto visual con todos los participantes.
2. Hable para que todos los participantes puedan oír: Hable con voz clara, fuerte y cordial.
3. Muestre su entusiasmo: Sea expresivo, muévase, use sus manos y sonría.
4. Recuerde cuánto tiempo tiene disponible: Planee su presentación de modo que no tenga que apresurarse. No permita que la clase se extienda mucho.
5. Muestre interés por los participantes:
   · Salúdelos cuando llegan a la sesión.
   · Dígales que aprecia el tiempo que dedican al proyecto y su asistencia.
   · Escuche lo que digan.
   · Hable de manera simple y sin rodeos.
   · Ayúdeles a fijar metas realistas para sus tareas.

- Manténgase tranquilo y tome las cosas con humor. Tenga una actitud positiva.
- Termine con un repaso de los puntos más importantes. Agradézcales su asistencia.

6. Trate de que todos participen: Las personas tienden a aprender más cuando participan activamente. Evite ser sólo usted quien habla al grupo:
7. Haga preguntas al grupo. Elogie las respuestas correctas. Corrija amablemente la información incorrecta.
8. Responda a las preguntas de los participantes. Sea honesto si no sabe la respuesta. Averigue las respuestas a las preguntas que no puede contestar.
9. Preste atención al contenido de cada sesión.

7 RECETAS SALUDABLES

Prepare una de las recetas saludables antes de cada clase. Sepa el número de participantes en su clase y lleve una cantidad suficiente de alimentos.

ENSALADA DE FRUTAS

Ingredientes: melon, fresa, sandillas y uvas

Procedimiento: Lave muy bien las frutas, las corte en trocitos y las eche en un trasto para servirlas.

SÁNDWICH DE ATÚN CON UN TÉ O FRESCO SIN AZÚCAR

Procedimiento: Lave muy bien la lechuga y escurra el atún. Después ponga las rodajas de pan integral, la lechuga y el atún. Prepare el fresco o té sin azúcar.

LICUADO DE FRUTA

Procedimiento: Corte una fruta (como papaya y piña) que le guste en trozos. Ponga la fruta en la licuadora junto con agua y vainilla. Licúe hasta que esté cremosa.

PAN CON FRIJOLES

Procedimiento: Cocine los frijoles en agua hasta que estén blandos. Condiméntelos con cebolla, ajo, comino, o oregano. Para espesar los frijoles, cuelelos y cocínelos a fuego lento en una sartén con un poco de agua (sin aceite). Después haga los frijoles colados y ponga una cuchara en un pan integral.

ATOLE CON FRIJOLES

Procedimiento: Prepare atole con leche descremada con avena integral y ponga los frijoles (el tipo lo que prefiere).

BOLSITAS DE FRUTA

Ingredientes: sandilla, mango y piña

Procedimiento: Lave muy bien las frutas, las corte en trocitos y las eche en una bolsa para servirlas. Puede poner limón encima de las frutas.

BANANOS Y CAFÉ

Procedimiento: Simplemente dé a los participantes bananos y café sin azúcar.

NOTA : *Sea creativo! Prepare diferentes recetas para proporcionar en capacitación. Lea Sesión 3 para seguir una alimentación saludable.*

LA CAPACITACIÓN SOBRE LA SALUD DEL CORAZÓN (RI TIJONÏK PA RUWI’ RI RAXNAQIL K’UXAJ)

(páginas 1 – 14) 1. Está usted en riesgo de desarrollar enfermedades del corazón?

(páginas 15 – 29) 2. Actué rápido frente a los síntomas de un ataque al corazón.

(páginas 30 – 48) 3. Piense en su corazón: Dígale Sí a la alimentación saludable y la actividad física.

(páginas 49 – 66) 4. Ayude a su corazón: Controle su presión arterial alta.

(páginas 67 – 85) 5. Sea bueno con su corazón: Mantenga su colesterol bajo control.

(páginas 86 – 107) 6. Proteja su corazón: Cuidese de la diabetes.

(páginas 108 - 117) 7. Repaso y graduación.

SESIÓN 1 ¿Está usted en riesgo (retzelal) de desarrollar enfermedades del corazón (taq ruyab'ilal k'uxaj)?

OBJETIVOS

AL FINALIZAR ESTA SESIÓN, LOS PARTICIPANTES CONOCERÁN:

- El proyecto Corazón Sano y Feliz
- Cómo funciona el corazón y su importancia
- Que enfermedades del corazón (taq ruyab'ilal k'uxaj) se pueden prevenir
- Podrán nombrar seis factores de riesgo (rub'anikil rutzilal) para las enfermedades del corazón (taq ruyab'ilal k'uxaj) que se pueden prevenir
- A los demás participantes del grupo

MATERIALES

PARA DIRIGIR ESTA SESIÓN USTED NECESITARÁ:

- Un recipiente transparente con 4 tazas de agua

HOJAS PARA REPARTIR:

DURANTE LA SESIÓN, ENTREGUE A LOS PARTICIPANTES ESTA HOJA:

- "Factores de riesgo para Las Enfermedades del Corazón que Usted Puede Controlar" (página 11)

CARTELES PARA MOSTRAR:

- Cartel derivado de dibujo educativo 1.1 (página 12)
- Cartel derivado de dibujo educativo 1.2 (página 13)
- Cartel derivado de la hoja "Una día con la familia Cojtí" (página 14)

ESQUEMA DE LA SESIÓN

INTRODUCCIÓN A LA SESIÓN

1. Bienvenida
2. Presentación del proyecto
3. Reglas

LA SESIÓN EN ACCIÓN

1. Actividad para conocernos mejor
2. Los secretos del corazón
   a. Cómo funciona el corazón
   b. La estructura del corazón
3. Información sobre las enfermedades del corazón (taq ruyab'ilal k'uxaj)
4. Actividad sobre los factores de riesgo (rub'anikil rutzilal)
5. Una día con la familia Cojtí

REPASO DE LOS PUNTOS MÁS IMPORTANTES DE HOY

CIERRE DE LA SESIÓN

NOTA : *Antes de la sesión lea las páginas 8-10 para preparar a responder las preguntas de los participantes.*

INTRODUCCIÓN A LA SESIÓN

1 | BIENVENIDA

- Preséntese a medida que los participantes entren a la clase.
- Pida a los participantes que se coloquen las etiquetas con su nombre en la blusa o camisa.
- Dé la bienvenida a los participantes a la sesión. Dígales que está muy contento de verlos.

2 | PRESENTACIÓN DEL PROYECTO

DIGA:

· Las enfermedades del corazón (taq ruyab'ilal k'uxaj) son una de las causas principales de muerte en Guatemala crearon la iniciativa del proyecto, para ayudar a la población guatemaltecos (as) en las comunidades rurales a combatir las enfermedades del corazón (taq ruyab'ilal k'uxaj).

· Juntos aprenderemos sobre los factores de riesgo (rub'anikil rutzilal) para las enfermedades del corazón (taq ruyab'ilal k'uxaj). También aprenderemos qué cosas pueden hacer ustedes con su familia para tener un corazón sano (raxnaqil k'uxaj).

· El manual acerca de la salud del corazón tiene 7 sesiones. Las sesiones cubren los siguientes temas:

HAGA : *Muestre el cartel derivado del dibujo educativo 1.1 (página 12).*

1. ¿Está usted en riesgo de desarrollar las enfermedades del corazón (taq ruyab'ilal k'uxaj)?
2. Actúe rápido frente a los síntomas (taq k'u't) de un ataque al corazón (b'ayb'ot k'uxaj).
3. Piense en su corazón: Dígale Sí a la alimentación saludable y la actividad física.
4. Ayude a su corazón: Controle su presión arterial alta (jotöl ruchuq'a' kïk').
5. Sea bueno con su corazón: Mantenga su colesterol bajo control (chöj ruchuq'a' ri saqab'uyül q'anal).
6. Proteja su corazón: Cuidese de la diabetes (kab'yab'il).
7. Repaso y graduación.

DIGA:

· Por favor, vengan a todas las sesiones. Yo voy a dirigir las sesiones, pero necesito la participación de cada uno de ustedes. Esto les ayudará a aprender la información y a disfrutar más las sesiones. Siéntanse en libertad de hacer preguntas. Las preguntas nos ayudarán a todos a aprender más.

NOTA : *Esta sección ayudará a los participantes a sentirse parte de un grupo. También le facilitará a usted la enseñanza.*

3 | REGLAS

DIGA:

· Todos se sentirán más cómodos si saben qué esperar de esta capacitación. Antes de comenzar, hablemos acerca de la mejor manera de dirigir las sesiones. Pongámonos de acuerdo como grupo acerca de algunas reglas básicas.Cada uno de ustedes deberá dedicar un poco de tiempo y esfuerzo a esta capacitación. Es importante que asistan a cada sesión y que lleguen a tiempo. Cada sesión dura alrededor 2 horas.

· Quiero darles unas sugerencias para que logren sacar el mayor provecho de cada sesión:

1. Siéntanse con libertad de hacer preguntas.
2. Compartan sus experiencias personales. Las sesiones tendrán así mayor significado para todos nosotros.

3. Traten de no salirse del tema. Tenemos mucha información que cubrir en un tiempo limitado.
4. (Opcional) Llamenme si tienen alguna pregunta o dudas sobre estas sesiones (de su numero de telefono). Estoy aquí para ayudarles de cualquiera manera que pueda.

DIGA:

· Como ustedes van a compartir sus experiencias y opiniones, necesitamos ponernos de acuerdo sobre algunas cosas.

NOTA : *Dé a los participantes unos minutos para responder.*

PREGUNTE:

· ¿Están de acuerdo en no compartir con nadie los comentarios de los otros participantes?

· ¿Están de acuerdo en no juzgar a los demás? ¿Se comprometen a tratar de asistir a todas las sesiones?

· ¿Tienen alguna otra regla que les gustaría añadir?

LA SESIÓN EN ACCIÓN

1 | ACTIVIDAD PARA CONOCERNOS MEJOR

NOTA : *Para dar comienzo a esta actividad, hable usted primero. Diga al grupo por qué le interesa la salud del corazón (raxnaqil k'uxaj). Con honestidad, hable de su experiencia personal. Dé a cada participante la oportunidad de participar.*

DIGA:

· Para empezar la sesión de hoy, vamos a presentarnos y tratar de conocernos un poco mejor.

PIDA A LOS PARTICIPANTES:

- Que den su nombre.
- Que digan por qué vinieron a las sesiones y qué esperan aprender.

DIGA:

· Ustedes pueden ayudar a su familia y amistades dando un buen ejemplo, compartiendo con ellos lo que aprenden y animándoles a vivir una vida más sana. Gracias por hacer este esfuerzo.

2 | LOS SECRETOS DEL CORAZÓN

DIGA:

· Ahora que ya nos conocemos un poco más, vamos a conocer algunos detalles sobre el corazón.

a | EL CORAZÓN Y CÓMO FUNCIONA

HAGA : *Muestre un recipiente transparente que contenga 1 litro (unas 4 tazas) de agua. Recuerde al grupo que el corazón bombea cinco veces esta cantidad de sangre por minuto.*

DIGA:

· El corazón es una parte maravillosa del cuerpo. Bombea aproximadamente 5 litros de sangre por minuto y late unas 100,000 (cien mil) veces por día.

DIGA:

· El corazón envía sangre a los pulmones para que se carguen de oxígeno (uxlab'il). Luego lleva la sangre oxigenada a todas las células (taq rujotayil k'aslemal) del cuerpo.

NOTA : *Muestre al grupo cómo hacer esto.*

DIGA:

- Ustedes pueden sentir el latido del corazón si se toman el pulso (tzop). Para encontrar el pulso (tzop), coloquen suavemente el dedo índice y el del medio de una mano en la parte de adentro de la muñeca de la otra mano. Deslicen los dos dedos por la muñeca en dirección al dedo pulgar hasta que sientan una leve tzopinem.

NOTA : *Muestre el dibujo educativo 1.2 con el cartel (página 13).*

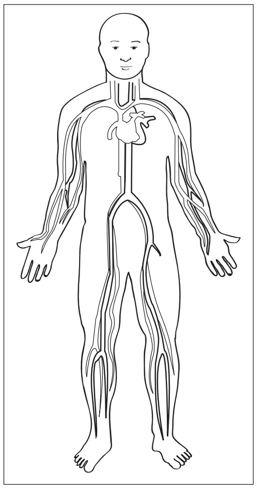

b | LA ESTRUCTURA DEL CORAZÓN (K'UXAJ)

DIGA:

- El corazón es un órgano hueco, muscular, en forma de cono, más o menos del tamaño de un puño.
- El corazón está ubicado en al lado izquierda del pecho. ¿Colocaron el corazón que les di en su debido lugar?
- El corazón es una de las partes principales del sistema circulatorio (kik'b'äl). Las otras partes son los vasos sanguíneos (taq rub'ey kïk') y la sangre.
- el sistema circulatorio (kik'b'äl) es muy importante para sostener la vida. Está compuesto por todos los vasos sanguíneos (taq rub'ey kïk') que llevan la sangre a todas las partes del cuerpo.
- Los vasos sanguíneos (taq rub'ey kïk') son largos tubos huecos de tejido, muy parecidos a las pajillas para beber. Hay varios tipos de los vasos sanguíneos (taq rub'ey kïk'). Los principales se llaman arterias venas (taq kaqib'öch') y capilares (taq k'amib'öch').
- El sistema circulatorio (kik'b'äl) lleva oxígeno (uxlab'il) y nutrientes (taq ilinemil) a las células (taq rujotayil k'aslemal) del cuerpo y remueve los productos de deshecho (mek'atzin k'ayinelil richin ri kik'b'äl).
- La sangre, con poco oxígeno (uxlab'il), entra al corazón a través de las venas más grandes del cuerpo.
- De allí, la sangre entra a los pulmones a través de las arterias pulmonares (rib'öch' pospo'y)
- En los pulmones se quitan los desechos de la sangre dióxido de carbono (xar uxlab'il). Entonces, la sangre recoge más oxígeno (uxlab'il).
- La sangre llena de oxígeno (uxlab'il) regresa al corazón. De allí la sangre es bombeada a través de la aorta (kite' kaqib'öch') al resto del cuerpo.

DIGA:

- Las enfermedades del corazón (taq ruyab'ilal k'uxaj) afectan al músculo del corazón (ruti'ojilal k'uxaj), a los vasos sanguíneos (taq rub'ey kïk') del corazón. Como el corazón es un órgano tan importante, es necesario prevenir estas enfermedades. Cuando el corazón se para, la vida se para. De modo que si se cuidan el corazón, estarán cuidando su vida.

NOTA : *Antes de la sesión, lea el cuadro de las páginas 8-10. Este cuadro muestra una lista de los factores de riesgo (taq rub'anikil rutzilal) para las enfermedades del corazón (taq ruyab'ilal k'uxaj) sobre los que usted puede hacer algo. También aparecen las medidas que usted puede tomar para prevenir o disminuir esos factores de riesgo (rub'anikil rutzilal).*

3 | INFORMACIÓN SOBRE LAS ENFERMEDADES DEL CORAZÓN (TAQ RUYAB'IL K'UXAJ)

DIGA:

- Ahora que sabemos lo importante que es el corazón, hablemos sobre las enfermedades del corazón (taq ruyab'ilal k'uxaj) y lo que podemos hacer para proteger nuestro corazón.

NOTA : *Dé a los participantes unos 2 a 5 minutos para contestar.*

PREGUNTE:

- ¿Conocen a alguien que tiene una enfermedad del corazón (ruyab'ilal k'uxaj)?

DIGA:

- Las enfermedades del corazón (taq ruyab'ilal k'uxaj) son un serio problema de salud para la población guatemalteca, una de las principales causas de muerte.
- Muchas personas creen que un ataque al corazón (rukamik k'uxaj) o ataque al cerebro (rukamik tzatzq'or) ocurre repentinamente cuando uno se lleva un susto, recibe malas noticias o tiene una fuerte emoción, por ejemplo, de enojo.
- un ataque al corazón (rukamik k'uxaj) o ataque al cerebro (rukamik tzatzq'or) puede parecer repentino, pero lo cierto es que las enfermedades del corazón (taq ruyab'ilal k'uxaj) se van desarrollando a través de los años. Generalmente comienzan cuando uno es muy joven.
- Es importante tomar medidas a cualquier edad para prevenir las enfermedades del corazón (taq ruyab'ilal k'uxaj). Deben tomar medidas para cuidar su cuerpo y disminuir la probabilidad de desarrollar las enfermedades del corazón (taq ruyab'ilal k'uxaj).
- Estas sesiones les mostrarán de qué manera ustedes y sus familias pueden tener un corazón más sano.

HAGA : *Entregue a los participantes la hoja "Factores de riesgo para las Enfermedades del Corazón que Usted Puede Controlar" (página 11).*

DIGA:

- En estas sesiones ustedes oirán hablar de los factores de riesgo (taq rub'anikil rutzilal). Así se llaman las características o hábitos que contribuyen a que una persona tenga más probabilidad de desarrollar enfermedades del corazón (taq ruyab'ilal k'uxaj). Algunas de estas características no se pueden cambiar, como la edad, la historia médica familiar y el hecho de ser hombre o mujer. Pero lo bueno es que uno sí puede hacer algo acerca de algunos de los factores de riesgo (taq rub'anikil rutzilal) como:
  - la presión aterial alta (jotöl ruchuq'a' kïk')
  - un nivel alto de colesterol (jotöl ruchuq'a' ri saqab'uyül q'anal) en la sangre
  - el hábito de fumar
  - la diabetes (kab'yab'il)
  - el sobrepeso
  - la falta de actividad física

4 | ACTIVIDAD SOBRE LOS FACTORES DE RIESGO (TAQ RUB'ANIKIL RUTZILAL)

PREGUNTE:

- ¿Está usted en riesgo (retzelal) de desarrollar las enfermedades del corazón (taq ruyab'ilal k'uxaj)? Lea en voz alta todos los factores de riesgo (taq rub'anikil rutzilal):
  - presión aterial alta (jotöl ruchuq'a' kïk')
  - nivel alto de colesterol (jotöl ruchuq'a' ri saqab'uyül q'anal) en la sangre
  - diabetes (kab'yab'il)
  - inactividad física
  - hábito de fumar
  - Sobrepeso

DIGA:

- Hay factores de riesgo (taq rub'anikil rutzilal) para las enfermedades del corazón (taq ruyab'ilal k'uxaj) que usted no puede cambiar o controlar:
  - Edad hay más riesgo (retzelal) cuando son mayores
  - Historia familiar:

- Familia con enfermedades del corazón (taq ruyab'ilal k'uxaj)
- Joven en la familia con enfermedades del corazón (taq ruyab'ilal k'uxaj) antes de los 65 años

DIGA:

- Mientras más factores de riesgo (taq rub'anikil rutzilal) tenga, mayor será su riesgo (retzelal) de desarrollar enfermedades del corazón (taq ruyab'ilal k'uxaj) o un ataque al cerebro (b'ayb'ot tzatzq'or). Hablen con su médico acerca de su riesgo (retzelal).

DIGA:

- En estas sesiones aprenderán cómo estos factores de riesgo (taq rub'anikil rutzilal) afectan la salud del corazón (raxnaqil k'uxaj). También verán distintas maneras cómo ustedes y sus familias pueden prevenirlos o controlarlos. Aprenderemos como:
  - Disminuir el consumo de sal.
  - Comer alimentos con bajo contenido de grasa saturada (ritzelal q'anal).
  - Consumir más frutas y verduras y más leche o productos de leche bajos en grasa (q'anal).
  - Medirse la presión aterial (ruchuq'a' kïk') y los niveles de colesterol (saqab'uyül q'anal) y de azúcar en la sangre.
  - Tomar los medicamentos recetados por su médico siguiendo sus instrucciones.
  - Dejar de fumar.
  - Mantenerse físicamente activos.
  - Perder peso si tiene sobrepeso.

NOTA : *Dé a los participantes uno minuto para responder.*

HAGA : *Muestre el cartel derivado de la hoja "Un día con la familia Cojtí" (página 14). Después, lea las preguntas en ésta página..*

5 | UNA DÍA CON LA FAMILIA COJTÍ

PREGUNTE:

- ¿Cómo se sintieron cuando supieron que ustedes tienen algunos factores de riesgo (taq rub'anikil rutzilal) para las enfermedades del corazón (taq ruyab'ilal k'uxaj)?

DIGA:

- Las personas actúan de diferentes maneras cuando se enteran que deben hacer cambios en sus hábitos para disminuir su riesgo (retzelal) de desarrollar enfermedades del corazón (taq ruyab'ilal k'uxaj). Escuchamos algunos ejemplos de la familia Cojtí.

PREGUNTE:

- ¿Con cuál miembro de la familia se identifican más?
- ¿Qué razones tienen ustedes para mejorar su salud?
- ¿Qué barreras les impiden hacer cambios en su vida diaria?
- ¿Qué pueden hacer para superar estas barreras?

REPASO DE LOS PUNTOS MÁS IMPORTANTES DE HOY

DIGA:

- Repasemos lo que aprendimos hoy.

PREGUNTE:

- ¿Cuáles son los factores de riesgo (taq rub'anikil rutzilal) para las enfermedades del corazón (taq ruyab'ilal k'uxaj) qué nosotros podemos prevenir o controlar?

    AÑADA LOS SIGUIENTES FACTORES SI NADIE LOS MENCIONA:

    - la presión aterial alta (jotöl ruchuq'a' kïk')
    - un nivel alto de colesterol (jotöl ruchuq'a' ri saqab'uyül q'anal) en la sangre
    - el hábito de fumar
    - los factores de riesgo (taq rub'anikil rutzilal)
    - el sobrepeso
    - la inactividad física

DIGA:

- Puede ser difícil cambiar viejos hábitos dañinos por nuevos hábitos saludables. Estas sesiones educativas les darán la información necesaria para poder hacer esos cambios poco a poco. Cuando otros vean que usted hace estos cambios saludables, tal vez también quieran hacer lo mismo.

CIERRE DE LA SESIÓN

DIGA:

- Gracias por asistir el día de hoy. ¿Les gustó la sesión de hoy?

DIGA:

- En la siguiente sesión hablaremos sobre cómo actuar rápido frente a los síntomas (taq k'u't) de un ataque al corazón (rukamik k'uxaj).
- También, pueden invitar a su familia, o una persona de su familia, a las clases siguientes.

PREGUNTE:

- ¿Tienen alguna pregunta sobre estas sesiones?

(OPCIONAL) PREGUNTE:

- ¿Qué piensan de la sesión de hoy?
- ¿Qué creen ustedes que funcionó y qué no funcionó?
- ¿Qué cambiaron ustedes en sus vidas como resultado de lo que aprendieron en esta sesión?

NOTA : *Dé un momento lo que dos personas pueden discutir y después dan su opinión en esta sesión.*

PRESÍON ARTERIAL ALTA (JOTÖL RUCHUQ'A' KÏK')

La presión arterial alta (jotöl ruchuq'a' kïk') (o hipertensión) se le llama la asesina silenciosa (meq'ajan kamisanel) porque no tiene los síntomas (taq k'u't). Sin embargo puede causar enfermedades muy graves.

LO QUE USTED NECESITA SABER

Cuando su presión arterial (ruchuq'a' kïk') está alta, su corazón trabaja más fuerte de lo que debería para bombear la sangre a todas las partes del cuerpo. Si no se trata, presión arterial alta (jotöl ruchuq'a' kïk') puede causar un ataque al corazón ataque al corazón (rukamik k'uxaj) o un ataque al cerebro (rukamik tzatzq'or), problemas de los ojos y los riñones (kinäq') y la muerte.

Chequéese su presión arterial (ruchuq'a' kïk') (mm Hg)*:

Nivel	Presión sistólica	Presión diastólica
**normal (chöj ruchuq'a' kïk')**	Menos de 120	Menos de 80
**prehipertensión (uchuq'amil)**	120-139	80-89
**presíon arterial alta (jotöl ruchuq'a' kïk')**	140 o más	90 o más

*Milímetros de mercurio.

LO QUE USTED PUEDE HACER PARA PREVENIR LAS ENFERMEDADES DEL CORAZÓN (TAQ RUYAB'ILAL K'UXAJ)

- Chequéese la presión arterial (ruchuq'a' kïk') una vez al año. Chequéesela más a menudo si tiene la presión aterial alta (jotöl ruchuq'a' kïk').
- Logre mantener un peso saludable.
- Manténgase físicamente activo, incluso a través del trabajo.
- Elija y prepare alimentos con menos sal.
- Coma más frutas, verduras y productos de leche bajos en grasa (q'anal).
- Si toma bebidas alcohólicas, hágalo en moderación.
- Si su médico le receta medicamentos para la presión aterial (ruchuq'a' kïk'), tómelos siguiendo sus instrucciones.
- Si fuma, deje de hacerlo.

UN NIVEL ALTO DE COLESTEROL (JOTÖL RUCHUQ'A' RI SAQAB'UYÜL Q'ANAL) EN LA SANGRE

El colesterol (saqab'uyül q'anal) en las arterias (kaqib'och'ilal) es como el sedimento en una tubería. El colesterol LDL (lipoproteína de baja densidad) (qajnäq ruchuq'a' nimak'wanel q'anal) también se conoce como el colesterol "malo". Demasiado colesterol LDL o colesterol malo (qajnäq ruchuq'a' nimak'wanel q'anal) puede obstruir las arterias (kaqib'och'ilal), lo que aumenta el riesgo (retzelal) de enfermarse del corazón y de tener un ataque al cerebro (rukamik tzatzq'or). El colesterol HDL (lipoproteína de alta densidad) (jotöl ruchuq'a' nimak'wanel q'anal) también se conoce como el colesterol "bueno". El HDL (jotöl ruchuq'a' nimak'wanel q'anal) ayuda a quitar el colesterol (saqab'uyül q'anal) de las arterias (kaqib'och'ilal).

LO QUE USTED NECESITA SABER

Chequéese su nivel total de colesterol (saqab'uyül q'anal) en la sangre. (mg/dl*):

**Deseable**	**Menos de 200**
**Moderadamente alto**	200-239
**Alto**	240 o más

Chequéese su nivel de colesterol LDL o colesterol malo (qajnäq ruchuq'a' nimak'wanel q'anal)** (mg/dl*):

**Deseable**	**Menos de 100**
**Casi deseable/un poco más alto que deseable**	100-129
**Moderadamente alto**	130-159
**Muy alto**	160 o más

* Miligramos por decilitros. ** El nivel de colesterol LDL (qajnäq ruchuq'a' nimak'wanel q'anal) ideal difiere para cada persona. Su médico le puede ayudar a fijar su meta para su nivel de colesterol LDL (qajnäq ruchuq'a' nimak'wanel q'anal).

Los niveles de HDL (colesterol bueno) (jotöl ruchuq'a' nimak'wanel q'anal) mayores de 60 mg/dl ayudan a disminuir su riesgo (retzelal) para las enfermedades del corazón (taq ruyab'ilal k'uxaj). Los niveles de HDL (jotöl ruchuq'a' nimak'wanel q'anal) menores de 40 mg/dl son uno de los principales factores de riesgo (taq rub'anikil rutzilal) para las enfermedades del corazón (taq ruyab'ilal k'uxaj). Un nivel normal de triglicéridos (ruwäch meq'anal) es menor de 150 mg/dl.

LO QUE USTED PUEDE HACER PARA PREVENIR LAS ENFERMEDADES DEL CORAZÓN (TAQ RUYAB'ILAL K'UXAJ)

- Hágase una prueba de sangre llamada perfil de lipoproteínas en el consultorio de su médico. Esta prueba mide todos los niveles de colesterol (saqab'uyül q'anal) (total, LDL (qajnäq ruchuq'a' nimak'wanel q'anal) y HDL (jotöl ruchuq'a' nimak'wanel q'anal)) así como los triglicéridos (ruwäch meq'anal).
- Si es mayor de 20 años, mídase el nivel de colesterol (saqab'uyül q'anal) en la sangre cada 5 años.
- Aprenda lo que significan sus niveles de colesterol (saqab'uyül q'anal). Si están altos, pregunte a su médico qué puede hacer para bajarlos.
- Elija alimentos bajos en grasa saturada (ritzelal q'anal), grasa transaturada (nima'itzelal q'anal) y colesterol (saqab'uyül q'anal).
- Logre mantener un peso saludable. Manténgase físicamente activo, incluso a través del trabajo.

TOME ESTAS MEDIDAS PARA CONTROLAR LOS:

- Limite los dulces, postres, golosinas, gaseosas, jugos y otras bebidas con un contenido alto de azúcar.
- Evite fumar y si toma bebidas alcohólicas, hágalo en moderación. Fumar aumenta los triglicéridos (ruwäch meq'anal) y disminuye el colesterol HDL (jotöl ruchuq'a' nimak'wanel q'anal). El exceso de alcohol también eleva los triglicéridos (ruwäch meq'anal).

LA DIABETES (KAB'YAB'IL)

La diabetes (kab'yab'il) ocurre cuando el cuerpo no pueda controlar la cantidad de azúcar en la sangre. Por eso muchas veces hay un nivel alto de azúcar en la sangre y eso puede dañar a las otras partes del cuerpo, por ejemplo los riñones (kinäq'), ojos y corazón.

LO QUE USTED NECESITA SABER

- La diabetes (kab'yab'il) es grave. Usted puede tenerla y no saberlo. Puede causar un ataque al corazón (rukamik k'uxaj) o un ataque al cerebro (rukamik tzatzq'or), ceguera, amputaciones y enfermedad de los riñones (kinäq').
- Tener sobrepeso es uno de los principales factores de riesgo (rub'anikil rutzilal) para la diabetes (kab'yab'il).

LO QUE USTED PUEDE HACER PARA PREVENIR LAS ENFERMEDADES DEL CORAZÓN (TAQ RUYAB'ILAL K'UXAJ)

- Averigüe si tiene diabetes (kab'yab'il).
- Mída su nivel de azúcar en la sangre por lo menos cada 3 años comenzando cuando cumpla 45 años.
- Debe medírselo a una edad más joven o más a menudo si está en riesgo (retzelal) de desarrollar la diabetes (kab'yab'il).

INACTIVIDAD FÍSICA

La falta de actividad física puede doblar su posibilidad de desarrollar enfermedades del corazón (taq ruyab'ilal k'uxaj) quitándole años a su vida.

LO QUE USTED NECESITA SABER

- Los adultos deben hacer por lo menos 30 minutos de actividad física moderada preferiblemente todos los días.
- Algunos adultos necesitan hasta 60 minutos de actividad física moderada (un nivel mediano) a vigorosa (alto nivel) la mayoría de los días para prevenir un aumento de peso.
- Los adultos que han tenido sobrepeso necesitan de 60 a 90 minutos de actividad física moderada todos los días para evitar volver a subir de peso.
- Los niños y los adolescentes necesitan por lo menos 60 minutos de actividad física moderada o vigorosa casi todos los días.

LO QUE USTED PUEDE HACER PARA PREVENIR LAS ENFERMEDADES DEL CORAZÓN (TAQ RUYAB'ILAL K'UXAJ)

- Deje de fumar ahora o disminuya poco a poco.
- Si no puede dejar de fumar la primera vez que lo trata, siga intentándolo.
- Si no fuma, no empiece.

HÁBITO DE FUMAR

Usted pone en peligro su salud y la de su familia cuando fuma.

LO QUE USTED NECESITA SABER

- Fumar cigarrillos crea adicción.
- Daña al corazón y a los pulmones. Puede elevar la presión aterial (ruchuq'a' kïk') y el colesterol (saqab'uyül q'anal) en la sangre.

LO QUE USTED PUEDE HACER PARA PREVENIR LAS ENFERMEDADES DEL CORAZÓN (TAQ RUYAB'ILAL K'UXAJ)

- Deje de fumar ahora o disminuya poco a poco.
- Si no puede dejar de fumar la primera vez que lo trata, siga intentándolo.
- Si no fuma, no empiece.

FACTORES DE RIESGO PARA LAS ENFERMEDADES DEL CORAZÓN QUE USTED PUEDE CONTROLAR

Los factores de riesgo son características o hábitos que aumentan la probabilidad de que una persona desarrolle cierta enfermedad.

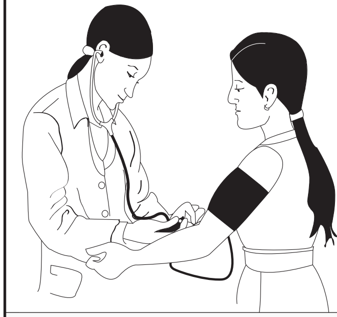

presión aterial alta

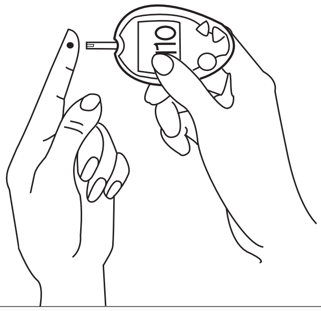

la diabetes

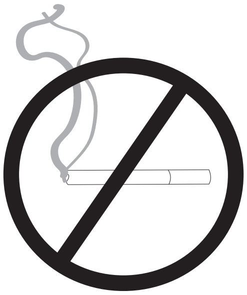

hábito de fumar

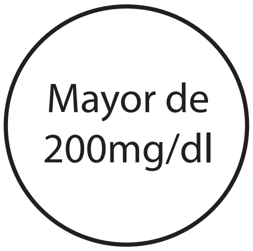

nivel alto de colesterol

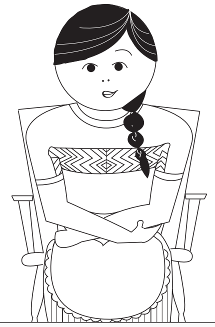

inactividad física

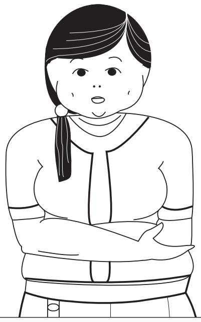

sobrepeso

FACTORES DE RIESGO PARA LAS ENFERMEDADES DEL CORAZÓN QUE USTED NO PUEDE CAMBIAR O CONTROLAR:

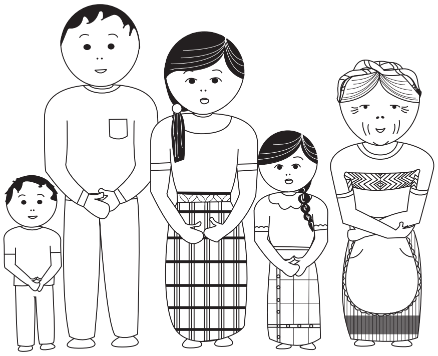

- Edad
  Hay más riesgo cuando son mayores.
- Historia familiar:
  - Familia con enfermedades del corazón
  - Joven en la familia con enfermedades del corazón antes de los 65 años

Mientras más factores de riesgo tenga mayor será su riesgo de desarrollar enfermedades del corazón taq ruyab'ilal k'uxaj. Hable con su médico acerca de su riesgo.

LA CAPACITACIÓN SOBRE LA SALUD DEL CORAZÓN

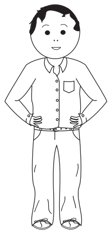

1. ESTÁ USTED EN RIESGO DE DESARROLLAR ENFERMEDADES DEL CORAZÓN?

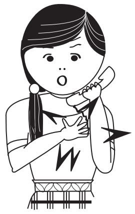

2. ACTUÉ RÁPIDO FRENTE A LOS SÍNTOMAS DE UN ATAQUE AL CORAZÓN.

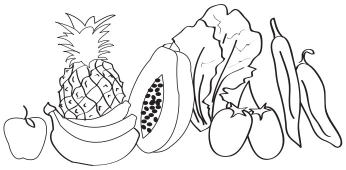

3. PIENSE EN SU CORAZÓN: DÍGALE SÍ A LA ALIMENTACIÓN SALUDABLE Y LA ACTIVIDAD FÍSICA.

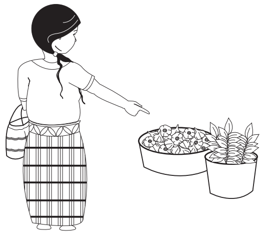

4. AYUDE A SU CORAZÓN: CONTROLE SU PRESIÓN ARTERIAL ALTA.

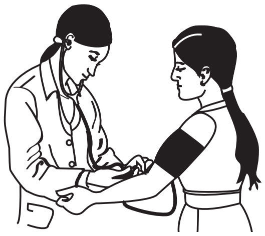

5. SEA BUENO CON SU CORAZÓN: MANTENGA SU COLESTEROL BAJO CONTROL.

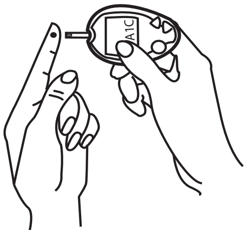

6. PROTEJA SU CORAZÓN: CUIDESE DE LA DIABETES.

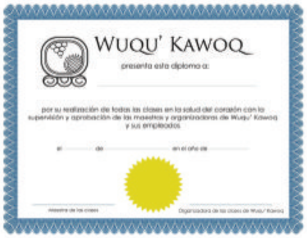

7. REPASO Y GRADUACIÓN.

1.2

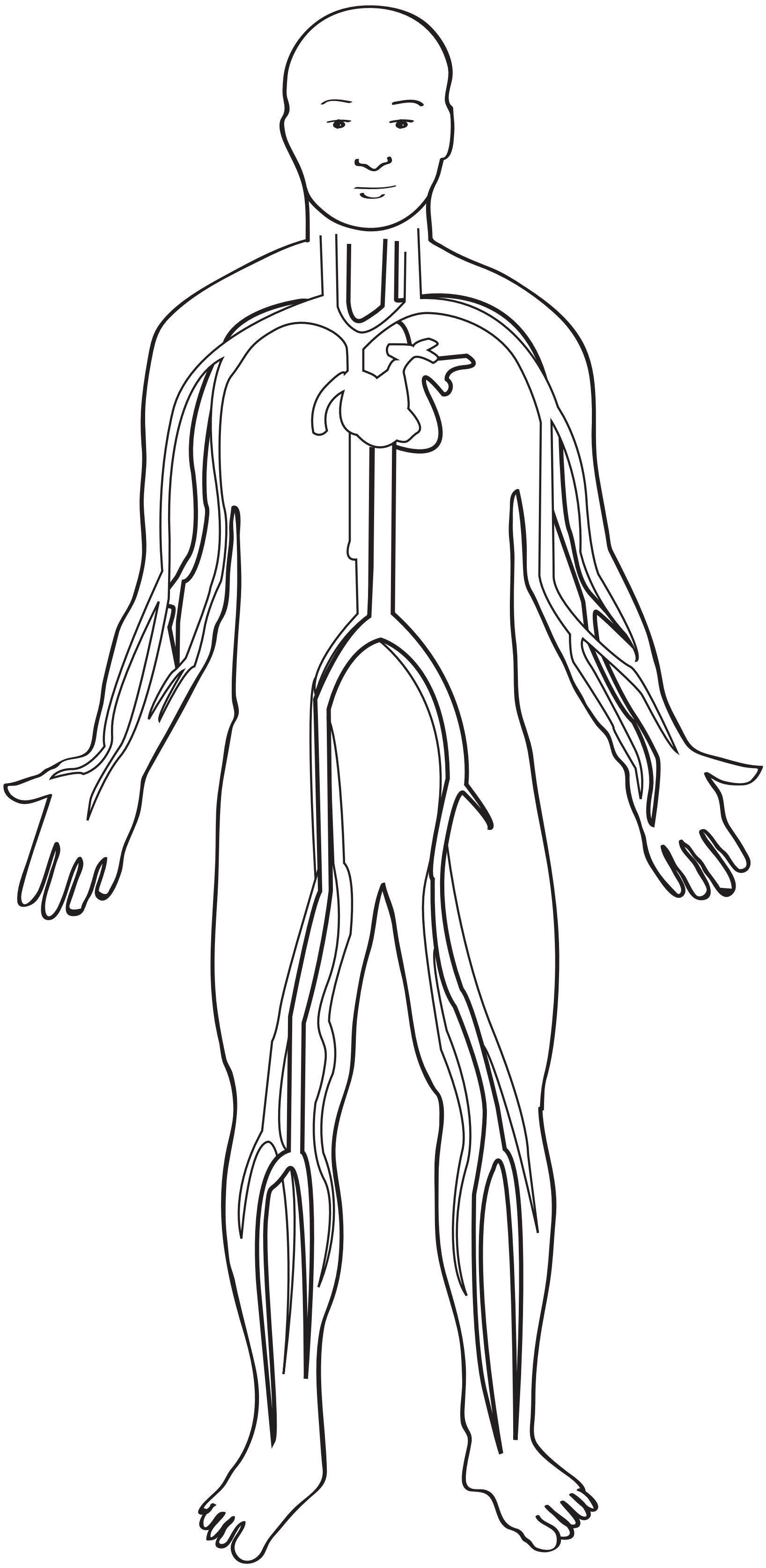

UN DÍA CON LA FAMILIA COJTÍ

Las personas actúan de diferentes maneras cuando se dan cuenta que tienen que hacer cambios para disminuir su riesgo de desarrollar enfermedades del corazón. Veamos cómo la familia Cojtí actúa cuando se entera de su riesgo.

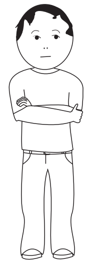

Xwan: "*A mí no me importa*".

Xwan se entera que tiene riesgo de desarrollar enfermedades del corazón, pero a él no le preocupa demasiado. Se molesta cuando le recuerdan que debe cambiar sus hábitos dañinos. Él no le pedirá a su esposa para que prepare la comida de manera diferente y le gusta comer comida de la calle a menudo. No tiene ninguna intención de hacer cambios para mejorar su salud. "Lo que será, será", dice Xwan.

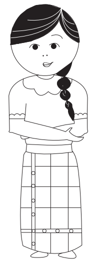

Ixkotz'i'j: "*¡No logro dar el primer paso!*"

Ixkotz'i'j está preocupada porque se ha enterado de su riesgo de desarrollar enfermedades del corazón. Ella ha pensado hacer algunos cambios pero no logra comenzar a hacerlos. Se siente indecisa y no está motivada a tomar medidas para mejorar la salud. "El que no oye consejos, no llega a viejo", teme Ixkotz'i'j.

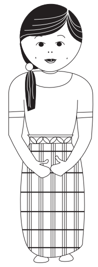

Ixchel: "*Estoy haciendo planes.*"

Ixchel sabe que está en riesgo de desarrollar enfermedades del corazón y cree que debe prepararse para hacer cambios. En vez de solo decir que comenzará a hacer cambios, ella prepara platillos saludables con menos grasa y se compra un par de zapatos para salir a caminar. Ella nunca toma un tuk tuk pero en lugar de eso camina a la Iglesia, a su trabajo, y al mercado. "Del dicho al hecho hay mucho trecho", piensa Ixchel.

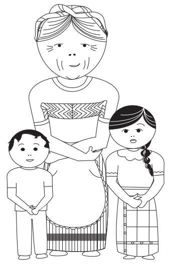

Nan Nikte: "*Mis nietos me motivan.*"

Pobreza entró en mi casa cuando me enteré de mi diabetes. Ella no podía trabajar y había menos comida para su familia. Hay tantas difcultades a causa de su enfermedad. Sus nietos crean esperanza para ella. Tiene un médico que la ayuda y se preocupa. Nan Nikte dice, "Voy a estar aquí para ver a mis nietos crecer.

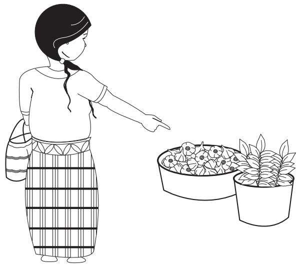

Ixkem: "*Estoy cambiando de rutina.*"

Apenas Ixkem supo que corría riesgo de enfermarse del corazón, está cambiando de rutina. Ahora, va a clases para aprender cómo mejorar su salud y pone en práctica lo que aprende. Tiene confianza y está contenta. Ella hace cambios sencillos y ayuda a que otros hagan lo mismo. Ixkem dice, "Más vale prevenir que lamentar".

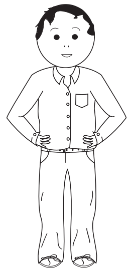

Lu': "*Me mantengo en el camino de la salud.*"

Lu' está muy motivado y hace cambios por el bien de su salud. Él sabe que es fácil regresar a los viejos hábitos. Su esposa es una fuerza motivador que ayuda mantener su buena salud diariamente. Hace planes para prevenir las recaídas y aprende a comenzar de nuevo cuando es necesario. Tiene una actitud positiva hacia la vida. Él pide ayuda y no se da por vencido.

SESIÓN 2

Actúe rápido frente a los síntomas (taq k'u't) de un ataque al corazón (rukamik k'uxaj)

OBJETIVOS

AL FINALIZAR ESTA SESIÓN, LOS PARTICIPANTES CONOCERÁN:

- Lo que es ataque al corazón (rukamik k'uxaj)
- Por qué es importante buscar tratamiento de inmediato en caso de un ataque al corazón (rukamik k'uxaj)
- Los síntomas (taq k'u't) de un ataque al corazón (rukamik k'uxaj)
- Por qué algunas personas se demoran en buscar ayuda en caso de un ataque al corazón (rukamik k'uxaj)
- Los beneficios de llamar a los servicios médicos de emergencia a los bomberos (taq chupüy q'aq') (122 y 123)
- Cómo prepararse por adelantado

MATERIALES

PARA DIRIGIR ESTA SESIÓN USTED NECESITARÁ:

- Pizarrón y marcadores o varias hojas grandes de papel, un marcador y cinta adhesiva

HOJAS PARA REPARTIR:

DURANTE LA SESIÓN, ENTREGUE A LOS PARTICIPANTES ESTA HOJA:

- "Lo que se siente al tener un ataque al corazón" (página 24)

CARTELES PARA MOSTRAR:

- Cartel derivado de dibujo educativo 2.1 (página 29)
- Cartel derivado de la hoja "Lo que se siente al tener un ataque al corazón" (página 24)
- Cartel derivado de la hoja "La acción rápida salva vidas" (página 25)

ESQUEMA DE LA SESIÓN

INTRODUCCIÓN A LA SESIÓN

1. Bienvenida
2. Repaso de la sesión de la semana anterior
3. Acerca de esta sesión

LA SESIÓN EN ACCIÓN

1. Los hechos importantes
2. ¿Qué es un ataque al corazón (rukamik k'uxaj)?
3. La importancia del tratamiento rápido (aninäq to'ïk) en caso de un ataque al corazón (rukamik k'uxaj)
4. ¿Cuáles son los síntomas (taq k'u't) de un ataque al corazón (rukamik k'uxaj)? La demora puede ser fatal
5. La función de los servicios médicos de emergencia
6. Cómo prepararse por adelantado
7. Acciones para alegrar el corazón

REPASO DE LOS PUNTOS MÁS IMPORTANTES DE HOY

TAREA SEMANAL

CIERRE DE LA SESIÓN

INTRODUCCIÓN A LA SESIÓN

1 | BIENVENIDA

- Dé la bienvenida a los participantes a la sesión.

2 | REPASO DE LA SESIÓN DE LA SEMANA ANTERIOR

DIGA:

· En la sesión anterior, hablamos sobre los factores de riesgo (rub'anikil rutzilal) para las enfermedades del corazón (taq ruyab'ilal k'uxaj).

PREGUNTE:

NOTA : *Dé a los participantes unos 3 minutos para responder.*

· ¿Quién recuerda los factores de riesgo (rub'anikil rutzilal) que podemos prevenir o controlar?

AÑADA LOS SIGUIENTES FACTORES SI NADIE LOS MENCIONA:

· la presión arterial alta (jotol ruchuq'a' kïk')
· un nivel alto de colesterol (jotöl ruchuq'a' ri saqab'uyül q'anal) en la sangre
· el hábito de fumar
· la diabetes (kab'yab'il)
· el sobrepeso
· la inactividad física

PREGUNTE:

NOTA : *Dé a los participantes unos 2 minutos para responder.*

· ¿Alguno de ustedes quisiera compartir lo que está haciendo para mejorar la salud de su corazón?

3 | ACERCA DE ESTA SESIÓN

DIGA:

· Las enfermedades del corazón (taq ruyab'ilal k'uxaj) toman muchos años para desarrollarse. Algunas veces, a pesar de nuestros mejores esfuerzos, las enfermedades del corazón (taq ruyab'ilal k'uxaj) pueden resultar en un ataque al corazón (rukamik k'uxaj). Por eso es importante saber cuáles son los síntomas (taq k'u't) de un ataque al corazón (rukamik k'uxaj) y qué deben hacer si los sienten. En la sesión de hoy, hablaremos sobre los síntomas (taq k'u't) y la importancia de actuar rápidamente si alguno de ustedes siente estos los síntomas (taq k'u't). Al final de esta sesión ustedes sabrán:

· Cómo funciona el corazón
· Lo que es un ataque al corazón (rukamik k'uxaj)
· Por qué es importante buscar tratamiento de inmediato (aninäq to'ïk) en caso de un ataque al corazón (rukamik k'uxaj)
· Los síntomas (taq k'u't) de un ataque al corazón (rukamik k'uxaj)
· Por qué algunas personas se demoran en buscar ayuda en caso de un ataque al corazón (rukamik k'uxaj)
· Los beneficios de llamar a los servicios médicos de emergencias 122 y 123.

LA SESIÓN EN ACCIÓN

1 | LOS HECHOS IMPORTANTES

DIGA:

· Cada año miles de guatemaltecos(as) tendrán un ataque al corazón (rukamik k'uxaj) y alrededor de la mitad de ellos morirán.

· Aproximadamente la mitad de las personas que mueren de un ataque al corazón (rukamik k'uxaj), mueren antes de llegar al hospital (aq'omab'äl jay).

HAGA : *Muestre el dibujo educativo 2.1 con el cartel (página 29).*

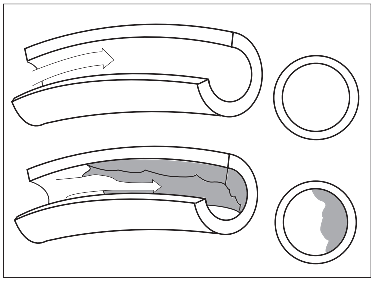

2 | ¿QUÉ ES UN ATAQUE AL CORAZÓN (RUKAMIK K'UXAJ)?

DIGA:

· Un ataque al corazón (rukamik k'uxaj) ocurre cuando se bloquea una de las arterias (kaqib'och'ilal) por donde pasa la sangre que va a una parte del corazón. Veamos cómo sucede esto.

· Las enfermedades que pueden causar un ataque al corazón (rukamik k'uxaj), se desarrollan a través del tiempo. La grasa (q'anal) se acumula dentro de las arterias (kaqib'och'ilal). Cuando esto pasa, las arterias (kaqib'och'ilal) se estrechan y no pueden pasar suficiente sangre, oxígeno (uxlab'il) y nutrientes para responder a las necesidades del corazón.

· Las arterias (kaqib'och'ilal) generalmente se obstruyen por un coágulo (ch'ojch'äq). Cuando esto pasa, se corta el flujo de sangre (rutz'ujal kïk') y comienza el ataque al corazón (rukamik k'uxaj).

· Si la obstrucción continúa, parte del músculo del corazón (ruti'ojilal k'uxaj) comenzará a morir.

DIGA:

· Cuando ocurre un ataque al corazón (rukamik k'uxaj), el tratamiento médico puede restaurar el flujo de sangre (rutz'ujal kïk'), impidiendo así que muera parte del músculo del corazón (ruti'ojilal k'uxaj).

· El tratamiento funciona mejor cuando se da lo más pronto posible, dentro de la primera hora después de que comienzan los síntomas (taq k'u't) de un ataque al corazón (rukamik k'uxaj).

· Una vez que parte del músculo del corazón (ruti'ojilal k'uxaj) muere, no hay nada que se pueda hacer para repararlo.

PREGUNTE:

NOTA : *Dé a los participantes un momento para responder.*

· ¿Tienen alguna pregunta sobre lo qué pasa durante un ataque al corazón (rukamik k'uxaj)?

3 | LA IMPORTANCIA DE DAR TRATAMIENTO RÁPIDO (ANINÄQ TO'ÏK) PARA UN ATAQUE AL CORAZÓN (RUKAMIK K'UXAJ)

DIGA:

· Los tratamientos para los ataques al corazón (taq rukamik k'uxaj) han mejorado con los años. Hace 20 años, no había mucho que se podía hacer para detener un ataque al corazón (rukamik k'uxaj).

- Hoy en día hay tratamientos que pueden abrir las las arterias (kaqib'och'ilal) del corazón, restaurando el flujo de sangre (rutz'ujal kïk').
- Los medicamentos que "rompen" coágulos (ch'ojch'äq) y otros tratamientos para abrir las arterias (kaqib'och'ilal) pueden disolver coágulos (ch'ojch'äq) y restaurar rápidamente el flujo de sangre (rutz'ujal kïk') al corazón.

DIGA:

- Estos tratamientos se deben dar lo más pronto posible, dentro de una hora del comienzo de los síntomas (taq k'u't).
- Mientras más músculo del corazón (ruti'ojilal k'uxaj) se salve, mejor es la probabilidad de que el paciente sobreviva y vuelva a su vida normal.

4 | ¿CUÁLES SON LOS SÍNTOMAS (TAQ K'U'T) DE UN ATAQUE AL CORAZÓN (RUKAMIK K'UXAJ)?

DIGA:

- Es importante saber reconocer los síntomas (taq k'u't) de un ataque al corazón (rukamik k'uxaj). Vamos a hacer una actividad que les ayudará a aprender cuáles son los síntomas (taq k'u't) de un ataque al corazón (rukamik k'uxaj).

PREGUNTE:

- ¿Qué los síntomas (taq k'u't) les harían a ustedes pensar que alguien está teniendo un ataque al corazón (rukamik k'uxaj)?

NOTA : *Escriba las respuestas de los participantes en el pizarrón o en un papelógrafo a la pared con cinta adhesiva.*

HAGA : *Entregue a cada participante la hoja "Lo que se siente al tener un ataque al corazón" (página 24). Muestre el cartel derivado de la hoja "Lo que siente al tener un ataque al corazón" y lea la hoja que entregó (página 24).*

DIGA:

- Estos son algunos de los síntomas (taq k'u't) que se reportan con más frecuencia por hombres y mujeres que han tenido un ataque al corazón (rukamik k'uxaj):

DIGA:

- El síntoma (k'u't) más común de un ataque al corazón (rukamik k'uxaj), tanto para hombres como para mujeres, es dolor o molestia en el pecho.
- No es necesario tener todos los síntomas (taq k'u't) para tener un ataque al corazón (rukamik k'uxaj).
- Los síntomas (taq k'u't) mencionados son los más comunes y la mayoría de las personas sienten más de uno de ellos. Algunos síntomas (taq k'u't) pueden ocurrir a la misma vez. Por ejemplo, la molestia en el pecho a menudo ocurre con dificultad para respirar. Asimismo, el dolor en los brazos, la sudoración y la náusea pueden ocurrir juntos.

DIGA:

- Muchas personas creen que todos los ataques al corazón (taq rukamik k'uxaj) ocurren como la persona siente un dolor aplastante en el pecho y se cae al suelo. Esto es un mito. La realidad es que no todos los ataques al corazón (taq rukamik k'uxaj) ocurren de esta manera. Muchos ataques al corazón (taq rukamik k'uxaj) comienzan lentamente, y los síntomas (taq k'u't) se hacen más fuertes gradualmente.
- Un ataque al corazón (rukamik k'uxaj) no siempre es un evento repentino que causa la muerte. Hay una variedad de síntomas (taq k'u't) que pueden ser un aviso de que una persona está en peligro.
- A veces el dolor o la molestia es leve y los síntomas (taq k'u't) pueden ir y venir.

NOTA : *Dé a los participantes unos 2 minutos para contestar. Escriba sus repuestas en el pizarrón o en un papelógrafo.*

PREGUNTE:

- ¿Por qué razones creen ustedes que las personas no saben que están teniendo un ataque al corazón (rukamik k'uxaj)?

AÑADA LAS SIGUIENTES RAZONES SI NO SE MENCIONAN:

- Las personas pueden confundir los síntomas (taq k'u't) de un ataque al corazón (rukamik k'uxaj) con los síntomas (taq k'u't) de otras enfermedades como artritis (rusipojik laqul), asma (tz'apipospo'y), cáncer (sachöy jotay) o insuficiencia cardiaca (chajinel k'ajkab') (cuando el corazón ya no puede bombear suficiente sangre al resto del cuerpo).
- Las personas pueden confundir los síntomas (taq k'u't) de un ataque al corazón (rukamik k'uxaj) con los síntomas (taq k'u't) de un músculo jalado (jek'on ti'ojilal), indigestión (rusachoj pamaj) o la gripe.
- Algunas personas no quieren creer que están en riesgo (retzelal) de un ataque al corazón (rukamik k'uxaj). Cuando tienen algún síntoma (k'u't), lo ignoran o esperan a ver si los síntomas (taq k'u't) empeoran antes de pedir ayuda.

5 | ES IMPORTANTE LLAMAR A LOS BOMBEROS (TAQ CHUPÜY Q'AQ') O LLEVAR A UN AYUDANTE DE SALUD

DIGA:

- Muchas veces, la gente espera demasiado antes de buscar atención médica cuando siente síntomas (taq k'u't) de un ataque al corazón (rukamik k'uxaj).
- Antes de buscar ayuda, algunas personas esperan de 2 a 4 horas, un día o aún más.
- Si cree que usted o alguien cercano está teniendo un ataque al corazón (rukamik k'uxaj), lo más importante que debe hacer es llamar a los bomberos (taq chupüy q'aq') o llevar a la persona inmediatamente al Servicio de Salud más cercano.
- Cuando por fin deciden pedir ayuda, las personas que están sufriendo un ataque al corazón (rukamik k'uxaj) a menudo toman acciones inmediatas. Solamente la mitad de todos los pacientes con ataque al corazón (rukamik k'uxaj) llegan al hospital (aq'omab'äl jay) en ambulancia (ruch'ich' aq'omab'äl).

HAGA : *Muestre el cartel derivado de la hoja "La acción rápida salva vidas" (página 25). Lea en alta voz.*

DIGA:

- Repasemos cómo actuar rápidamente cuando crean que alguien está teniendo un ataque al corazón (rukamik k'uxaj).

NOTA : *Dé a los participantes unos 2 minutos para que respondan.*
HAGA : *Escriba sus respuestas en el pizarrón o en un papelógrafo.*

PREGUNTE:

- Piensen en ustedes y en sus familias. ¿Qué les impediría llamar los bomberos (taq chupüy q'aq') rápidamente si piensan que están teniendo un ataque al corazón (rukamik k'uxaj)?

AÑADA ESTAS RAZONES SI NO LAS MENCIONAN:

- No pensaban que sus síntomas (taq k'u't) eran suficientemente graves.
- No sabían todas las ventajas para salvar vidas que tiene el llamar a los números 122 y 123 de los bomberos (taq chupüy q'aq'). Es como traer la sala de emergencia a su casa.
- Pensaban que sería más rápido conducirse ellos mismos al hospital (aq'omab'äl jay).

- Les preocupaba sentir vergüenza cuando un vehículo de emergencia (una ambulancia (ruch'ich' aq'omab'äl) o camión de bomberos (taq chupüy q'aq')) llegue a sus puertas.
- Les preocupaba el costo de la ambulancia (ruch'ich' aq'omab'äl) y de la atención médica.
- Les daba miedo avergonzarse si los síntomas (taq k'u't) eran una falsa alarma.

DIGA:

· Si usted está teniendo un ataque al corazón (rukamik k'uxaj) y ni usted ni nadie más puede llamar a los números 122 y 123 pídale a alguien que lo lleve al hospital (aq'omab'äl jay) de inmediato. Nunca conduzca usted mismo. Puede perder el conocimiento en el camino, poniéndose en peligro usted mismo y a otros.

· Es fácil conversar sobre lo que se haría si ocurre un ataque al corazón (rukamik k'uxaj), pero no es tan fácil hacer lo correcto si usted o alguien conocido realmente está teniendo los síntomas (taq k'u't). Ahora vamos a tomarnos el tiempo de hacer unas dramatizaciones que los prepararán en caso de que la situación les ocurra en la vida real.

NOTA : *Pida voluntarios del grupo que forme un equipo con usted. Dé a los voluntarios una de las tres hojas con las diferentes dramatizaciones que acompañan la hoja "La acción rápida salva vidas" (páginas 26–28). Presenten todas las dramatizaciones para el grupo. Tomen unos minutos para mirar todas las dramatizaciones.*

NOTA : *Dé a los participantes un momento para responder.*

PREGUNTE:

· Ahora que han visto las tres escenas, ¿qué piensan que es lo más importante que deben hacer cuando alguien tiene los síntomas (taq k'u't) de un ataque al corazón (rukamik k'uxaj)?

SI NADIE DICE LA REPUESTA CORRECTA, DIGA:

· La respuesta es llamar a los bomberos (taq chupüy q'aq') en menos de 5 minutos.

6 | LA FUNCIÓN DE LOS SERVICIOS MÉDICOS DE EMERGENCIA

DIGA:

· Nuestros actores hicieron un gran trabajo demostrándonos la importancia de llamar a los bomberos (taq chupüy q'aq') inmediatamente. Llamando a los números 122 y 123 es como traer la sala de emergencia a su casa.

NOTA : *Dé a los participantes unos 3 minutos para responder.*

PREGUNTE:

· ¿Qué otros beneficios se tienen al llamar a los servicios médicos de emergencia?

AÑADA ESTAS RESPUESTAS SI NO LAS MENCIONAN:

· Los bomberos (taq chupüy q'aq') llegan rápidamente y pueden comenzar el tratamiento de inmediato. Traen oxígeno (uxlab'il), medicamentos para el corazón, para aliviar el dolor y otros más, como la aspirina (raq'om tunäy che'), que pueden ayudar en caso de un ataque al corazón (rukamik k'uxaj).

· En muchos lugares, los paramédicos (achaq'omanela') están vinculados con un hospital (aq'omab'äl jay). Pueden mandar información sobre la condición del paciente a la sala de emergencia aun antes de que el paciente llegue al hospital (aq'omab'äl jay).

- Los pacientes con un ataque al corazón (rukamik k'uxaj) que son llevados en ambulancia (ruch'ich' aq'omab'äl) reciben tratamiento más rápido cuando llegan al hospital (aq'omab'äl jay).
- Su corazón puede dejar de latir durante un ataque al corazón (rukamik k'uxaj). Los paramédicos (achaq'omanela') tienen el equipo necesario para hacer que el corazón vuelva a latir si eso ocurre.

DIGA:

- Es una buena idea planear por adelantado quién cuidará de su familia en una emergencia. Los paramédicos (achaq'omanela') generalmente llamarán a un pariente o amigo para que haga estos arreglos en caso de ser necesario.

PREGUNTE:

NOTA : *Dé a los participantes unos 5 minutos para que hablen sobre sus respuestas.*

- ¿Qué planes haría usted si tuviera que ir de repente al hospital (aq'omab'äl jay) en una ambulancia (ruch'ich' aq'omab'äl)?

7 | CÓMO PREPARARSE POR ADELANTADO

DIGA:

- Hemos aprendido mucho hoy sobre los ataques al corazón (taq rukamik k'uxaj) y qué hacer si ocurre alguno. Hay buenas noticias. Prepararse por adelantado con su familia le puede ayudar a sobrevivir un ataque al corazón (rukamik k'uxaj). A continuación les daré algunas medidas que pueden tomar ahora para estar preparados en caso de un posible ataque al corazón (rukamik k'uxaj).
- Ahora ya conocen los síntomas (taq k'u't) de un ataque al corazón (rukamik k'uxaj). Hablen con su familia y amigos sobre estos síntomas (taq k'u't) y la importancia de llamar a los bomberos (taq chupüy q'aq') de inmediato. Ustedes así como los demás adultos de la familia, deben hablar con el personal del centro de salud sobre el riesgo (retzelal) que tienen de un ataque al corazón (rukamik k'uxaj) y cómo disminuirlo.

NOTA : *Dé a los participantes un momento para responder en voz alta.*

- Pida a voluntarios que digan cómo explicarían a sus familias la importancia de hacer un plan de acción.
- Pida que den un ejemplo en caso de que los participantes estén en riesgo (retzelal) de tener un ataque al corazón (rukamik k'uxaj).
- Pida otros ejemplos en caso de que otro miembro de la familia como el abuelo, la esposa o un tío, corra riesgo (retzelal) de tener un ataque al corazón (rukamik k'uxaj).
- Pídales que digan cómo van a lograr que toda la familia sepa lo que deben hacer en caso de un ataque al corazón (rukamik k'uxaj).

REPASO DE LOS PUNTOS MÁS IMPORTANTES DE HOY

DIGA:

- Repasemos lo que aprendimos hoy.

PREGUNTE:

- ¿Qué es un ataque al corazón (rukamik k'uxaj)?

AÑADA LOS SIGUIENTES FACTORES SI NADIE LOS MENCIONA:

- Un ataque al corazón (rukamik k'uxaj) ocurre cuando una arteria (kaq'ib'öch') se obstruye y el flujo de sangre (rutz'ujal kïk') al corazón se corta. Si el flujo de sangre (rutz'ujal kïk') se corta, partes del músculo del corazón (ruti'ojilal k'uxaj) comienzan a morirse.

PREGUNTE:

- ¿Qué detiene un ataque al corazón (rukamik k'uxaj)?

AÑADA LOS SIGUIENTES FACTORES SI NADIE LOS MENCIONA:

- Acción y tratamiento médico rápido restauran el flujo de sangre (rutz'ujal kïk') y salvan al músculo del corazón (ruti'ojilal k'uxaj).

PREGUNTE:

- ¿Por qué es importante obtener tratamiento rápidamente?

AÑADA LOS SIGUIENTES FACTORES SI NADIE LOS MENCIONA:

- el tratamiento rápido (aninäq to'ïk) puede evitar que parte del músculo del corazón (ruti'ojilal k'uxaj) se muera.
- Es mejor recibir tratamiento dentro de la primera hora después de que comienzan los síntomas (taq k'u't). Esto mejorará la probabilidad de sobrevivir y ayudará a salvar el músculo del corazón (ruti'ojilal k'uxaj).
- Hay medicamentos para disolver los coágulos (ch'ojch'äq), que abren la arteria (kaq'ib'öch) y restauran el flujo de sangre (rutz'ujal kïk').

PREGUNTE:

- ¿Cuáles son algunos de los síntomas (taq k'u't) de un ataque al corazón (rukamik k'uxaj)?

AÑADA LOS SIGUIENTES FACTORES SI NADIE LOS MENCIONA:

- Presión en el pecho.
- Molestia en uno o ambos brazos, la espalda o el estómago.
- Molestia en el cuello o la mandíbula.
- Dificultad para respirar con molestia o sin molestia en el pecho.
- Malestar en el estómago o náusea.
- Mareo o sudor frío.

PREGUNTE:

- ¿Qué deberán hacer si experimentan estos síntomas (taq k'u't)?

AÑADA LOS SIGUIENTES FACTORES SI NADIE LOS MENCIONA:

- Llamen a los bomberos (taq chupüy q'aq') en 5 minutos, aún si no están seguros de tener un ataque al corazón (rukamik k'uxaj). Cuando llaman al 122 o 123, reciben tratamiento más rápido.
- No conduzca su carro al hospital (aq'omab'äl jay).

TAREA SEMANAL

DIGA:

- Hoy han aprendido mucho sobre cómo estar preparados si alguien tiene un ataque al corazón (rukamik k'uxaj). Ahora pensemos cómo pueden poner en práctica lo que aprendieron. Les pido que piensen en un cambio que pueden hacer en su vida diaria. Esto será su tarea semanal.

- Sean específicos sobre qué piensan hacer, cómo piensan hacerlo y cuándo comenzarán. Algunos ejemplos incluyen:

NOTA : *Dé a los participantes 3 minutos para que piensen en una tarea.*

  - En la próxima semana, piensen en lo que harìan si alguien tiene un ataque al corazón (rukamik k'uxaj) en la casa, en el trabajo, a media noche o en alguna otra situación.
  - Esta semana, hablen con su familia y amigos sobre los síntomas (taq k'u't) de un ataque al corazón (rukamik k'uxaj) y la necesidad de llamar a los números 122 y 123 de inmediato.
  - Hagan una cita en el próximo mes para hablar con el promotor de salud (tzijöy aq'omab'äl) sobre su riesgo (retzelal) de tener un ataque al corazón (rukamik k'uxaj).

(OPCIONAL) DIGA:

- Un valor personal es una cualidad que ustedes consideran valiosa o importante. Los valores personales les pueden ayudar a hacer cambios en su vida diaria para mejorar su salud.

(OPCIONAL) DIGA:

- Nuestro valor de hoy es la serenidad. La serenidad les ayuda a mantenerse calmados aun cuando se enfrentan a problemas o preocupaciones. Les puede ayudar a manejar el estrés (sachk'u'x) y la ira, dos cosas que pueden contribuir a aumentar el riesgo (retzelal) de un ataque al corazón (rukamik k'uxaj).

NOTA : *Dé a los participantes unos 5 minutos para que compartan sus ideas.*

(OPCIONAL) PREGUNTE:

- ¿Cómo puede la serenidad u otro valor personal ayudarles a cumplir con su tarea?

(OPTIONAL) DIGA:

- En la próxima sesión hablaremos sobre cómo les fue con su tarea.

CIERRE DE LA SESIÓN

NOTA : *Dé a los participantes un momento para ver si tienen algún comentario.*

(OPCIONAL) PREGUNTE:

- ¿Qué piensan de la sesión de hoy? ¿Qué creen ustedes que funcionó y qué no funcionó? ¿Qué cambiaron ustedes en sus vidas como resultado de lo que aprendieron en esta sesión?

DIGA:

- Gracias por asistir el día de hoy y completar esta sesión tan importante.

DIGA:

- En la próxima sesión hablaremos sobre la actividad fisica.

NOTA : *Reflexión para el presentador de la sesión. Piense en la sesión de hoy.*

LO QUE SE SIENTE AL TENER UN ATAQUE AL CORAZÓN

¡Actúe rápido! Llame a los bomberos. Puede salvar su vida!

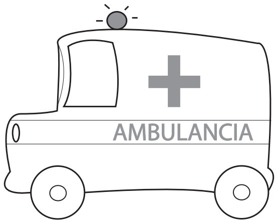

Hay tratamientos que pueden parar un ataque al corazón mientras ocurre. Estos tratamientos funcionan mejor cuando se dan dentro de la primera hora en que comienzan los síntomas. Si usted cree que está sufriendo un ataque al corazón, llame a los bomberos de inmediato.

CONOZCA LOS SÍNTOMAS DE UN ATAQUE AL CORAZÓN

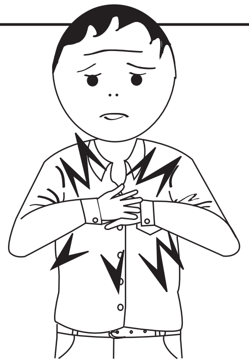

Presión en el pecho

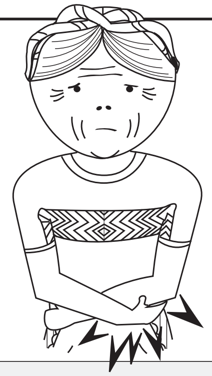

Molestia o incomodidad en uno o ambos brazos, la espalda o el estómago

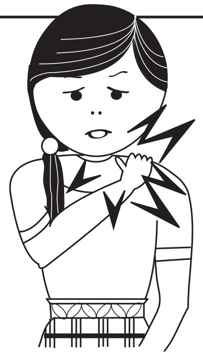

Molestia o incomodidad en el cuello o en la mandíbula

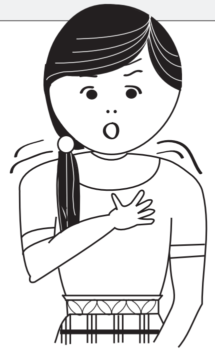

Dificultad para respirar

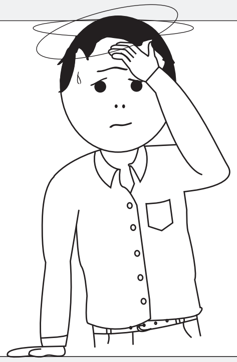

Sensación de mareo o sudor frío

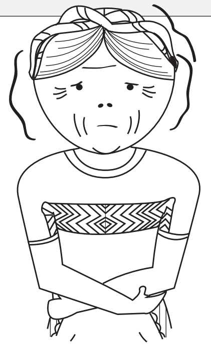

Malestar en el estómago o náusea

LA ACCIÓN RÁPIDA SALVA VIDAS

Es posible que dude que esté teniendo un ataque al corazón

Un ataque al corazón no tiene que ser repentino ni muy doloroso. Tal vez no sepa lo que le está pasando. Pero es importante buscar ayuda de inmediato.

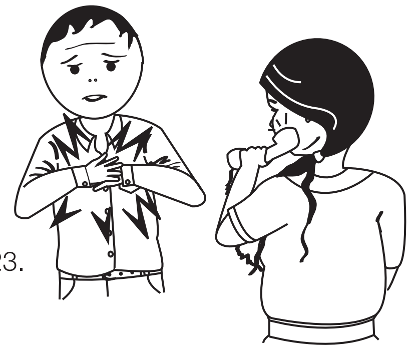

¡Actúe rápido! Llame al 123.

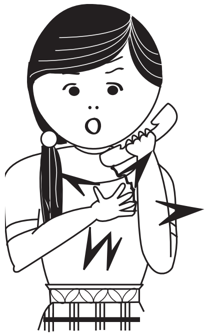

Llame al 123 en menos de 5 minutos.
No conduzca usted mismo al hospital.

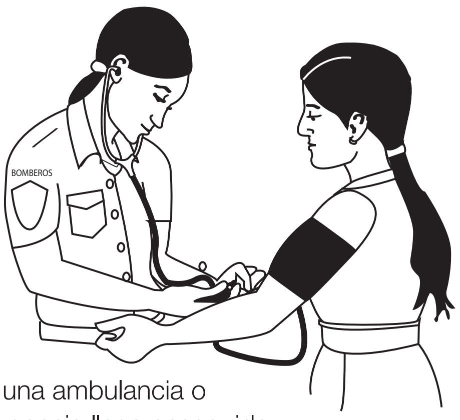

Al llamar al 123, una ambulancia o vehículo de emergencia llega enseguida. El tratamiento puede comenzar de inmediato.

MEDIDAS QUE AYUDAN A SOBREVIVIR UN ATAQUE AL CORAZÓN

- Aprenda los síntomas de un ataque al corazón y actúe rápido si los siente.
- Hable con su familia y amigos sobre estos síntomas y la necesidad de llamar al 123 de inmediato.
- Pregúntele al médico cuál es su riesgo de tener un ataque al corazón y cómo lo puede disminuir.

Dramatización 1 : En La Casa

Actor 1:

Usted está en la casa desayunando con su esposo(a). Le dice a su esposo(a) que se despertó sintiéndose algunas molestias. Usted tiene una variedad de síntomas, incluyendo:

- Siente presión o molestia en su pecho
- Le duele el brazo
- Siente que le falta el aire
- Se siente un poco mareado

Actor 2:

Tiene cara de preocupado(a). No está seguro que pasa, pero su esposo(a) luce enfermo(a). Le dice a su esposo(a) que hace poco escuchó hablar sobre los síntomas de un ataque al corazón y que usted está preocupado(a) de que eso pueda ser el problema. Le dice que tal vez lo mejor es llamar a los números 122 y 123.

Actor 1:

Usted insiste que no es nada, probablemente un poco de indigestión. Que ya pasará y usted estará bien.

Actor 2:

Usted contesta informándole a su esposo(a) por qué es importante llamar a los números 122 y 123 de inmediato:

- Aun cuando no se está seguro que sea un ataque al corazón, es mejor buscar ayuda.
- Si es un ataque al corazón, el tratamiento rápido puede prevenir daños al corazón.
- Llegar rápidamente al hospital significa que se puede comenzar el tratamiento de inmediato y tal vez salvar su vida.

Actor 1:

Usted está en su trabajo una tarde cuando se da cuenta que su compañero de trabajo no luce bien. Le pregunta si algo le pasa.

Actor 2:

Usted contesta que vino a trabajar esa mañana sintiéndose un poco mal. Usted describe los siguientes:

- Presión o molestia en medio del pecho
- Dificultad para respirar
- Malestar en el estómago
- Mareo
- Un dolor que le recorre el brazo izquierdo

Actor 1:

Usted le dice que ha escuchado que estos síntomas pueden significar un ataque al corazón. De ser así, lo mejor es que lo examinen de inmediato en el hospital. Usted ofrece llamar a los números 122 y 123.

Actor 2:

Usted le da a su compañero muchas razones de por qué esto no es necesario:

- Usted comió demasiado en el almuerzo. Sólo es indigestión, no es nada grave.
- No quiere hacer una escena en el trabajo y que todos se preocupen.
- Quiere esperar a ver si el dolor desaparece después de un rato.
- Si se va al hospital, no habrá quien recoja a su nieto de la guardería.

Actor 1:

Le dice a su compañero por qué es importante llamar a los números 122 y 123 de inmediato y hace la llamada.

Dramatización 3 : De Noche

Actor 1:

Usted está en casa una noche leyendo una revista cuando de repente comienza a sentirse muy enfermo. Llama al vecino por teléfono para decirle que no se siente bien. Le describe los siguientes:

- Repentinamente siente un dolor muy fuerte en medio del pecho.
- Le falta el aire y está comenzando a sudar frío

Usted cree que es algo malo y cree que debe conducir hasta el hospital.
Le pide a su vecino que lo acompañe.

Actor 2:

Usted le dice a su vecino que está preocupado porque estos síntomas se parecen a los de un ataque al corazón. Le dice que en este caso es mejor llamar al número 122 o 123 en vez de conducir él mismo, para así llegar al hospital sin peligro y ser tratado de inmediato.

Actor 1:

Usted protesta diciendo que no quiere despertar a todo el vecindario y causar una escena con la sirena y las luces y que es más fácil conducir.

Actor 2:

Usted le dice a su vecino por qué es mejor llamar a los servicios médicos de emergencia.

- El personal de emergencia puede comenzar el tratamiento médico de inmediato.
- Si su corazón se para, el personal de emergencia lo puede revivir.
- Los pacientes que tienen un ataque al corazón y llegan por ambulancia tienden a recibir un tratamiento más rápido cuando llegan al hospital.

Actor 1:

Usted reconoce que su vecino tiene razón. Le pide a su vecino que llame al número 122 o 123 inmediatamente y que después venga a hacerle compañía.

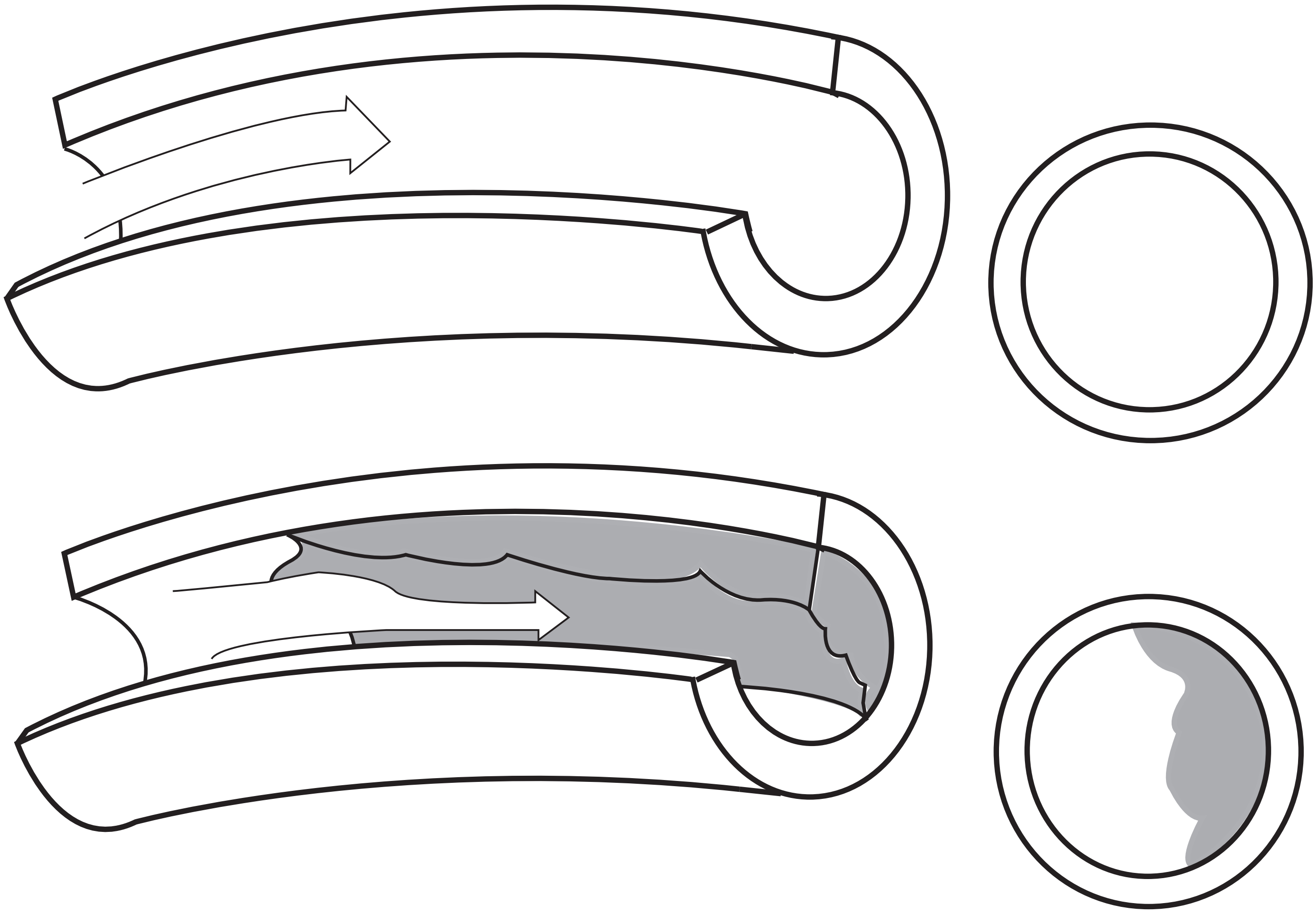

SESIÓN 3

Piense en su corazón:
Dígale SÍ a la alimentación saludable y la actividad física

OBJETIVOS

AL FINALIZAR ESTA SESIÓN, LOS PARTICIPANTES CONOCERÁN:

- Los tres grupos para una cada comida saludable
- Cómo consumir porciones saludables
- Cómo escoger alimentos para tener un corazón sano (raxnaqil k'uxaj)
- Que la actividad física es buena para el corazón y para la salud en general
- Caminar a paso ligero es una actividad física sencilla que pueden hacer casi todas las personas
- Hay distintas maneras de incorporar más actividad física dentro de la rutina diaria

MATERIALES

PARA DIRIGIR ESTA SESIÓN USTED NECESITARÁ:

- Pizarrón, varias hojas grandes de papel, marcadores y cinta adhesiva
- un juego de tazas para medir un cantidad de fideos, arroz, fresco o aceite vegetal

HOJAS PARA REPARTIR:

DURANTE LA SESIÓN, ENTREGUE A LOS PARTICIPANTES ESTA HOJAS:

- "Identificar alimentos saludables" (página 43)
- "¡Se necesitan las tres grupos para una comida saludable!" (página 44)
- "Tamaño de las Porciones" (página 45)
- "Ejercicios de estiramiento" (página 48)

CARTELES PARA MOSTRAR:

- Cartel derivado de la hoja "Identificar alimentos saludables" (página 43)
- Cartel derivado de la hoja "¡Se necesitan los tres grupos para una comida saludable!" (página 44)
- Cartel derivado de la hoja "Tamaño de las Porciones" (página 45)
- Cartel derivado de dibujo educativo 3.1 (página 46)
- Cartel derivado de dijubjo educativo 3.2 (página 47)
- Cartel derivado de "Ejercicios de estiramiento" (página 48)

ESQUEMA DE LA SESIÓN

INTRODUCCIÓN A LA SESIÓN

1. Bienvenida
2. Repaso de la sesión de la semana anterior
3. Acerca de esta sesión

LA SESIÓN EN ACCIÓN

1. Identificar los grupos de alimentos y entender cómo actúan de maneras diferentes en el cuerpo
2. Reconocer los tamaños de las porciones y cuántas porciones deben comer de cada grupo de alimentos
3. Actividad de tamaño de la porción
4. Los beneficios de la actividad física
5. Tipos de actividad física

REPASO DE LOS PUNTOS MÁS IMPORTANTES DE HOY

TAREA SEMANAL

CIERRE DE LA SESIÓN

INTRODUCCIÓN A LA SESIÓN

1 | BIENVENIDA

- Dé la bienvenida a los participantes a la sesión.

2 | REPASO DE LA SESIÓN DE LA SEMANA ANTERIOR

NOTA : *Dé a los participantes unos 2 minutos para responder.*
HAGA : *Escriba las respuestas en el pizarrón o un papelógrafo pegado a la pared.*

DIGA:

- La semana pasada hablamos acerca de los síntomas (taq k'u't) de un ataque al corazón (rukamik k'uxaj). ¿Quién recuerda cuáles son los síntomas (taq k'u't)?

PREGUNTE:

- ¿Quién recuerda los factores de riesgo (rub'anakil rutzilal) que podemos prevenir o controlar?

AÑADA LOS SIGUIENTES SÍNTOMAS (TAQ K'U'T) SI NO LOS MENCIONAN:

- dolor o presión en el pecho
- molestia o incomodidad en el cuello o la mandíbula, uno o ambos brazos, la espalda o el estómago
- dificultad para respirar
- sudor frío
- náusea o malestar en el estómago
- sensación de mareo

PREGUNTE:

- ¿Quién recuerda qué deberían hacer si sienten estos síntomas (taq k'u't)?

RESPUESTA: Llamar a los bomberos en menos de 5 minutos, aún si no estuviera seguro de tener un ataque al corazón (rukamik k'uxaj). Al llamar a los números 122 y 123 será atendido más rápido.
- No conduzca usted mismo al hospital (aq'omab'äl jay).

3 | ACERCA DE ESTA SESIÓN

DIGA:

- En la sesión de hoy hablaremos sobre la alimentación saludable y la actividad física y lo importante que son éstos para la salud del corazón (raxnaqil k'uxaj). Cuando termine la sesión, ustedes sabrán:
  - Identificar los grupos de alimentos y entender cómo actúan de maneras diferentes en el cuerpo.
  - Escoger una variedad de alimentos para tener un cuerpo sano.
  - Reconocer los tamaños correctos de las porciones y cuántas porciones deben comer de cada grupo de alimentos.
  - Explicar las características básicas de una dieta saludable y planear comidas adecuadas.
  - Cómo ustedes y sus familias se pueden beneficiar de la actividad fisica.
  - Qué tipos de actividades físicas son buenas para ustedes y para su corazón.

LA SESIÓN EN ACCIÓN

1 | IDENTIFICAR LOS GRUPOS DE ALIMENTOS Y ENTENDER CÓMO ACTÚAN DE MANERAS DIFERENTES EN EL CUERPO

DIGA:

- Es importante comer una variedad de comidas para consumir las vitaminas (taq uchuq'ab'äl) y nutrientes (taq ilinemil) que el cuerpo necesita. Una persona debe consumir comida saludable de cuatro grupos principales de alimentos.

HAGA : *Entregue la hoja "Identificar Alimentos Saludables" y muestre el cartel. (página 43)*

GRUPO 1 | CARBOHIDRATOS (TAQ KAB'AQ'AL)

DIGA:

- El primer grupo contiene los carbohidratos (taq kab'aq'al), los cuales son comidas importantes que nos dan energía (uchuq'ab'il). Los carbohidratos (taq kab'aq'al) son todos los cereales, granos, fideos, arroces, y panes; y son elementos que tienen mucha energía (uchuq'ab'il). Si una persona come demasiados carbohidratos (taq kab'aq'al) (sin incluir otras comidas), entonces los niveles de azúcar en el cuerpo pueden subir mucho. Esto es especialmente importante para las personas con diabetes (kab'yab'il), porque deben mantener un equilibrio en la cantidad de carbohidratos (taq kab'aq'al) que comen durante el día (es decir, comer el mismo número de porciones en cada comida.) Todos los carbohidratos (taq kab'aq'al) tienen ese efecto en el cuerpo, pero los carbohidratos (taq kab'aq'al) con más fibra (uchuq'a') (como los granos integrales, arroz integral, tortillas de maíz, etc.) ayudan a que el azúcar no suba tanto como con las comidas bajas en fibra (uchuq'a') (pan blanco, pasteles, arroz blanco, etc.). Por otro lado, todos los carbohidratos (taq kab'aq'al) como los pasteles, galletas dulces, y dulces le subirán aún más los niveles de azúcar en la sangre.

DIGA:

- Éstos son algunos ejemplos de carbohidratos (taq kab'aq'al) saludables:

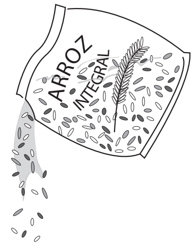

- arroz integral

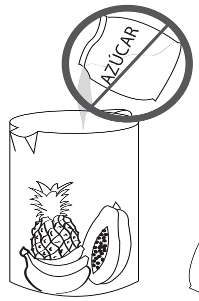

- frescos sin azúcar

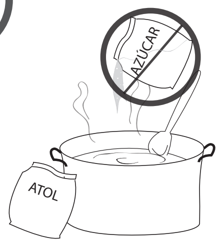

- atol sin azúcar

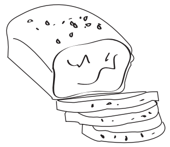

- pan integral

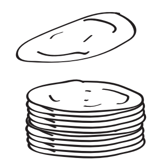

- tortillas de maíz

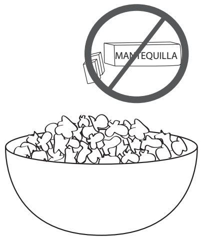

- poporopos sin mantequilla

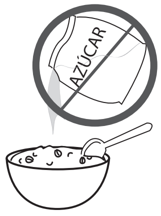

- avena en hojuela, cocinada sin azúcar

GRUPO 2 | PROTEÍNAS (TAQ RUCHUQ'A' CH'AMICH'ÄQ)

DIGA:

- El segundo grupo contiene las proteínas (taq ruchuq'a' ch'amich'äq). Las proteínas (taq ruchuq'a' ch'amich'äq) le dan al cuerpo varios nutrientes (taq ilinemil), y dan energía (uchuq'ab'il) que dura la mayor cantidad de tiempo. La mayoría de las proteínas (taq ruchuq'a' ch'amich'äq) no suben los niveles de azúcar en la sangre, por lo que ayudan a mantener un equilibrio. Sin embargo, es importante saber que proteínas (taq ruchuq'a' ch'amich'äq) como el yogur y la leche sí pueden subir los niveles de azúcar, y por eso hay que controlar cuánto comen. Las proteínas (taq ruchuq'a' ch'amich'äq) también son importantes para los músculos (taq ti'ojilal), el crecimiento (k'iyinem) y para sanar heridas.

DIGA:

- Éstos son algunos ejemplos de proteínas (taq ruchuq'a' ch'amich'äq) saludables:

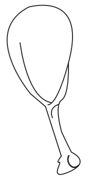

- carne y pollo sin grasa (q'anal), y sin pellejo

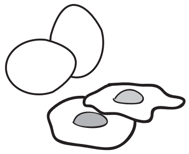

- huevos

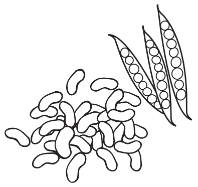

- frijoles y legumbres

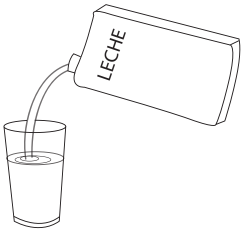

- leche

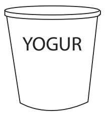

- yogur natural sin grasa y azúcar

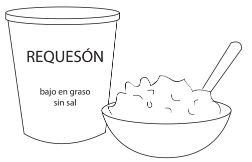

- requesón bajo en grasa (q'anal) y sin sal

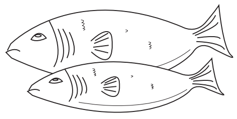

- pescado

GRUPO 3 | FRUTAS Y VERDURAS

DIGA:

- El tercer grupo contiene las frutas y verduras, que ayudan a mantener un nivel de azúcar en la sangre bajo. Es importante notar que las frutas sí suben los niveles de azúcar, pero esto no significa que alguien con no pueda comerlas, sólo debe hacerlo con moderación. La mayoría de verduras no suben el azúcar (hay algunas que son la excepción, como el maíz, arvejas, papa y camote.) Asi mismo, las frutas y verduras protejen al cuerpo contra enfermedades debido a la alta cantidad de las vitaminas (taq uchuq'ab'äl) que tienen. Otro dato importante de las frutas y verduras es que tienen, especialmente en la cáscara y las semillas pequeñas (como las fresas y moras), por lo que ayudan a mantener un nivel de azúcar en la sangre más balanceado.

GRUPO 4 | GRASAS (Q’ANAL)

DIGA:

- El último grupo contiene las grasas (q’anal), éstas le dan energía (uchuq’ab’il) al cuerpo y toman más tiempo en digerirse y absorberse, por lo que probablemente se sentirá más lleno después de comer comidas altas en grasas (q’anal). Las grasas (q’anal) son alimentos que el cuerpo necesita para sus funciones diarias, pero que deben comer en cantidades limitadas. Si una persona come demasiadas grasas (q’anal) (sin incluir otras comidas), empezará a subir de peso, y con esto puede incrementar los riesgos (taq retzelal) de enfermedades como la diabetes (kab’yab’il), presión arterial alta (jotöl ruchuq’a’ kïk’), y otros problemas del corazón. Es importante comer otras comidas como frutas, verduras, productos lácteos y carnes para recibir las vitaminas (taq uchuq’ab’äl) que el cuerpo necesita y mantener un peso equilibrado y saludable.

DIGA:

- Éstos son algunos ejemplos de grasas (q’anal) saludables:
  - aceite de oliva o vegetal
  - semillas de marañón y nueces tostadas, sin sal

HAGA : *Entregue a los participantes la hoja “Se necesitan las tres grupos para cada comida saludable” (página 44) y muestre con el cartel. Lea en voz alta.*

DIGA:

- Para crear un plato equilibrado (chojalin läq), éste debe estar dividido en tres partes, como lo demuestra el cartel, indicando los tres grupos más importantes:

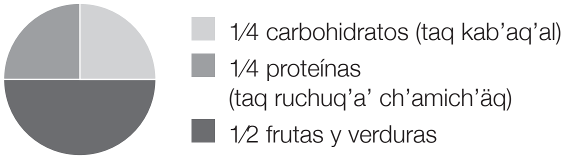

NOTA : *Todas las comidas que comemos suben el azúcar en la sangre, pero no hay que olvidar que las bebidas también pueden causar que el azúcar en la sangre suba demasiado, como las bebidas dulces. Por ejemplo: gaseosas, jugos, frescos con azúcar, café con azúcar o atol con azúcar.*

- Si se dan cuenta, las grasas (q’anal) no se incluyen como uno de los grupos principales, ya que generalmente cuando se usa aceite vegetal (ichajilal yaq’anal), o mantequilla (qasq’anal) para cocinar, se consumen las porciones adecuadas. Además, hay varias comidas que naturalmente tienen un poco de grasa (q’anal), como el huevo, la carne, y el pollo, entre otros, y no es necesario consumir grandes cantidades para cumplir con lo que el cuerpo necesita. Por eso, no se cuentan como un grupo aparte en el cartel.

2 | RECONOCER LOS TAMAÑOS CORRECTOS DE LAS PORCIONES Y CUÁNTAS PORCIONES DEBEN COMER DE CADA GRUPO DE ALIMENTOS

DIGA:

- Los alimentos que escogen para comer cada día afectan su salud. Vamos a ver algunos consejos sobre cómo escoger alimentos saludables de los diferentes grupos de alimentos.

DIGA:

- También es importante saber que la *cantidad* de comida que se come. Lo más importante es reconocer la cantidad de carbohidratos (taq kab’aq’al) y dulces que se come con cada comida, porque comer demasiado puede hacer que el nivel de azúcar en la sangre suba a niveles muy altos. Las personas que son diabéticas deben reconocer que las comidas como pan dulce, tortillas (taq wäy), jugo, y helado tienen

carbohidratos (taq kab'aq'al) y que pueden subir los niveles de azúcar demasiado. Es por esta razón que no deben consumir muchas porciones de éstas, para evitar que el azúcar suba más arriba de lo debido.

HAGA : *Entregue a los participantes la hoja "Tamaño de las Porciones" (página 45) y muestre con el cartel.*

HAGA : *Muestre algunos ejemplos de los porciones.*

CARBOHIDRATOS (TAQ KAB'AQ'AL)

DIGA:

- Comer 1-2 porciones de carbohidratos (taq kab'aq'al) por comida.
- Media Taza (4 onzas) de:
  - fideos
  - pasta
  - arroz
  - cereal cocinado (como avena o atol)
  - maiz/elote
  - papas
  - helado
  - plátano
- Media Taza (4 onzas) de:
  - frescos con azúcar
  - atol o mosh con azúcar
  - jugos
  - gaseosas
  - café con azúcar
- O coma:
  - 2-3 tortillas
  - 1 rodaja de pan o 1 pan del tamaño de su puño
  - 4 galletas pequeñas
  - 3-5 pedazos de dulces
  - 1 taza de cereal listo para comer (como Corn Flakes), el tamaño de su puño

NOTA : *No se olvide de que los bananos, plátanos, papas, arvejas y maíz contienen azúcar, como los dulces, y pueden aumentar el azúcar en la sangre. No coma dulces, pasteles, golosinas y azúcares agregados todo los días.*

DIGA:

- Los cereales integrales contienen fibra (uchuq'a') de las cáscaras de los granos, y esto ayuda al cuerpo a absorber el azúcar más lentamente. Esto signifca que el azúcar en la sangre es menos después de comer granos integrales que granos blancos. Algunos ejemplos de granos integrales son el trigo entero, los granos enteros u hojuelas de avena, las gachas de avena (mosh).

DIGA:

- Si tiene hambre, pruebe cereales de granos integrales (como avena) con poca o ninguna sal ni mantequilla.

GRASAS Y ACEITES (Q'ANAL CHUQA' YAQ'ANAL)

PREGUNTE:

- ¿Cuántos de ustedes consumen la mayoría de sus grasas (q'anal) de los alimentos como las nueces, pescado o los aceits vegetals (taq yaq'anal)?

DIGA:

- Es importante que limiten las grasas sólidas (ritzelal q'anal) como la mantequilla (qasq'anal) y la margarina en barra. También es importante limitar los alimentos que contienen estas grasas sólidas (ritzelal q'anal).
- Muestre los tamaños adecuados de porciones para la mayoría de aceites (yaq'anal) y grasas (q'anal) son:
  - 1 cucharadita de aceite vegetal (ichajilal yaq'anal)
  - 1 cucharadita de mantequilla suave
  - 1/4 taza (1-2 oz) de semillas o nueces

FRUTAS Y VERDURAS

DIGA:

- Coman más frutas y verduras.
  Traten de llenar la mitad de su plato todos los días con éstas.

FRUTAS

DIGA:

- Consuman una variedad de frutas frescas o secas en 4 – 5 porciones al día.
- Cuando se coma una fruta entera, el tamaño adecuado de la porción de ésta debe ser mediano, o igual al tamaño de su puño (por ejemplo, una manzana del tamaño de su puño). Al comer fruta cortada, la porción es de 1 taza. La porción de fruta seca es 1/4 de taza, o lo que cabe adentro de su puño.
- No consuman jugos de frutas que contienen mucho azúcar. Consuman más las frutas enteras porque la cáscara tiene más fibra (uchuq'a').
- Pongan un tazón de frutas sobre la mesa, y escoja una fruta en vez de un pastel, pan o galletas para postre.

VERDURAS

DIGA:

- Consuman una variedad de verduras, 4 – 5 porciones al día.
- Cuando coma verduras crudas, la porción adecuada es de 1 taza (como el tamaño de sus dos manos juntas en forma de "traste hondo"), y cuando es verdura cocida, el tamaño de la porción es de 1 de sus manos abiertas en forma de traste hondo.
- Coman más verduras como las zanahorias y los camotes. Elijan camote en lugar de papas. Cuando coman papa no le quiten la cáscara, porque ésta tiene fibra (uchuq'a').
- Planeen algunas de las comidas alrededor de un plato principal hecho de verduras como una sopa con frijoles, o verduras salteadas al estilo chino, con apenas un poquito de grasa (q'anal).

PROTEÍNAS (TAQ RUCHUQ'A' CH'AMICH'ÄQ)

DIGA:

- Consuman un alimento rico en proteínas (taq ruchuq'a' ch'amich'äq) en cada comida para ayudar a controlar el azúcar en la sangre. Consuman una variedad de alimentos con proteína (ruchuq'a' ch'amich'äq).
- Prueben leche de soya en lugar de leche.
- Elijan pollo y otras aves o cortes de carne de res que sean bajos en grasa (q'anal), y quítenles toda la grasa (q'anal) y pellejos visibles para limitar la cantidad de grasa saturada (ritzelal q'anal) que consumen.
- Elijan frijoles, alverjas, nueces, semillas y otras legumbres como buenas fuentes de proteínas (taq ruchuq'a' ch'amich'äq).

DIGA:

- La porción adecuada para las proteínas (taq ruchuq'a' ch'amich'äq) equivale a un pedazo del tamaño de la palma de su mano, y el grosor de su dedo meñique, de lo siguiente:
  - carne (res, cerdo)
  - pollo, pavo
  - peces
- Un cuarto de taza (1-2 oz, lo que cabe en su puño cerrado) de cada uno de los siguientes alimentos:
  - nueces
  - semillas
- Una taza de los siguientes alimentos:
  - yogur
  - leche
- O coman:
  - 1 huevo
  - 1 pedazo de queso (el tamaño de su dedo gordo, o una rodaja delgada del tamaño de su palma)
  - Media taza de frijoles

3 | ACTIVIDAD DE LOS TAMAÑOS DE LAS PORCIONES

DIGA:

- Ahora veamos lo que recuerdan acerca de los tamaños de las porciones.

HAGA : *Lea las siguientes preguntas, mostrando la taza medidora que corresponda a cada respuesta.*

PREGUNTA:

- ¿Cuánto es una porción de una verdura cocinada?

RESPUESTA: 1/2 taza

PREGUNTA:

- ¿Cuánta leche o yogur se considera una porción?

RESPUESTA: 1 taza

PREGUNTA:

· ¿Me pueden dar un ejemplo de una porción del grupo de los granos?

RESPUESTA: Ejemplos de una porción del grupo de los granos incluyen:

- una rebanada de pan
- 1 onza (alrededor de 1 taza) de cereal listo para comer, o 1/2 taza
- de cereal cocinado como la avena.
- 1/2 taza de arroz o de pasta o fideos cocinada

PREGUNTA:

· ¿A qué equivale una porción de fruta?

RESPUESTA: 1 manzana mediana, banano, o naranja, 1 taza de fruta cruda, 1/2 taza jugo de fruta (ruya'al ruwäch che') o 1/4 taza de fruta (ruya'al ruwäch che') seca

PREGUNTA:

· ¿Cuánto es una porción de frijoles cocidos?

RESPUESTA: 1/2 taza

PREGUNTE:

· ¿Qué pueden hacer para comer menos grasa (q'anal), colesterol (saqab'uyül q'anal) y sal?

AGREGUE LAS SIGUIENTES RESPUESTAS SI NO SE MENCIONAN:

- Cuando hagan las compras:
  - Elijan una variedad de granos enteros, frutas y verduras.
  - Escojan productos de leche, salsas para las ensaladas y mayonesas (taq saqatzep) que sean bajos en grasas (q'anal) o sin grasa (q'anal).
  - Elijan los cortes de carne que tengan bajo contenido de grasa (q'anal). Recorte el exceso de grasa (q'anal).
- Cuando cocinen:
  - Usen aceite vegetal (ichajilal yaq'anal) en vez de mantequilla (qasq'anal).
  - Cocinen con métodos que usan poco aceite (yaq'anal), como hornear, asar o hervir (sin añadir grasa (q'anal)) en lugar de freír.
- Cuando coman:
  - Coman menos grasas (q'anal), aceites (yaq'anal) y dulces.
  - Quítenle el pellejo al pollo y al pavo. ¡Tiren el pellejo! No se lo coman.
  - No coman más de cuatro yemas de huevo por semana.
  - Disminuyan la cantidad de alimentos altos en grasa (q'anal) pero con poco valor nutritivo, como gaseosas, pasteles, tortas, galletas dulces y saladas, tortrix, chicharrones, papas fritas y otros productos similares.

4 | LOS BENEFICIOS DE LA ACTIVIDAD FÍSICA

DIGA:

- La actividad física puede ayudarle a:
  - bajar la presión arterial (ruchuq'a' kïk')
  - contolar el colesterol (saqab'uyül q'anal) en la sangre
  - disminuir la probabilidad de desarrollar enfermedades del corazón (taq ruyab'ilal k'uxaj), diabetes (kab'yab'il) y cáncer (sachöy jotay)
  - fortalecer el corazón y los pulmones
  - formar y mantener huesos, músculos (taq ti'ojilal) y articulaciones saludables (raxnaqil laqul)
  - controlar el peso
  - tener más energía (uchuq'ab'il)
  - sentirse mejor acera de sí mismo
  - reducir el estrés (sachk'u'x)
  - disminuir los sentimientos de depresión y ansiedad
  - dormir mejor

DIGA:

- Las personas se sienten mejor cuando están activas. La actividad física y alimentación saludable les puede ayudar a perder el exceso de peso.

5 | TIPOS DE ACTIVIDAD FÍSICA

PREGUNTE:

- ¿Qué hacen ustedes para mantenerse físicamente activos?

NOTA : *Dé al grupo 2 minutos para responder.*
HAGA : *Escriba las repuestas en el pizarrón o en un papelógrafo pegado a la pared con cinta adhesiva.*

DIGA:

- Hay diferentes tipos de actividad física. Dentro de lo que es la actividad física se encuentran algunas de las cosas que ustedes probablemente hacen todos los días, como caminar, lavar la ropa, trabajar en el campo y mercado o los trabajos domésticos.

HAGA : *Muestre el dibujo educativo 3.1 con el cartel (página 46).*

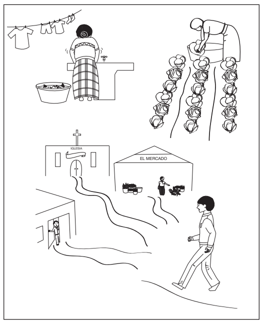

HAGA : *Muestre el dibujo educativo 3.2 con el cartel (página 47).*

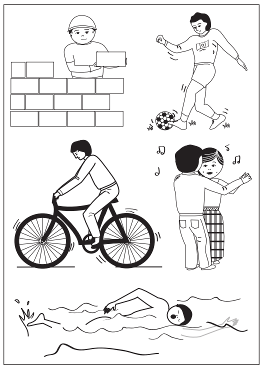

DIGA:

- Tal vez quieran comenzar con actividades moderadas (es decir, de un nivel medio). Las actividades moderadas requieren que ustedes hagan un poco de esfuerzo físico, pero les permite mantener una conversación mientras las hacen.
- Algunos ejemplos de actividades moderadas incluyen:
  - caminar a paso ligero a la iglesia, el mercado o casa de un amigo
  - trabajar en el campo (cultivar las plantas, recoger verduras o frutas)
  - realizar mantenimiento de la casa

DIGA:

- Después de un tiempo podrán pasar a hacer actividades más vigorosas (es decir, de alto nivel). Las actividades vigorosas requieren un esfuerzo físico más grande y aceleran los latidos del corazón y la respiración. Generalmente, no se puede mantener una conversación mientras se hace alguna actividad vigorosa. Las actividades vigorosas de las que ustedes pueden disfrutar incluyen:
  - jugar fútbol, baloncesto o béisbol
  - nadar varios largos de una piscina
  - andar en bicicleta rápidamente o en subida
  - hacer trabajo fuerte de construcción
  - caminar rápido, distancias más largas y/o en subida

¡SIMPLEMENTE MUÉVASE!

DIGA:

- Ahora voy a mostrarles algunos ejercicios de estiramiento. Mírenme y después traten de hacer los ejercicios ustedes. Algunos ejercicios son más fáciles que otros. Con el tiempo y la práctica, podrán hacerlos todos. Si al principio los encuentran un poco difíciles, hagan lo que puedan. Les voy a dar una hoja que les indica cómo hacer estos ejercicios de estiramiento. Utilicen la hoja para hacer los ejercicios en su casa.

HAGA : *Entregue a cada participante las hojas "Ejercicios de estiramiento" y muestre con el cartel (página 48).*

REPASO DE LOS PUNTOS MÁS IMPORTANTES DE HOY

DIGA:

- Ahora repasemos lo que aprendimos el día de hoy.

PREGUNTE:

- ¿Cuáles alimentos debemos comer con menos frecuencia?

RESPUESTA: Deberíamos limitar alimentos que tienen un alto contenido de grasa (q'anal), sal y azúcar, incluyendo:

- Alimentos con alto contenido de grasa (q'anal) como frijoles refritos, pollo frito y cortes de carne grasosos
- Alimentos salados, como las papas fritas y los nachos
- Alimentos con alto contenido de grasa (q'anal) y de azúcar como pastelería, galletas y chocolate
- Alimentos con mucha azúcar como dulces y gaseosas

PREGUNTE:

- ¿Por qué es útil saber cuántas porciones se debe comer de cada uno de los principales grupos de alimentos?

RESPUESTA: Cuando se sabe la cantidad de alimentos que se debe comer cada día, es más fácil controlar la cantidad de grasa (q'anal) que se consume.

PREGUNTE:

- ¿Cómo creen ustedes que la actividad física les puede ayudar?

RESPUESTA: La actividad física realizada regularmente puede ayudar a:

- fortalecer el corazón y los pulmones
- formar y mantener huesos, músculos (taq ti'ojilal), articulaciones saludables (raxnaqil laqul)
- controlar el peso
- tener más energía (uchuq'ab'il)
- sentirse mejor acerca de sí mismo
- reducir el estrés (sachk'u'x)
- disminuir los sentimientos de depresión y ansiedad
- dormir mejor

PREGUNTE:

- ¿Cuál es la actividad que casi todos pueden hacer?

RESPUESTA: caminar a paso ligero

TAREA SEMANAL

DIGA:

· Hoy han aprendido mucho sobre cómo mantener alimentación saludable y actividad física. Ahora pensemos cómo pueden poner en práctica lo que aprendieron. Les pido que piensen en un cambio que pueden hacer en su vida diaria. Esto será su tarea semanal.

- Sean específicos sobre qué piensan hacer, cómo piensan hacerlo y cuándo comenzarán. Algunos ejemplos incluyen:

NOTA : *Dé a los participantes 3 minutos para que piensen en una tarea.*

  · Haremos pollo al horno en vez de pollo frito para la próxima cena familiar.

  · Comenzando mañana, comeremos una verdura y una fruta (ruwäch che') más al día.

HAGA : *Escriba alguna de las ideas para las tareas en el pizarrón o en un papelógrafo pegado.*

PREGUNTE:

· ¿Quién nos quiere decir cuál es su tarea?

(OPCIONAL) DIGA:

· El mantener en mente un valor personal puede ayudarlos a hacer del comer saludable para el corazón parte de su vida familiar. Recuerden que un valor personal es una cualidad que consideren importante.

(OPCIONAL) DIGA:

· Nuestro valor para hoy es saber divertirse. Saber divertirse les ayudará a sentirse alegre por sus esfuerzos para mejorar su salud y la de su familia. Les ayudará a vencer los retos, permitiéndoles reírse cuando quieran llorar. El saber divertirse también les puede motivar a convertir sus tareas en hábitos permanentes.

NOTA : *Dé a los participantes 2 minutos para que piensen en una tarea.*

(OPCIONAL) PREGUNTE:

· ¿Cómo pueden usar el saber divertirse u otro valor, para ayudarles a cumplir con su tarea?

DIGA:

· La próxima semana hablaremos sobre cómo les fue con sus tareas. No se olviden de continuar trabajando para cumplir sus tareas de hacer más actividad física, de comer menos sal, grasa saturada (ritzelal q'anal), grasa transaturada (nima'itzelal q'anal) y colesterol (saqab'uyül q'anal) y de controlar su presión arterial alta (jotöl ruchuq'a' kïk').

CIERRE DE LA SESIÓN

(OPCIONAL) PREGUNTE:

· ¿Qué piensan de la sesión de hoy? ¿Qué creen ustedes que funcionó y qué no funcionó? ¿Qué cambiaron ustedes en sus vidas como resultado de lo que aprendieron en esta sesión?

NOTA : *Dé a los participantes un momento para ver si tienen algún comentario.*

DIGA:

· Gracias por asistir el día de hoy y completar esta sesión tan importante.

DIGA:

· En la próxima sesión hablaremos sobre presión arterial alta (jotöl ruchuq'a' kïk').

NOTA : *Reflexión para el presentador de la sesión. Piense en la sesión de hoy.*

IDENTIFICAR
ALIMENTOS SALUDABLES

GRUPO 1 | CARBOHIDRATOS (TAQ KAB'AQ'AL)

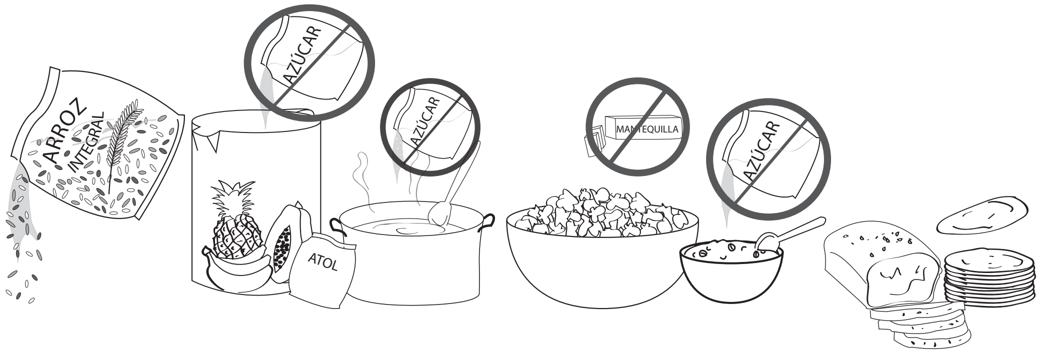

GRUPO 2 | PROTEÍNAS (TAQ RUCHUQ'A' CH'AMICH'ÄQ)

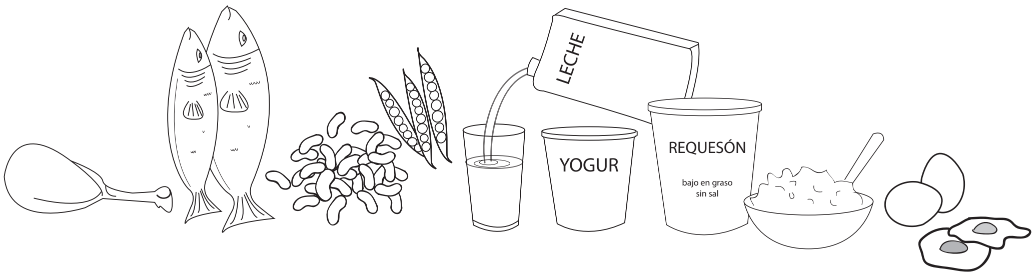

GRUPO 3 | FRUTAS Y VERDURAS

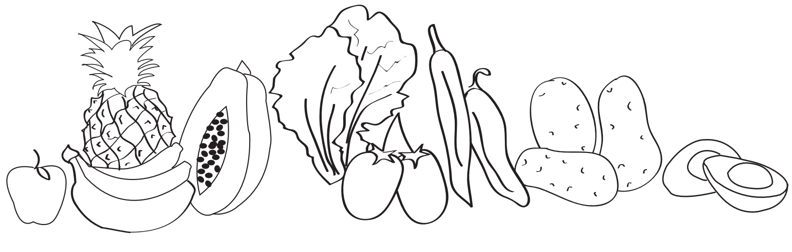

GRUPO 4 | LAS GRASAS

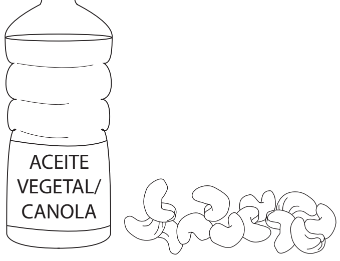

¡SE NECESITAN LAS TRES GROUPOS PARA CADA COMIDA SALUDABLE!

¡Crear un plato equilibrado!

1/4 carbohidratos 1/4 proteínas 1/2 frutas y verduras

CARBOHIDRATOS

Estos son alimentos que traen mucha energía pero hacen que el nivel de azúcar de la sangre suba muy rápido. Si se comen muchos de estos alimentos a la vez, puede hacer que el nivel de azucar suba demasiado.

PROTEÍNA

Los alimentos con proteína traen muchos [nutrientes] y dan mucha energía. Ayudan a mantener estable el nivel de azúcar en la sangre.

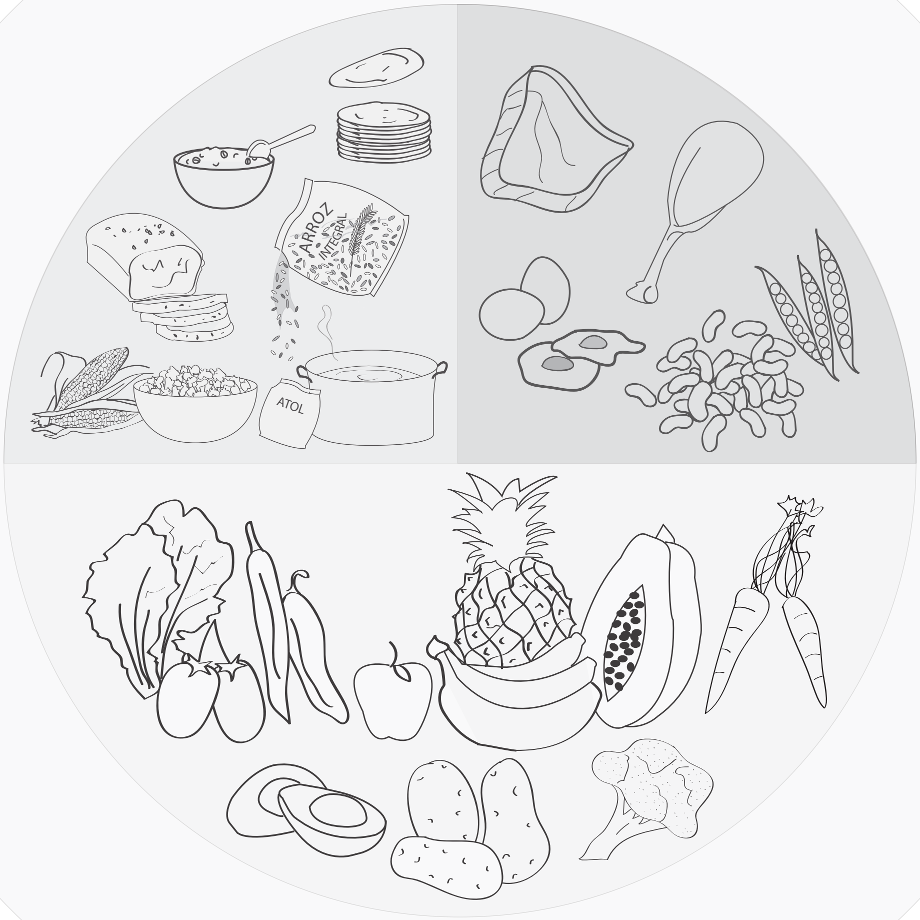

ALIMENTOS QUE PROTEGEN: FRUTAS Y VERDURAS

Estos son alimentos como frutas y verduras que ayudan a mantener bajo el nivel de azúcar en la sangre. Protegen el cuerpo contra la enfermedad porque traen muchas vitaminas y nutrientes.

No hay que olvidar que lo que estan bebiendo puede elevar el azúcar en sangre demasiado alta, como dulces.

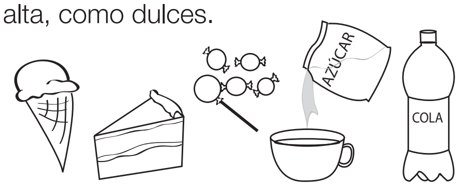

Gaseosa, jugo y fresco, café o atol (con azúcar) cuentan como carbohidratos.

TAMAÑO DE LAS PORCIONES

NOTA : *El puño es más o menos el tamaño de una taza.*

CARBOHIDRATOS

Comer 1-2 porciones de carbohidratos por comida.

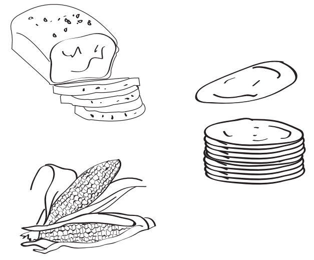

- 2-3 tortillas taq wäy
- pan (el tamaño de su puño)
- 4 galletas pequeñas
- 3-5 pedazos de dulces
- 1 taza de cereal listo para comer

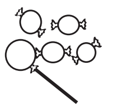

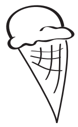

MEDIA TAZA:

- fideos
- pasta
- arroz
- cereale cocinado
- maiz/elote
- papas
- helado
- plátano/banano

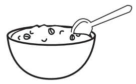

FRUTAS Y VERDURAS

Lo mejor es comer frutas y verduras enteros. Las frutas y verduras (taq ruwäch che' chuqa' ruwäch ichaj) representan la mitad de cada comida. Coma muchas frutas y verduras como es posible.

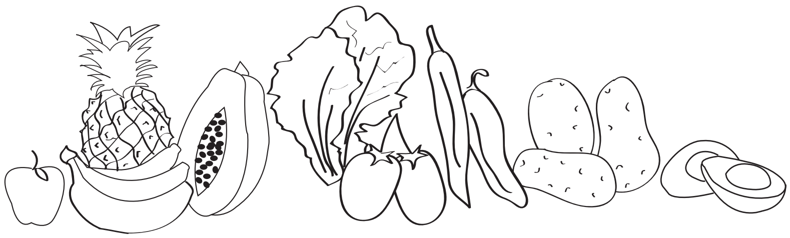

PROTEÍNAS

Tienen un alimento rico en proteínas en cada comida para ayudar a controlar el azúcar en la sangre.

- 1 huevo
- 1 pedazo de queso (el tamaño de 1/2 su puño)
- media taza de frijoles

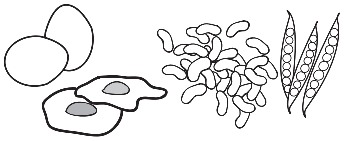

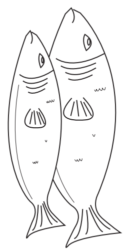

UN PEDAZO DEL TAMANO DE LA PALMA DE SU MANO:

- came (res, cerdo)
- pollo, pavo
- peces

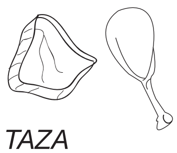

UN QUARTO DE TAZA DE CADA UNO DE LOS SIGUIENTES ALIMENTOS:

- nueces
- semillas

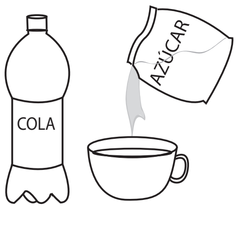

MEDIA TAZA:

- frescos con azúcar
- atol o mosh con azúcar
- jugos
- gaseosa
- café con azúcar

3.1

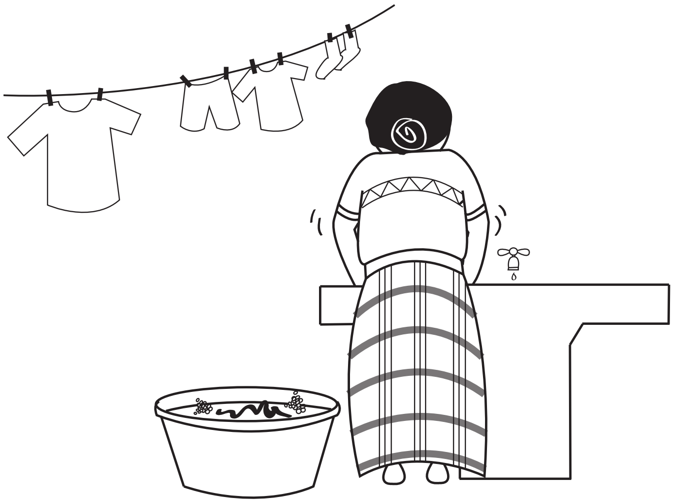

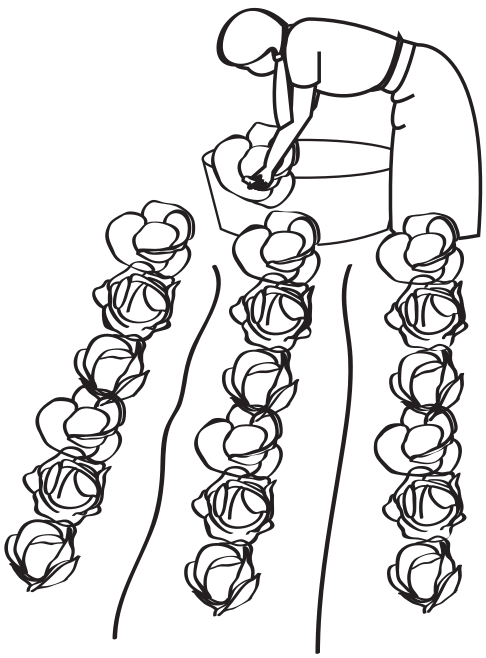

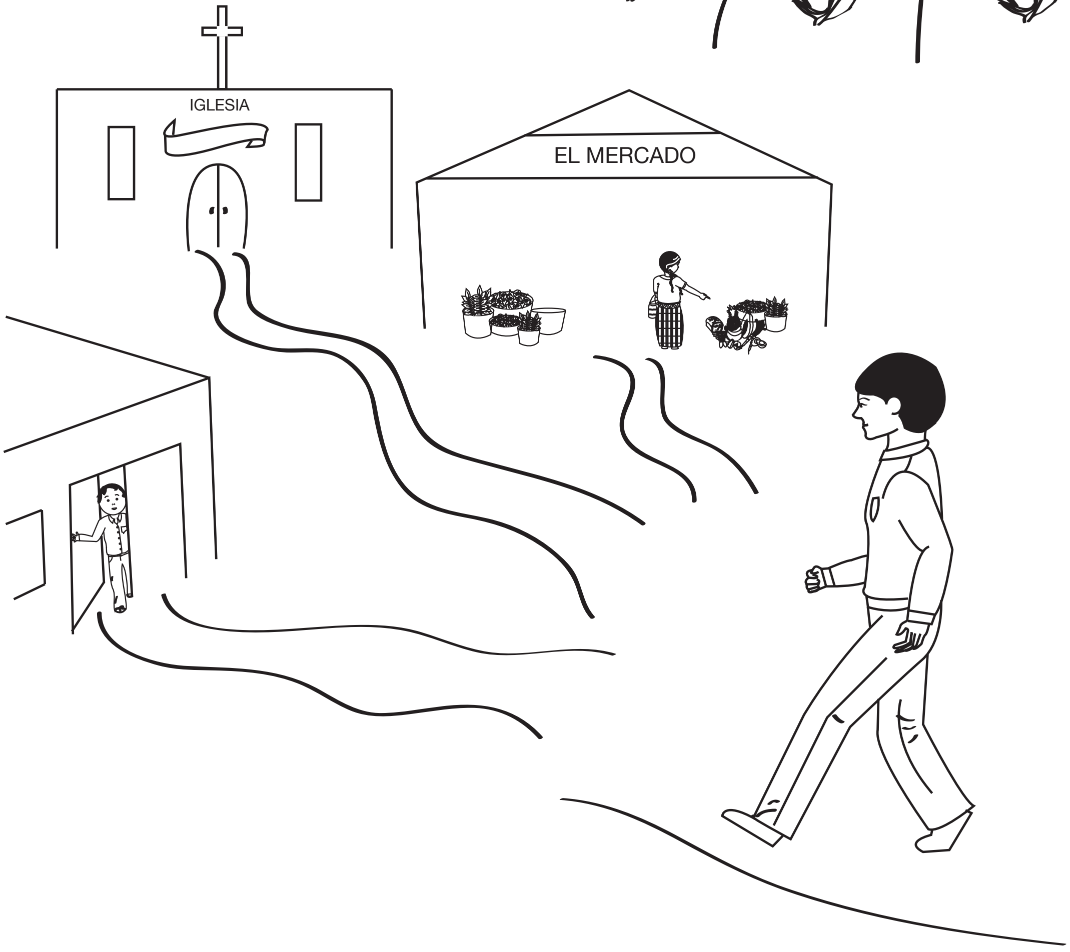

3.2

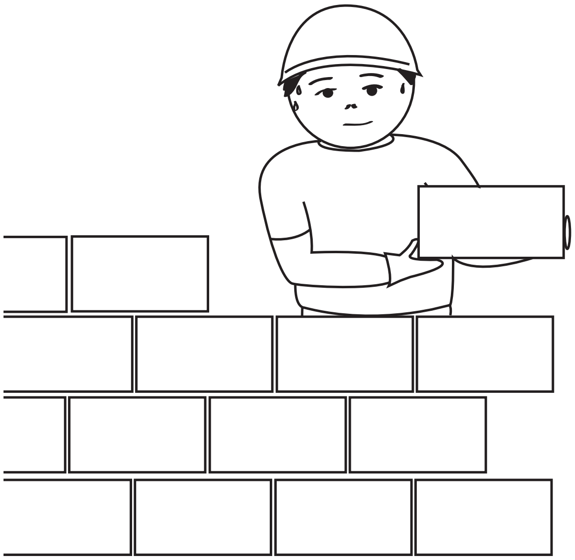

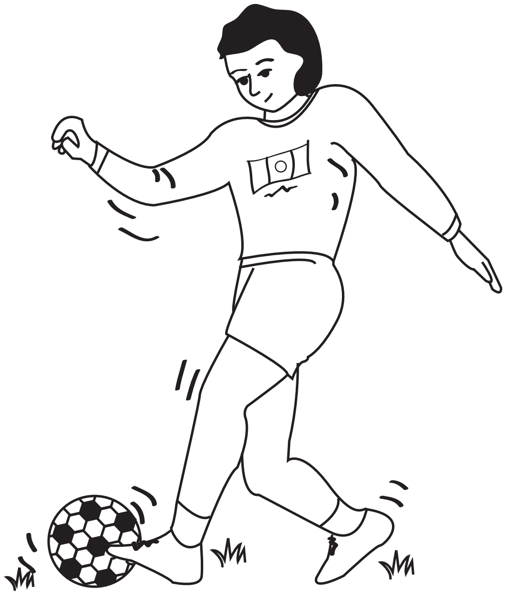

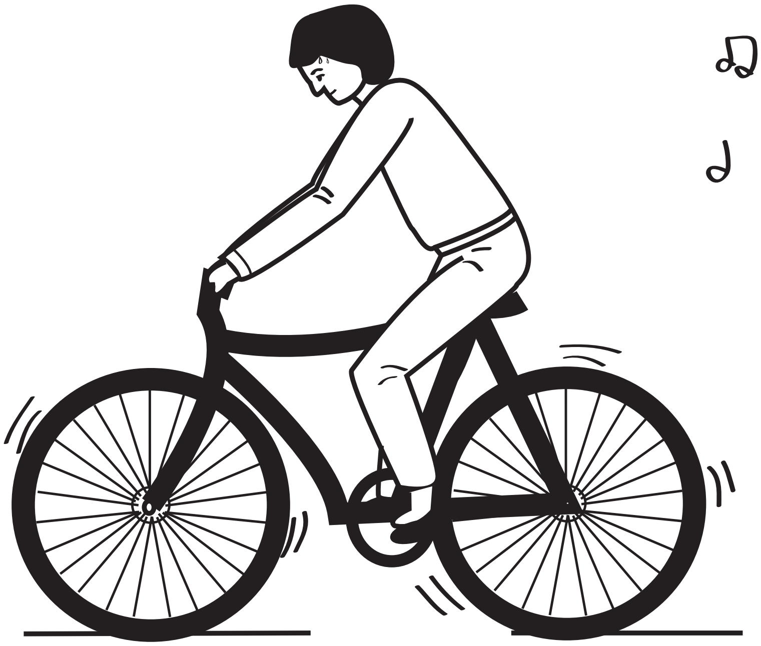

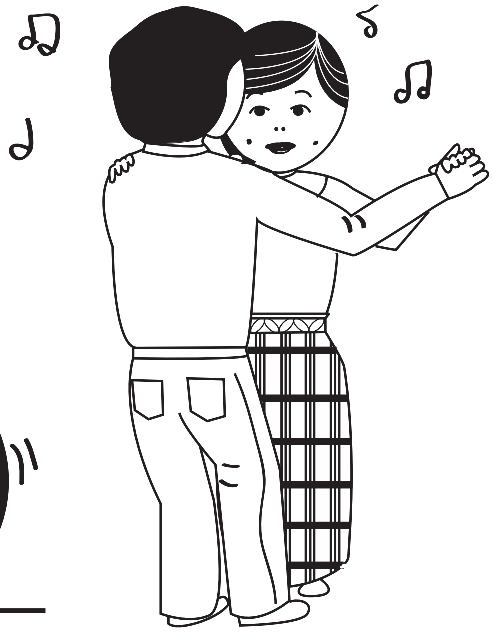

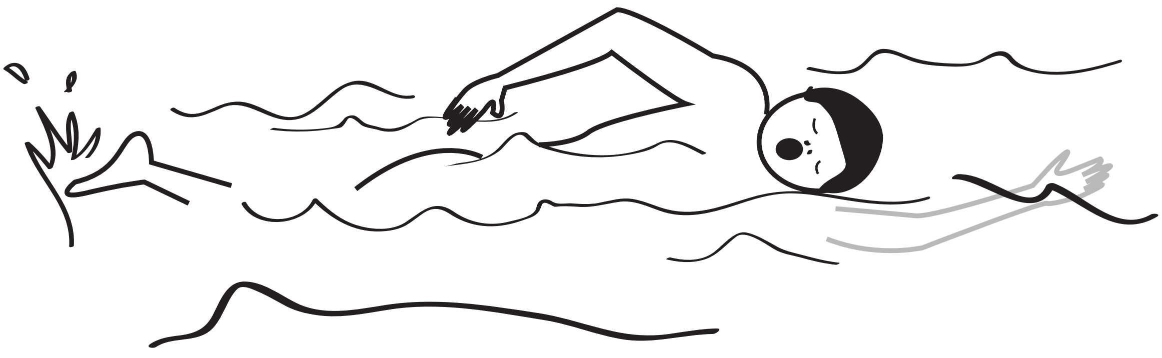

EJERCICIOS DE ESTIRAMIENTO

Haga estos ejercicios de estiramiento suavemente y en forma lenta, sin saltar.

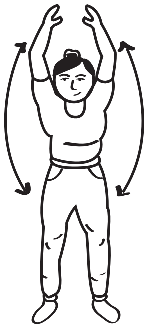

1. RESPIRACIÓN PROFUNDA
Levante los brazos, respire, baje los brazos, exhale. Todo esto repitalo dos veces.

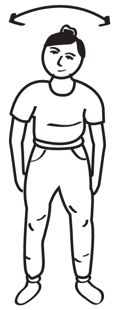

2. ESTIRAMIENTO DEL CUELLO
Mueva la cabeza despacito de un lado al otro 2 veces en cada dirección.

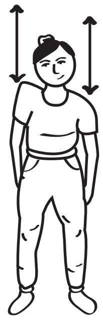

3. ESTIRAMIENTO DE LOS HOMBROS
Mueva los hombros hacia arriba y hacia abajo 5 veces en cada lado.

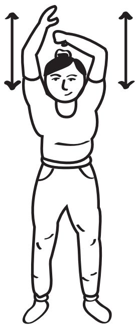

4. ESTIRAMIENTO DE LOS COSTADOS
Mueva los costado hacia arriba y hacia abajo 5 veces en cada dirección.

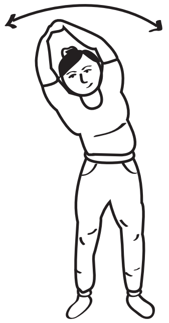

5. ESTIRAMIENTO DE LA CINTURA
Estirese de lado a lado 3 veces en cada dirección.

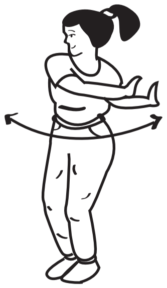

6. TORSIÓN
Tuerza la cintura de lado a lado 3 veces en cada dirección.

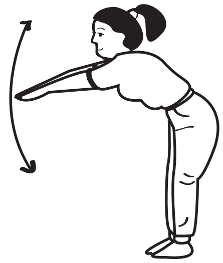

7. ESTIRAMIENTO
De abajo hacia arriba 5 veces.

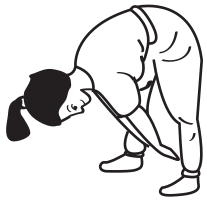

8. ESTIRAMIENTO DE CINTURA, ESPALDA Y PIERNAS
Pase los brazos entre las piernas 6 veces.

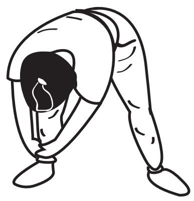

9. ESTIRAMIENTO DE LAS PIERNAS (1)
Agárrese el tobillo y sostengalo unos segundos 4 veces cada lado.

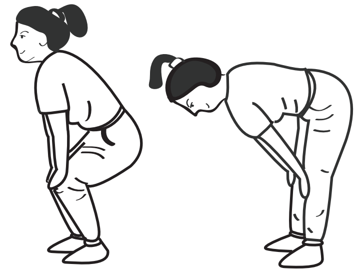

10. ESTIRAMIENTO DE LAS PIERNAS (2)
De abajo hacia arriba 5 veces.

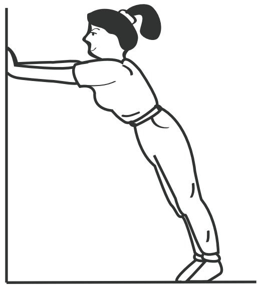

9. ESTIRAMIENTO DE LAS PIERNAS (3)
Mueva los talones hacia arriba y hacia abajo 6 veces.

SESIÓN 4

Ayude a su corazón: Controle su presión arterial alta (jotöl ruchuq'a' kïk')

OBJETIVOS

AL FINALIZAR ESTA SESIÓN, LOS PARTICIPANTES CONOCERÁN:

- Lo que es presión arterial (ruchuq'a' kïk')
- Que lo mejor es tener una presión arterial (ruchuq'a' kïk') menor de 120/80 mm Hg
- Que la presión arterial (ruchuq'a' kïk') entre 120/80 y 139/89 mm Hg se llama prehipertensión arterial (uchuq'anem)
- Que una presión arterial (ruchuq'a' kïk') de 140/90 mm Hg o más se considera alta
- Lo que es un derrame cerebral (rukamik tzatzq'or) y cuáles son sus [síntomas]
- Que si comen menos sal pueden disminuir riesgo (retzelal) de desarrollar presión arterial alta (jotöl ruchuq'a' kïk')
- Que hay cosas que se pueden hacer para disminuir la cantidad de sal en la alimentación

MATERIALES

PARA DIRIGIR ESTA SESIÓN USTED NECESITARÁ:

- Pizarrón, marcadores, almohadilla, varias hojas grandes de papel y cinta adhesiva
- Cucharas para medir (1 cucharadita, 1⁄4 de cucharadita)
- Una pequeña cantidad de sal
- Dos platos plásticos de colores

(OPCIONAL) NOTA : *Haga arreglos para que un profesional de la salud (un médico de Wuqu' Kawoq) vaya a la sesión a tomar la presión arterial (ruchuq'a' kïk') de los participantes.*

HOJAS PARA REPARTIR:

DURANTE LA SESIÓN, ENTREGUE A LOS PARTICIPANTES ESTA HOJAS:

- Lista de lugares donde los participantes pueden medirse presión arterial (ruchuq'a' kïk')

NOTA : *Prepare esta lista antes de la sesión. Puede encontrar información en un hospital, clínica o centro de salud local.*

- "Hábitos saludables contra la presión arterial alta" (página 63)
- "Conozca los síntomas de un derrame cerebral. Actúe rápidamente" (página 64)
- "Use hierbas e especies en lugar de sal" (página 66)

CARTELES PARA MOSTRAR:

- Cartel derivado de dibujo educativo 4.1 (página 62)
- Cartel derivado de "Hábitos saludables contra la presión arterial alta" (página 63)
- Cartel derivado de "Empiece hoy mismo a hacer estos cambios" (página 65)
- Cartel derivado de "Use hierbas e especies en lugar de sal" (página 66)

ESQUEMA DE LA SESIÓN

INTRODUCCIÓN A LA SESIÓN

1. Bienvenida
2. Repaso de la sesión de la semana anterior
3. Acerca de esta sesión

LA SESIÓN EN ACCIÓN

1. Los hechos importantes
2. Información sobre la presión arterial (ruchuq'a' kïk') y derrame cerebral (rukamik tzatzq'or)
3. Cómo prevenir la presión arterial alta (jotöl ruchuq'a' kïk')
4. Sal: ¿Cuánto necesitamos?
5. Reduzca sal
6. Cuidado con las bebidas alcohólicas
7. Controle su presión arterial alta (jotöl ruchuq'a' kïk') con medicamentos

REPASO DE LOS PUNTOS MÁS IMPORTANTES DE HOY

TAREA SEMANAL

CIERRE DE LA SESIÓN

INTRODUCCIÓN A LA SESIÓN

1 | BIENVENIDA

- Dé la bienvenida a los participantes a la sesión.

2 | REPASO DE LA SESIÓN DE LA SEMANA ANTERIOR

DIGA:

· La semana pasada hablamos acerca de por qué ustedes tienen que mantenerse a la alimentación saludable y la actividad física.

PREGUNTE:

· ¿Cómo les va con su tarea de incluir en sus comidas una variedad de alimentos saludables para el corazón? ¿En qué les fue bien? ¿Qué problema encontraron (como el rechazo a los cambios o falta de interés de la familia)? ¿Cómo lo resolvieron?

PREGUNTE:

· ¿Cuáles alimentos debemos comer con menos frecuencia?

RESPUESTA: Deberíamos limitar alimentos que tienen un alto contenido de grasa (q'anal), sal y azúcar, incluyendo:

· Alimentos con alto contenido de grasa (q'anal) como frijoles refritos, pollo frito y cortes de carne grasosos
· Alimentos salados, como las papas fritas y los nachos
· Alimentos con alto contenido de grasa (q'anal) y de azúcar como pastelería, galletas y chocolate
· Alimentos con mucha azúcar como dulces y bebidas gaseosas

PREGUNTE:

· ¿Por qué es útil saber cuántas porciones se debe comer de cada uno de los principales grupos de alimentos?

RESPUESTA: Cuando se sabe la cantidad de alimentos que se debe comer cada día, es más fácil controlar la cantidad de grasa (q'anal) que se consume.

NOTA : *Dé a los participantes 3 minutos para responder.*
HAGA : *Escriba las respuestas en el pizarrón o en un papelógrafo pegado a la pared.*

PREGUNTE:

· ¿Qué recuerdan sobre los beneficios de hacer más actividad física?

AÑADA LOS SIGUIENTES BENEFICIOS SI NO LOS MENCIONAN:

· La actividad física ayuda a:

· fortalecer el corazón y los pulmones
· formar y mantener saludable a los huesos, músculos (ti'ojilal) y articulaciones (laqul)
· sentirse mejor con usted mismo
· controlar el peso
· bajar la presión arterial alta (jotöl ruchuq'a' kïk')
· controlar el colesterol (saqab'uyül q'anal) en la sangre
· dormir mejor
· reducir el estrés (sachk'u'x) y los sentimientos de depresión y ansiedad
· tener más energía (uchuq'ab'il)
· disminuir la probabilidad de desarrollar diabetes (kab'yab'il), azúcar alta en la sangre, enfermedades del corazón (taq ruyab'ilal k'uxaj) y cáncer (sachöy jotay)

NOTA : *Comparta con el grupo lo que usted hizo y cualquier problema que haya tenido que enfrentar.*

DIGA:

· Al terminar la última sesión ustedes hicieron la tarea de ser más activos.

NOTA : *Dé al grupo unos 2 minutos para responder.*

PREGUNTE:

· ¿Quién de ustedes quisiera compartir con el grupo lo que hizo? ¿Qué problemas encontraron? (con relación a no tener tiempo o no tener apoyo de la familia) ¿Cómo los resolvieron?

3 | ACERCA DE ESTA SESIÓN

DIGA:

· La sesión de hoy es sobre la presión arterial (ruchuq'a' kïk') y las medidas que podemos tomar para bajar la presión arterial alta (jotöl ruchuq'a' kïk') o para prevenir que suba.

LA SESIÓN EN ACCIÓN

1 | LOS HECHOS IMPORTANTES

DIGA:

· En Guatemala, hay muchas personas que tienen presión arterial alta (jotöl ruchuq'a' kïk'), también llamada hipertensión (uchuq'anem). Casi un tercio de ellos no saben que la tienen alta.

· Otros tienen lo que se llama prehipertensión (uchuq'anem). Esto significa que están en riesgo (retzelal) de desarrollar presión arterial alta (jotöl ruchuq'a' kïk').

HAGA : *Muestre el dibujo educativo 4.1 con el cartel (página 62).*

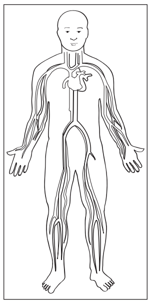

2 | INFORMACIÓN SOBRE LA PRESIÓN ARTERIAL (RUCHUQ'A' KÏK') Y EL ATAQUE AL CEREBRO (RUKAMIK TZATZQ'OR)

DIGA:

· La presión arterial (ruchuq'a' kïk') es la fuerza que ejerce la sangre contra las paredes de las arterias (kaqib'och'ilal). La presión arterial (ruchuq'a' kïk') se necesita para que circule la sangre por todo el cuerpo.

· Es importante que sepan y que recuerden cuál es su presión arterial (ruchuq'a' kïk'). Anoten sus números cada vez que se midan presión arterial (ruchuq'a' kïk').

NOTA : *Dé al grupo unos 2 minutos para responder.*

PREGUNTE:

· ¿Ustedes saben cuál es su presión arterial (ruchuq'a' kïk')?

HAGA : *Copie este cuadro en el pizarrón o en un pedazo de papelógrafo pegado a la pared.*

NOTA PARA EL PROMOTOR : *La lectura de la presión arterial consiste de dos números. La presión sistólica (cuando el corazón late) sobre la presión diastólica (cuando el corazón se relaja entre latidos). Se escribe la medida como un númo encima del otro, con el número sistólico arriba y el número diastólico abajo. Por ejemplo, una lectura de presión de sangre de 120/80mm (milimetros de mercurio) Hg, se lee como "120 sobre 80".*

LOS NIVELES DE PRESIÓN ARTERIAL (RUCHUQ'A' KÏK'):

Nivel	Sistólica (mm Hg)	Diastólica (mm Hg)	Resultados
**presión arterial normal (choj ruchuq'a' kïk')**	Menor de 120	Menor de 80	▪ ¡Bien hecho!
**prehipertensión (uchuq'anem)**	120-139	80-89	▪ Vigile su presión arterial (ruchuq'a' kïk').<br>▪ Es hora de hacer cambios en su alimentación y el nivel de actividad física que hace. Visite el centro de salud si tiene diabetes (kab'yab'il).
**presión arterial alta (jotöl ruchuq'a' kïk')**	140 o más	90 o más	▪ Consulte con su médico o enfermera en el centro de salud (aq'omab'äl jay) sobre cómo controlarla.

DIGA:

- Una presión arterial normal (choj ruchuq'a' kïk') es menor de 120/80 mm Hg. Una presión arterial (ruchuq'a' kïk') entre 120/80 y 139/89 mm Hg se considera prehipertensión (uchuq'anem). Esto quiere decir que aún no se tiene la presión arterial alta (jotöl ruchuq'a' kïk') pero que es probable desarrollarla en el futuro a menos que se hagan cambios en los hábitos de salud (rub'anelal raxnaqil). La presión arterial (ruchuq'a' kïk') está alta cuando llega o sobrepasa los 140/90 mm Hg.
- Cuando se tiene la presión arterial alta (jotöl ruchuq'a' kïk'), el corazón tiene que bombear más fuerte que lo necesario, para llevar la sangre a todas las partes del cuerpo. La presión arterial alta (jotöl ruchuq'a' kïk') aumenta las probabilidades de tener un ataque al corazón (rukamik k'uxaj) o derrame cerebral (rukamik tzatzq'or), problemas de los riñones y ceguera.
- A la presión arterial alta (jotöl ruchuq'a' kïk') se le conoce como la "asesina silenciosa (meq'ajan kamisanel)" porque generalmente no presenta síntomas (taq k'u't). La mayoría de personas que la tienen no se sienten enfermas hasta que les da un ataque al corazón (rukamik k'uxaj) o un ataque al cerebro (rukamik tzatzq'or) o cualquier otro problema causado por la presión arterial alta (jotöl ruchuq'a' kïk').

DERRAME CEREBRAL (RUKAMIK TZATZQ'OR)

DIGA:

- La presión arterial (ruchuq'a' kïk') es uno de los principales factores de riesgo (rub'anikil rutzilal) para un derrame cerebral (rukamik tzatzq'or). Hay otros factores de riesgo (rub'anikil rutzilal) que aumentan las probabilidades de tener un derrame cerebral (rukamik tzatzq'or), tales como las enfermedades del corazón (taq ruyab'ilal k'uxaj), el hábito de fumar, la diabetes (kab'yab'il) y un nivel alto de colesterol (jotöl ruchuq'a' ri saqab'uyül q'anal) en la sangre.

NOTA : *Dé a los participantes unos 2 minutos para responder.*

HAGA : *Dé a cada uno de los participantes la hoja "Conozca los síntomas de un derrame cerebral. Actúe rápidamente" (página 64).*

PREGUNTE:

· ¿Conocen a alguien que haya tenido un derrame cerebral (rukamik tzatzq'or)?

DIGA:

· El derrame cerebral (rukamik tzatzq'or) también se conoce como ataque al cerebro, apoplejía, embolia cerebral o accidente cerebrovascular. Ocurre cuando la sangre repentinamente deja de llegar al cerebro. El derrame cerebral (rukamik tzatzq'or) es un problema muy grave y puede resultar en discapacidad o la muerte.

DIGA:

· Los síntomas de un derrame cerebral (rukamik tzatzq'or) ocurren repentinamente. Se puede tener un solo síntoma (k'u't) o varios de ellos. Estos son:
  · falta de sensación en la cara, brazo o pierna (especialmente en un solo lado del cuerpo)
  · confusión, dificultad para hablar y para entender a los demás
  · problemas para ver con uno o ambos ojos
  · dificultad para caminar, mareo, pérdida del equilibrio (sachinäq chojalil) o de coordinación dolor de cabeza fuerte y repentino

DIGA:

· Los mini derrame cerebrales (taq ch'uti rukamik tzatzq'or) tienen algunos de los mismos síntomas que un derrame cerebral (rukamik tzatzq'or) pero duran menos y generalmente no causan daño al cerebro. Un mini derrame cerebrales (taq ch'uti rukamik tzatzq'or) es un síntoma (k'u't) de que un derrame cerebral (rukamik tzatzq'or) puede ocurrir en el futuro.

· Los mini derrame cerebrales (taq ch'uti rukamik tzatzq'or) son síntomas (taq k'u't) de un ataque al cerebro (rukamik tzatzq'or) que pueden durar unos pocos segundos o el día entero y después desaparecer. Usted debe actuar inmediatamente si usted o alguien que conoce tiene las señales de un mini derrame cerebral (ch'uti rukamik tzatzq'or). Llame a los números 122 y 123 de los bomberos (taq chupüy q'aq') enseguida y así ayudará a evitar problemas graves.

· Medirse la presión arterial (ruchuq'a' kïk') es fácil y no causa dolor. Medírsela por lo menos una vez al año es la mejor manera de averiguar si la tienen alta. Si ya saben que tienen la presión arterial alta (jotöl ruchuq'a' kïk'), deben medírsela más a menudo.

DÉ A CADA UNO DE LOS PARTICIPANTES:

- Una lista de lugares (como centros de salud y hospitales (taq aq'omab'äl jay) donde pueden medirse la presión arterial (ruchuq'a' kïk').

HAGA : *Pida a los participantes que levanten la mano si alguien en su familia tiene la presión arterial alta (jotöl ruchuq'a' kïk')*

3 | CÓMO PREVENIR LA PRESIÓN ARTERIAL ALTA (JOTÖL RUCHUQ'A' KÏK')

DIGA:

· Si alguien en su familia tiene la presión arterial alta (jotöl ruchuq'a' kïk'), ustedes tienen mayor riesgo (nimak'ayew) de tenerla. Aunque no la tengan alta ahora, como alguien en la familia la tiene, ustedes corren un riesgo mayor (nimak'ayew) de desarrollarla.

HAGA : *Entregue la hoja y muestre el cartel "Hábitos saludables contra la presión arterial alta" (página 63) y lea en voz alta las medidas para bajar o evitar que suba la presión arterial (ruchuq'a' kïk').*

· Lo bueno es que ustedes pueden tomar medidas para bajar la presión arterial alta (jotöl ruchuq'a' kïk') o evitar que suba. Veamos qué pueden hacer.

NOTA : *Dé a los participantes unos 3 minutos para responder.*

PREGUNTE:

· ¿Qué medidas pueden tomar ustedes o sus familias para prevenir o bajar la presión arterial alta (jotöl ruchuq'a' kïk')?

NOTA : *Para esta actividad necesitará un poco de sal y cucharas para medir los siguientes tamaños: 1/4 de cucharadita y una cucharadita. Use los platos plásticos de colores para poner la sal (atz'am) que mide y mostrar.*

4 | SAL: ¿CUÁNTO NECESITAMOS?

DIGA:

· Si ustedes comen menos sal, pueden ayudar a prevenir o bajar la presión arterial alta (jotöl ruchuq'a' kïk'). La sal forma parte de las mezclas que se usan para dar sabor a los alimentos y para conservarlos. Algunos ejemplos de estos son el consomé (no importa si es de pollo o res), la sal de ajo o cebolla, saborín, ablandadores de carne y salsa soya.

HAGA : *Muestre ahora 1/4 de cucharadita de sal (atz'am).*

· Veamos ahora cuánto sal realmente necesitamos.

· La mayoría de las personas comen mucho más de un cuarto de cucharadita por día.

· Traten de reducir la cantidad de sal proveniente de todos los alimentos y bebidas a menos de aproximadamente 1 cucharadita de sal.

HAGA : *Muestre ahora 1 cucharadita de sal (atz'am).*

· La mayoría de la gente come aproximadamente 2 1/2 cucharaditas de sal diarias.

HAGA : *Muestre ahora 2 1/2 cucharaditas de sal (atz'am).*

· Esta cantidad es 8 a 12 veces más de sal de lo que el cuerpo necesita. Esta costumbre de comer tanta cantidad de sal puede llevar a desarrollar presión arterial alta (jotöl ruchuq'a' kïk').

DIGA:

· Ahora hablemos sobre los alimentos que tienen un alto contenido de sal.

  · La sal que comemos viene de los alimentos empaquetados ("comida chatarra") . Por ejemplo, las verduras y sopas enlatadas, así mismo las plataninas, tostadas, dobladas, pollo frito, papas fritas, papalinas saladas, nachos (tortillitas, tortrix, ricitos o Doritos) y las carnes con alto contenido de sal como las salchichas, jamón y tocino.

  · Cuando comen fuera de su casa, la mayoría de los alimentos tienen un contenido alto de sal. Si tienen ustedes la presión alta (jotöl ruchuq'a' kïk'), traten de no comer afuera muy a menudo.

INFORMACIÓN ADICIONAL PARA EL PROMOTOR DE SALUD (TZIJÖY AQ'OMAB'ÄL), EL POTASIO (CHAJIL), EL CALCIO (CHUN) Y EL MAGNESIO (JIK'CHUN)

Las investigaciones recientes han demostrado que los alimentos ricos en potasio son importantes para protegerse contra la presión arterial alta (jotöl ruchuq'a' kïk'). Los alimentos ricos en calcio (chun) y en magnesio (jik'chun) también pueden ayudar. Coman alimentos ricos en estos nutrientes.

- Potasio (chajil): se encuentra en bananos, plátanos, tomates, papas, naranjas, ciruelas pasas, espinaca y frijoles.
- Calcio (chun): se encuentra en el queso, la leche, y el yogur (escoja los que tienen poca grasa (q'anal) o semidescremados); el jugo de naranja enriquecido con calcio; verduras (taq ruwäch ichaj) de hojas verdes; y pescado fresco o enlatado, previamente pasados por agua.
- Magnesio (jik'chun): se encuentra en el pan y los cereales de granos integrales, los frijoles, nueces y semillas, acelga, espinaca y hierva mora.

INFORMACIÓN ADICIONAL PARA EL PROMOTOR DE SALUD (TZIJÖY AQ'OMAB'ÄL) EL PLAN ALIMENTARIO PARA BAJAR LA PRESIÓN ARTERIAL (RUCHUQ'A' KÏK')

El plan de alimentación saludable contra la hipertensión (uchuq'anem) es conocido como el plan alimentario. Para ayudarle a bajar la presión arterial (ruchuq'a' kïk') este plan recomienda consumir:

- Muchas frutas y verduras
- Productos de leche sin grasa (q'anal) (descremados) o con un bajo contenido de grasa (q'anal) (semidescremados)
- Productos de granos integrales (tunuchuq'a' awäs)
- Pescado, pollo y carne con poca grasa (q'anal)
- Nueces, semillas y frijoles
- El plan alimentario también incluye:
- Menos sal
- Cantidades pequeñas de grasas (q'anal), aceites (yaq'anal) y dulces
- Cantidades pequeñas de dulces y bebidas con alto contenido de azúcar.

Seguir el plan alimentario y comer alimentos bajos en sal es beneficioso para las personas que tienen la presión arterial alta (jotöl ruchuq'a' kïk'). También es bueno para las personas que no tienen la presión arterial alta (jotöl ruchuq'a' kïk'), pero que desean mantener su corazón sano (raxnaqil k'uxaj).

DIGA:

· Ahora haremos una actividad que nos ayudaría a elegir alimentos con bajo contenido de sal. Primero voy a describir una situación en la que pueden encontrarse ustedes o algún pariente suyo. Luego vamos a aprender a elegir alimentos con bajo contenido de sal.

ELIJA LOS MEJORES ALIMENTOS PARA XWAN

· Xwan tenía la presión arterial (ruchuq'a' kïk') levemente alta la última vez que visitó al médico. El médico le dijo que disminuyera la cantidad de sal que comía. Ayude a Ixchel, la esposa de Xwan, a elegir alimentos que le ayuden a Xwan a seguir el consejo del médico. Voy a hacer algunas preguntas y ustedes escojan el alimento con el contenido más bajo de sal.

HAGA : *Haga las siguientes preguntas. Después de que los participantes hayan indicado sus repuestas, delas repuestas correctas.*

PREGUNTE:

· ¿Debería Ixchel escoger naranja con pepitas molidas de ayote (chilacayote) o naranja sin sal ni pepita molida?

RESPUESTA: naranja sin sal ni pepita molida. Las pepitas molidas vienen preparados con más sal.

PREGUNTE:

· ¿Debería preparar frijoles de la casa o enlatados?

RESPUESTA: frijoles de la casa. Los frijoles enlatados vienen preparados con más grasa (q'anal). Es mejor escoger los frijoles de la casa.

PREGUNTE:

· Si quiere consumir menos sal, ¿es mejor comer plataninas ó rebanadas de mango sin sal ni pepita molida?

RESPUESTA: rebanadas de mango sin sal ni pepita molida.

PREGUNTE:

· Cuando va a sazonar la comida, ¿Debería Ixchel usar consomé y sasonadores o hierbas e especies naturales?

RESPUESTA: hierbas e especies. El consomé y sasonadores tienen mucha sal agregada.

NOTA : *Ver página 66 "Use hierbas e especies en lugar de sal."*

HAGA : *Muestre el cartel "Empiece hoy mismo a hacer estos cambios" (página 64) y lea en voz alta las medidas.*

5 | REDUZCA LA SAL

DIGA:

· Revisemos ahora algunas sugerencias prácticas que les ayudarán a comer menos sal.

PREGUNTE:

· ¿Por qué les resulta difícil comer menos sal?

HAGA : *Escriba las respuestas que le den en un pizarrón o un papelógrafo pegado a la pared. Luego añada algunas soluciones posibles. Vea en el cuadro siguiente algunos problemas y sus soluciones.*

EJEMPLOS

PROBLEMAS QUE PUEDEN ENCONTRAR	*SOLUCIONES*
La comida no tiene nada de sabor.	Use especies o hierbas para agregar sabor a los alimentos. (Vea la hoja "Use hierbas e especies en lugar de sal" en la página 66).
La familia se va a disgustar.	Reduzca poco a poco la sal. Use cada vez menos sal cuando cocine, de modo que su familia se vaya acostumbrando al sabor.
Es difícil quitarse el hábito de agregar sal.	Reduzca lentamente la cantidad de sal. Con el tiempo se acostumbrará a usar menos sal. Elija marcas de alimentos que tengan menos sal. Quite el salero de la mesa.
Es difícil preparar la comida separado.	Hable con su familia sobre la importancia de esto. Estas sesiones son preparación para eso.

6 | CUIDADO CON LAS BEBIDAS ALCOHÓLICAS

DIGA:

- Si toman muchas bebidas alcohólicas, es posible que su presión arterial (ruchuq'a' kïk') se eleve. El alcohol también puede dañar el hígado, el cerebro y el corazón. Esto es importante tomar en cuenta cuando se está tratando de lograr o mantener un peso saludable.
- Si usted no bebe, no empiece. Si toma bebidas alcohólicas, hágalo sólo en cantidades moderadas. Esto quiere decir que:
  - Los hombres no deben tomar más de dos tragos por día.
  - Las mujeres no deben tomar más de un trago por día.
  - Las mujeres embarazadas o que estén dando el pecho no deben beber nada de alcohol.

7 | CONTROLE SU PRESIÓN ARTERIAL (RUCHUQ'A' KÏK') CON MEDICAMENTOS

DIGA:

- Si tienen la presión arterial alta (jotöl ruchuq'a' kïk'), quizás no sea suficiente hacer los cambios que mencionamos anteriormente para bajar su presión arterial (ruchuq'a' kïk'). Hay medicamentos disponibles para bajar la presión arterial (ruchuq'a' kïk'). Estos medicamentos funcionan de diferentes maneras.

DIGA:

- Si ustedes toman medicamentos para la presión arterial alta (jotöl ruchuq'a' kïk'), hay cosas que pueden hacer para que los medicamentos funcionen mejor. Lea en voz alta el siguiente:
  - Asegúrese de tomar el medicamento todos los días si el medico así se lo recetó, no solamente en los días en que no se sienta bien.
  - Dé al médico el nombre de cualquier otro medicamento, remedio casero, hierba o suplementos que tome. Lleve todo a su cita médica.
  - Avísele enseguida al médico si los medicamentos le hacen sentir mal o enfermo. Pregúnte al médico si le debería cambiar la dosis o el medicamento.
  - Compre su receta a tiempo para no quedarse sin el medicamento.
  - Mídase la presión arterial (ruchuq'a' kïk') a menudo para ver si los medicamentos le están haciendo efecto.
  - No deje de tomar sus medicamentos si su presión arterial (ruchuq'a' kïk') está bien. Eso quiere decir que los medicamentos están funcionando.

INFORMACIÓN ADICIONAL PARA EL PROMOTOR DE SALUD (TZIJÖY AQ'OMAB'ÄL) MEDICAMENTOS PARA LA PRESIÓN ARTERIAL ALTA (JOTÖL RUCHUQ'A' KÏK')

- Muchas personas que tienen la presión arterial alta (jotöl ruchuq'a' kïk') toman más de un medicamento para controlarla.
- Algunos medicamentos pueden causar reacciones adversas o efectos secundarios. Si esto pasa, llame al médico para que le ajuste la dosis o medicamento.
- NO AUTOMEDICARSE.
- NO DEJARSE LLEVAR POR CONSEJOS MEDICOS DICHOS POR OTRAS PERSONAS.
- Comer más frutas y verduras, menos sal, tomar más agua, perder peso y hacer más actividad física puede ayudar a que los medicamentos trabajen mejor.

HAGA : *Dé a los participantes la hoja "Use hierbas e especies en lugar de sal" (página 66). Pídales que esta semana usen algunas de las hierbas e especies en lugar de sal cuando cocinen.*

REPASO DE LOS PUNTOS MÁS IMPORTANTES DE HOY

DIGA:

· Repasemos lo que aprendimos hoy.

PREGUNTE:

· ¿Qué es la presión arterial (ruchuq'a' kïk')?

RESPUESTA: La presión arterial (ruchuq'a' kïk') es la fuerza que ejerce la sangre contra las paredes de las arterias (kaqib'och'ilal). La presión arterial (ruchuq'a' kïk') es necesaria para que la sangre circule por el cuerpo.

PREGUNTE:

· ¿Qué es una presión arterial normal (choj ruchuq'a' kïk')?

RESPUESTA: Una presión arterial normal (loman ruchuq'a' kïk') es menor de 120/80 mm Hg.

PREGUNTE:

· ¿Qué es la prehipertensión (uchuq'anem)?

RESPUESTA: La presión arterial (ruchuq'a' kïk') entre 120/80 y 139/89 mm Hg se llama prehipertensión (uchuq'anem). Esto quiere decir que aún no tiene presión arterial alta (jotöl ruchuq'a' kïk'), pero es probable que la desarrolle en el futuro a menos que haga cambios en sus hábitos de salud (rub'anelal raxnaqil).

PREGUNTE:

· ¿Qué es la presión arterial alta (jotöl ruchuq'a' kïk')?

RESPUESTA: La presión arterial alta (jotöl ruchuq'a' kïk') es de 140/90 mm Hg o más. Mídase su presión arterial (ruchuq'a' kïk').

PREGUNTE:

· ¿Por qué es peligrosa la presión arterial alta (jotöl ruchuq'a' kïk')?

RESPUESTA: La presión arterial alta (jotöl ruchuq'a' kïk') puede causar un ataque al corazón o derrame cerebral (rukamik k'uxaj o rukamik tzatzq'or), problemas de los riñones (kinäq') o de los ojos y la muerte.

PREGUNTE:

· ¿Qué pueden hacer para ayudar a que funcionen mejor sus medicamentos para la presión arterial alta (jotöl ruchuq'a' kïk')?

RESPUESTA: Comer más frutas y verduras y menos sal, tomar más agua, perder peso y hacer más actividad física. Tomar los medicamentos siguiendo las instrucciones del médico. Avisarle al médico si los medicamentos le hacen sentir enfermo.

PREGUNTE:

· ¿Por qué es importante reducir la sal en la comida?

RESPUESTA: Es importante comer menos sal para ayudar a prevenir o bajar la presión arterial alta (jotöl ruchuq'a' kïk').

PREGUNTE:

· ¿Cuáles son algunas maneras para reducir la sal?

RESPUESTA: Usar hierbas e especies para sazonar los alimentos pero ¡tenga cuidado! Algunos sazonadores como la sal de ajo o de cebolla tienen un contenido alto de sal. Fijarse en la etiqueta de los alimentos para escoger los que tienen menos sal. Comer más frutas y verduras entre las comidas en lugar de bocadillos salados como nueces, ricitos, tortrix, Doritos y plataninas. También consumir bebidas alcohólicas afecta la presión arterial (ruchuq'a' kïk').

Beber demasiadas bebidas alcohólicas puede elevar la presión arterial (ruchuq'a' kïk').

PREGUNTE:

· ¿Qué es un derrame cerebral (rukamik tzatzq'or)?

RESPUESTA: Un derrame cerebral (rukamik tzatzq'or) ocurre cuando la sangre repentinamente deja de llegar al cerebro. En el derrame cerebral (rukamik tzatzq'or), un vaso sanguíneo (rub'ey kïk') que va al cerebro se rompe. Pero el resultado es igual, las células (taq rujotayil k'aslemal) del cerebro no reciben oxígeno (uxlab'il) y se mueren.

PREGUNTE:

· ¿De qué otra manera se conoce derrame cerebral (rukamik tzatzq'or)?

RESPUESTA: Un derrame cerebral (rukamik tzatzq'or) es un problema muy grave que se conoce por varios nombres como ataque al cerebro, embolia cerebral, apoplejía o accidente cerebrovascular..

TAREA SEMANAL

DIGA:

- Han aprendido mucho hoy sobre cómo prevenir y cómo controlar la presión arterial alta (jotöl ruchuq'a' kïk'). También aprendieron como reducir la sal de sus dietas. Ahora pensemos cómo pueden poner en práctica lo que aprendieron. Les pido que piensen en un cambio que pueden hacer en su vida diaria. Esto será su tarea semanal.

NOTA : *Dé a los participantes 3 minutos para que piensen en una tarea.*

DIGA:

- Sean específicos sobre qué piensan hacer, cómo piensan hacerlo y cuándo comenzarán. Algunos ejemplos incluyen:
  - Desde mañana, pasaré los alimentos enlatados por agua antes de cocinarlos o comerlos.
  - A partir de mañana, quitaré el salero de la mesa.

HAGA : *Escriba las ideas para tareas en el pizarrón o papelógrafo pegado a la pared.*

PREGUNTE:

- ¿Alguno de ustedes quiere compartir su tarea con los demás?

(OPCIONAL) DIGA:

- Si mantenemos un valor personal en mente, éste nos puede ayudar a lograr cambios en nuestra vida diaria para prevenir y controlar la presión arterial alta (jotöl ruchuq'a' kïk'). Recuerden que un valor personal es una cualidad que ustedes consideren importante.

(OPCIONAL) DIGA:

- Nuestro valor de hoy es ser abierto. Si creen en ser abiertos significa que están dispuestos a vencer dudas y a tratar de hacer los cambios necesarios para mejorar su salud, como comer menos alimentos con alto contenido de sal.

NOTA : *Dé a los participantes 3 minutos para que piensen en una tarea.*

(OPCIONAL) PREGUNTE:

- ¿Cómo pueden usar el ser abiertos o otro valor para ayudarles a cumplir con su tarea?

DIGA:

- En la próxima sesión conversaremos sobre cómo les fue con su tarea.

CIERRE DE LA SESIÓN

DIGA:

- Gracias por asistir el día de hoy.
- ¿Qué comentarios me pueden dar acerca de la sesión?

NOTA : *Espere un rato a ver si los participantes tienen algún comentario.*

DIGA:

- Espero verlos en la próxima sesión que será dedicada a cómo prevenir y controlar el colesterol alto (jotöl ruchuq'a' ri saqab'uyül q'anal) en la sangre.

NOTA : *Reflexión para el presentador de la sesión. Piense en la sesión de hoy. ¿Qué cree usted que funcionó y qué no funcionó? ¿Qué cambió en su vida como resultado de lo que aprendió en esta sesión?*

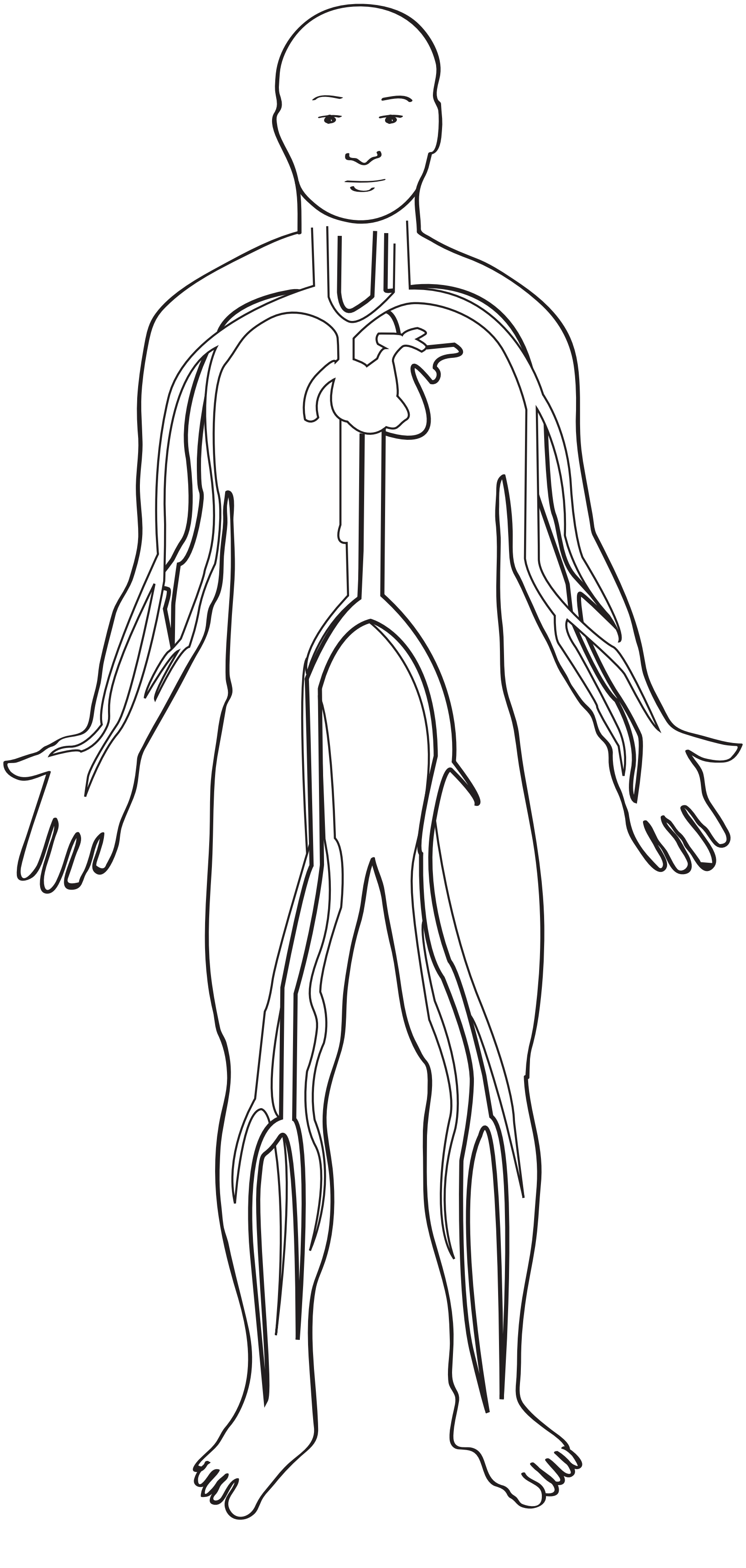

HÁBITOS SALUD-ABLES CONTRA LA PRESIÓNARTERIAL ALTA

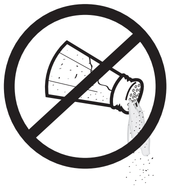

PARA PREVENIR PRESIÓN ARTERIAL ALTA:

1. Use menos sal. Prepare alimentos sin sodio o bajo en sal, utilizando más hierbas e especies en vez de sales y consomé.

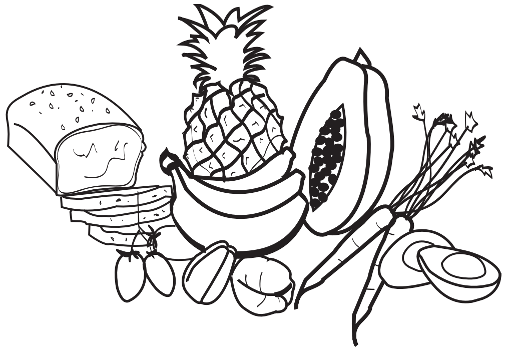

2. Coma más frutas, verduras, pan y cereales integrales y productos derivados de la leche sin grasa (descremada) o con bajo contenido de grasa (semidescremada).

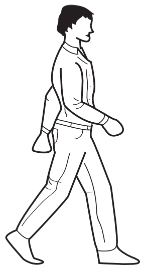

3. Haga actividad física todos los días. Puede caminar, practicar deportes o realizar cualquier actividad que le guste (incluyendo actividad física durante su trabajo).

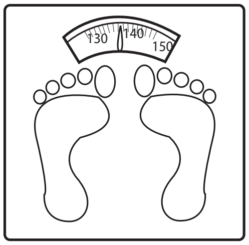

4. Mantenga un peso saludable. Trate de no aumentar de peso. Pierda peso si tiene sobrepeso. Trate de perder peso poco a poco, alrededor de 1 a 2 libras por semana hasta que alcance un peso saludable.

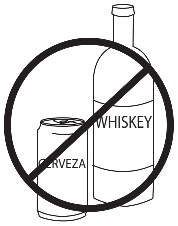

5. Reduzca el consumo de bebidas alcohólicas. Los hombres que beben no deben tomar más de uno o dos tragos por día. Las mujeres que beben no deben tomar más de un trago por día. Las mujeres embarazadas no deben beber nada de alcohol.

PARA BAJAR LA PRESIÓN ARTERIAL ALTA:

1. Lleve a la práctica estos consejos:

· Mantenga un peso saludable
· Haga de 30 a 60 minutos de actividad física todos los días.
· Coma menos alimentos con alto contenido de sal.
· Coma más frutas, verduras, pan y cereales de granos integrals y productos derivados de la leche sin grasa (descremada) o con bajo contenido de grasa (semidescremada).
· No tome bebidas alcohólicas.

2. Tome el medicamento todos los días como lo indica su médico.
3. Mídase la presión arterial con frecuencia.

CONOZCA LOS SÍNTOMAS DE UN DERRAME CEREBRAL. ACTÚE RÁPIDAMENTE

El derrame cerebral ocurre cuando la sangre repentinamente deja de llegar al cerebro y se mueren las células del cerebro por falta de oxígeno. El derrame cerebral es un problema muy grave y puede resultar en discapacidad o la muerte.

LOS SÍNTOMAS DE UN DERRAME CEREBRAL SON:

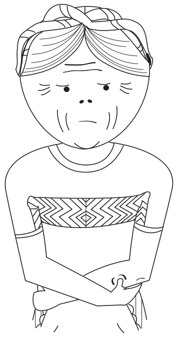

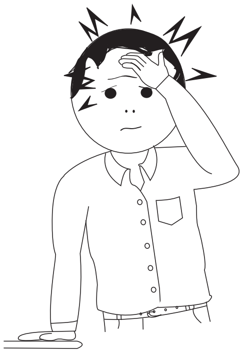

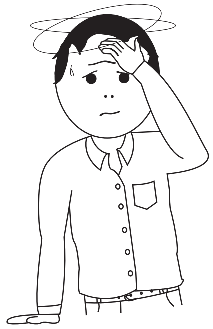

- Falta de sensación en la cara, brazo o pierna, especialmente en un solo lado del cuerpo
- Dolor de cabeza fuerte y repentino
- dificultad para caminar, mareo, pérdida del equilibrio o de la coordinación*

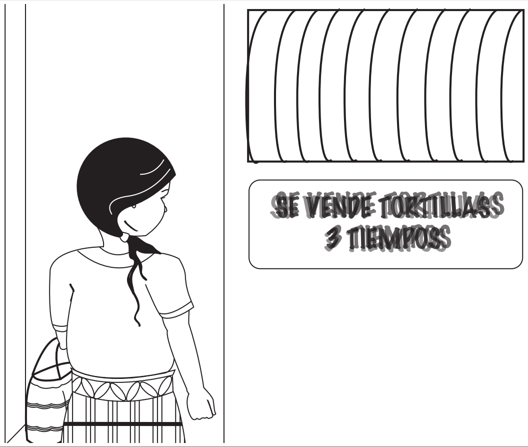

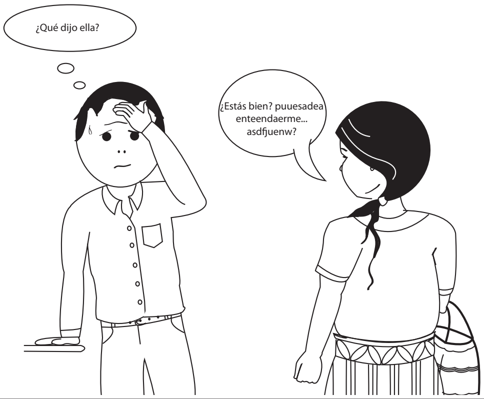

- Dificultad para caminar, mareo, dificultad para ver con uno o ambos ojos o de la coordinación
- Confusión, dificultad para hablar y para entender a los demás

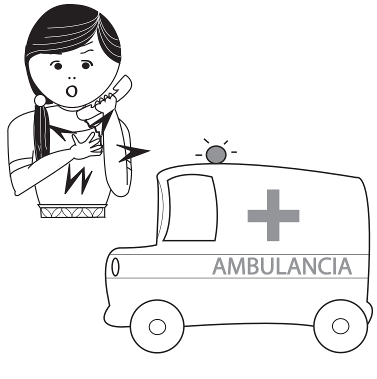

EL DERRAME CEREBRAL ES GRAVE, ¡CADA SEGUNDO CUENTA!

- Aprenda los síntomas de un ataque al cerebro y enséñeselos a sus amigos y familia.
- Llame al 122 o 123 inmediatamente si usted tiene cualquiera de estos síntomas.
- El tratamiento a tiempo puede reducir los daños causados por un ataque al cerebro. Usted debe obtener tratamiento durante las primeras 3 horas después de su primer síntoma.

El derrame cerebral se conoce por varios nombres, el más común, ataque cerebral. También puede llamarse embolia, ataque cerebrovascular o apoplejía.

EMPIECE HOY MISMO A HACER ESTOS CAMBIOS

CUANDO VAYA DE COMPRAS:

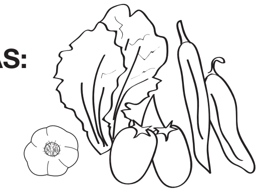

1. Compre verduras frescas.
2. Compre ajos frescos o en polvo en lugar de sal de ajo.
3. Verifique que la etiqueta de los alimentos diga: “bajo contenido de sal” o “sin sal agregada.

CUANDO COCINE:

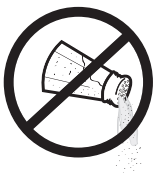

1. Poco a poco, vaya disminuyendo la cantidad de sal que usa, hasta que ya no la use más.
2. Cuando cocine frijoles, arroz, fideos o verduras, no ponga sal al agua.
3. Reduzca la cantidad de carne con alto contenido de sal como el jamón, las salchichas y el chorizo.

CUANDO COMA:

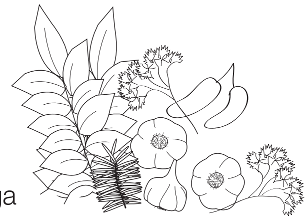

1. En lugar de sal, use hierbas e especies naturales.
2. Poco a poco, reduzca la cantidad de sal que mantenga en la mesa hasta no usar nada.
3. Elija frutas y verduras en lugar de bocadillos salados como papas fritas, plataninas, chicharrones y tostadas.

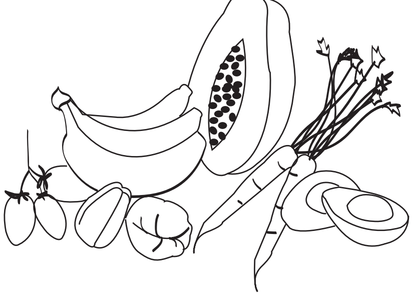

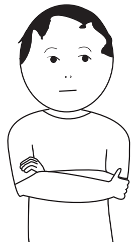

Xwan sabe controlar su presión arterial alta. Para no olvidarse, toma las pastillas para la presión arterial alta antes del desayuno todos los días. Camina a diario y ya no fuma. Xwan descubrió que la comida puede tener buen sabor con menos sal.

¡HAGA SU TAREA PARA LOGRAR LO QUE XWAN HA HECHO! CONSIDERE ESTOS EJEMPLOS:

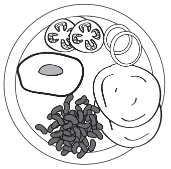

DESAYUNO

Coma 1 ó 2 tortillas de maíz con frijoles cocidos, y un huevo con tomate (chile pimiento) y cebolla y sin sal.

ALMUERZO

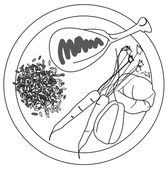

Prepare un arroz sazonado con tomate (chile pimiento) y cebolla, y la mitad de la sal que normalmente usa. Combínelo con carne asada con poca grasa y picadillo de tomate, y verduras cocidas con hierbas.

CENA

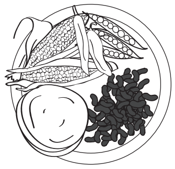

Prepare una mezcla de verduras en el sartén como güicoy, frijol cocido, elote, arveja, haba, cebolla y tomate. Sazónelo con hierbas como cilantro o perejil, la mitad de la sal que usaría, y pimienta. Sírvalo sobre tortillas de maíz.

ENTRE LAS COMIDAS

Coma una fruta en lugar de ricitos. Coma una rodaja de pan integral con 1 cucharada de frijol colado en lugar de jamón o embutidos.

La salud es oro. ¡Vale la pena invertir en su salud y en la de su familia!

USE HIERBAS E ESPECIES EN LUGAR DE SAL

- Chile (ik) en polvo: Úselo en sopas, frutas, verduras, ensaladas, carnes y pescados.
- Albahaca (tuq'si'j): Úsela en sopas, ensaladas, verduras, pescado y carnes.
- Canela (jub'ül che'): Úsela en atoles, panes y bocadillos.
- Cilantro (chïp): Úselo en carnes, caldos, salsas, estofados y arroz.
- Clavo de olor (q'utusimaj): Úselo en guisados, ensaladas y verduras.
- Jengibre (qutaq'oxe'): Úselo en sopas, ensaladas, verduras y carnes.
- Nuez moscada (amolo'em nimak'ilinaq'): Úsela en arroz.
- Orégano (jub'usi'j: Úselo en sopas, ensaladas, verduras, carnes y pollo.
- Perejil (rexil): Úselo en ensaladas, aguacates, pescado y carnes.
- Cebolla (xnakät): Úselo en carnes, pollos, sopas y ensaladas, frijoles, bistec, caldo de pollo y arroz.
- Romero (josjïk q'ayïs): Úselo en ensaladas, verduras, pescado y carnes.
- Tomillo (chaq'okal): Úselo en ensaladas, verduras, guisados, pescado y pollo.
- Hierba buena (kaxlan ixk'ij): Úselo con aguacate.
- Menta (achixk'i'j): Úselo con aguacate.
- Apio (kotichaj): Úselo en ensaladas, sopas, verduras y caldos de pollo o res.
- Ajo (anx): Úselo con los frijoles (kinäq').

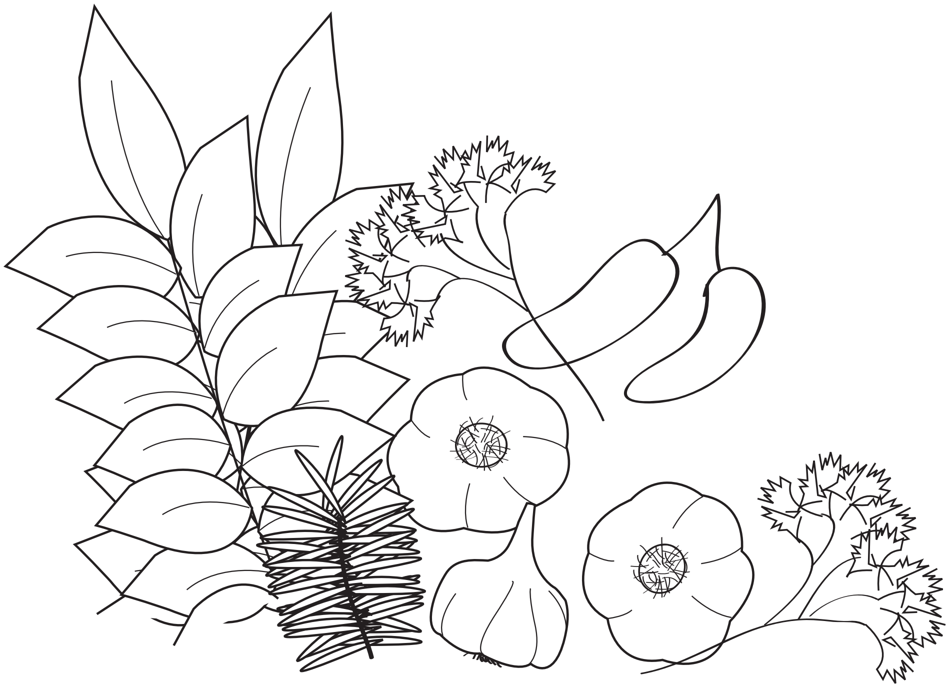

NOTA : *Para empezar, use pequeñas cantidades de estas hierbas e especies para ver si les gusta a usted y su familia.*

SESIÓN 5

Sea bueno con su corazón: Mantenga su colesterol bajo control (choj ruchuq'a' ri saqab'uyül q'anal)

OBJETIVOS

AL FINALIZAR ESTA SESIÓN, LOS PARTICIPANTES CONOCERÁN:

- Lo que es el colesterol (saqab'uyül q'anal) y cómo afecta al cuerpo
- Cuáles son los niveles saludables de colesterol
- Qué pasos tomar para bajar sus niveles de colesterol (taq ruchuq'a' saqab'uyül q'anal) en la sangre

MATERIALES

PARA DIRIGIR ESTA SESIÓN USTED NECESITARÁ:

- Pizarrón, papelógrafo, marcadores y cinta adhesiva
- Un juego de cucharas para medir*
- Una botella de aceite vegetal (ichajilal yaq'anal)*
- Escoja tres de los siguientes alimentos
  - Frijoles cocinados*
  - Una salchicha*
  - Una naranja*

* *Prepare antes de la sesión (vea la página 75). La margarina es necesaria para esta actividad pero puede usar un dibujo o foto de los demás alimentos.*

HOJAS PARA REPARTIR:

DURANTE LA SESIÓN, ENTREGUE A LOS PARTICIPANTES ESTA HOJAS:

- Lista de lugares donde los participantes pueden hacerse el examen del colesterol (saqab'uyül q'anal)*
- Las recetas acerca de "Cómo cocinar con menos grasa" (página 85).

* *Prepare esta lista antes de la sesión. Puede encontrar información en un hospital, clínica o centro de salud local.*

CARTELES PARA MOSTRAR:

- El cartel derivado de dibujo educativo 5.1 (página 81)
- El cartel derivado de dibujo educativo 5.2 (página 82)
- El cartel derivado de dibujo educativo 5.3 (página 83).
- El cartel derivado de la hoja "Haga algunos de estos cambios sencillos" (página 84)

ESQUEMA DE LA SESIÓN

INTRODUCCIÓN A LA SESIÓN

1. Bienvenida
2. Repaso de la sesión de la semana anterior
3. Acerca de esta sesión

LA SESIÓN EN ACCIÓN

1. Información sobre el colesterol (saqab'uyül q'anal) en la sangre
2. El colesterol (saqab'uyül q'anal) y las enfermedades del corazón (taq ruyab'ilal k'uxaj)
3. ¿Cuáles son sus niveles de colesterol (taq ruchuq'a' saqab'uyül q'anal)?
   a. ¿Qué significan los triglicéridos (ruwäch meq'anal)?
   b. El síndrome metabólico (retal na'il) y su salud
4. Arterias sanas (k'asäs kaqib'och'ilal)
5. Información sobre la grasa saturada (ritzelal q'anal), la grasa transaturada (nima'itzelal q'anal) y el colesterol (saqab'uyül q'anal) en la alimentación
6. Actividad: Cómo cocinar con menos grasa saturada (ritzelal q'anal)

REPASO DE LOS PUNTOS MÁS IMPORTANTES DE HOY

TAREA SEMANAL

CIERRE DE LA SESIÓN

INTRODUCCIÓN A LA SESIÓN

1 | BIENVENIDA

- Dé la bienvenida a los participantes a la sesión.

2 | REPASO DE LA SESIÓN DE LA SEMANA ANTERIOR

DIGA:

· La semana pasada hablamos de la sal.

NOTA : *Dé la repuesta correcta si los participantes no la saben.*

PREGUNTE:

· ¿Alguien recuerda por qué deberían tratar de limitar la cantidad de sal en lo que comen?

RESPUESTA: Se debe comer menos sal (atz'am) para ayudar a prevenir o bajar presión arterial alta (jotöl ruchuq'a' kïk')

PREGUNTE:

· ¿Alguien recuerda algunas maneras de reducir la cantidad de sal (atz'am) que comen?

RESPUESTA: Para reducir la sal, ustedes pueden:

· Usar hierbas e especies en lugar de sal para sazonar los alimentos.
· Entre las comidas, comer más frutas, verduras y productos derivados de la leche con bajo contenido de grasa (q'anal).
· Escoger nueces ó poporopos de maíz sin sal.

PREGUNTE:

· ¿Qué recuerdan sobre los beneficios de hacer más actividad física?

AÑADA LOS SIGUIENTES BENEFICIOS SI NO LOS MENCIONAN:

· La actividad física ayuda a:

· fortalecer el corazón y los pulmones
· formar y mantener saludable a los huesos, músculos (taq ti'ojilal) y articulaciones (laqul)
· sentirse mejor con usted mismo
· controlar el peso
· bajar la presión arterial alta (jotöl ruchuq'a' kïk')
· controlar el colesterol (saqab'uyül q'anal) en la sangre
· dormir mejor
· reducir el estrés (sachk'u'x) y los sentimientos de depresión y ansiedad
· tener más energía (uchuq'ab'il)
· disminuir la probabilidad de desarrollar diabetes (kab'yab'il), azúcar alta en la sangre, enfermedades del corazón (taq ruyab'ilal k'uxaj) y cáncer (sachöy jotay)

DIGA:

· Al terminar la última sesión ustedes hicieron la tarea de elegir alimentos con menos sal.

NOTA : *Dé a los participantes 3 minutos para responder.*

PREGUNTE:

· ¿Por qué no nos cuentan qué hicieron? ¿Qué problemas encontraron? ¿Cómo los resolvieron?

3 | ACERCA DE ESTA SESIÓN

DIGA:

- Hoy vamos a hablar sobre el nivel alto de colesterol (jotöl ruchuq'a' ri saqab'uyül q'anal) en la sangre como un factor de riesgo (rub'anikil rutzilal) para enfermedades del corazón (taq ruyab'ilal k'uxaj). Aprenderemos qué podemos hacer para mantener bajo el colesterol (saqab'uyül q'anal) en la sangre. También haremos juntos algunas actividades que muestran cuánta grasa saturada (ritzelal q'anal) tienen ciertos alimentos y cómo cocinar con menos grasa saturada (ritzelal q'anal). También aprenderemos cómo mantener baja la cantidad de grasas transaturadas (nima'itzelal q'anal) y colesterol (saqab'uyül q'anal) que consumimos.

LA SESIÓN EN ACCIÓN

1 | INFORMACIÓN SOBRE EL COLESTEROL (SAQAB'UYÜL Q'ANAL) EN LA SANGRE

DIGA:

- Si una persona tiene nivel demasiado alto de colesterol (yalan jotöl ruchuq'a' saqab'uyül q'anal) en la sangre, es más probable que desarrolle enfermedades del corazón (taq ruyab'ilal k'uxaj). Estos son algunos datos sobre el colesterol (saqab'uyül q'anal) en la sangre:
  - Un alto porcentaje de guatemaltecos tienen un nivel alto de colesterol (jotöl ruchuq'a' ri saqab'uyül q'anal) en la sangre y muchos de ellos lo ignoran.

NOTA : *En esta parte de la sesión se explica: ¿Qué es el colesterol (saquab'uyül q'anal)? ¿Porqué el cuerpo lo necesita? ¿Cuánto colesterol (saquab'uyül q'anal) necesita y de dónde proviene?*

2 | EL COLESTEROL (SAQAB'UYÜL Q'ANAL) Y LAS ENFERMEDADES DEL CORAZÓN (TAQ RUYAB'ILAL K'UXAJ)

DIGA:

- El colesterol (saqab'uyül q'anal) es una sustancia blanda como la cera (saqab'uyül achi'el ri sewa), parecida a la grasa (q'anal). El cuerpo utiliza el colesterol (saqab'uyül q'anal) para producir hormonas y algunas vitaminas (taq sanäm chuqa' taq uchuq'ab'äl).
- El colesterol (saqab'uyül q'anal) viene de dos fuentes:
  1. El colesterol (saqab'uyül q'anal) que circula por la sangre se llama colesterol sanguíneo (ruk'elal nimak'wanel q'anal).
  2. El colesterol (saqab'uyül q'anal) también proviene de los alimentos que comemos. Los alimentos que vienen de los animales tienen colesterol (saqab'uyül q'anal). El colesterol (saqab'uyül q'anal) que viene de los alimentos que aumentan el nivel de colesterol (ruchuq'a' saqab'uyül q'anal) son las grasas saturadas y las grasas transaturadas (taq ritzelal q'anal chuqa' taq nima'itzelal q'anal). Hoy aprenderemos cómo reducir el consumo de estas grasas (taq q'anal).

DIGA:

- Si tienen un nivel demasiado alto de colesterol (yalan jotöl ruchuq'a' saqab'uyül q'anal) en la sangre, corren más riesgo (retzelal) de tener un ataque al corazón (rukamik k'uxaj), derrame cerebral (rukamik tzatzq'or) y otros problemas de salud.

PREGUNTE:

NOTA : *Dé a los participantes un minuto para responder.*

- ¿Conocen a alguien que tiene un nivel alto de colesterol (jotöl ruchuq'a' saqab'uyül q'anal) en la sangre?

DIGA:

· Algunas cosas que afectan el nivel de colesterol (ruchuq'a' saqab'uyül q'anal) en la sangre no se pueden cambiar. Estas son: la edad, si se es hombre o mujer, o la historia familiar (si ustedes tienen parientes con nivel alto de colesterol (jotöl ruchuq'a' saqab'uyül q'anal). Hay cosas que sí pueden cambiar como el tipo de alimentos que comen, la cantidad de actividad física que hacen y su peso.

DIGA:

· Ustedes pueden lograr prevenir o bajar su nivel de colesterol (ruchuq'a' saqab'uyül q'anal) en la sangre tomando las siguentes medidas:

  · Comiendo una alimentación saludable para el corazón que sea baja en grasas saturadas (taq ritzelal q'anal), grasas transaturadas (taq nima'itzelal q'anal) y colesterol (saqab'uyül q'anal).
  · Tomando bastante agua pura.
  · Haciendo actividad física todos los días.
  · Manteniendo un peso saludable.

HAGA : *Muestre el cartel derivado del dibujo educativo 5.3 (página 83).*

DIGA:

· El colesterol (saqab'uyül q'anal) circula por la sangre en la forma de lipoproteínas (enimak'wanel q'anal). Hay dos tipos de lipoproteínas (enimak'wanel q'anal):

  · LDL: colesterol malo (itzel saqab'uyül q'anal), transportan el colesterol (saqab'uyül q'anal) a los vaso sanguíneos (taq rub'ey kïk') tapándolos como el sedimento o residuo en una tubería. Por esto, generalmente se le conoce al LDL: colesterol malo (itzel saqab'uyül q'anal).
  · El colesterol (saqab'uyül q'anal) también viaja en la sangre dentro de las lipoproteínas de alta densidad, o HDL (ütz saqab'uyül q'anal). El colesterol HDL (ütz saqab'uyül q'anal) ayuda a quitar colesterol (saqab'uyül q'anal) acumulado en las arterias (kaqib'och'ilal). Por eso es que a menudo se le conoce como HDL: colesterol bueno (ütz saqab'uyül q'anal).

3 | ¿CUÁLES SON SUS NIVELES DE COLESTEROL (TAQ RUCHUQ'A' SAQAB'UYÜL Q'ANAL)?

PREGUNTE:

· ¿Cómo pueden averiguar si tienen un nivel alto de colesterol (jotöl ruchuq'a' saqab'uyül q'anal) o de grasas (taq q'anal) en la sangre?

RESPUESTA: El colesterol (saqab'uyül q'anal) se mide con un análisis de sangre que puede llevarse a cabo en el consultorio de algun médico o en un lugar donde hacen este tipo de exámenes como en una feria de salud (runimaq'ij raxnaqil), laboratorios privados (mewinaqilem tojtob'äl), liga del corazón y otros.

· Un perfil lípido (wujil meq'anal) mide los niveles de cada tipo de grasa (q'anal) en la sangre. Mide el colesterol total, LDL: colesterol malo (itzel saqab'uyül q'anal), el colesterol HDL (ütz saqab'uyül q'anal) y los triglicéridos (ruwäch meq'anal) (hablaremos sobre los triglicéridos (ruwäch meq'anal) más adelante). Tienen que estar en ayunas unas 12 horas antes de hacerse este examen. Se recomienda que las personas mayores de 20 años se hagan este análisis.

NOTA : *Si los participantes preguntan, explíqueles que el colesterol (saqab'uyül q'anal) se mide en miligramos por decilitros (mg/dl).*

HAGA : *Muestre el dibujo educativo 5.1 con el cartel (página 81).*

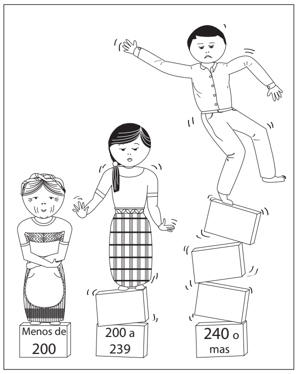

ESCRIBA EN UN PAPELÓGRAFO LA SIGUIENTE INFORMACIÓN:

¡HÁGASE CHEQUEAR!

- El perfil de lípidos mide el nivel de colesterol (ruchuq'a' saqab'uyül q'anal) y triglicéridos (taq ruwäch meq'anal).
- Se puede hacer la prueba en los servicios de salud.
- Las personas mayores de 20 años deben hacerse el perfil de lípidos (wujil meq'anal).

TOME ACCIÓN.

- Coma alimentos bajos en grasas (taq q'anal) y en colesterol (saqab'uyül q'anal), lo que le ayudará a bajar su nivel de colesterol (saqab'uyül q'anal) en la sangre, su peso y a prevenir las enfermedades del corazón (taq ruyab'ilal k'uxaj).

EL SIGNIFICADO DE SUS NIVELES DE COLESTEROL (SAQAB'UYÜL Q'ANAL): COLESTEROL TOTAL

Menos de 200 mg/dl	¡Buena noticia! Manténgase así!
200 a 239 mg/dl	¡Alerta! Este nivel es moderadamente alto y necesita cuidado. Es tiempo de hacer cambios en lo que come, en la cantidad de actividad física que hace y en su peso.
Mayor de 240 mg/dl	¡Peligro! Usted tiene un nivel alto y mucho riesgo (retzelal) de tener las arterias obstruidas (qat'el kaqib'och'ilal), lo que puede resultar en un ataque al corazón (rukamik k'uxaj). Consulte al médico.

EL COLESTEROL LDL O COLESTEROL MALO (ITZEL SAQAB'UYÜL Q'ANAL): ¡MANTÉNGALO BAJO!

Menor de 100 mg/dl	Deseable
100 a 129 mg/dl	Casi deseable
130 a 159 mg/dl	Moderadamente alto
Mayor de 160 mg/dl	Alto

El colesterol HDL o colesterol bueno (ütz saqab'uyül q'anal): Mientras más alto, ¡mejor!
Manténgalo mayor de 40 mg/dl.
Triglicéridos (ruwäch meq'anal): Mantenga sus (ruwäch meq'anal) en menos de 150 mg/dl.

INFORMACIÓN ADICIONAL PARA EL PROMOTOR (TZIJÖY AQ'OMAB'ÄL) NIVEL ALTO DE COLESTEROL (JOTÖL RUCHUQ'A' SAQAB'UYÜL Q'ANAL)

Menor de 200 mg/dL	Deseable. ¡Buena noticia!c
200 a 239 mg/dL	**Moderadamente alto**<br>▪ Dependiendo de los otros factores de riesgo (taq rub'anikil rutzilal) que tengan, pueden tener un mayor riesgo (nimak'ayew) para las enfermedades del corazón (taq ruyab'ilal k'uxaj).<br>▪ Pregúntenle al médico cuál es su riesgo (retzelal) para enfermedades del corazón (taq ruyab'ilal k'uxaj).
Mayor de 240 mg/dL	**Alto**<br>▪ Tienen un alto riesgo (retzelal) de tener las arterias obstruidas (q'atel kaqib'och'ilal) y un ataque al corazón (rukamik k'uxaj).<br>▪ Pregúntele al médico cuál es su riesgo (retzelal) para enfermedades del corazón (taq ruyab'ilal k'uxaj).

NIVELES EL COLESTEROL HDL (ÜTZ SAQAB'UYÜL Q'ANAL) Y LDL (ITZEL SAQAB'UYÜL Q'ANAL)

**LDL: colesterol malo (itzel saqab'uyül q'anal)**	**¡Manténganlo bajo!**
Menor de 100	Deseable
100 a 129	Casi deseable
130 a 159	Moderadamente alto
Mayor de 160	Alto

HDL: colesterol bueno (ütz saqab'uyül q'anal)
En los hombres: 40 – 50 mg/dl
En las mujeres: 50 – 60 mg/dl

HDL: COLESTEROL BUENO (ÜTZ SAQAB'UYÜL Q'ANAL)

**En los hombres**	**En las mujeres**
40 – 50 mg/dl	50 – 60 mg/dl

INFORMACIÓN ADICIONAL PARA EL PROMOTOR (TZIJÖY AQ’OMAB’ÄL)

El riesgo (retzelal) de enfermedades del corazón (taq ruyab’ilal k’uxaj) y el nivel de LDL: colesterol malo (itzel saqab’uyül q’anal). La meta para el nivel de LDL: colesterol malo (itzel saqab’uyül q’anal) es diferente para cada persona y depende de cuáles otros factores de riesgo (rub’anikil retzelal) tenga. El número de factores de riesgo (rub’anikil retzelal), como la presión arterial alta (jotöl ruchuq’a’ kïk’), un nivel bajo de colesterol HDL (qajnäq ütz saqab’uyül q’anal), una historia familiar de enfermedades del corazón (taq ruyab’ilal k’uxaj), edad y sexo, y el hábito de fumar, afectarán la meta para el nivel de LDL: colesterol malo (itzel saqab’uyül q’anal). Mientras más alto sea el nivel del LDL: colesterol malo (itzel saqab’uyül q’anal) y mientras más factores de riesgo (rub’anikil retzelal) se tengan, mayor es la probabilidad de desarrollar enfermedades del corazón (taq ruyab’ilal k’uxaj) o de tener un ataque al corazón (rukamik k’uxaj). El médico les puede ayudar a fijar una meta para su nivel de LDL: colesterol malo (itzel saqab’uyül q’anal).

A | ¿QUÉ SIGNIFICAN LOS TRIGLICÉRIDOS (RUWÄCH MEQ’ANAL)?

NOTA : *Dé a los participantes 1 minuto para que respondan.*

PREGUNTE:

· ¿Han oído hablar de los triglicéridos (ruwäch meq’anal)?

DIGA:

· Los triglicéridos (ruwäch meq’anal) son otro tipo de grasa (q’anal) en la sangre. Si ustedes consumen demasiada grasas (taq q’anal), azúcar, bebidas alcohólicas o fuman, el cuerpo hará más triglicéridos (ruwäch meq’anal). Cuando el nivel de triglicéridos (ruwäch meq’anal) está alto, se tiene un mayor riesgo (nimak’ayew) de enfermarse del corazón.

· Un nivel saludable de triglicéridos (ruwäch meq’anal) es menor de 150 mg/dl.

· Las personas con un nivel alto de triglicéridos (ruwäch meq’anal) a menudo tienen un nivel bajo de colesterol HDL (qajnäq ütz saqab’uyül q’anal). Las personas con diabetes (kab’yab’il) también pueden tener un nivel alto de triglicéridos (jotöl ruchuq’a’ ruwäch meq’anal) y un nivel bajo de colesterol HDL (qajnäq ütz saqab’uyül q’anal).

B | EL SÍNDROME METABÓLICO (RETAL NA’IL) Y SU SALUD

DIGA:

· Hoy vamos a aprender sobre el síndrome metabólico (retal na’il). El síndrome metabólico (retal na’il) es un trastorno que está formado por un grupo de cinco factores de riesgo (rub’anikil rutzilal) que afectan la salud del corazón.

· Muchas personas tienen este problema de salud y no saben que lo tienen.

HAGA : *Muestre el dibujo educativo 5.2 con el cartel (página 82).*

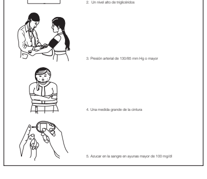

DIGA:

· Vamos a nombrar los cinco factores que forman el síndrome metabólico (retal na’il). Alcen la mano derecha y hagan un puño. Cuando nombre cada factor, levanten un dedo si ustedes tienen ese factor.

DIGA:

· Los cinco factores que forman el síndrome metabólico (retal na’il) son:

1. Un nivel bajo del colesterol HDL (qajnäq ütz saqab’uyül q’anal):
   · menor de 50 mg/dl para las mujeres
   · menor de 40 mg/dl para los hombres

HAGA : *El nivel de presión arterial (ruchuq'a' kïk') que se considera un factor de riesgo (rub'anikil retzelal) para el síndrome metabólico (retal na'il) es 130/85. Este nivel difiere del nivel de presión arterial alta (jotöl ruchuq'a' kïk') que se usa como definición de la presión arterial alta (jotöl ruchuq'a' kïk'), 140/90.*

2. Un nivel de triglicéridos (ruwäch meq'anal) de 150 mg/dl o más.
3. La presión arterial (ruchuq'a' kïk') de 130/85 mm Hg* o más (si cualquiera de los dos números está elevado, cuenta como factor de riesgo (rub'anikil retzelal)).
4. Niveles de azúcar en la sangre en ayunas más altos de lo normal, es decir, de 110 o más.
5. Una medida grande de cintura:
   - más de 35 pulgadas (88 cm) en las mujeres
   - más de 40 pulgadas (102 cm) en los hombres

PREGUNTE:

- ¿Cuántos dedos levantaron?

DIGA:

- Si ustedes tienen por lo menos tres de estos factores de riesgo (rub'anikil retzelal), tienen el síndrome metabólico (retal na'il).
- El síndrome metabólico (retal na'il) aumenta sus posibilidades de desarrollar enfermedades del corazón (taq ruyab'ilal k'uxaj), diabetes (kab'yab'il) o un derrame cerebral (rukamik tzatzq'or).
- Las personas con el síndrome metabólico (retal na'il) deben perder peso y hacer más actividad física.

DIGA:

- Si usted cree que tiene el síndrome metabólico (retal na'il), consulte con un medico o los Servicios de Salud.

4 | ARTERIAS SANAS (K'ASÄS KAQIB'OCH'ILAL)

DIGA:

- La sangre corre libremente a todas las células (taq rujotay k'aslem) del cuerpo cuando las las arterias (kaqib'och'ilal) están sanas y normales.
- Cuando el colesterol LDL: colesterol malo (itzel saqab'uyül q'anal) es demasiado alto, el colesterol (saqab'uyül q'anal) puede quedar atrapado en las paredes de las arterias endureciéndolas (kowiläj taq kaqib'öch'). La apertura de las arterias (kaqib'och'ilal) puede obstruirse, haciéndose más estrecha para el paso de la sangre.

DIGA:

- Los alimentos con alto contenido de grasa saturada y grasa transaturada (ritzelal q'anal chuqa' nima'itzelal q'anal) obstruirán las arterias (kaqib'och'ilal), que son las tuberías de sus cuerpos. Por lo tanto, queremos tratar a nuestro cuerpo tan bien como tratamos a las tuberías.

5 | INFORMACIÓN SOBRE LA GRASA SATURADA (RITZELAL Q'ANAL), LA GRASA TRANSATURADA (NIMA'ITZELAL Q'ANAL) Y EL COLESTEROL (SAQAB'UYÜL Q'ANAL) EN LA ALIMENTACIÓN

HAGA : *Escriba las respuestas que le den en un pizarrón o un papelógrafo pegado a la pared. Luego añada algunas soluciones posibles. Vea en el cuadro siguiente algunos problemas y sus soluciones.*

DIGA:

- Hoy vamos a hablar sobre los diferentes tipos de grasa (q'anal) en los alimentos y cómo afectan la salud del corazón (raxnaqil k'uxaj).
  - Hay dos tipos principales de grasa (q'anal): la grasa saturada (ritzelal q'anal) y la grasa insaturada (rutzil q'anal) y [la insaturada]. La mayoría de los alimentos contienen alguna combinación de estos tipos de grasa (q'anal).

- La grasa saturada (ritzelal q'anal) se encuentra en alimentos que vienen de animales y que tienen un nivel alto de grasa (q'anal) como las carnes, queso, leche y mantequilla. Coma menos de las grasas saturadas (taq ritzelal q'anal) porque son malos y aumentará sus probabilidades de desarrollar enfermedades del corazón (taq ruyab'ilal k'uxaj).
- Un tipo de grasa (q'anal) llamada grasa transaturada (nima'itzelal q'anal) también eleva el colesterol (saqab'uyül q'anal). La grasa transaturada (nima'itzelal q'anal) se encuentra en una variedad de productos horneados como: las galletas, tortas y pasteles, así como en las frituras y la margarina en barra. Seleccione la margarina suave (b'uyül q'ayiq'anal) liviana en tasa en vez de la de barra (dura).
- La grasa saturada (ritzelal q'anal) y la grasa transaturada (nima'itzelal q'anal) elevarán el nivel de colesterol (ruchuq'a' saqab'uyül q'anal) en la sangre más que cualquier otra cosa que usted coma. Esto aumentará sus probabilidades de desarrollar enfermedades del corazón (taq ruyab'ilal k'uxaj).

DIGA:

- Es importante limitar las grasas saturadas (taq ritzelal q'anal) y grasas transaturadas (taq nima'itzelal q'anal) que ustedes ingieran, pero un poco de grasa alimentaria (ilinel q'anal) es importante para mantenerse saludable. Las grasas (taq q'anal) son necesarias porque el cuerpo las utiliza para almacenar y dar energía (uchuq'ab'il).

PREGUNTE:

NOTA : *Dé a los participantes 2 minutos para responder.*
HAGA : *Escriba sus respuestas en el pizarrón o papelógrafo pegado a la pared.*

- ¿Qué tipos de grasa (q'anal) usan ustedes para cocinar sus comidas?

HAGA : *Muestre a los participantes una barra de mantequilla.*

DIGA:

- La mantequilla (qasq'anal) es un ejemplo de alimento con un alto contenido de grasa saturada (ritzelal q'anal).
- La grasa saturada (ritzelal q'anal) generalmente es sólida o dura a la temperatura ambiente y se encuentra en los alimentos derivados de animales.

DIGA:

- Los siguientes alimentos tienen un alto contenido de grasa saturada (ritzelal q'anal):

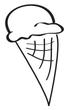

  - El helado
  - Los cortes de carne más grasosos, como la paletilla, la carne molida regular, las costillas, el chorizo y el tocino.
  - El pellejo del pollo, pavo, cerdo y los chicharrones
  - La mantequilla
  - Pan dulce
  - Tortillas y panes dulces o de manteca.

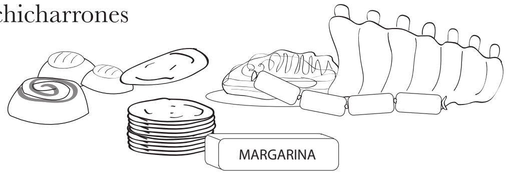

- Los siguientes alimentos tienen un contenido más bajo de grasa saturada (ritzelal q'anal) y grasa transaturada (nima'itzelal q'anal):
  - Los cortes de carne con bajo contenido de grasa (q'anal), como la espaldilla, el lomo, y la carne molida con contenido de grasa (q'anal) extra bajo
  - Pescado
  - Pollo o pavo sin pellejo
  - Arroz
  - Frijoles
  - Aceite vegetal (ichajilal yaq'anal)
  - Tortillas de maíz
  - Las frutas y verduras

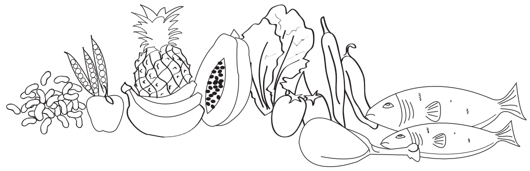

DIGA:

HAGA : *Muestre a los participantes una botella de [aceite vegetal].*

- Lo importante es limitar la cantidad de grasa saturada (ritzelal q'anal) que comen.

DIGA:

- Las grasas insaturadas (taq rutzil q'anal) generalmente son líquidas a temperatura ambiente. Cuando use grasas (taq q'anal) y aceites (taq yaq'anal), escoja los que tengan menos grasa saturada (ritzelal q'anal).
- Elija con más frecuencia (los más bajos en grasa saturada (ritzelal q'anal)):
  - Aceites de vegetal y soya.
- Elija con menos frecuencia (los más altos en grasa saturada):
  - Margarina en barra o grasa (q'anal) de mantequilla.

DIGA:

- Los alimentos con alto contenido de colesterol (saqab'uyül q'anal) también pueden elevar el colesterol (saqab'uyül q'anal) en la sangre y aumentar el riesgo (retzelal) de enfermedades del corazón (taq ruyab'ilal k'uxaj). El colesterol (saqab'uyül q'anal) alimentario se encuentra solamente en los alimentos provenientes de animales. Los siguientes son los alimentos con más alto contenido de colesterol (saqab'uyül q'anal):
  - las yemas de huevo
  - los órganos de los animales, también llamados vísceras, tales como el hígado, los riñones, los sesos, las tripas, el corazón y la lengua

DIGA:

- Los alimentos provenientes de las plantas no contienen el colesterol (saqab'uyül q'anal). Los alimentos que no contienen el colesterol (saqab'uyül q'anal) son:
  - las frutas como el banano, la piña, el mango y la manzana
  - las verduras como el tomate, el chile verde, la lechuga y el maíz
  - los frijoles de todas clases
  - el arroz como el arroz integral
  - los granos como los contenidos en las tortillas de maíz y el pan integral
  - los cereales como la avena

DIGA:

- Los alimentos derivados de las plantas generalmente tienen un contenido más bajo de grasa saturada (ritzelal q'anal) y no tienen el colesterol (saqab'uyül q'anal).

LEA LA SIGUIENTE HOJA:

¿QUÉ ELIGE IXCHEL PARA EL DESAYUNO?

Ixchel tiene poco tiempo en la mañana para preparar el desayuno. Generalmente desayuna con un pan dulce con 1/4 de taza de café y 2 cucharaditas de azúcar.

Ayuden a Ixchel a escoger algunos alimentos para el desayuno que tengan un contenido más bajo de grasa saturada (ritzelal q'anal), grasa transaturada (nima'itzelal q'anal) y el colesterol (saqab'uyül q'anal) que los que ella seleccionó.

NOTA : *la respuesta correcta está subrayada.*

PREGUNTE:

- ¿Un pan dulce o <u>una rodaja de pan desabrido (pan frances) con frijoles de la casa</u>?
- ¿Frijoles entalados o <u>frijoles de la casa</u>?
- ¿Café con leche o <u>café sin leche</u>?
- ¿Una pan dulce o <u>una champurrada de avena</u>?
- ¿Un pastel dulce con relleno de fruta o <u>un banano</u>?

NOTA : *Escriba las respuestas en el pizarrón o papelógrafo pegado a la pared. De ser necesario, ayude al voluntario a elegir algunos alimentos de reemplazo.*

PIDA A UN VOLUNTARIO QUE DESCRIBA:

- ¿Qué come generalmente para el desayuno?
- ¿Qué alimentos tienen un contenido más alto de grasa (q'anal)?
- ¿Qué alimentos con contenido más bajo de grasa (q'anal) pueden elegir para reemplazarlos?

HAGA : *Escribe los alimentos y cucharadas de grasa en el pizarrón o papelógrafo.*

Alimentos	Cucharadas de grasa (q'anal) (gramos de grasa (q'anal))
2 cucharadas de crema	Alrededor de 1 1/4 cucharadas (5 gramos)
1 onza de papalinas (unos 15)	Alrededor de 7 1/2 cucharadas (28 gramos)
1 salchicha	Alrededor de 4 cucharadas (16 gramos)
1 taza de frijoles cocidos	Alrededor de 1/4 cucharada (1 gramo)
1 naranja	Ninguna
2 pastelitos	Alrededor de 4 1/2 cucharadas (18 gramos)
1/2 papa cocida, preparada con tomate y cebolla	Ninguna
1 porción de sopa instantánea	Alrededor de 3 1/4 cucharadas (13 gramos)
1 bolsa de Tortrix, Ricitos, o Doritos	Alrededor de 2 1/2-4 cucharadas (10–16 gramos)

DIGA:

· Como pueden ver, es fácil sobrepasarse el límite de grasa (q'anal) recomendado cuando comemos alimentos con alto contenido de grasa (q'anal), como frituras, algunos alimentos preparados, salchichas, papalinas y helado. Para que sea más fácil consumir menos grasa (q'anal), coman más alimentos con bajo contenido de grasa (q'anal) como frutas, verduras, arroz y frijoles.

**Alimentos**	**Sustitutos con contenido más bajo de grasa (q'anal)**
2 huevos revueltos	Puede usar 1 huevo entero, y solo la clara de otro. O bien, si desea, únicamente las claras.
margarina	aceite vegetal (ichajilal yaq'anal) o de girasol
salchichas	salchicha con bajo contenido de grasa (q'anal), o de pechuga de pollo

DIGA:

· Ustedes también pueden reducir fácilmente la cantidad de grasa (q'anal) que comen si limitan los alimentos con alto contenido de grasa (q'anal) o los comen en porciones más pequeñas.

NOTA : *Dé a los participantes 2 minutos para responder.*
HAGA : *Escriba las respuestas en el pizarrón o en un papelógrafo pegado a la pared.*

6 | CÓMO COCINAR CON MENOS GRASA SATURADA (RITZELAL Q'ANAL)

PREGUNTE:

· ¿Antes de empezar esta actividad ¿me pueden decir qué pueden hacer para cocinar con menos grasa saturada (ritzelal q'anal)?

NOTA : *Lea en voz alta cada receta "Cómo cocinar con menos grasa" (página 85). Repase cada uno de los platos de la lista.*

DIGA:

· En la próxima actividad veremos cómo preparar alimentos con menos grasa saturada (ritzelal q'anal).

NOTA : *Dé a los participantes unos minutos para responder.*

PREGUNTE:

· ¿Por qué las recetas de la hoja contienen menos grasa saturada (ritzelal q'anal)?

AÑADA CUALQUIERA DE LAS SIGUIENTES RAZONES QUE NO SE MENCIONEN:

· Los frijoles de la casa tienen un bajo contenido de grasa (q'anal) si no se les añade grasa (q'anal).

· El arroz hervido naturalmente tiene un bajo contenido de grasa (q'anal) si no se le añade grasa (q'anal).

· Se asan las papas cocidas y no fritas.

· Se quita el cebo de la carne antes de preparla.

· El licuado de frutas se hace con agua.

NOTA : *Lea en voz alta la hoja "Haga algunos de estos cambios sencillos" y muestre el cartel (página 84).*

REPASO DE LOS PUNTOS MÁS IMPORTANTES DE HOY

DIGA:

- Ahora repasemos lo que han aprendido el día de hoy.

PREGUNTE:

- ¿Cuál puede ser la causa de que las arterias (kaqib'och'ilal) se obstruyan?

  RESPUESTA: La acumulación del colesterol (saqab'uyül q'anal) en las arterias (kaqib'och'ilal) las obstruye.

PREGUNTE:

- ¿Cuál es el nivel total de colesterol (saqab'uyül q'anal) en la sangre que se considera deseable?

  RESPUESTA: Menos de 200 mg/dl.

PREGUNTE:

- ¿Qué medidas pueden tomar para mantener bajo el nivel de colesterol (qajnäq saqab'uyül q'anal) en la sangre?

  RESPUESTA: comer una variedad de granos integrales, frutas y verduras
  - comer alimentos con bajo contenido de grasa saturada (ritzelal q'anal), grasa transaturada (nima'itzelal q'anal) y el colesterol (saqab'uyül q'anal)
  - mantener un peso saludable
  - mantenerse fisicamente activo

PREGUNTE:

- ¿Pueden nombrar algunos alimentos con alto contenido de grasa saturada (ritzelal q'anal)?

  RESPUESTA: los cortes de carne grasosos, el pellejo del pollo, la leche entera y la mantequilla (qasq'anal)

PREGUNTE:

- ¿Cuáles son algunas de las cosas que pueden hacer para que los alimentos tengan menos grasa (q'anal)?

  RESPUESTA: Quitar la grasa (q'anal) de la carne antes de cocinarla.
  - Quitar el pellejo del pollo o pavo y no comérselo.
  - Hornear, hervir, azar o cocinar a la parrilla los alimentos en lugar de freírlos.
  - Quitar la grasa (q'anal) de la sopa o caldo antes de servirlo.

PREGUNTE:

- ¿Pueden nombrar algunos alimentos saludables para el corazón?

  RESPUESTA: frutas como naranjas, peras, manzanas, duraznos y bananos
  - verduras como repollo, brócoli, zanahorias, alcachofa y habas verdes
  - legumbres como arvejas y frijoles
  - granos de cereal integral como la avena

TAREA SEMANAL

DIGA:

- Han aprendido mucho hoy sobre cómo prevenir el colesterol alto en la sangre (jotöl ruchuq'a' saqab'uyül q'anal) en la sangre y cómo comer menos grasa saturada (ritzelal q'anal), grasa transaturada (nima'itzelal q'anal) y el colesterol (saqab'uyül q'anal). Ahora veamos cómo pueden poner en práctica lo que han aprendido. Les pido que piensen en un cambio que puedan hacer en su vida diaria para mantener su colesterol bajo control (choj ruchuq'a' saqab'uyül q'anal). Esto será su tarea semanal.

DIGA:

- Sean específicos sobre qué piensan hacer, cómo piensan hacerlo y cuándo comenzarán. Aquí les doy unos ejemplos:
  - Le quitaré el pellejo al pollo y botaré el pellejo.
  - Cocinaré el pescado al horno en vez de freírlo con aceite (yaq'anal).

HAGA : *Escriba las ideas para tareas en el pizarrón o en un papelógrafo.*

PREGUNTE:

- ¿Quién quiere compartir con los demás la tarea que hizo?

(OPCIONAL) DIGA:

- Nuestro valor de hoy es la moderación. La moderación aumenta su capacidad de hacer selecciones saludables y de asumir la responsabilidad de los hábitos que necesita cambiar. La moderación también les ayuda a mejorar su manera de comer. Por ejemplo, puede ayudarles a limitar el tamaño de las porciones y a no servirse comida una segunda vez.

(OPCIONAL) DIGA:

- Nuestro valor de hoy es ser abierto. Si creen en ser abiertos significa que están dispuestos a vencer dudas y a tratar de hacer los cambios necesarios para mejorar su salud, como menos alimentos con alto contenido de sal, grasas saturadas (ritzelal q'anal), grasas transaturadas (nima'itzelal q'anal) y colesterol (saqab'uyül q'anal).

NOTA : *Dé a los participantes unos minutos para que compartan sus pensamientos.*

(OPCIONAL) PREGUNTE:

- ¿Cómo pueden usar la moderación u otro valor para ayudarse a cumplir con su tarea?

DIGA:

- En la próxima sesión conversaremos sobre cómo les fue con su tarea. Recuerden que deben continuar trabajando con su tarea de hacer más actividad física y de reducir la sal, grasas (taq q'anal) y colesterol (saqab'uyül q'anal) en su alimentación.

CIERRE DE LA SESIÓN

DIGA:

- Gracias por asistir el día de hoy.

NOTA : *Espere un rato a ver si los participantes tienen algún comentario.*

- ¿Qué comentarios me pueden dar acerca de la sesión?

DIGA:

  - Piense en la sesión de hoy. ¿Qué cree usted que funcionó y qué no funcionó? ¿Qué cambió en su vida como resultado de lo que aprendió en esta sesión?

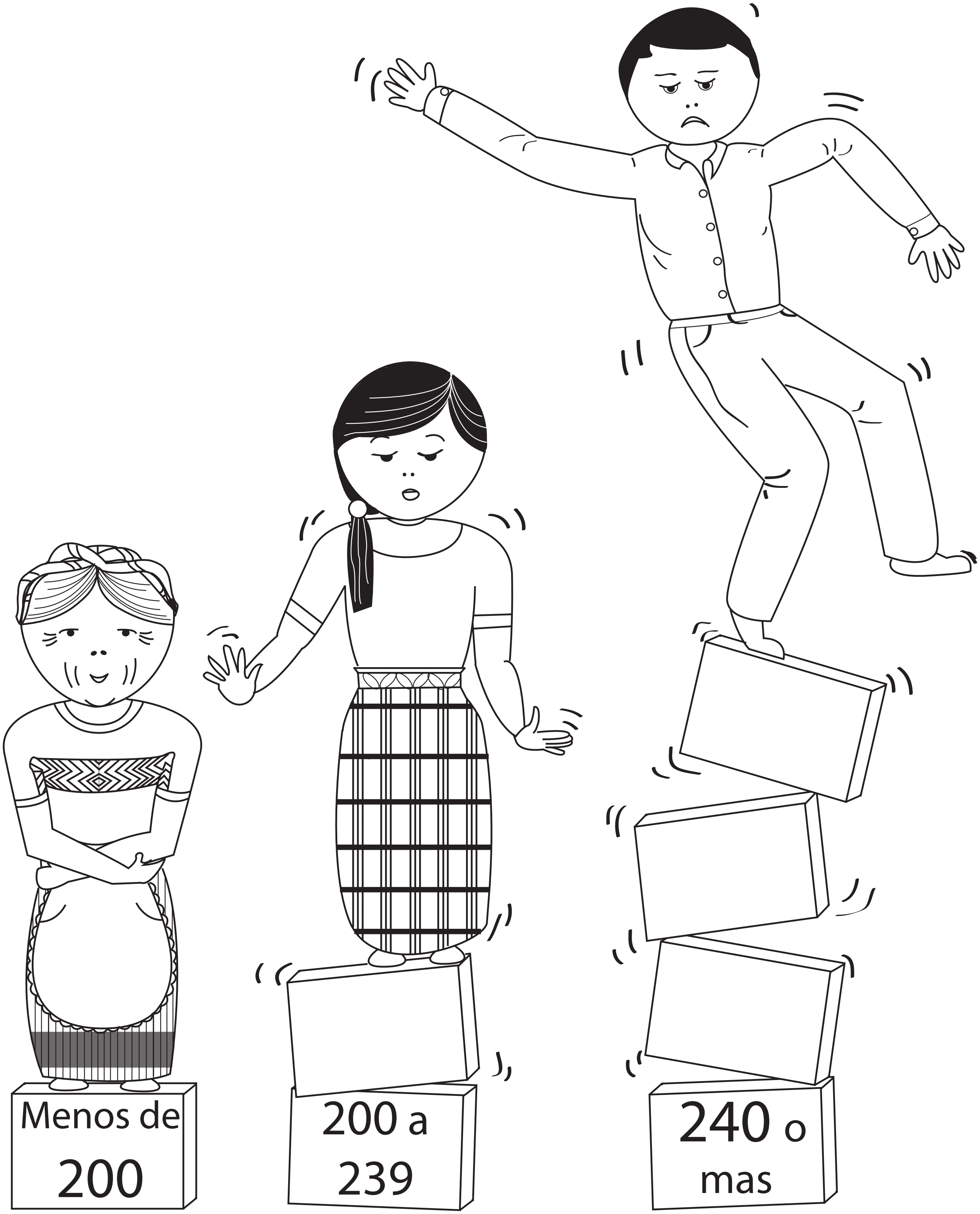
Menos de
200
200 a
239
240 o
mas

3 DE 5 SÍNTOMAS = EL SÍNDROME METABÓLICO

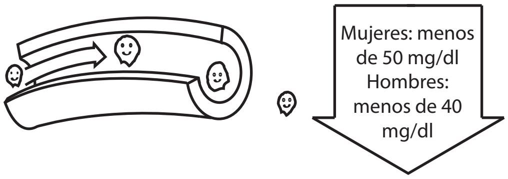

1. Un nivel bajo de colesterol HDL

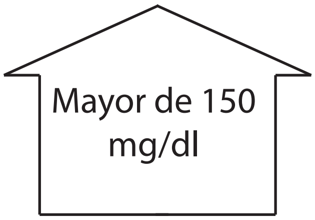

2. Un nivel alto de triglicéridos

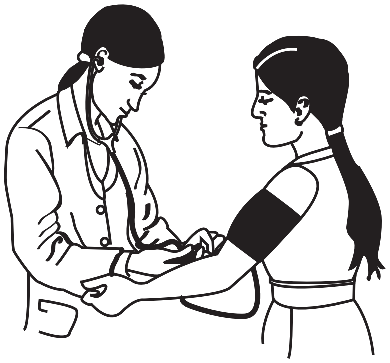

3. Presión arterial de 130/85 mm Hg o mayor

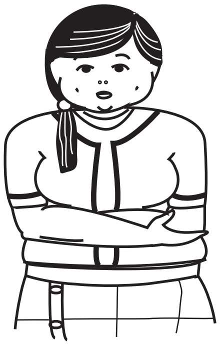

4. Una medida grande de la cintura

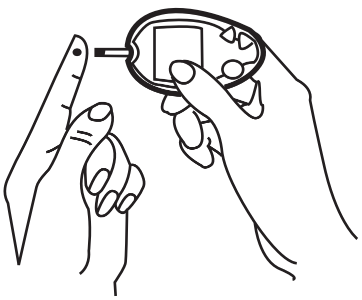

5. Azucar en la sangre en ayunas mayor de 100 mg/dl

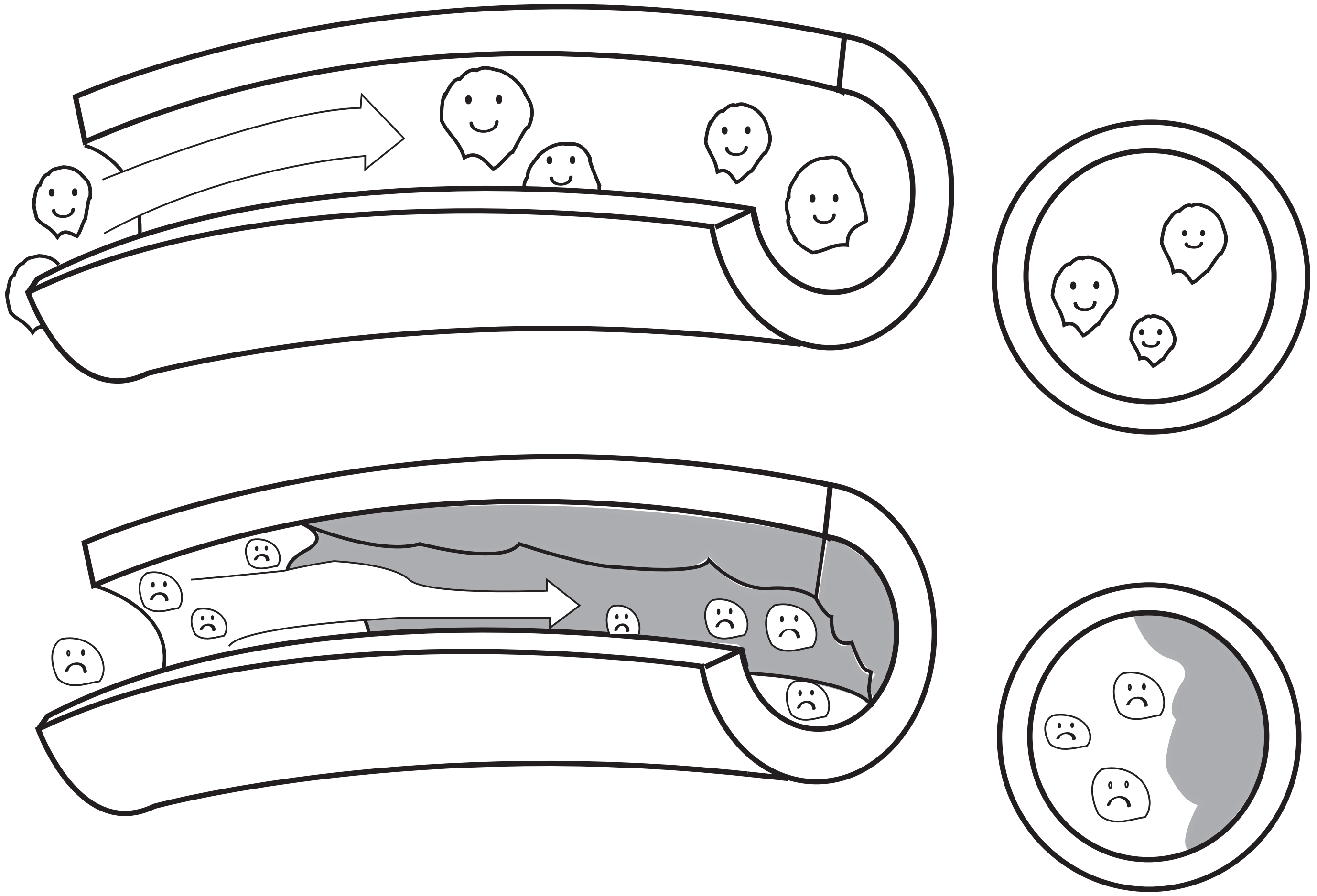

HAGA ALGUNOS DE ESTOS CAMBIOS SENCILLOS

CUANDO COCINE:

1. Recorte la grasa de la carne. Quite el pellejo y la grasa al pollo o pavo antes de cocinarlo.
2. Cocine quitándelo el cebo a la carne enjuagelo con agua caliente. Esto elimina la mitad de la grasa.
3. Enfríe la sopa y quítele la capa de grasa que se forma en la parte superior.

CUANDO COMA:

1. Use mayonesa, aderezos y crema sin grasa o bajos en grasa.
2. Use pequeñas cantidades de aceite vegetal en lugar de margarina.
3. Elija frutas y verduras en lugar de alimentos con mucha grasa, como papas fritas.

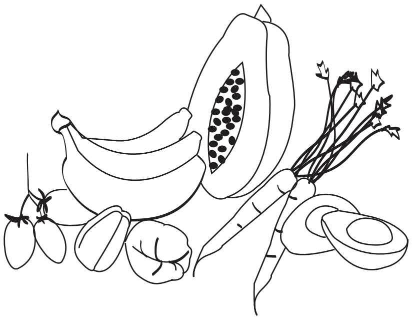

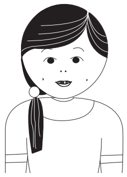

Ixchel sabe que si come alimentos con alto contenido de grasa saturada aumentará su nivel de colesterol. Por eso ha preparado su caldo favorito quitándole el cebo a la carne.

¡HAGA SU TAREA PARA LOGRAR LO QUE XWAN HA HECHO! CONSIDERE ESTOS EJEMPLOS:

DESAYUNO

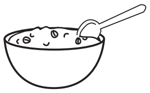

Escoja tortillas de maíz. Escoja cereales como avena cebada, en lugar de cereales refinados altos en azúcar.

ALMUERZO

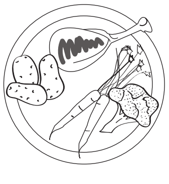

Coce papas con todo y cáscara, y sirva con el pollo asado sin pellejo y una ensalada de zanahoria cruda y brócoli.

CENA

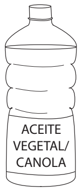

Quite toda la grasa visible de la carne, y cocine la carne con verduras utilizando aceite vegetal. Sirva con arroz integral.

ENTRE LAS COMIDAS

Elija un pan sin mucha azúcar (por ejemplo: pan frances) con 1/4 taza de frijoles.

La salud es oro. ¡Vale la pena invertir en su salud y en la de su familia!

CÓMO COCINAR CON MENOS GRASA

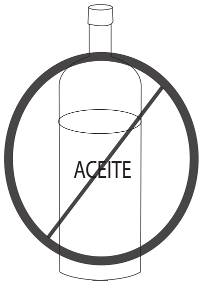

¡NO AGREGUE ACEITE!

FRIJOLES

1. Cocine los frijoles en agua hasta que estén blandos.
2. Condiméntelos con cebolla, ajo, comino, oregano, y si lo desea, 1/4 cucharadita de sal.
3. Para espesar los frijoles, cuelelos y cocínelos a fuego lento en una sartén con un poco de agua (sin aceite).

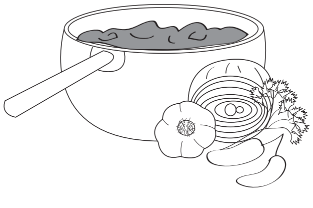

ARROZ HERVIDO

1. Hierva 2 tazas de agua y añada 1 taza de arroz, y cebolla, ajo y chile pimiento picados.
2. Tape bien y cocine a fuego lento por 20 minutos.

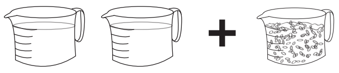

PAPAS O YUCA ASADA

1. Si está usando papas, déjelas con la cáscara. Si está usando yuca, pélela; y hiérva cualquiera que esté usando hasta que esté suave.
2. Corte las papas o la yuca en trozos. Colóquelas en un sartén rociado con una pequeña cantidad de aceite vegetal para que no se peguen. Puede también colocarlas en el comal con un poco de aceite vegetal hasta que estén dorados.
3. Cocine hasta que estén dorados, de ambos lados.

CALDO DE GALLINA BAJO EN GRASA

1. Corte cebolla, ajo, tomates, zanahoria, y güisquil en trocitos. Póngalos a cocinar en una olla con suficiente agua para hacer un caldo.
2. Quítele la piel a la gallina antes de ponerla en la olla con las verduras. Póngala en la olla.
3. Sazone con hierbas de su elección como cilantro, perejil, orégano, comino, etc.
4. Déjelo hervir hasta que esté completamente cocida la gallina, y sirva con un poco de arroz cocido de acuerdo a la receta de arriba.

CARNE CON PAPAS

1. En una sartén, dore el ajo y las cebollas picadas en 1 cucharadita de aceite vegetal. Añada la carne y cocine hasta que esté dorada. Cocine quitándelo el cebo a la carne enjuagelo con agua caliente.
2. Corte en cubos las papas con la cáscara.
3. Condimente con una pequeña cantidad de orégano, comino en polvo, pimienta negra, utilizando únicamente 1/4 de cucharadita de sal.
4. Cocine hasta que las papas estén suaves.

LICUADO DE FRUTA

1. Corte una fruta que le guste en trozos.
2. Ponga la fruta en la licuadora junto con leche sin grasa (descremada) o agua y vainilla.
3. Licúe hasta que esté cremosa.

SESIÓN 6 Proteja su corazón: Cuidese de la diabetes (kab'yab'il)

OBJETIVOS

AL FINALIZAR ESTA SESIÓN, LOS PARTICIPANTES CONOCERÁN:

- Lo que es la diabetes (kab'yab'il) y cómo afecta al cuerpo
- Los síntomas (taq k'u't) de la diabetes (kab'yab'il)
- Que la diabetes (kab'yab'il) es uno de los principales factores de riesgo (rub'anikil rutzilal) para las enfermedades del corazón (ruyab'ilal k'uxaj)
- Los niveles de azúcar en la sangre (taq ruchuq'a' kab' pa kïk') y su significado
- Cómo prevenir y controlar la diabetes (kab'yab'il)
- El contenido de azúcar en las bebidas comunes

MATERIALES

PARA DIRIGIR ESTA SESIÓN USTED NECESITARÁ:

- Pizarrón, marcadores, almohadilla, varias hojas grandes de papel y cinta adhesiva
- Dos botellas de agua*
- Colorante de comida roja*
- 1 taza de miel*
- Una bolsa de azúcar*

(OPCIONAL) NOTA : *Haga arreglos para que un profesional de la salud, promotor (tzijöy aq'omab'äl) o educador de diabetes (kab'yab'il) venga a la sesión para medir el nivel de azúcar en la sangre (ruchuq'a' kab' pa kïk') de los participantes.*

HOJAS PARA REPARTIR:

DURANTE LA SESIÓN, ENTREGUE A LOS PARTICIPANTES ESTA HOJAS:

- Lista de lugares donde los participantes pueden medirse el nivel de azúcar en la sangre (ruchuq'a' kab' pa kïk')**
- "¿Está en riesgo de desarrollar diabetes tipo 2?" (página 101)
- "Los síntomas de la diabetes" (página 102)
- "El buen cuidado de sus pies" (página 105)
- "Descubra el azúcar en su bebida favorita" (página 107)*

CARTELES PARA MOSTRAR:

- Cartel derivado de la hoja "¿Está en riesgo de desarrollar diabetes tipo 2?" (página 101).
- Cartel derivado de la hoja "Los síntomas de la diabetes" (página 102)
- Cartel derivado de dibujo educativo 6.1 (página 103)
- Cartel derivado de dibujo educativo 6.2 (página 104)
- Cartel derivado de la hoja "El buen cuidado de sus pies" (página 105)
- Cartel derivado de la hoja "Cuide su corazón: Conozca los pasos claves para controlar la diabetes" (página 106)

* *Prepare antes de la sesión (vea la pagina 89).*

** *Pesta lista antes de la sesión. Puede encontrar la información en un hospital, clínica o el departamento de salud local.*

ESQUEMA DE LA SESIÓN

INTRODUCCIÓN A LA SESIÓN

1. Bienvenida
2. Repaso de la sesión de la semana anterior
3. Acerca de esta sesión

LA SESIÓN EN ACCIÓN

1. Los hechos importantes
2. ¿Qué es la diabetes (kab'yab'il)?
3. ¿Cuáles son sus niveles de colesterol (saqab'uyül q'anal)?
4. Tipos de diabetes (kab'yab'il)
5. Los factores de riesgo (rub'anikil rutzilal) para la diabetes (kab'yab'il)
6. Los síntomas (taq k'u't) de la diabetes (kab'yab'il)
7. Los niveles de azúcar en la sangre (taq ruchuq'a' kab' pa kïk')
   a. La hipoglicemia (qajikab')
   b. La hiperglicemia (jotokab')
8. Las complicaciones de la diabetes (kab'yab'il)
9. Los pasos claves para controlar la diabetes (kab'yab'il)
10. Actividad. en grupo: Cómo mantenerse saludable con la diabetes (kab'yab'il)

REPASO DE LOS PUNTOS MÁS IMPORTANTES DE HOY

TAREA SEMANAL

CIERRE DE LA SESIÓN

INTRODUCCIÓN A LA SESIÓN

1 | BIENVENIDA

- Reciba cordialmente a los participantes.

2 | REPASO DE LA SESIÓN DE LA SEMANA ANTERIOR

DIGA:

· En la sesión anterior hablamos de lo importante que es reducir la grasa saturada (ritzelal q'anal), la grasa transaturada (nima'etzelal q'anal) y el colesterol (saqab'uyül q'anal) en nuestra alimentación.

PREGUNTE:

· ¿Pueden ustedes nombrar tres cosas que pueden hacer para que los alimentos tengan menos grasa (q'anal)?

SI LOS PARTICIPANTES NO LAS MENCIONAN, ÉSTAS SON ALGUNAS DE LAS POSIBLES REPUESTAS QUE SE PUEDEN DAR:

· Quitar la grasa (q'anal) de la carne antes de cocinarla.
· Quitar y tirar el pellejo de pollo antes de cocinarlo.
· Cocinar al horno, a la parrilla, hervir o asar los alimentos en lugar de freírlos.

NOTA : *Dé a los participantes 3 minutos para responder.*

PREGUNTE:

· ¿Qué hicieron para cumplir la tarea de la sesión anterior de comer menos la grasa saturada (ritzelal q'anal), la grasa transaturada (nima'etzelal q'anal) y el colesterol (saqab'uyül q'anal)?

· ¿Qué les funcionó?

· ¿Tuvieron algún problema?

3 | ACERCA DE ESTA SESIÓN

DIGA:

· En la sesión de hoy aprenderemos sobre lo que podemos hacer para prevenir y controlar la diabetes (kab'yab'il). Si alguno de ustedes tiene diabetes (kab'yab'il), tendrá que trabajar con un médico, un nutricionista (ajsöy ilinem) o promotor de salud (tzijöy aq'omab'äl) para que le ayuden a vigilar y controlar el nivel de azúcar en la sangre (ruchuq'a' kab' pa kïk').

LA SESIÓN EN ACCIÓN

1 | LOS HECHOS IMPORTANTES

DIGA:

- La diabetes (kab'yab'il) es un grave problema para las familias guatemaltecas. Afecta a hombres, mujeres y niños.
- Actualmente en Guatemala muchas personas adultas padecen de diabetes (kab'yab'il).
- La diabetes (kab'yab'il) es una enfermedad crónica que daña lentamente al cuerpo. Aunque no tiene cura, sus complicaciones se pueden prevenir.
- La diabetes (kab'yab'il) es uno de los principales factores de riesgo (rub'anikil rutzilal) para [las enfermedades del corazón].
- Las enfermedades del corazón (taq ruyab'ilal k'uxaj) son una de las principales causas de muerte para las personas con diabetes (kab'yab'il). Es muy probable que las personas mayores que tienen diabetes (kab'yab'il) también tienen alguna enfermedad del corazón (ruyab'ilal k'uxaj).

2 | ¿QUÉ ES LA DIABETES (KAB'YAB'IL)?

DIGA:

- Ahora hablaremos sobre lo que es la diabetes (kab'yab'il) y los diferentes tipos de diabetes (kab'yab'il).

DIGA:

- La diabetes (kab'yab'il) ocurre cuando el cuerpo no puede controlar la cantidad alta de azúcar en la sangre (jotöl ruchuq'a' kab' pa kïk') y eso puede dañar a las otras partes del cuerpo, por ejemplo los riñones, ojos y corazón.

PREGUNTE:

NOTA : *Dé a los participantes un minuto para responder.*

- ¿Qué es la prediabetes (lijöl kab'yab'il)?

  AÑADA LAS SIGUIENTES RESPUESTAS SI NADIE LAS MENCIONA:

  - La prediabetes (lijöl kab'yab'il) es una condición en la que los niveles de azúcar en la sangre (taq ruchuq'a' kab' pa kïk') se encuentran más altos de lo normal pero no lo suficientemente alto para ser diabetes (kab'yab'il).
  - Las personas con la prediabetes (lijöl kab'yab'il) tienen más probabilidad de desarrollar la diabetes (kab'yab'il) dentro de los próximos 10 años.
  - Las personas con la prediabetes (lijöl kab'yab'il) también tienen más probabilidad de tener un ataque al corazón (rukamik k'uxaj) o derrame cerebral (rukamik tzatzq'or).

3 | TIPOS DE DIABETES (KAB'YAB'IL)

DIGA:

- Hay dos tipos principales de diabetes (kab'yab'il).

LA DIABETES TIPO 1 (NAB'EY RUWÄCH KAB'YAB'IL):

- Se necesita usar una bomba (q'axb'äl) o inyecciones (tzuq'ub'äl) de insulina todos los días.
- Generalmente se encuentra en los niños, adolescentes o adultos jóvenes.
- Afecta alrededor del 5 al 10 por ciento de las personas con diabetes (kab'yab'il).

LA DIABETES TIPO 2 (RUKA'N RUWÄCH KAB'YAB'IL):

- Ocurre con más frecuencia en las personas que tienen sobrepeso y son inactivas por lo que se recomienda hacer ejercicio y bajar de peso. El riesgo (retzelal) de desarrollar la diabetes tipo 2 (ruka'n ruwäch kab'yab'il) aumenta si ustedes tiene algún familiar cercano– padre, madre, hermano o hermana – que tenga diabetes (kab'yab'il).
- El tratamiento es con pastillas (taq setaq'om) o a veces con inyecciones (tzuq'ub'äl) de insulina en algunos casos.
- Puede ocurrir a cualquier edad, pero es más común en personas mayores.
- Está aumentando en los niños, especialmente si tienen sobrepeso .
- Afecta alrededor del 90 al 95 por ciento de las personas con diabetes (kab'yab'il).

DIGA:

- La diabetes gestacional (yawa' kab'yab'il) es una clase especial de la diabetes tipo 2 (ruka'n ruwäch kab'yab'il):
  - Algunas mujeres embarazadas desarrollan este tipo de diabetes (kab'yab'il), pero generalmente desaparece cuando el bebé nace.
  - Las mujeres obesas y las mujeres con historia familiar de diabetes (kab'yab'il) también tienen un mayor riesgo (nimak'ayew) de tener diabetes gestacional (yawa' kab'yab'il).
  - Las mujeres que han tenido diabetes gestacional (yawa' kab'yab'il) tienen una mayor probabilidad de desarrollar la diabetes tipo 2 (ruka'n ruwäch kab'yab'il) más adelante en sus vidas. Sus hijos también tienen mayor riesgo (nimak'ayew) de tener sobrepeso y diabetes (kab'yab'il).

DIGA:

- En las diferentes sesiones, hemos hablado de cómo vivir sanamente. La diabetes tipo 2 (ruka'n ruwäch kab'yab'il) se puede prevenir y controlar eligiendo alimentos saludables, haciendo actividad física, y tomando los medicamentos que su médico le receta. Si alguno de ustedes tiene diabetes (kab'yab'il), es importante que esté bajo el cuidado de un médico y que se mida el nivel de azúcar en la sangre (ruchuq'a' kab' pa kïk') siguiendo las indicaciones de su médico.

DIGA:

- Ahora vamos a hacer una actividad para entender mejor lo que es la diabetes (kab'yab'il). Veremos la diferencia entre la sangre con un nivel normal de azúcar (choj ruchuq'a' kab') y la sangre con un nivel alto de azúcar (jotöl ruchuq'a' kab' pa kïk').

HAGA : *Antes de la sesión: Prepare la demostración del azúcar en la sangre.*

1. Coloque 2 botellas plásticas transparentes sobre la mesa.
2. Agregue una taza de agua y 3 gotas de colorante de comida roja en una botella Esta botella representa la sangre con un nivel normal de azúcar.
3. Agregue una taza de miel y 3 gotas de colorante de comida roja en la otra botella. Ésta representa la sangre con un nivel alto de azúcar.

HAGA : *Muestre la botella de agua sin azúcar y lentamente inclínela de un lado al otro para mostrar cómo el líquido se mueve fácilmente.*

DIGA:

- Fíjense cómo la sangre se mueve fácil y libremente. Esto representa la sangre con un nivel normal de azúcar.

HAGA : *Muestre la botella con miel y lentamente inclínela de un lado al otro para mostrar el movimiento lento del líquido.*

DIGA:

· Fíjense lo lento que se mueve la sangre y lo gruesa que es cuando el nivel de azúcar en la sangre está alto. Esto representa cómo es la sangre con un nivel alto de azúcar. El azúcar se acumula en la sangre en vez de ser utilizado en el cuerpo. Esto produce un nivel alto de azúcar en la sangre.

4 | LOS FACTORES DE RIESGO PARA LA DIABETES (KAB'YAB'IL)

NOTA : *Dé a los participantes 3 minutos para contestar.*
HAGA : *Escriba las respuestas en el pizarrón o en un papelógrafo pegado a la pared.*

PREGUNTE:

· ¿Alguien sabe cuáles son algunos de los factores de riesgo (rub'anikil rutzilal) que pueden aumentar sus probabilidades de desarrollar diabetes (kab'yab'il)?

HAGA : *Muestre el cartel derivado de la hoja "¿Está en riesgo (retzelal) de desarrollar diabetes tipo 2?" (página 101).*
HAGA : *Dé a los participantes la hoja "¿Está en riesgo (retzelal) de desarrollar diabetes tipo 2 (ruka'n ruwäch kab'yab'il)?" (página 101). Al leer cada factor de riesgo (rub'anikil rutzilal), pídales a los participantes que piensen sobre los factores de riesgo (rub'anikil rutzilal) que ellos tienen.*

DIGA:

· Lo bueno es que si están con riesgo (retzelal) de desarrollar diabetes (kab'yab'il), pueden retrasar su comienzo o prevenirla haciendo más actividad física y haciendo más actividad física y alimentación saludable.

5 | LOS SÍNTOMAS (TAQ K'U'T) DE LA DIABETES (KAB'YAB'IL)

NOTA : *Dé a los participantes 3 minutos para que contesten.*

PREGUNTE:

· ¿Alguien sabe cuáles son las señales o los síntomas (taq k'u't) de la diabetes (kab'yab'il)?

HAGA : *Dé a los participantes la hoja "Los síntomas de la diabetes" (página 102).*
HAGA : *Muestre el cartel derivado de la hoja "Los síntomas de la diabetes" (página 102).*

DIGA:

· Ahora, repasemos los síntomas (taq k'u't) de la diabetes (kab'yab'il):

- Cansancio
- Mucha sed
- Orinar frecuentemente
- Mucha hambre
- Pérdida inexplicable de peso
- Heridas o llagas que no se curan
- Piel muy seca
- Sensación de hormigueo en los pies
- Visión borrosa
- Irritabilidad

DIGA:

- Con frecuencia, las personas con diabetes tipo 2 (ruka'n ruwäch kab'yab'il) no tienen síntomas (taq k'u't). Si usted tiene uno o más de estos síntomas (taq k'u't), consulte a su médico inmediatamente.

HAGA : *Muestre el dibujo educativo 6.1 con el cartel (página 103).*

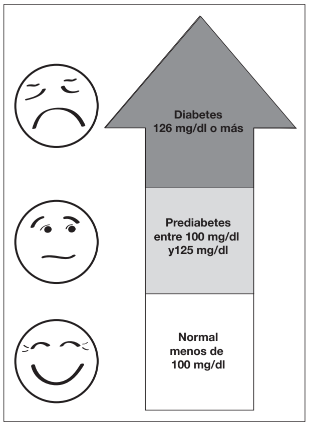

HAGA : *Muestre el dibujo educativo 6.2 con el cartel (página 104).*

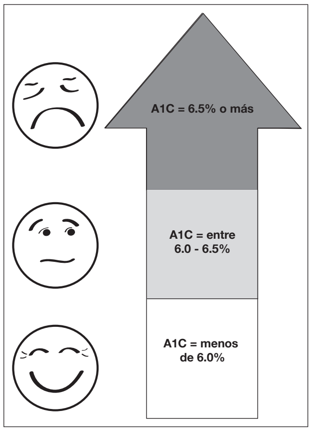

6 | LOS NIVELES DE AZÚCAR EN LA SANGRE

DIGA:

- Un nivel menor de 100 mg/dl de azúcar en la sangre en ayunas es normal. ¡Ésta es buena noticia!
    - Un nivel de azúcar en la sangre (ruchuq'a' kab' pa kïk') en ayunas entre 100 y 125 mg/dl es un nivel prediabético (lijöj kab'yab'inel). Es una señal de aviso de que están en riesgo (retzelal) de desarrollar diabetes tipo 2 (ruka'n ruwäch kab'yab'il). Es hora de mantener su dieta y de ponerse a hacer actividad física por todos los días.
    - Un nivel de azúcar en la sangre (ruchuq'a' kab' pa kïk') en ayunas de 126 mg/dl en adelante significa que tienen diabetes (kab'yab'il). Si tienen diabetes (kab'yab'il), visiten al médico y otros proveedores de cuidados de la salud para aprender a controlarla.
    - Es importante que las personas que tienen diabetes (kab'yab'il) aprendan los pasos clave para controlar la diabetes (kab'yab'il): hacerse la prueba de azúcar y mantener la presión arterial (ruchuq'a' kïk') y el colesterol (saqab'uyül q'anal) en niveles normales. Aprenderemos sobre estos pasos clave más adelante.

DIGA:

- Hay una prueba más exacta para medir el nivel de azúcar en la sangre (ruchuq'a' kab' pa kïk') y el diagnóstico de la diabetes llamada A1C. Mide en porcentaje (%).
    - Un porcentaje menos de 6.0% es normal. Esta es buena noticia!
    - Un porcentaje entre 6.0 – 6.5% es un nivel prediabético (lilöj kab'yab'inel). Es una señal de aviso de que están en riesgo (retzelal) de desarrollar diabetes tipo 2 (ruka'n ruwäch kab'yab'il). Es hora de mantener su dieta y de ponerse a hacer actividad física por todos los días.
    - Un porcentaje mayor de 6.5% significa que tienen diabetes (kab'yab'il). Si tienen diabetes (kab'yab'il), visiten al médico y otros proveedores de cuidados de la salud para aprender a controlarla.

DIGA:

- También se pueden hacer la prueba de azúcar sin estar en ayunas en las ferias de salud (runimaq'ij raxnaqil) y otros lugares en su comunidad. Esto se hace usando un glucómetro y una pequeña cantidad de sangre. Un nivel más de 125 mg/dl de azúcar en la sangre sin estar en ayunas, puede significar que usted tiene diabetes (kab'yab'il). Visiten a su médico para que les haga la prueba de azúcar en ayunas. Recuerden que deben ayunar por 8 horas.

INFORMACIÓN ADICIONAL PARA EL PROMOTOR DE SALUD (TZIJÖY AQ'OMAB'ÄL)
NIVELES DE AZÚCAR EN LA SANGRE (TAQ RUCHUQ'A' KAB' PA KÏK')

Nivel	A1C	Azúcar en la sangre en ayunas	1 a 2 horas después de las comidas	Resultados
Normal	≤ 6.0%	70–99 mg/dl	70–139 mg/dl	¡Bien hecho!
rediabetes (lijöl kab'yab'il)	6.0 – 6.5%	100–125 mg/dl	140–199 mg/ dl	Esto es una señel de que usted está en riesgo (retzelal) de desarollar diabetes tipo 2. La nivel de azúcar es más alto de lo nivel normal, pero, no tan alta que es la diabetes. Usted tiene que tomar medidas para prevenir la diabetes (kab'yab'il).
Diabetes (kab'yab'il)	≤ 6.5%	126 mg/dl o más	200 mg/dl o más	Usted tiene diabetes. Colaborar con su médico y otros organizadores de salud para controlar su nivel de azúcar (ruchuq'a' kab'), presión arterial (ruchuq'a' kïk') y colesterol (saqab'uyül q'anal)

PARA EXPLICAR LO QUE ES UN NIVEL DE A1C ES: Piense en cómo se adhiere el azúcar a las cosas, y cuando está adherido desde hace mucho tiempo, es más difícil de quitar. En el cuerpo, el azúcar se adhiere a los glóbulos rojos (käq rujotayil k'aslemal), en particular a las proteínas (taq ruchuq'a' ch'amich'äq). Los glóbulos rojos (käq rujotayil k'aslemal) que circulan en el cuerpo vivo por casi tres meses antes de morir. La prueba A1C mide el azúcar se ha adherido a las células (taq rujotay k'aslem) durante el tiempo anterior. Mide en porcentaje (%).

DIGA:

- Las personas con diabetes (kab'yab'il) pueden tener problemas si sus niveles de azúcar en la sangre suben o bajan demasiado.

A | LA HIPOGLICEMIA (QAJIKAB')

DIGA:

- La hipoglicemia (qajikab') se desarrolla cuando los niveles de azúcar en la sangre (taq ruchuq'a' kab' pa kïk') son bajos. Las personas con diabetes (kab'yab'il) pueden desarrollar hipoglicemia (qajikab').

DIGA:

- Las personas con diabetes (kab'yab'il) pueden desarrollar hipoglicemia (qajikab') cuando:
  - comen muy poco o saltan o retrasan una comida.
  - se inyectan demasiada insulina o toman demasiado medicamento oral para la diabetes.
  - hacen demasiada actividad física.
  - toman bebidas alcohólicas.

DIGA:

- los síntomas (taq k'u't) de la hipoglicemia (qajikab') son:
  - dolor de cabeza
  - sentirse tembloroso
  - ansiedad
  - mareo
  - debilidad
  - irritabilidad
  - sudor frío

DIGA:

- Las personas con diabetes (kab'yab'il) deben consultar con su médico para aprender qué deben hacer cuando tienen un nivel bajo de azúcar en la sangre (qajnäq ruchuq'a' kab' pa kïk').

DIGA:

- Las personas con diabetes (kab'yab'il) pueden sufrir de hipoglicemia (qajikab'). Si su nivel de azúcar baja a menos de 70 mg/dl, su médico les puede decir que coman o tomen uno de los siguientes:
  - 1/2 vaso de jugo (de naranja, manzana o uva)
  - 1 taza de leche
  - 1 taza de atole
  - 2 cucharaditas de azúcar o miel

B | LA HIPERGLICEMIA (JOTOKAB')

DIGA:

- La hiperglicemia (jotokab') ocurre cuando el nivel de azúcar en la sangre (ruchuq'a' kab' pa kïk') está demasiado alto. Las personas con diabetes (kab'yab'il) desarrollan hiperglicemia (jotokab') cuando:
  - comen demasiado.
  - Hacen menos actividad física de lo normal.
  - no toman sus medicamentos.
  - están enfermas o con mucho estrés (sachk'u'x) o tensión.

DIGA:

- Los síntomas (taq k'u't) de la hiperglicemia (jotokab') son:
  - sentir mucha sed
  - orinar frecuentemente
  - piel seca
  - sentir mucha hambre
  - visión borrosa
  - cansancio
  - náusea
  - irritabilidad

DIGA:

- Las personas con diabetes (kab'yab'il) deben consultar con su médico sobre qué deben hacer si tienen un nivel alto de azúcar en la sangre (jotöl ruchuq'a' kab' pa kïk').

DIGA:

- Si su nivel de azúcar en la sangre (ruchuq'a' kab' pa kïk') está demasiado alto, tal vez sea necesario cambiar sus medicamentos o su plan de alimentación.

7 | COMPLICACIONES DE LA DIABETES (KAB'YAB'IL)

DIGA:

- La diabetes (kab'yab'il) puede producir cambios en el cuerpo. Estos cambios pueden causar problemas, conocidos como complicaciones.

PREGUNTE:

- ¿Puede alguien nombrar alguna de las complicaciones de la diabetes (kab'yab'il)?

NOTA : *Dé a los participantes 3 minutos para contestar.*
HAGA : *Escriba las respuestas en el pizarrón o en papelógrafo pegado a la pared.*

DIGA:

- Con el tiempo, la diabetes (kab'yab'il) puede dañar los vasos sanguíneos grandes (taq nim rub'ey kïk') en el cerebro, el corazón, las piernas y los pies. También puede afectar a los vasos sanguíneos pequeños (taq ch'uti rub'ey kïk'), en los riñones y los ojos. La diabetes (kab'yab'il) puede resultar en:
  - Un ataque al corazón (rukamik k'uxaj)
  - Un derrame cerebral (rukamik tzatzq'or)
  - Amputación (qupinem) de los pies o de las piernas: La diabetes (kab'yab'il) puede causar daño a los nervios (k'amab'äl). La primera señal de daño a los nervios (k'amab'äl) es la pérdida de sensación en los pies o en las piernas. Si ustedes tienen dañado los nervios (k'amab'äl), pueden sentir dolor en los dedos de los pies, en las plantas de los pies o en las piernas.
  - Enfermedad de los riñones: Con la diabetes (kab'yab'il), el exceso de azúcar en la sangre daña los vasos sanguíneos (taq rub'ey kïk') dentro de los riñones.
  - Enfermedades de los ojos: La diabetes (kab'yab'il) puede dañar su vista o incluso causar la ceguera. Las personas con diabetes (kab'yab'il) deben hacerse un examen de los ojos con un médico especialista en los ojos (oftalmólogo) una vez al año.
  - Problemas dentales: Es común que las personas que no tienen un buen control de su diabetes (kab'yab'il) tengan un mayor riesgo (nimak'ayew) de caries (ruchikopil eyaj) e infecciones dentales (taq ruchikopil eyaj). Para evitar el daño de sus dientes, cepílleselos y use hilo dental todos los días.

DIGA:

- Con el tiempo, la diabetes (kab'yab'il) puede afectar todas las partes del sistema nervioso (k'amab'äl). Esto puede llevar a:
  - la pérdida de sensación en los pies o en las piernas (k'isnäq na'onem pa aqanaj). (Por eso tiene que mantener sus pies limpios, secos, suaves y protegidos).
  - pérdida de fuerza en los músculos (k'isnäq uchuq'a' pa ti'ojilal
  - cambios en la digestión, en el control de la vejiga (etan retal ri yakchulaj) y en la función sexual (rusamaj kojolwinaqil).

HAGA : *Entregue y repase con los participantes la hoja "El buen cuidado de sus pies" (página 105). Muestre el cartel.*

8 | LOS PASOS CLAVE PARA CONTROLAR LA DIABETES (KAB'YAB'IL)

DIGA:

· Si ustedes tienen diabetes (kab'yab'il), mantener sus niveles de azúcar en la sangre bajo control puede ayudarles a prevenir o retrasar las complicaciones.

HAGA : *Muestre el cartel "Cuide su corazón: Conozca los pasos claves para controlar la diabetes" (página 106).*

DIGA:

· Hemos aprendido que las personas con diabetes (kab'yab'il) tienen mayor probabilidad de tener un ataque al corazón (rukamik k'uxaj) o un derrame cerebral (rukamik tzatzq'or). Ahora veamos cómo cuidar al corazón, aprendiendo los pasos claves para controlar la diabetes (kab'yab'il).

HAGA : *Muestre el cartel derivado de la hoja "Descubra el azúcar en su bebida favorita" (página 107).*

DIGA:

· Ustedes vean la cantidad de azúcar que contiene su bebida favorita.

DIGA:

· Encuentren su bebida favorita en esta lista. Vean cuánta azúcar tiene la bebida. Muchas bebidas tienen un alto contenido de azúcar.

DIGA:

· Como pueden ver, es fácil que las personas que eligen muchas bebidas con azúcar. El alto consumo de azúcar resulta en sobrepeso. Traten de beber más agua o bebidas no azucaradas en lugar de bebidas con azúcar o gaseosas no dietéticas.

NOTA : *Esta actividad ayudará a los participantes a pensar en maneras que puedan ayudar a que sus parientes y amigos con diabetes (kab'yab'il) se mantengan saludables. Usted puede escoger una o más escenas para repasar.*

9 | CÓMO MANTENERSE SALUDABLE CON LA DIABETES (KAB'YAB'IL)

HAGA : *Lea las hojas de "Como mantenerse saludable con la diabetes: Historias de la vida real (páginas 95 – 97).*

DIGA:

· Lea la escena y los participantes piensen en soluciones para resolver el problema. Escojan a un miembro de su grupo para que lea en voz alta la escena y escriban las repuestas de su grupo a cada una de las preguntas.

NOTA : *Dé a los participantes 7 minutos para completar esta actividad.*

HAGA : *Lea en voz alta la historia y dé repuestas a las preguntas.*

HAGA : *Después ha leído sus respuestas, pregunte al resto del grupo si tienen otras soluciones que puedan compartir.*

10 | ACTIVIDAD EN GRUPO: CÓMO MANTENERSE SALUDABLE CON LA DIABETES (KAB'YAB'IL)

ESCENA 1: LA FIESTA DE CUMPLEAÑOS DE XWAN

El sábado es el cumpleaños de Xwan. Su familia le hará una gran fiesta y muchos parientes traerán comida deliciosa y postres como pasteles, tortas y helados. Ixchel no ha estado en una fiesta familiar desde que su médico le dijo que tenía diabetes (kab'yab'il). Ella se ha esforzado para aprender a comer saludablemente. Ahora, Ixchel se pregunta qué platillo puede llevar a la fiesta de cumpleaños y qué alimentos debería comer mientras está allí.

PREGUNTE:

· ¿Qué puede hacer Ixchel para comer saludablemente mientras está en la fiesta?

RESPUESTA: comer tortillas de maíz

· comer pan desabrido (pan frances) en lugar de pan dulce
· comer porciones más pequeñas
· No repetirse una segunda porción de alimentos con mucho azúcar. Si tiene hambre, puede comer más ensalada de fruta fresca.
· Consumir menos alimentos dulces, alimentos con alto contenido de grasas (q'anal), salsas blancas, salsas de las carnes y bebidas alcohólicas.

NOTA : *Dé las siguientes respuestas a cada pregunta, si no se mencionan.*

PREGUNTE:

· ¿Qué más puede hacer Ixchel?

RESPUESTA: Comer un bocadillo antes de la fiesta para no tener mucha hambre.

· Caminar después de comer.

ESCENA 2: LOS ZAPATOS NUEVOS DE LU'

Lu' se compró un nuevo par de zapatos hace una semana. Se los puso dos días seguidos porque no le molestaban los pies. Sin embargo, al quitarse los zapatos al final del segundo día, Lu' notó unas manchas rojas en ambos pies y ampollas en su dedo gordo del pie derecho. Se pregunta qué podría hacer con las manchas rojas y las ampollas en sus pies. Lu' está preocupado porque no sintió las llagas en sus pies. También se preocupa de que su diabetes no está bajo control.

PREGUNTE:

· ¿Qué puede hacer Lu' sobre las llagas en sus pies?

RESPUESTA: Las personas con diabetes deben ir a un médico si tienen una cortadura, ampollas o cualquier señal de infección en sus pies.

· Lu' debe hacerse un examen completo de los pies que incluya una inspección, pruebas de sensación y otras pruebas más.
· Como Lu' ya no tiene sensación en sus pies, todo los días debe prestar cuidados especiales a sus pies, fijándose cuidadosamente para ver si hay algún cambio o infección.

NOTA : *Dé las siguientes respuestas y sugerencias si no se mencionan.*

NOTA : *Dé las siguientes respuestas a las preguntas, si no se mencionan.*

PREGUNTE:

· ¿Qué deben tomar en cuenta las personas con diabetes (kab'yab'il) cuando compran zapatos?

RESPUESTA: Sea cuidadoso cuando compra zapatos nuevos.

· Escoja zapatos que le queden bien y sean de cuero o de lona, con cordones o correas, un forro liso, puntas redondeadas, tacos bajos y firmes, y plantillas suaves.
· Pídale al vendedor de zapatos que le mida ambos pies. Pruebe los zapatos caminando con ellos por lo menos unos 5 minutos en la tienda.
· Si le aprietan o duelen los zapatos, ¡no los compre!
· Acostúmbrese a los zapatos poco a poco, usándolos solamente de 1 a 2 horas por día. Cada vez que se quite sus medias y zapatos, vea si sus pies están irritados o rojos en alguna parte. Nunca use zapatos nuevos todo el día.

ESCENA 3: ¿QUÉ LE PASA A IXTZ'UNUN?

Primera visita: Ixtz'unun visita a su hermana Ixq'anil, que es una promotora (tzijöy aq'omab'äl). Ixtz'unun le dice que está cansada, con sed todo el tiempo y que le dan deseos de orinar con frecuencia. Le pregunta si cree que ella pudiera tener diabetes (kab'yab'il).

PREGUNTE:

· ¿Qué le puede decir Ixq'anil a Ixtz'unun?

RESPUESTA: Aconsejarle que vaya a la clínica y se haga la prueba de azúcar en la sangre. Si tiene diabetes (kab'yab'il), debe ser tratado de inmediato. Ixq'anil no puede hacer un diagnóstico.

· Explicarle que es más probable tener diabetes (kab'yab'il) cuando se tiene sobrepeso y no se hace mucha actividad física. Otros factores de riesgo (rub'anikil rutzilal) para la diabetes (kab'yab'il) incluyen (1) tener uno o ambos padres con diabetes (kab'yab'il), (2) tener otros miembros de la familia con diabetes (kab'yab'il), y (3) edades mayores.

Segunda visita: Ixtz'unun visita a Ixq'anil de nuevo. Le dice que fue al doctor quien le confirmó que tiene diabetes (kab'yab'il). Ixtz'unun tiene miedo.

PREGUNTE:

· ¿Cómo puede Ixq'anil ayudar a que Ixtz'unun venza su miedo de tener diabetes (kab'yab'il)?

RESPUESTA: Decirle que la mayoría de las personas con diabetes (kab'yab'il) sienten miedo, tristeza o ira en algún momento u otro.

· Decirle que algunas personas con diabetes (kab'yab'il) vencen al miedo cuando aprenden lo que pueden hacer para controlar la diabetes (kab'yab'il) y mantenerse saludables.

· Animarla a que vaya a clases con promotoras (taq tzijöy aq'omab'äl) y se haga miembro de un grupo de apoyo que ayuda a las personas con diabetes (kab'yab'il) a mantenerse saludables. Compartir los sentimientos con otras personas que viven con la diabetes (kab'yab'il) le puede servir de ayuda.

· Animarla a que le pida a su médico que le mida su presión arterial (ruchuq'a' kïk')y el colesterol (saqab'uyül q'anal). También animarla a que aprenda cómo disminuir su riesgo (retzelal) de tener un ataque al corazón (rukamik k'uxaj) o derrame cerebral (rukamik tzatzq'or).

· Aconsejarla que vaya con algún médico especialista de los ojos (oftalmólogo) para que le verifique los ojos y detecte cualquier problema que pueda tener.

REPASO DE LOS PUNTOS MÁS IMPORTANTES DE HOY

DIGA:

- Ahora repasemos lo que hemos aprendido.

PREGUNTE:

- ¿Que es la diabetes (kab'yab'il)?

RESPUESTA: La diabetes (kab'yab'il) ocurre cuando el cuerpo no puede controlar la cantidad alta de azúcar en la sangre y eso puede danar a las otras partes del cuerpo, por ejemplo los riñones, ojos y corazón.

PREGUNTE:

- ¿Cuales son los factores de riesgo (rub'anikil rutzilal) para la diabetes (kab'yab'il)?

RESPUESTA: Entre los factores de riesgo (rub'anikil rutzilal) para la diabetes (kab'yab'il) están:

- Miembros de la familia que tienen diabetes (kab'yab'il)
- El sobrepeso
- Tener edad avanzada
- Hacer poca actividad física
- Haber tenido diabetes gestacional (yawa' kab'yab'il)
- Presión arterial alta (jotöl ruchuq'a' kïk')
- Niveles anormales de colesterol (saqab'uyül q'anal); cuando el colesterol (saqab'uyül q'anal) está bajo o el nivel de triglicéridos (taq ruwäch meq'anal) está alto.

PREGUNTE:

- ¿Cómo se puede prevenir o retrasar el comienzo de la diabetes tipo 2?

RESPUESTA: Se puede prevenir o retrazar el comienzo de la diabetes tipo 2 (ruka'n ruwäch kab'yab'il):

- Siguen un plan de alimentación saludable y hacen actividad física.

PREGUNTE:

- ¿Cuáles son los pasos claves para controlar la diabetes (kab'yab'il)?

RESPUESTA: Es necesario hacerse la prueba de sangre llamada A1C por lo menos dos veces al año. Esta prueba sirve para medir como ha controlado el nivel de azúcar en la sangre (ruchuq'a' kab' pa kïk') durante los últimos 3 meses. La meta para las personas con diabetes (kab'yab'il) es que el A1C sea menos de 6.5.

- Bajar la presión arterial alta (jotöl ruchuq'a' kïk'). La presión arterial (ruchuq'a' kïk') alta hace que su corazón trabaje demasiado fuerte. Aumenta el riesgo (retzelal) de tener un ataque al corazón (rukamik k'uxaj) o derrame cerebral (rukamik tzatzq'or) y de dañar los riñones y los ojos. La meta para las personas con diabetes (kab'yab'il) es que la presión arterial (ruchuq'a' kïk') sea menos de 130/80 mm Hg.
- Mantener el colesterol a niveles normales (taq choj ruchuq'a' saqab'uyül q'anal). El colesterol malo (LDL) (itzel saqab'uyül q'anal) se acumula y tapa las arterias (taq kaqib'och'ilal). Puede causar un ataque al corazón (rukamik k'uxaj) o derrame cerebral (rukamik tzatzq'or). La meta para las personas con diabetes (kab'yab'il) es que el colesterol (LDL) (itzel saqab'uyül q'anal) sea menor de 100 mg/dl.

PREGUNTE:

· ¿Cómo afecta la diabetes (kab'yab'il) al cuerpo?

RESPUESTA: La diabetes (kab'yab'il) puede causar daño al corazón, cerebro, riñones y ojos. También puede causar daño a los nervios (k'amab'äl) lo que reduce la sensación en los pies (na'onem pa aqanaj). De igual manera, la diabetes (kab'yab'il) puede afectar el flujo de la sangre (rutz'ujal kïk') a las piernas y pies. Esto puede resultar en heridas (taq sokotajïk) o llagas (taq nimach'a'k) que no se sanan y a la amputación (qupinem).

TAREA SEMANAL

DIGA:

· Han aprendido mucho hoy sobre cómo prevenir la diabetes (kab'yab'il). Ahora veamos cómo pueden poner en práctica lo que han aprendido. Piensen en un cambio que puedan hacer en su vida diaria para prevenir o controlar la diabetes (kab'yab'il). Esto será su tarea semanal.

DIGA:

· Sean específicos sobre qué planean hacer, cómo lo planean hacer y cuándo comenzarán. Veamos algunos ejemplos:

  · Me chequearé el nivel del azúcar en la sangre este mes.
  · Esta semana, tomaré agua con el almuerzo en lugar de gaseosas u otras bebidas con azúcar.

PREGUNTE:

HAGA : *Escriba las ideas para tareas en el pizarrón o en un papelógrafo.*

· ¿Quién quiere compartir con los demás la tarea que hizo?

(OPCIONAL) DIGA:

· El mantener en mente un valor personal puede ayudarles a tomar acciones para reducir el riesgo (retzelal) de desarrollar diabetes (kab'yab'il) o de controlarla si ya tienen la enfermedad. Recuerden que un valor personal es una cualidad que consideran importante.

(OPCIONAL) DIGA:

· Nuestro valor para hoy es la alegría o gozo. La alegria puede reemplazar el aburrimiento, la indiferencia y la depresión con un pensamiento y sentimiento positivo. La alegría les puede ayudar a que gocen al preparar alimentos de manera saludable. La alegría es contagiosa y otros pueden seguir su ejemplo.

NOTA : *Dé a los participantes 3 minutos para que compartan sus pensamientos.*

(OPCIONAL) PREGUNTE:

· ¿Cómo pueden usar la alegría u otro valor, para ayudarles a cumplir con su tarea?

DIGA:

· La próxima semana hablaremos sobre cómo les fue con sus tareas. No se olviden de continuar con sus tareas de comer menos sal, grasa saturada (ritzelal q'anal), grasa transaturada (nima'itzelal q'anal) y colesterol (saqab'uyül q'anal), de lograr y mantener un peso saludable de hacer más actividad física.

CIERRE DE LA SESIÓN

DIGA:

· Muchas gracias por asistir hoy ¿Qué les pareció esta sesión? Espero verlos en la próxima sesión donde hablaremos sobre como gozar con su familia de comidas saludables para el corazón.

DIGA:

  · En la proxima sesión vamos a repasar lo que han aprendido. Por favor traigan sus hojas de cada una de las clases.

NOTA : *Reflexión para el presentador de la sesión. Piense en la sesión de hoy. ¿Qué cree usted que funcionó y qué no funcionó?*

¿ESTÁ EN RIESGO DE DESARROLLAR DIABETES TIPO 2?

Piense en los factores de riesgo que tiene. Mientras más factores de riesgo tiene, mayor es su riesgo para desarrollar la diabetes tipo 2. Solamente su médico puede determinar si tenga diabetes. En su próxima consulta, ¡averígüelo con certeza!

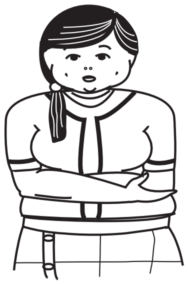

- ¿Tiene sobrepeso? (especialmente si tiene mucho peso alrededor de la cintura)

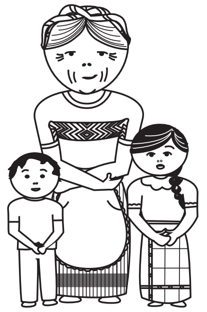

- Alguien de su familia inmediata (padre, madre, hermano o hermana) tiene diabetes

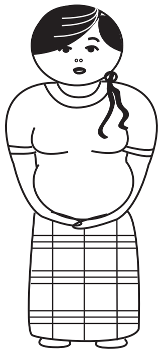

- Ha tenido diabetes gestacional

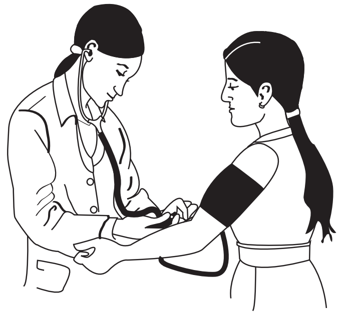

- Su presión arterial es de 140/90 mm Hg o más, o el médico le ha dicho que tiene la presión arterial alta

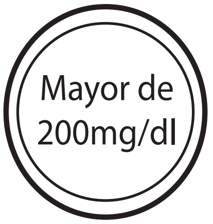

- Sus niveles de colesterol no son normales. Su colesterol HDL (el colesterol “bueno”) es de 35 mg/dl o menos, o su nivel de triglicéridos es de 250 mg/dl o más.

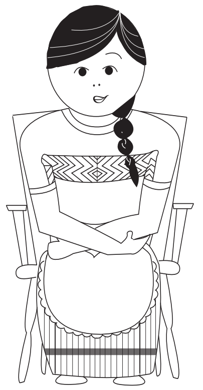

- Hace muy poca actividad física. Hace actividad física menos de 3 veces por semana.

LOS SÍNTOMAS DE LA DIABETES

Los síntomas de la diabetes tipo 2 se desarrollan gradualmente. Algunas personas no tienen síntomas y otras sí. Algunos de los síntomas de la diabetes son:

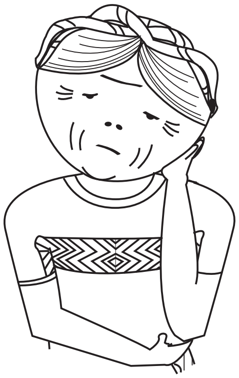

- Cansancio

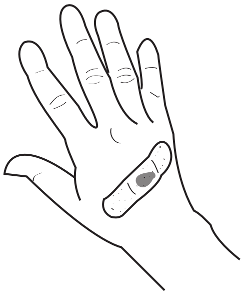

- Heridas o llagas que no se curan

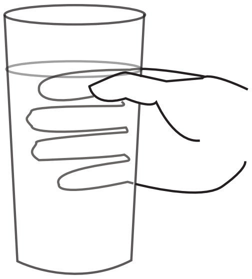

- Sentir mucha sed

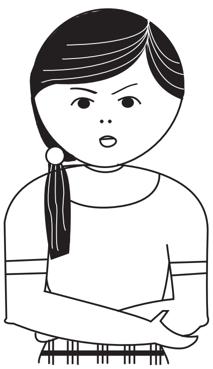

- Irritabilidad

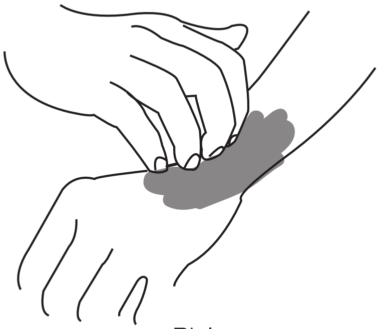

- Piel muy seca

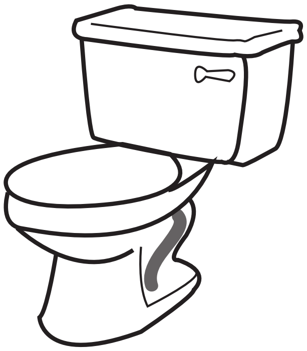

- Orinar frecuentemente

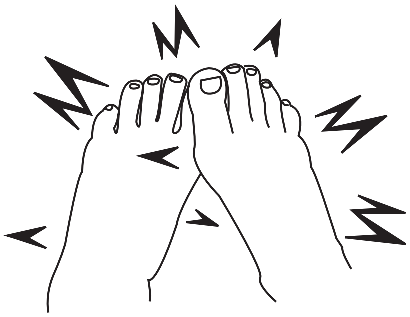

- Sensación de hormigueo en los pies

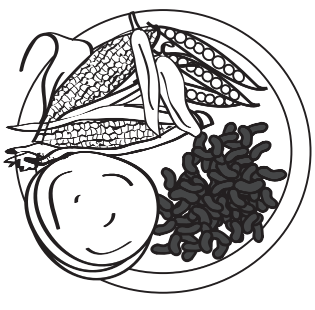

- Sentir mucha hambre

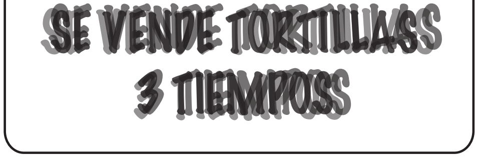

- Visión borrosa

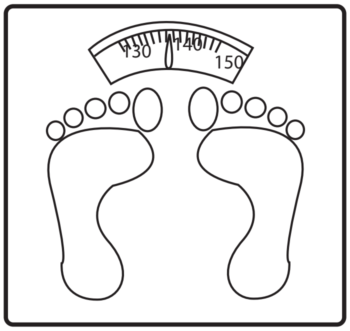

- Pérdida inexplicable de peso

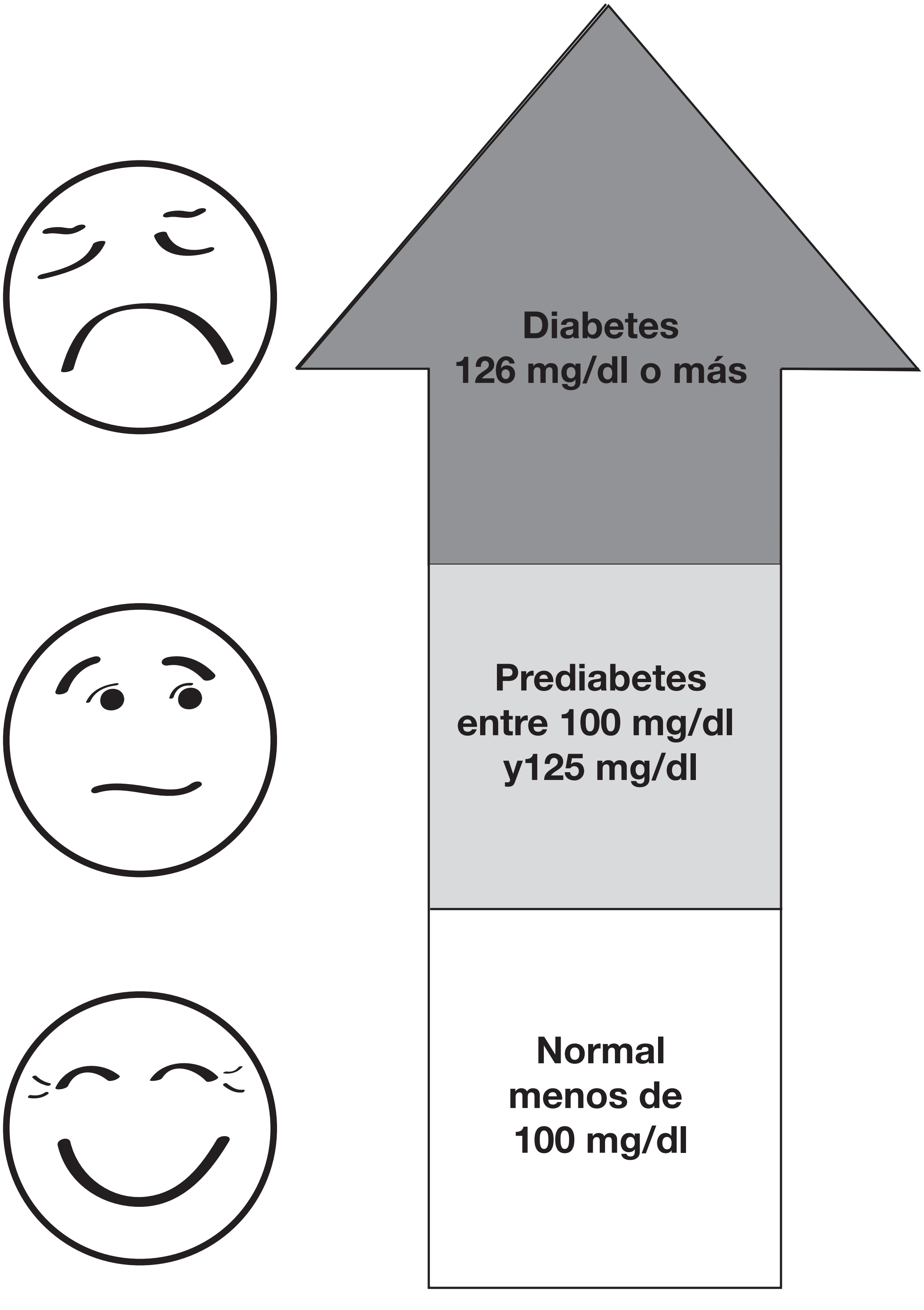
Diabetes
126 mg/dl o más
Prediabetes
entre 100 mg/dl
y125 mg/dl
Normal
menos de
100 mg/dl

6.2

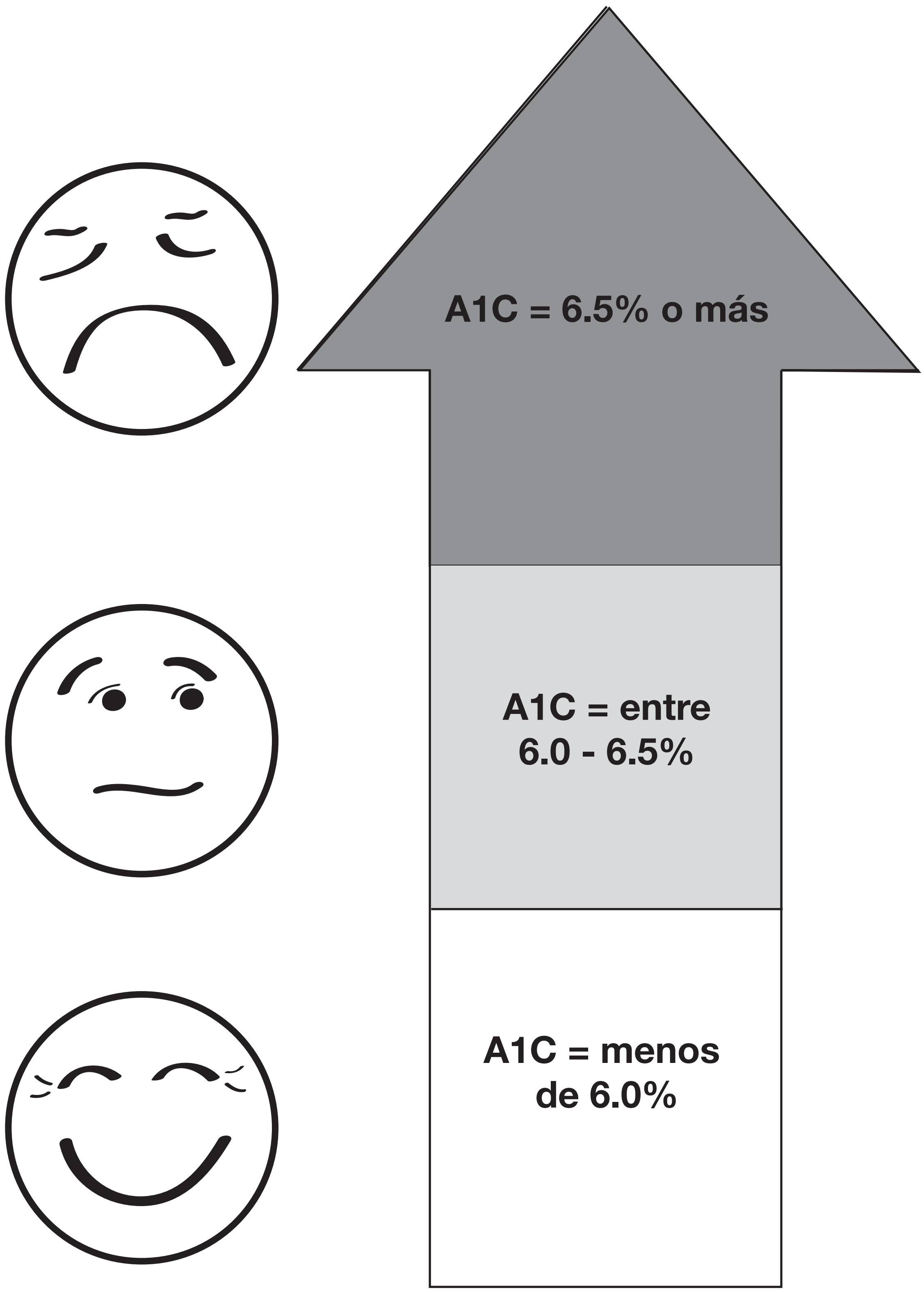

EL BUEN CUIDADO DE SUS PIES

CUIDADO DIARIO

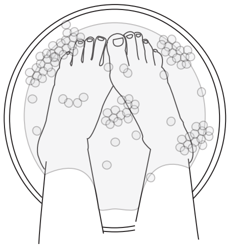

- Lávese los pies en agua tibia todos los días. Séqueselos con cuidado, especialmente entre los dedos.

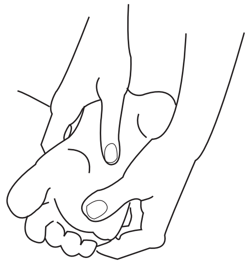

- Examine sus pies todos los días para ver si tiene heridas, llagas, ampollas, enrojecimiento, callos u otros problemas. Si no puede agacha se o alzar las piernas para ver la planta de los pies, use un espejo o pida a alguien que le haga el favor de revisarle los pies.

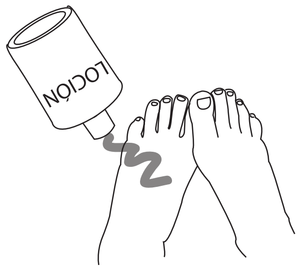

- Si tiene la piel seca, póngase loción en los pies después de lavarlos y secarlos. No se ponga loción entre los dedos de los pies.

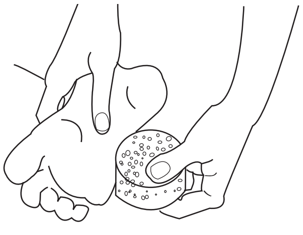

- Después de bañarse, utilice una lima de cartón o piedra pómez para suavizar los callos delicadamente. No use navaja de bolsillo ni hojillas de afeitarse que le puedan cortar la piel.

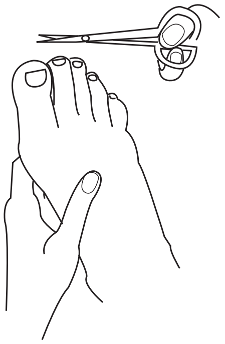

- Córtese las uñas de los pies cada semana después de bañarse.

EXAMEN PERIÓDICO DE LOS PIES

- Recuerde a su médico que le revise los pies en cada visita.
- Hágase un examen completo de los pies una vez al año. Si tiene problemas con sus pies, pídale a su proveedor de cuidados de la salud que se los examine cada 3 ó 6 meses.

CALZADO

- Use calcetines o medias y zapatos bien acolchonados en todo momento. No ande descalzo.
- Cámbiese los calcetines o medias todos los días para mantener sus pies limpios y suaves.
- Compre zapatos que sean amplios y le permitan "respirar" a sus pies.

CUIDE SU CORAZÓN: CONOZCA LOS PASOS CLAVES PARA CONTROLAR LA DIABETES

Algunas personas tienen "prediabetes". La prediabetes ocurre cuando los niveles de azúcar en la sangre son más altos de lo normal pero aún no están en el nivel de la diabetes. Las personas con esta condición pueden reducir el riesgo de desarrollar diabetes bajando un poco de peso y aumentando su actividad física.

Si usted tiene diabetes, puede ayudar a evitar tener complicaciones controlando sus niveles de azúcar en la sangre.

LOS PASOS CLAVE PARA CONTROLAR LA DIABETES

Si tiene diabetes, hay tres pasos clave que le pueden ayudar a disminuir su riesgo de un ataque al corazón o derrame cerebral. Siga estos pasos:

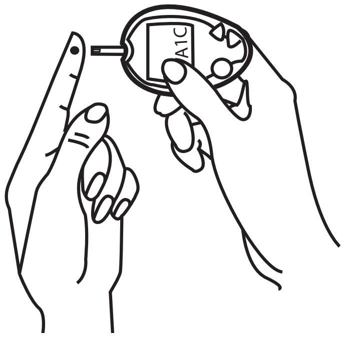

- Es necesario hacerse la prueba de sangre llamada A1C por lo menos dos veces al año. Esta prueba sirve para medir como ha controlado el nivel de azúcar en la sangre durante los últimos 3 meses. La meta para las personas con diabetes es que el A1C sea menos de 7.

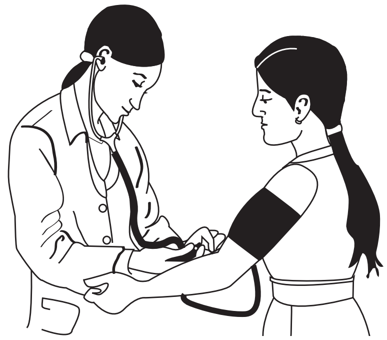

- Baje la presión arterial alta. La presión arterial alta hace que su corazón trabaje demasiado fuerte. Aumenta su riesgo para un ataque al corazón o derrame cerebral, dañar los riñones y los ojos. Mídase su presión arterial en cada visita al médico. La meta para las personas con diabetes es que la presión arterial sea menos de 130/80 mm Hg.

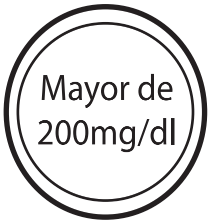

- Mantenga el colesterol a niveles normales. LDL se acumula y tapa las arterias. Puede causar un ataque al corazón o derrame cerebral. Mídase LDL por lo menos una vez al año. La meta para las personas con diabetes es que el LDL sea menor de 100mg/dl.

NO SE OLVIDE DE PREGUNTAR AL MÉDICO: DESAYUNO

- ¿Cuáles son mis niveles de la prueba de A1C, presión arterial y colesterol?
- ¿Cuáles deberían ser mis niveles para estas pruebas?
- ¿Qué pasos debo tomar para alcanzar los niveles recomendados?

Anote los cambios que hará esta semana para controlar el nivel de azúcar y colesterol en la sangre y la presión arterial.

DESCUBRA EL AZÚCAR EN SU BEBIDA FAVORITA*

Bebida (porción de 12 onzas)	Gramos de azúcar	Número aproximado de cucharaditas de azúcar
Agua pura	0 gramos	0 cucharaditas
Super cola	41 gramos	10 1⁄4 cucharaditas
Gaseosa dietética	0 gramos	0 cucharaditas
Jugo de guayaba	48 gramos	12 cucharaditas
Jugo de mango	50 gramos	12 1⁄2 cucharaditas
Horchata	33 gramos	8 1⁄4 cucharaditas
Gaseosa sabor de naranja	52 gramos	13 cucharaditas
Bebida azucarada en polvo (Tang, Sprim, Flash, Yus de Toky)	36 gramos	9 cucharaditas
Limonada con azúcar	25 gramos	6 1⁄4 cucharaditas
Limonada sin azúcar	0 gramos	0 cucharaditas
Jugo de uva	32 gramos	8 cucharaditas
Jugo de naranja	20 gramos	5 cucharaditas
Ponche de frutas	46 gramos	11 1⁄2 cucharaditas
Bebida deportiva (Gatorade)	8 gramos	2 cucharaditas
Té con azúcar	33 gramos	8 1⁄2 cucharaditas
Té sin azúcar	0 gramos	0 cucharaditas

*Nota: Muchas bebidas contienen un nivel más alto azúcar la que aparece en este listado.

SESIÓN 7 Repaso y Graduación

OBJETIVOS

AL FINALIZAR ESTA SESIÓN, LOS PARTICIPANTES HABRÁN REPASANDO TODAS LAS SESIONES Y RECIBIRÁN SUS DIPLOMAS.

MATERIALES

PARA DIRIGIR ESTA SESIÓN USTED NECESITARÁ:

- Pizarrón, marcadores, almohadilla, varias hojas grandes de papel y cinta adhesiva
- Diplomas con el nombre del participante y la firma.*

* *Prepare antes de la sesión. Escribe el nombre del participante y firme cada diploma.*

HOJAS PARA REPASAR:

DURANTE LA SESIÓN, REPASE ESTAS HOJAS:

- "Factores de riesgo para las Enfermedades del Corazón que Usted Puede Controlar" (página 11)
- "Conozca los síntomas de un derrame cerebral. Actúe rápidamente." (página 64)
- "Se necesitan las tres grupos para una comida saludable" (página 44)
- "Tamaño de las Porciones" (página 45)
- "Use hierbas e especies en lugar de sal." (página 66)
- "¿Está en riesgo de desarrollar diabetes tipo 2?" (página 101)
- "Los síntomas de la diabetes" (página 102)
- "El buen cuidado de sus pies" (página 105)

CARTELES PARA MOSTRAR:

- Cartel derivado de la hoja "Lo que se siente al tener un ataque al corazón" (página 24)
- Cartel derivado de la hoja "La acción rápida salva vidas" (página 25)
- Cartel derivado de la hoja "Se necesitan los tres grupos para una comida saludable" (página 44)
- Cartel derivado de la hoja "Tamaño de las Porciones" (página 45)
- Cartel derivado de "Hábitos saludables contra la presión arterial alta" (página 63)
- Cartel derivado de dibujo educativo 5.1 (página 81)
- Cartel derivado de dibujo educativo 5.2 (página 82)
- Cartel derivado de la hoja "¿Está en riesgo de desarrollar diabetes
- tipo 2?" (página 101)
- Cartel derivado de la hoja "Los síntomas de la diabetes" (página 102)
- Cartel derivado de dibujo educativo 6.1 (página 103)
- Cartel derivado de dibujo educativo 6.2 (página 104)
- Cartel derivado de la hoja "El buen cuidado de sus pies" (página 105)

ESQUEMA DE LA SESIÓN

INTRODUCCIÓN A LA SESIÓN

1. Bienvenida
2. Acerca de esta sesión

LA SESIÓN EN ACCIÓN

Repaso de la sesión 1: ¿Está usted en riesgo de desarrollar enfermedades del corazón (taq ruyab'ilal k'uxaj)?
Repaso de la sesión 2: Actúe rápido frente a los síntomas (taq k'u't) de un ataque al corazón (rukamik k'uxaj).
Repaso de la sesión 3: Piense en su corazón: Dígale Sí a la alimentación saludable y la actividad física.
Repaso de la sesión 4: Controle su presión arterial alta (jotöl ruchuq'a' kïk').
Repaso de la sesión 5: Sea bueno con su corazón: Mantenga su colesterol bajo control (chöj ruchuq'a' ri saqab'uyül q'anal).
Repaso de la sesión 6: Proteja su corazón: Cuidese de la diabetes (kab'yab'il).

GRADUACIÓN

CIERRE DE LA SESIÓN

INTRODUCCIÓN A LA SESIÓN

1 | BIENVENIDA

- Dé la bienvenida a los participantes a la sesión.

2 | ACERCA DE ESTA SESIÓN

DIGA:

· En la sesión de hoy hablaremos sobre la alimentación saludable y la actividad física y lo importante que son éstos para la salud del corazón (raxnaqil k'uxaj). Cuando termine la sesión, ustedes sabrán:

  · Identificar los grupos de alimentos y entender cómo actúan de maneras diferentes en el cuerpo
  · Escoger una variedad de alimentos para tener un cuerpo sano
  · Reconocer los tamaños correctos de las porciones y cuántas porciones deben comer de cada grupo de alimentos
  · Explicar las características básicas de una dieta saludable y planear comidas adecuadas
  · Cómo ustedes y sus familias se pueden beneficiar de la actividad fisica
  · Qué tipos de actividades físicas son buenas para ustedes y para su corazón

LA SESIÓN EN ACCIÓN

NOTA : *Los participantes pueden ver la siguiente hoja después las preguntas: "Factores de riesgo para las Enfermedades del Corazón que Usted Puede Controlar" (página 11).*

REPASO DE SESIÓN 1 | ¿ESTÁ USTED EN RIESGO DE DESARROLLAR ENFERMEDADES DEL CORAZÓN (TAQ RUYAB'ILAL K'UXAJ)?

DIGA:

· Ahora repasemos lo que han aprendido sobre las enfermedades del corazón (taq ruyab'ilal k'uxaj) en la primera clase.

PREGUNTE:

· ¿Cuáles son los factores de riesgo (rub'anikil rutzilal) para enfermedades del corazón (taq ruyab'ilal k'uxaj) qué nosotros podemos prevenir o controlar?

  AÑADA LOS SIGUIENTES FACTORES SI NADIE LOS MENCIONA:
  · la presión arterial alta (jotöl ruchuq'a' kïk')
  · un nivel alto de colesterol (jotöl ruchuq'a' saqab'uyül q'anal) en la sangre
  · el hábito de fumar
  · la diabetes (kab'yab'il)
  · el sobrepeso
  · la inactividad física

HAGA : *Muestre los siguientes carteles:*
· Cartel derivado de dibujo educativo 2.2 "Lo que se siente al tener un ataque al corazón" (página 24)
· Cartel derivado de dibujo educativo 2.3 "La acción rápida salva vidas" (página 25)

REPASO DE SESIÓN 2 | ACTÚE RÁPIDO FRENTE A LOS SÍNTOMAS (TAQ K'U'T) DE UN ATAQUE AL CORAZÓN (RUKAMIK K'UXAJ).

DIGA:

· Ahora repasemos lo que han aprendido como actúe rápido frente a los síntomas (taq k'u't) de un ataque al corazón (rukamik k'uxaj).

PREGUNTE:

· ¿Qué es un ataque al corazón (rukamik k'uxaj)?

RESPUESTA: Un ataque al corazón (rukamik k'uxaj) ocurre cuando una arteria (kaqib'öch') se obstruye y el flujo de sangre (rutz'ujal kïk') al corazón se corta. Si el flujo de sangre (rutz'ujal kïk') se corta, partes del músculo del corazón (ruti'ojilal k'uxaj) comienzan a morirse.

PREGUNTE:

· ¿Qué detiene un ataque al corazón (rukamik k'uxaj)?

RESPUESTA: Acción y tratamiento médico rápido restauran el flujo de sangre (rutz'ujal kïk') y salvan al músculo del corazón (ruti'ojilal k'uxaj).

PREGUNTE:

· ¿Por qué es importante obtener tratamiento rápidamente (aninäq to'ïk)?

RESPUESTA: El tratamiento rápido (aninäq to'ïk) puede evitar que parte del músculo del corazón (ruti'ojilal k'uxaj) se muera.

- Es mejor recibir tratamiento dentro de la primera hora después de que comienzan los síntomas (taq k'u't). Esto mejorará la probabilidad de sobrevivir y ayudará a salvar el músculo del corazón (ruti'ojilal k'uxaj).
- Hay medicamentos para disolver los coágulos (ch'ojch'äq), que abren la arteria (kaqib'öch') y restauran el flujo de sangre (rutz'ujal kïk').

PREGUNTE:

· ¿Cuáles son algunos de los síntomas (taq k'u't) de un ataque al corazón (rukamik k'uxaj)?

RESPUESTA: presión en el pecho

- molestia en uno o ambos brazos, la espalda o el estómago
- molestia en el cuello o la mandíbula
- dificultad para respirar con molestia o sin molestia en el pecho
- malestar en el estómago o náusea
- mareo o sudor frío

PREGUNTE:

· ¿Qué deberán hacer si experimentan estos síntomas?

RESPUESTA: Llamen a los bomberos en 5 minutos, aún si no están seguros de tener un ataque al corazón. Cuando llaman al 122 o 123, reciben tratamiento más rápido.

- No conduzca su carro al hospital.

HAGA : *Muestre los siguientes carteles:*
· Cartel derivado de la hoja "Se necesitan los tres grupos para una comida saludable" (página 44). Los participantes pueden repasar con la hoja.
· Cartel derivado de la hoja "Tamaño de las Porciones" (página 45). Los participantes pueden repasar con la hoja.(página 45).

NOTA : *Los participantes pueden ver las siguientes hojas después las preguntas:*
· "Se necesitan los tres grupos para una comida saludable" (página 44)
· "Tamaño de las Porciones" (página 45)

REPASO DE SESIÓN 3 | PIENSE EN SU CORAZÓN: DÍGALE SÍ A LA ALIMENTACIÓN SALUDABLE Y LA ACTIVIDAD FÍSICA.

DIGA:

- Ahora repasemos lo que han aprendido sobre la alimentación saludable y la actividad física.

PREGUNTE:

- ¿Que necesita para crear un plato equilibrado (chojalin läq)?

RESPUESTA: Un plato equilibrado (chojalin läq) debe estar dividido en tres partes, como lo demuestra el cartel, indicando los tres grupos más importantes:

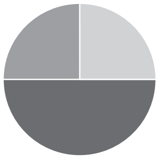

- Si se dan cuenta, las grasas (taq q'anal) no se incluyen como uno de los grupos principales, ya que generalmente cuando se usa aceita vegetal (ichajilal yaq'anal), o mantequilla (qasq'anal) para cocinar, se consumen las porciones adecuadas. Además, hay varias comidas que naturalmente tienen un poco de grasa (q'anal), como el huevo, la carne, y el pollo, entre otros, y no es necesario consumir grandes cantidades para cumplir con lo que el cuerpo necesita. Por eso, no se cuentan como un grupo aparte en el cartel.

PREGUNTE:

- ¿Cuáles alimentos debemos comer con menos frecuencia?

RESPUESTA: Deberíamos limitar alimentos que tienen un alto contenido de grasa (q'anal), sal y azúcar, incluyendo:

- alimentos con alto contenido de grasa (q'anal) como frijoles refritos, pollo frito y cortes de carne grasosos
- alimentos salados, como las papas fritas y los nachos
- alimentos con alto contenido de grasa (q'anal) y de azúcar como pastelería, galletas y chocolate
- alimentos con mucha azúcar como dulces y bebidas gaseosas

PREGUNTE:

- ¿Por qué es útil saber cuántas porciones se debe comer de cada uno de los principales grupos de alimentos?

RESPUESTA: Cuando se sabe la cantidad de alimentos que se debe comer cada día, es más fácil controlar la cantidad de grasa (q'anal) que se consume.

PREGUNTE:

- ¿Cómo creen ustedes que la actividad física les puede ayudar?

RESPUESTA: La actividad física realizada regularmente puede ayudar a:

- fortalecer el corazón y los pulmones
- formar y mantener huesos, músculos (taq ti'ojilal) y articulaciones saludables (taq raxnaqil laqul)
- controlar el peso
- tener más energía (uchuq'ab'il)
- sentirse mejor acerca de sí mismo
- reducir el estrés (sachk'u'x)
- disminuir los sentimientos de depresión y ansiedad
- dormir mejor

PREGUNTE:

· ¿Cuál es la actividad que casi todos pueden hacer?

RESPUESTA: caminar a paso ligero

HAGA : *Muestre el cartel:*
· Cartel derivado de "Hábitos saludables contra la presión arterial alta" (página 63).

NOTA : *Los participantes pueden ver las siguientes hojas después las preguntas:*
· "Conozca los síntomas de un derrame cerebral. "Actúe rápidamente." (página 64)
· "Use hierbas e especies en lugar de sal." (página 66)

REPASO DE SESIÓN 4 | CONTROLE SU PRESIÓN ARTERIAL ALTA (JOTÖL RUCHUQ'A' KÏK').

DIGA:

· Ahora repasemos lo que han aprendido como controle su presión arterial alta (jotöl ruchuq'a' kïk').

PREGUNTE:

· ¿Qué es la presión arterial (ruchuq'a' kïk')?

RESPUESTA: La presión arterial (ruchuq'a' kïk') es la fuerza que ejerce la sangre contra las paredes de las arterias (kaqib'och'ilal). La presión arterial (ruchuq'a' kïk') es necesaria para que la sangre circule por el cuerpo.

PREGUNTE:

· ¿Qué es una presión arterial normal (choj ruchuq'a' kïk')?

RESPUESTA: La presión arterial normal (choj ruchuq'a' kïk') es menor de 120/80 mm Hg.

PREGUNTE:

· ¿Qué es la [prehipertensión]?

RESPUESTA: La presión arterial (ruchuq'a' kïk') entre 120/80 y 139/89 mm Hg se llama [prehipertensión]. Esto quiere decir que aún no tiene presión arterial alta (jotöl ruchuq'a' kïk'), pero es probable que la desarrolle en el futuro a menos que haga cambios en sus [hábitos de salud].

PREGUNTE:

· ¿Qué es la presión arterial alta (jotöl ruchuq'a' kïk')?

RESPUESTA: La [presión arterial alta] es de 140/90 mm Hg o más. Mídase su presión arterial alta (jotöl ruchuq'a' kïk').

PREGUNTE:

· ¿Por qué es peligrosa la presión arterial alta (jotöl ruchuq'a' kïk')?

RESPUESTA: La presión arterial alta (jotöl ruchuq'a' kïk') puede causar un ataque al corazón (rukamik k'uxaj) o [derrame], problemas de los riñones o de los ojos y la muerte.

PREGUNTE:

· ¿Qué pueden hacer para ayudar a que funcionen mejor sus medicamentos para la presión arterial alta (jotöl ruchuq'a' kïk')?

RESPUESTA: Comer más frutas y verduras y menos sal, tomar más agua, perder peso y hacer más actividad física. Tomar los medicamentos siguiendo las instrucciones delmédico. Avisarle al médico si los medicamentos le hacen sentir enfermo.

PREGUNTE:

· ¿Por qué es importante reducir la sal en la comida?

RESPUESTA: Es importante comer menos sal para ayudar a prevenir o bajar la presión arterial (ruchuq'a' kïk').

PREGUNTE:

· ¿Cuáles son algunas maneras para reducir la sal?

RESPUESTA: Usar hierbas e especies para sazonar los alimentos pero ¡tenga cuidado! Algunos sazonadores como la sal de ajo o de cebolla tienen un contenido alto de sal. Fijarse en la etiqueta de los alimentos para escoger los que tienen menos sal. Comer más frutas y verduras entre las comidas en lugar de bocadillos salados como nueces, ricitos, tortrix, Doritos y plataninas. También consumir bebidas alcohólicas afecta la presión arterial (ruchuq'a' kïk').

· Beber demasiadas bebidas alcohólicas puede elevar la presión arterial (ruchuq'a' kïk').

PREGUNTE:

· ¿Qué es un derrame cerebral (rukamik tzatzq'or)?

RESPUESTA: Un derrame cerebral (rukamik tzatzq'or) ocurre cuando la sangre repentinamente deja de llegar al cerebro. En el derrame cerebral, un vaso sanguíneo (rub'ey kïk') que va al cerebro se rompe.

DIGA:

· Pero el resultado es igual, las células (taq rujotay k'aslem) del cerebro no reciben oxígeno (uxlab'il) y se mueren.

PREGUNTE:

· ¿De qué otra manera se conoce derrame cerebral (rukamik tzatzq'or)?

RESPUESTA: Un derrame cerebral (rukamik tzatzq'or) es un problema muy grave que se conoce por varios nombres como ataque al cerebro, embolia cerebral, apoplejía o accidente cerebrovascular.

HAGA : *Muestre los siguientes carteles:*
· Cartel derivado de dibujo educativo 5.1 (página 81)
· Cartel derivado de dibujo educativo 5.2 (página 82)

REPASO DE SESIÓN 5 | SEA BUENO CON SU CORAZÓN: MANTENGA SU COLESTEROL BAJO CONTROL (CHOJ RUCHUQ'A' RI SAQAB'UYÜL Q'ANAL).

DIGA:

· Ahora repasemos lo que han aprendido como mantenga su colesterol bajo control (chöj ruchuq'a' ri saqab'uyül q'anal).

PREGUNTE:

· ¿Cuál puede ser la causa de que las arterias (kaqib'och'ilal) se obstruyan?

RESPUESTA: la acumulación del colesterol (saqab'uyül q'anal) en las arterias (kaqib'och'ilal) las obstruye

PREGUNTE:

· ¿Cuál es el nivel total de colesterol (saqab'uyül q'anal) en la sangre que se considera deseable?

RESPUESTA: menos de 200 mg/dl

PREGUNTE:

· ¿Qué medidas pueden tomar para mantener [bajo el nivel de colesterol (qajnäj saqab'uyül q'anal) en la sangre?

RESPUESTA: comer alimentos con bajo contenido de grasa saturada (ritzelal q'anal), grasa transaturada (nima'etzelal q'anal) y colesterol (saqab'uyül q'anal)

· mantener un peso saludable
· mantenerse físicamente activo
· comer una variedad de granos integrales, frutas y verduras

PREGUNTE:

· ¿Pueden nombrar algunos alimentos con alto contenido de grasa saturada (ritzelal q'anal)?

RESPUESTA: los cortes de carne grasosos, el pellejo del pollo, la leche entera y la mantequilla (qasq'anal)

PREGUNTE:

· ¿Cuáles son algunas de las cosas que pueden hacer para que los alimentos tengan menos grasa (q'anal)?

RESPUESTA: quitar la grasa (q'anal) de la carne antes de cocinarla

· quitar el pellejo del pollo o pavo y no comérselo
· hornear, hervir, azar o cocinar a la parrilla los alimentos en lugar de freírlos
· quitar la grasa (q'anal) de la sopa o caldo antes de servirlo

PREGUNTE:

· ¿Pueden nombrar algunos alimentos saludables para el corazón?

RESPUESTA: frutas como naranjas, peras, manzanas, duraznos y bananos

· verduras como repollo, brócoli, zanahorias, alcachofa y habas verdes
· legumbres como arvejas y frijoles
· granos de cereal integral como la avena

HAGA : *Muestre los siguientes carteles:*
· Cartel derivado de la hoja "¿Está en riesgo de desarrollar diabetes tipo 2?" (página 101)
· Cartel derivado de la hoja "Los síntomas de la diabetes" (página 102)
· Cartel derivado de dibujo educativo 6.1 (página 103)
· Cartel derivado de dibujo educativo 6.2 (página 104)
· Cartel derivado de la hoja "El buen cuidado de sus pies" (página 105)

NOTA : *Los participantes pueden ver las siguientes hojas después las preguntas:*
· "¿Está en riesgo de desarrollar diabetes tipo 2?" (página 101)
· "Los síntomas de la diabetes" (página 102)
· "El buen cuidado de sus pies" (página 105)

REPASO DE SESIÓN 6 | PROTEJA SU CORAZÓN: CUIDESE DE LA DIABETES (KAB'YAB'IL).

DIGA:

· Ahora repasemos lo que han aprendido sobre la la diabetes (kab'yab'il).

PREGUNTE:

- ¿Que es la diabetes (kab'yab'il)?

RESPUESTA: La diabetes (kab'yab'il) ocurre cuando el cuerpo no puede controlar la cantidad alta de azúcar en la sangre (jotöl ruchuq'a' kab' pa kïk') y eso puede danar a las otras partes del cuerpo, por ejemplo los riñones, ojos y corazón.

PREGUNTE:

- ¿Cuales son los factores de riesgo (rub'anikil rutzilal) para la diabetes (kab'yab'il)?

RESPUESTA: Entre los factores de riesgo (rub'anikil rutzilal) para la diabetes (kab'yab'il) están:

- miembros de la familia que tienen diabetes (kab'yab'il)
- el sobrepeso
- tener edad avanzada
- hacer poca actividad física
- haber tenido diabetes gestacional (yawa' kab'yab'il)
- la presión arterial (ruchuq'a' kïk')
- niveles anormales de colesterol (saqab'uyül q'anal); cuando el colesterol bueno (ütz saqab'uyül q'anal) está bajo o el nivel de triglicéridos (ruwäch meq'anal) está alto

PREGUNTE:

- ¿Cómo se puede prevenir o retrasar el comienzo de la diabetes tipo 2 (ruka'n ruwäch kab'yab'il)?

RESPUESTA: Se puede prevenir o retrazar el comienzo de la diabetes tipo 2 (ruka'n ruwäch kab'yab'il):

- Siguen un plan de alimentación saludable y hacen actividad física.

PREGUNTE:

- ¿Cuáles son los pasos claves para controlar la diabetes (kab'yab'il)?

RESPUESTA: Es necesario hacerse la prueba de sangre llamada A1C por lo menos dos veces al año. Esta prueba sirve para medir como ha controlado el nivel de azúcar en la sangre durante los últimos 3 meses. La meta para las personas con diabetes (kab'yab'il) es que el A1C sea menos de 6.5.

- Bajar la presión arterial (ruchuq'a' kïk'). La presión arterial (ruchuq'a' kïk') hace que su corazón trabaje demasiado fuerte. Aumenta el riesgo de tener un ataque al corazón (rukamik k'uxaj) o derrame cerebral (tzaqnäq ruchuq'a tzatzq'or) y de dañar los riñones y los ojos. La meta para las personas con diabetes (kab'yab'il) es que la presión arterial (ruchuq'a' kïk') sea menos de 130/80 mm Hg.
- Mantener el colesterol a niveles normales (taq choj ruchuqa' saqab'uyul q'anal). El colesterol malo LDL (nimak'wanel q'anal) se acumula y tapa las arterias (kaqib'och'ilal). Puede causar un ataque al corazón (rukamik k'uxaj) o derrame cerebral (rukamik tzatzq'or). La meta para las personas con diabetes (kab'yab'il) es que el colesterol LDL (nimak'wanel q'anal) sea menor de 100 mg/dl.

PREGUNTE:

- ¿Cómo afecta la diabetes (kab'yab'il) al cuerpo?

RESPUESTA: La diabetes (kab'yab'il) puede causar daño al corazón, cerebro, riñones y ojos. También puede causar daño a los nervios (k'amab'äl) lo que reduce la sensación en los pies. De igual manera, la diabetes (kab'yab'il) puede afectar el flujo de la sangre (rutz'ujal kïk') a las piernas y pies. Esto puede resultar en heridas o llagas que no se sanan y a la amputación (qupinem).

GRADUACIÓN

HAGA : *Dé a cada participante su diploma con la firma.*

DIGA:

- Felicidades a ustedes! Han completado la capacitación acerca de la salud del corazón (raxnaqil k'uxaj).

CIERRE DE LA SESIÓN

NOTA : *Espere un rato a ver si los participantes tienen algún comentario.*

DIGA:

- Gracias por asistir el día de hoy.
- ¿Qué comentarios me pueden dar acerca del proyecto?

DIGA:

- Piense en el proyecto.

PREGUNTE:

- ¿Qué cree usted que funcionó y qué no funcionó?
- ¿Qué cambió en su vida como resultado de lo que aprendió en el proyecto?
